# Selection of timing of continuous renal replacement therapy in patients with acute kidney injury: A meta-analysis of randomized controlled trials

**DOI:** 10.1371/journal.pone.0320351

**Published:** 2025-03-25

**Authors:** Jiawei Qi, Wenwen Wu, Jingzhu Wang, Xin Guo, Chengyun Xia

**Affiliations:** 1 Department of Nephrology, Affiliated Hospital of North Sichuan Medical College, Nanchong, Sichuan, China; 2 Department of Clinical Medicine, North Sichuan Medical College, Nanchong, Sichuan, China; University of Bari: Universita degli Studi di Bari Aldo Moro, ITALY

## Abstract

Acute kidney injury (AKI) is associated with high death rates and unfavorable outcomes. Previous studies evaluating the effect of the timing of CRRT therapy on the prognosis of patients with AKI have shown inconsistent results. Consequently, we aimed to assess the impact of continuous renal replacement therapy (CRRT) initiation on the outcomes of patients with AKI. This meta-analysis identified eligible randomized controlled trials (RCTs) via comprehensive searches of PubMed, Embase, and the Cochrane databases from their creation until June 1, 2024. Outcomes, including 28-, 60-, and 90-day mortality and adverse event incidence, were compared between the early and delayed CRRT groups post-randomization. Twelve RCTs (n = 1,244) were included. Meta-analysis indicated that early initiation of CRRT did not significantly affect 28-day mortality (RR 0.91 [0.79, 1.06]; p =  0.23; I^2^ =  0). Early CRRT initiation correlated with a shorter length of ICU stay [MD -3.24 (-5.14, -1.35); p = 0.0008; I^2^ = 36%] but did not significantly affect hospital stay duration [MD -7.00 (-14.60, 0.60); p = 0.07; I^2^ = 38%]. The early initiation of CRRT was associated with a significant reduction in RRT dependency at discharge [RR 0.57 (0.32, 0.99); P = 0.05; I^2^ = 0%; P = 0.47]. Compared to delayed CRRT, early CRRT was associated with higher incidence rates of hypotension [RR 1.26 (1.06, 1.50); p = 0.008; I^2^ = 0%], thrombocytopenia [RR 1.53 (1.11, 2.10); p = 0.009; I^2^ = 0%], and hypophosphatemia [RR 3.35 (2.18, 5.15); p < 0.00001; I^2^ = 11%]. Our findings suggest that although early CRRT initiation is associated with short intensive care unit stays and reduced RRT dependence, it has no significant effect on mortality and is in fact associated with higher incidence rates of hypotension, thrombocytopenia, and hypophosphatemia. Therefore, early CRRT should be used clinically with caution and consideration of potential adverse effects.

## Introduction

Acute kidney injury (AKI) is associated with high mortality and unfavorable outcomes, with a rising global incidence [1–3]. Life-threatening consequences associated with AKI, such as metabolic acidosis, pulmonary edema, and hyperkalemia, are treated with renal replacement therapy (RRT). However, RRT can lead to RRT-related complications and increase healthcare resource utilization. Consequently, the optimal initiation timing for RRT without severe complications remains debatable. While several studies have demonstrated that early RRT initiation may offer a survival advantage for patients with AKI [4,5], recent meta-analyses [6–8] have reported conflicting results. Further, there is no comprehensive analysis including all current randomized controlled trials (RCTs) that focus on continuous renal replacement therapy (CRRT) as a treatment option for patients with AKI. While the Cochrane review does include a sub-analysis by modality, it only examines a limited number of outcome metrics and incorporates few studies related to CRRT. Therefore, in this meta-analysis, we aimed to assess the prognosis of patients with AKI by comparing the effects of early versus delayed CRRT commencement.

## Methods

The Preferred Reporting Items for Systematic Reviews and Meta-Analyses (PRISMA 2020) guidelines were followed in the reporting of this meta-analysis (**S1** Checklist).

### Search strategy and selection process

As of June 1, 2024, comprehensive searches were carried out in the PubMed, Embase, and Cochrane databases using a combination of subject terms and free words related to AKI, RRT, timing, and delay. In addition, references from relevant meta-analyses and studies were manually reviewed. **S1 Table** presents detailed search strategies. J.Q. and W.W. independently conducted study search and screening, The search strategy was developed jointly by J.Q. and W.W. resolving discrepancies through discussion.

### Criteria for inclusion and exclusion

The inclusion criteria were as follows: (1) population: patients with AKI of various causes; (2) interventions: early CRRT initiation in the intervention group; (3) comparison: early versus delayed CRRT initiation; (4) outcomes: at least one of the 28-, 60-, 90-, or 14-day mortality; and (5) study type: RCT. The exclusion criteria were as follows: (1) non-randomized studies; (2) lack of a well-defined early versus delayed initiation strategy; (3) initial treatment in the intervention group not being CRRT-related; and (4) repeated analysis of experimental data. No restrictions were placed on the publication language.

### Data gathering and bias risk evaluation

Data were extracted independently by J.Q. and W.W., including the basic characteristics of the included RCTs (authors, year and country of publication, design and setting, patient mean age, scores before randomization [SOFA or APACHE II], number of patients, male-female ratio, type of CRRT initiation), adverse events, and the primary and secondary results. Using the Cochrane risk of bias methodology (RoB 2), J.Q. and W.W. evaluated the risk of bias independently. Discrepancies were resolved through discussion or consultation with J.W.

### Outcomes

The primary outcome was 28-day mortality. Secondary outcomes were mortality at days 14, 60, and 90; ICU and hospital-based mortality; number of patients receiving RRT; number of patients dependent on RRT (at discharge and day 28); length of stay in the ICU and hospital; and mechanical ventilation days. The incidence of adverse events, including bleeding, hypotension, hypophosphatemia, arrhythmia, hypocalcemia, and thrombocytopenia, was also assessed.

### Data analysis

Analyses were carried out using RevMan 5.4. The Mantel–Haenszel method was used to calculate the risk ratios (RRs) and 95% confidence intervals (CIs) for binary variables, whereas the inverse variance method determined the mean differences (MDs) and 95% CIs for continuous variables. Heterogeneity was evaluated using the χ^2^ test and I^2^ statistics. When the p-value was greater than 0.10, and I^2^ was less than 30%, we used the fixed-effects model; in contrast, when the p-value was less than or equal to 0.10, or I^2^ was greater than or equal to 30%, we used the random-effects model. Statistical significance was indicated by p-values less than 0.05. Funnel plots were used to evaluate publication bias. Subgroup analyses were based on baseline characteristics before randomization, including the average age of the participants (≥64 years or < 64 years), cause of AKI (sepsis or other factors), and severity of the condition (SOFA score ≥ 12 or APACHE II score ≥ 25). For continuous variables, where only the median (Md) and interquartile range (IQR) were reported, we used standard approximation methods to obtain the mean (M) and standard deviation (SD) as follows: Md =  M, IQR =  1.35SD. A sensitivity analysis of the primary result was also performed to evaluate the effect of single studies on overall 28-day mortality by systematically excluding individual studies and altering the effect model.

### Grading of evidence

The quality of evidence for primary and secondary outcomes, as well as adverse reactions, was graded using the GRADEpro Guideline Development Tool software.

## Results

**Fig 1:**
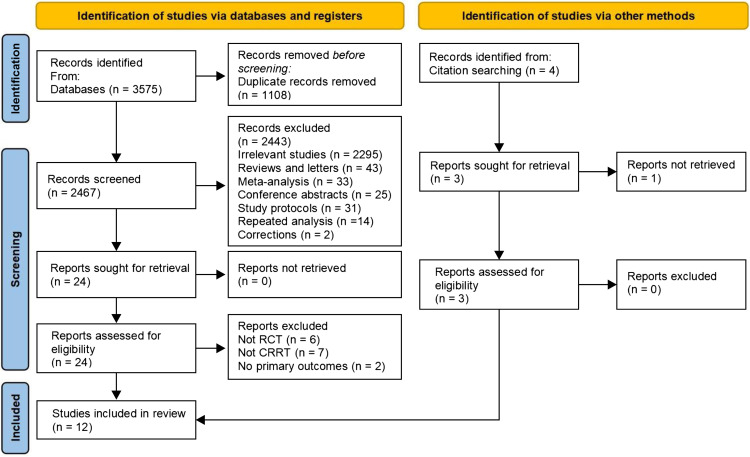
PRISMA 2020 flow diagram. RCT randomized controlled trial, CRRT continuous renal replacement therapy.

A total of 3,575 records were obtained from the three databases by the search strategy; of these, 1108 were duplicates; nine studies were deemed eligible for inclusion after the full text and abstracts were screened; the remaining three were found through citation searching; eventually, 12 studies were included in the meta-analysis [9–20] (Fig 1). The twelve studies included 1244 patients, with 617 in the early CRRT group and 627 in the delayed group. The causes of AKI included sepsis (n = 5), post-cardiac surgery (n = 2), and multiple factors (n = 5). Study characteristics are presented in Table 1. The information for included and excluding studies after removing duplicate records are detailed in S2 Table. The quality assessment rated the studies as having a moderate or low risk of bias (S1 Fig and S3 Table). More detailed research data are available in S4 Table.

**Table 1:** Characteristics of the included studies.

Study	Publication date	Country	Population included	Mean age (patients)	Scores before randomization	Sex ratio (men, women)	CRRT Mode	Outcome
**Combes et al. [** **10** **]**	2015	France	Cardiac surgery	59.50 (224)	SOFA: 11.80	79%, 21%	HVHF, CVVHDF	30-day mortality
**Srisawat et al. [** **11** **]**	2018	Thailand	Mixed	66.80 (40)	SOFA: 9.30	55%, 45%	CVVH	28-day mortality
**Zarbock et al. [** **12** **]**	2016	Germany	Surgical	67.00 (231)	SOFA: 15.80	63%, 37%	CVVHDF	90-day mortality
**Lumlertgul et al. [** **13** **]**	2018	Thailand	Mixed	67.10 (118)	SOFA: 12	49%, 51%	CVVH	28-day mortality
**Geri et al. [** **14** **]**	2019	France	Medical	67.3 (35)	SOFA: 8.70	71%, 29%	HCOCVVHD CVVH	28-day mortality
**Payen et al. [** **15** **]**	2009	France	Sepsis	58.1 (76)	SOFA: 11.00	71%, 29%	CVVH	14-day mortality
**Sugahara et al. [** **16** **]**	2004	Japan	Cardiac surgery	64.50 (28)	APACHE II: 18.50	64%, 36%	CVVHD	14-day mortality
**Xia et al. [** **17** **]**	2019	China	Sepsis	66.40 (60)	SOFA: 9.60	55%, 45%	CVVH	28-day mortality
**Yang et al. [** **18** **]**	2019	China	Sepsis	58.90 (142)	NA	61%, 39%	CVVH	28-day mortality
**Yin et al. [** **19** **]**	2018	China	Sepsis	60.80 (63)	APACHE II: 26.60	67%, 33%	CVVH	28-day mortality
**Bouman et al. [** **9** **]**	2002	Netherland	Mixed	68.50 (71)	SOFA: 10.40	59%, 41%	LVHF	28-day mortality
**An et al. [** **20** **]**	2021	China	Sepsis	60.30 (156)	APACHE II: 22.20	57%, 43%	CVVH	60-day mortality

SOFA, sepsis-related Organ Failure; APACHE II, Acute Physiology and Chronic Health Evaluation II; CRRT, continuous renal replacement therapy; RRT, renal replacement therapy; CVVH, continuous venovenous hemofiltration; CVVHD, continuous venovenous hemodialysis; CVVHDF, continuous venovenous hemodiafiltration; LVHF, low-volume hemofiltration; HVHF, high-volume hemofiltration; HCO-CVVHD, high cut-off continuous venovenous hemodialysis; NA, not available.

### Main outcome

**Fig 2:**
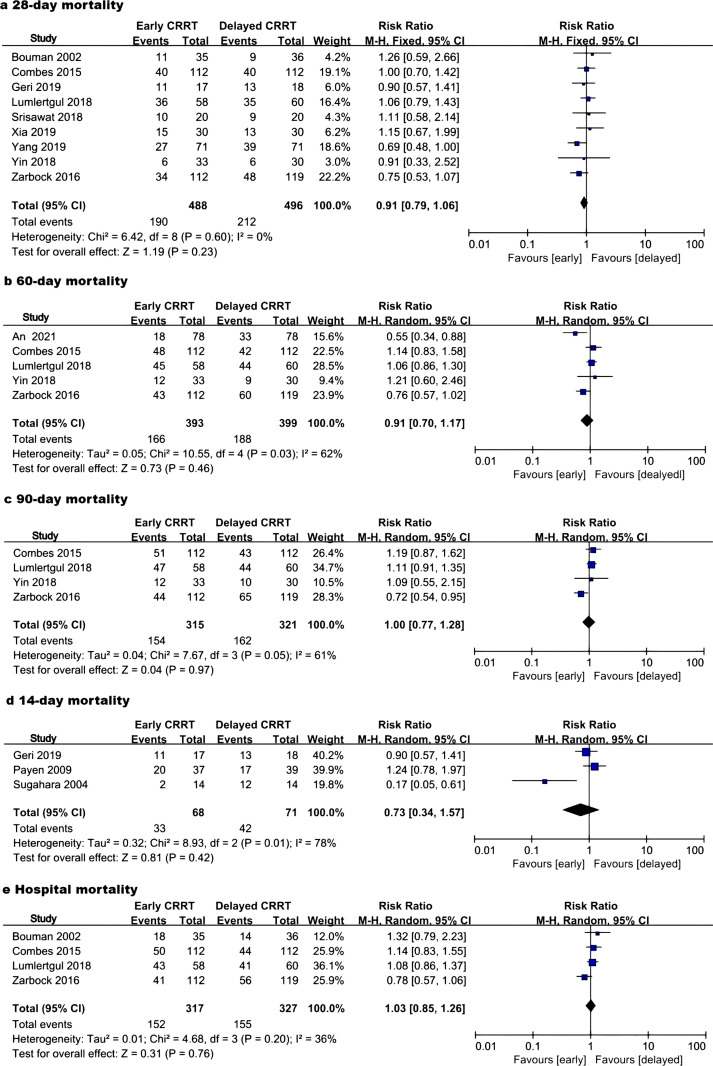
Forest plot of mortality. CRRT, continuous renal replacement therapy; M-H, Mantel–Haenszel; CI confidence interval.

The main outcome, 28-day mortality, was reported in nine studies [9–14,17–19], including 984 patients. The pooled mortality rate was 38.93% (190/488) in the early CRRT group and 42.74% (212/496) in the delayed CRRT group. Pooled analysis indicated a non-significantly lower 28-day mortality incidence in the early CRRT group (Fig 2a), with low heterogeneity between studies (p = 0.60; I^2^ = 0%). A fixed-effects model was applied. The funnel plot did not indicate significant publication bias (S2 Fig).

### Secondary outcome

#### Mortality.

Five studies assessed 60-day mortality [10,12,13,19,20] [Fig 2b] (792 patients), four examined 90-day mortality [10,12,13,19] [Fig 2c] (636 patients), three evaluated 14-day mortality [14–16] [Fig 2d] (139 patients), and four investigated in-hospital mortality [9,10,12,13] [Fig 2e] (644 patients). The analysis indicated no significant between-group differences in 60-day mortality [RR 0.91 (0.70, 1.17); P = 0.46; I^2^ = 62%; P = 0.03], 90-day mortality [RR 1.00 (0.77, 1.28); P = 0.97; I^2^ = 61%; P = 0.05], 14-day mortality [RR 0.73 (0.34, 1.57); P = 0.42; I^2^ = 78%; P = 0.01], or hospital-based mortality [RR 1.03 (0.85, 1.26); P = 0.76; I^2^ = 36%; P = 0.20]. I^2^ ≥ 30% or p ≤ 0.10, all using a random-effects model.

#### Number of patients receiving RRT.

Five studies reported on the number of patients receiving RRT [9–13], with 99.41% (335/337) of the patients in the early CRRT group and 73.49% (255/347) of them in the delayed CRRT group undergoing RRT during treatment. The early CRRT group exhibited a significantly higher proportion of patients receiving RRT [RR 1.41 (1.09, 1.83); P = 0.009; I^2^ = 94%; p ≤ 0.00001] (S3 Fig a).

#### Number of patients dependent on RRT (at discharge and day 28).

Six studies [10–13,16,17] (407 patients) documented RRT dependency on day 28, and four studies [9,10,12,13] (332 patients) recorded RRT dependency at discharge. Comparative analysis revealed no significant differences in RRT dependency on day 28 [RR 0.62 (0.33, 1.18); P = 0.15; I^2^ = 45%; P = 0.11] (S3 Fig b). However, early initiation of CRRT was associated with a significant reduction in RRT dependency at discharge [RR 0.57 (0.32, 0.99); P = 0.05; I^2^ = 0%; P = 0.47] (S3 Fig c).

#### Length of stays in the ICU and hospital.

Five studies [9,12,13,18,19] (625 patients) and four studies [9,12,13,19] (483 patients) reported the length of stay in the ICU and hospital, respectively. Meta-analysis indicated a significantly shorter ICU stay for the early CRRT group [MD -3.24 (-5.14, -1.35); P = 0.0008; I^2^ = 36%; P = 0.18] (S3 Fig d). However, no apparent difference in length of hospital stay was observed between groups [MD -7.00 (-14.60, 0.60); P = 0.07; I^2^ = 38%; P = 0.18] (S3 Fig e).

#### Duration of mechanical ventilation.

Four studies encompassing 589 patients reported the duration of mechanical ventilation [9,10,12,19]. The analysis revealed no significant between-group difference in the duration of mechanical ventilation, including survivors and non-survivors [MD -1.67 (-4.24, 0.91); P =  0.20; I^2^ =  75%; P = 0.008] (S3 Fig f).

#### Adverse events during treatment.

**Fig 3:**
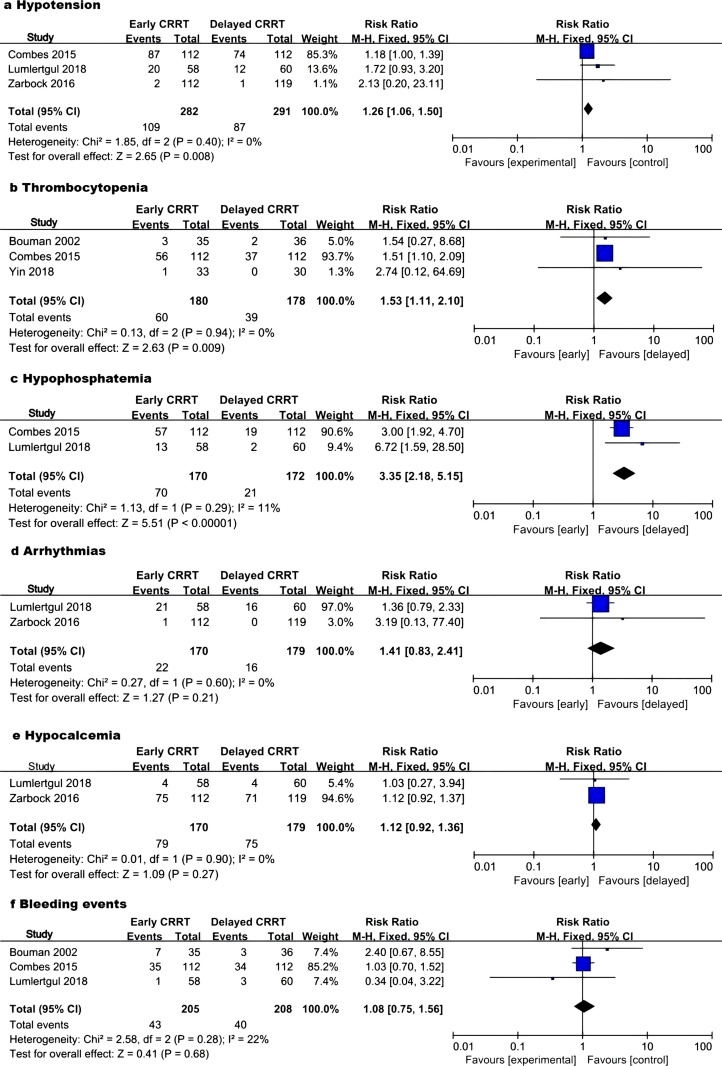
Forest plot of adverse events. CRRT, continuous renal replacement therapy; M-H, Mantel–Haenszel; CI, confidence interval.

Three studies involving 573 patients reported hypotension [10,12,13]. The early CRRT group experienced a higher incidence of hypotensive events [RR 1.26 (1.06, 1.50); P = 0.008; I^2^ =  0%; P = 0.40] (Fig 3a). Similarly, three studies with 358 patients reported an increased incidence of thrombocytopenia in the early CRRT group [RR 1.53 (1.11, 2.10); P =  0.009; I^2^ =  0%; P = 0.94] (Fig 3b). Hypophosphatemia was also more prevalent in the early CRRT group, as indicated by two studies with 342 patients [RR 3.35 (2.18, 5.15); P < 0.00001; I^2^ =  11%; P = 0.29] (Fig 3c). For arrhythmia, hypocalcemia, and bleeding events, the RRs respectively were 1.41 [(0.83, 2.41); P = 0.21; I^2^ =  0%; P = 0.60] (Fig 3d), 1.12 [(0.92, 1.36); P = 0.27; I^2^ =  0%; P = 0.90] (Fig 3e), and 1.08 [(0.75, 1.56); P = 0.68; I^2^ =  22%; P = 0.28] (Fig 3f).

### Grading of evidence

The results of evidence quality evaluation are presented in S5 Table. The quality of evidence was deemed “high” for the following outcomes: 28-day mortality, hospital mortality, and adverse events (including hypotension, thrombocytopenia, hypophosphatemia, hypocalcemia, and bleeding events).

### Subgroup and sensitivity analyses

Subgroup analysis demonstrated that variables such as average age of participants (≥64 years or < 64 years), AKI etiology (sepsis or other factors), and condition severity (SOFA scores ≥ 12 or APACHE II scores ≥ 25) did not significantly affect 28-day mortality (Table 2, S4 Fig, S5 Fig, and S6 Fig). Sensitivity analysis, involving the exclusion of individual studies and adjustments to the effect model, confirmed the robustness of the primary result (Table 3 and S7 Fig).

**Table 2:** Subgroup analysis of 28-day mortality.

Group	No. of trials	No. of patients			Risk ratio (95% CI)			P-value	Heterogeneity	
									I^2^ index (%)	P-value
**Average age**										
** <64 years**	3	429				0.85 (0.67, 1.09)		0.20	3	0.36
** >64 years**	6	555				0.96 (0.80, 1.15)		0.64	0	0.62
**AKI etiology**										
** Sepsis**	3		265			0.82 (0.61, 1.09)		0.18	16	0.30
** Non-sepsis/mixed factors**	6		719			0.95 (0.80, 1.13)		0.57	0	0.68
**SOFA/APACHE II scores**										
** Low score**	5	430		1.05 (0.83, 1.32)			0.69		0	0.92
** High score**	3	412		0.89 (0.71, 1.11)			0.30		13	0.32

SOFA, sepsis-related organ failure; APACHE II, Acute Physiology and Chronic Health Evaluation II; AKI, acute kidney injury.

**Table 3:** Sensitivity analysis of 28-day mortality.

Excluded research	No. of patients	Risk ratio (95% CI)	P-value	Heterogeneity	
**I**^**2**^ **index (%)**	**P-value**
**Bouman 2002**	913	0.90 (0.77, 1.04)	0.17	0	0.57
**Combes 2015**	760	0.89 (0.76, 1.05)	0.18	0	0.52
**Geri 2019**	946	0.92 (0.79, 1.07)	0.26	0	0.49
**Lumlertgul 2018**	866	0.89 (0.75, 1.05)	0.15	0	0.63
**Srisawat 2018**	944	0.91 (0.78, 1.05)	0.20	0	0.53
**Xia 2019**	924	0.90 (0.77, 1.05)	0.17	0	0.57
**Yang 2019**	842	0.97 (0.82, 1.13)	0.67	0	0.83
**Yin 2018**	921	0.91 (0.79, 1.06)	0.24	0	0.49
**Zarbock 2016**	753	0.96 (0.82, 1.13)	0.63	0	0.68

CI, confidence interval.

## Discussion

This meta-analysis, encompassing 12 RCTs, assessed the effects of starting CRRT early versus late on AKI patient outcomes. The analysis revealed no notable variations in mortality at days 14, 28, 60, and 90 based on CRRT initiation timing, indicating no apparent survival benefit for early CRRT initiation in patients with AKI. Sensitivity analyses, including model variations and exclusion of individual RCTs, confirmed the robustness of these primary results. These results contribute to our understanding whether the CRRT modality influences outcomes in this contentious area.

Previously, the connection between the timing and results of AKI RRT has primarily been examined through retrospective studies and meta-analyses. However, recent advancements in large-scale, high-quality RCTs [21] and meta-analyses [6–8] have led to divergent conclusions, with a growing consensus that early RRT initiation does not confer a survival advantage for patients with AKI. Some meta-analyses [6–8] did not restrict the early RRT intervention mode. Li [22] limited RRT intervention to patients with sepsis, whereas Xia [23] included fewer RCTs and lacked subsequent studies. Our meta-analysis, which included all AKI patients with early CRRT initiation as the regimen, and incorporated the latest RCTs, provides a comprehensive and updated perspective.

For the interventions in the included studies, the selected CRRT modes varied, and patient criteria for transitioning to intermittent dialysis also differed. Disparities existed in the causes of AKI, CRRT initiation criteria, and inclusion criteria across the RCTs; partial pooled analyses revealed significant heterogeneity. Consequently, the results should be approached with caution.

In this study, the early CRRT group had more patients receiving RRT, compared to the delayed CRRT group, consistent with other studies [6–8]. In the delayed CRRT group, most patients, except for those who died or had other complications, did not require RRT because of renal function recovery. This indicates that implementing delayed strategies could reduce medical resource utilization and mitigate CRRT-related adverse effects. However, the AKIKI2 trial [24] randomly assigned patients with AKI to delayed and more-delayed CRRT strategy groups, with the more delayed strategy group demonstrating no additional benefits or potential harm. Thus, an appropriately delayed initiation strategy groups, tailored to the patient’s specific conditions, may be more advantageous than delaying until mandatory indications such as evident hyperkalemia, acidosis, or pulmonary edema emerge.

Additionally, this meta-analysis demonstrated that the early start of CRRT significantly reduced the number of patients dependent on RRT at discharge, and no discernible difference in hospital stay was observed, except for reduced ICU stay duration. Pooled analysis of adverse events revealed a greater risk of hypotension, thrombocytopenia, and hypophosphatemia in the early CRRT group, while no significant differences were noted in cardiac arrhythmias, hypocalcemia, and bleeding events.

Early initiation of CRRT facilitates the removal of toxins and inflammatory mediators in sepsis, potentially preventing its progression. However, subgroup analysis indicated that early CRRT initiation did not confer significant benefits to patients with AKI in the sepsis subgroup. This finding aligns with that of the IDEAL-ICU trial [25], where early RRT initiation in septic patients with AKI did not result in a lower 90-day mortality rate. Although theoretically, reducing unbound cytokines might mitigate organ damage in patients with sepsis and lower mortality, cytokine aggregation in the tissue gap may influence outcomes [26]. Therefore, an optimized strategy for clearing cytokines in tissues is necessary to enhance outcomes in patients with sepsis.

This study had several strengths. First, it incorporated a greater number of RCTs using CRRT as the treatment regimen for early intervention, including the latest research from 2019 onwards [14,17,18,20]. Second, it provided a comprehensive evaluation of the impact of CRRT initiation timing on treatment results, such as mortality rates at various intervals and the incidence of adverse events. However, this study also had some limitations. Primarily, there was significant variability in CRRT initiation timing among the included studies. Additionally, the lack of subgroup analyses based on different CRRT initiation timings precludes a more nuanced assessment of the effect of CRRT on survival outcomes. Finally, some studies involved patients initially receiving CRRT but transitioning to intermittent RRT once hemodynamic stability and other requirements had been achieved.

## Conclusion

In conclusion, our findings indicate that early CRRT may increase the risk adverse outcomes without improving prognosis. Therefore, patients with AKI and those treated in settings with limited medical resources may benefit more from appropriate delays in starting CRRT.

## Supporting information

S1 Fig Risk of bias summary.
(TIF)

S2 Fig Funnel plot to evaluate publication bias.
(TIF)

S3 Fig Forest plot of the secondary outcomes. (TIF)

S4 Fig Subgroup analyses by average age of participants. (TIF)

S5 Fig Subgroup analyses by AKI etiology. (TIF)

S6 Fig Subgroup analyses by the scores. (TIF)

S7 Fig Forest plot by random effects. (TIF)

S1 Checklist PRISMA 2020 checklist.
(DOCX)

S1 Table Search strategy.
(DOCX)

S2 Table Studies included and excluded.
(DOCX)

S3 Table Risk of bias of included studies.
(DOCX)

S4 Table Raw data used in the current meta-analysis.
(DOCX)

S5 Table Assessment of certainty of evidence.
(DOCX)

## References

[1] Hoste EAJ, Bagshaw SM, Bellomo R, Cely CM, Colman R, Cruz DN, et al. Epidemiology of acute kidney injury in critically ill patients: the multinational AKI-EPI study. Intensive Care Med. 2015;41(8):1411–23. doi: 10.1007/s00134-015-3934-7 26162677

[2] Silver SA, Harel Z, McArthur E, Nash DM, Acedillo R, Kitchlu A, et al. Causes of Death after a Hospitalization with AKI. J Am Soc Nephrol. 2018;29(3):1001–10. doi: 10.1681/ASN.2017080882 29242248 PMC5827605

[3] Hoste EAJ, Kellum JA, Selby NM, Zarbock A, Palevsky PM, Bagshaw SM, et al. Global epidemiology and outcomes of acute kidney injury. Nat Rev Nephrol. 2018;14(10):607–25. doi: 10.1038/s41581-018-0052-0 30135570

[4] Karvellas CJ, Farhat MR, Sajjad I, Mogensen SS, Leung AA, Wald R, et al. A comparison of early versus late initiation of renal replacement therapy in critically ill patients with acute kidney injury: a systematic review and meta-analysis. Crit Care. 2011;15(1):R72. doi: 10.1186/cc10061 21352532 PMC3222005

[5] Fayad AII, Buamscha DG, Ciapponi A. Timing of renal replacement therapy initiation for acute kidney injury. Cochrane Database Syst Rev. 2018;12(12):CD010612. doi: 10.1002/14651858.CD010612.pub2 30560582 PMC6517263

[6] Gaudry S, Hajage D, Benichou N, Chaïbi K, Barbar S, Zarbock A, et al. Delayed versus early initiation of renal replacement therapy for severe acute kidney injury: a systematic review and individual patient data meta-analysis of randomised clinical trials. Lancet. 2020;395(10235):1506–15. doi: 10.1016/S0140-6736(20)30531-6 32334654

[7] Li X, Liu C, Mao Z, Li Q, Zhou F. Timing of renal replacement therapy initiation for acute kidney injury in critically ill patients: a systematic review of randomized clinical trials with meta-analysis and trial sequential analysis. Crit Care. 2021;25(1):15. doi: 10.1186/s13054-020-03451-y 33407756 PMC7789484

[8] Fayad AI, Buamscha DG, Ciapponi A. Timing of kidney replacement therapy initiation for acute kidney injury. Cochrane Database Syst Rev. 2022;11(11):CD010612. doi: 10.1002/14651858.CD010612.pub3 36416787 PMC9683115

[9] Bouman CSC, Oudemans-Van Straaten HM, Tijssen JGP, Zandstra DF, Kesecioglu J. Effects of early high-volume continuous venovenous hemofiltration on survival and recovery of renal function in intensive care patients with acute renal failure: a prospective, randomized trial. Crit Care Med. 2002;30(10):2205–11. doi: 10.1097/00003246-200210000-00005 12394945

[10] Combes A, Bréchot N, Amour J, Cozic N, Lebreton G, Guidon C, et al. Early High-Volume Hemofiltration versus Standard Care for Post-Cardiac Surgery Shock. The HEROICS Study. Am J Respir Crit Care Med. 2015;192(10):1179–90. doi: 10.1164/rccm.201503-0516OC 26167637

[11] Srisawat N, Laoveeravat P, Limphunudom P, Lumlertgul N, Peerapornratana S, Tiranathanagul K, et al. The effect of early renal replacement therapy guided by plasma neutrophil gelatinase associated lipocalin on outcome of acute kidney injury: A feasibility study. J Crit Care. 2018;43:36–41. doi: 10.1016/j.jcrc.2017.08.029 28843662

[12] Zarbock A, Kellum JA, Schmidt C, Van Aken H, Wempe C, Pavenstädt H, et al. Effect of Early vs Delayed Initiation of Renal Replacement Therapy on Mortality in Critically Ill Patients With Acute Kidney Injury: The ELAIN Randomized Clinical Trial. JAMA. 2016;315(20):2190–9. doi: 10.1001/jama.2016.5828 27209269

[13] Lumlertgul N, Peerapornratana S, Trakarnvanich T, Pongsittisak W, Surasit K, Chuasuwan A, et al. Early versus standard initiation of renal replacement therapy in furosemide stress test non-responsive acute kidney injury patients (the FST trial). Crit Care. 2018;22(1):101. doi: 10.1186/s13054-018-2021-1 29673370 PMC5909278

[14] Geri G, Grimaldi D, Seguin T, Lamhaut L, Marin N, Chiche J-D, et al. Hemodynamic efficiency of hemodialysis treatment with high cut-off membrane during the early period of post-resuscitation shock: The HYPERDIA trial. Resuscitation. 2019;140:170–7. doi: 10.1016/j.resuscitation.2019.03.045 30974188

[15] Payen D, Mateo J, Cavaillon JM, Fraisse F, Floriot C, Vicaut E, et al. Impact of continuous venovenous hemofiltration on organ failure during the early phase of severe sepsis: a randomized controlled trial. Crit Care Med. 2009;37(3):803–10. doi: 10.1097/CCM.0b013e3181962316 19237881

[16] Sugahara S, Suzuki H. Early start on continuous hemodialysis therapy improves survival rate in patients with acute renal failure following coronary bypass surgery. Hemodial Int. 2004;8(4):320–5. doi: 10.1111/j.1492-7535.2004.80404.x 19379436

[17] Xia Y, Shi H, Wu W, Wang X. Effect of urinary NGAL on the timing of renal replacement therapy in patients with acute renal injury associated with sepsis. Med J Chin PLA. 2019;44(07):605–10.

[18] Yang T, Zhou R, Yao H, Peng L. Effect of timing of continuous renal replacement therapy on prognosis of sepsis patients with acute renal injury. Mod Instrum Med Treat. 2019;25(05):11–5.

[19] Yin J, Zheng R, Lin H, Shao J. Influence of initiation timing of continuous renal replacement therapy on prognosis of patients with septic shock and acute kidney injury. J Clin Med Pract. 2018;22(07):63–6.

[20] An N, Chen R, Bai Y, Xu M. Efficacy and prognosis of continuous renal replacement therapy at different times in the treatment of patients with sepsis-induced acute kidney injury. Am J Transl Res. 2021;13(6):7124–31. 34306472 PMC8290701

[21] STARRT-AKI Investigators, Canadian Critical Care Trials Group, Australian and New Zealand Intensive Care Society Clinical Trials Group, United Kingdom Critical Care Research Group, Canadian Nephrology Trials Network, Irish Critical Care Trials Group, et al. Timing of Initiation of Renal-Replacement Therapy in Acute Kidney Injury. N Engl J Med. 2020;383(3):240–51. doi: 10.1056/NEJMoa2000741 32668114

[22] Li Y, Li H, Zhang D. Timing of continuous renal replacement therapy in patients with septic AKI: A systematic review and meta-analysis. Medicine (Baltimore). 2019;98(33):e16800. doi: 10.1097/MD.0000000000016800 31415389 PMC6831327

[23] Xia Z-J, He L-Y, Pan S-Y, Cheng R-J, Zhang Q-P, Liu Y. Disease Severity Determines Timing of Initiating Continuous Renal Replacement Therapies: A Systematic Review and Meta-Analysis. Front Med (Lausanne). 2021;8:580144. doi: 10.3389/fmed.2021.580144 34869398 PMC8636750

[24] Gaudry S, Hajage D, Martin-Lefevre L, Lebbah S, Louis G, Moschietto S, et al. Comparison of two delayed strategies for renal replacement therapy initiation for severe acute kidney injury (AKIKI 2): a multicentre, open-label, randomised, controlled trial. Lancet. 2021;397(10281):1293–300. doi: 10.1016/S0140-6736(21)00350-0 33812488

[25] Barbar SD, Clere-Jehl R, Bourredjem A, Hernu R, Montini F, Bruyère R, et al. Timing of Renal-Replacement Therapy in Patients with Acute Kidney Injury and Sepsis. N Engl J Med. 2018;379(15):1431–42. doi: 10.1056/NEJMoa1803213 30304656

[26] Honore PM, Jacobs R, Joannes-Boyau O, De Regt J, Boer W, De Waele E, et al. Septic AKI in ICU patients. diagnosis, pathophysiology, and treatment type, dosing, and timing: a comprehensive review of recent and future developments. Ann Intensive Care. 2011;1(1):32. doi: 10.1186/2110-5820-1-32 21906387 PMC3224527

