# Supplementary figures and images for: Selection of timing of continuous renal replacement therapy in patients with acute kidney injury: A meta-analysis of randomized controlled trials
Source: PLoS One. 2025 Mar 25;20(3):e0320351. doi: 10.1371/journal.pone.0320351 (PMC11936205; doi:10.1371/journal.pone.0320351)

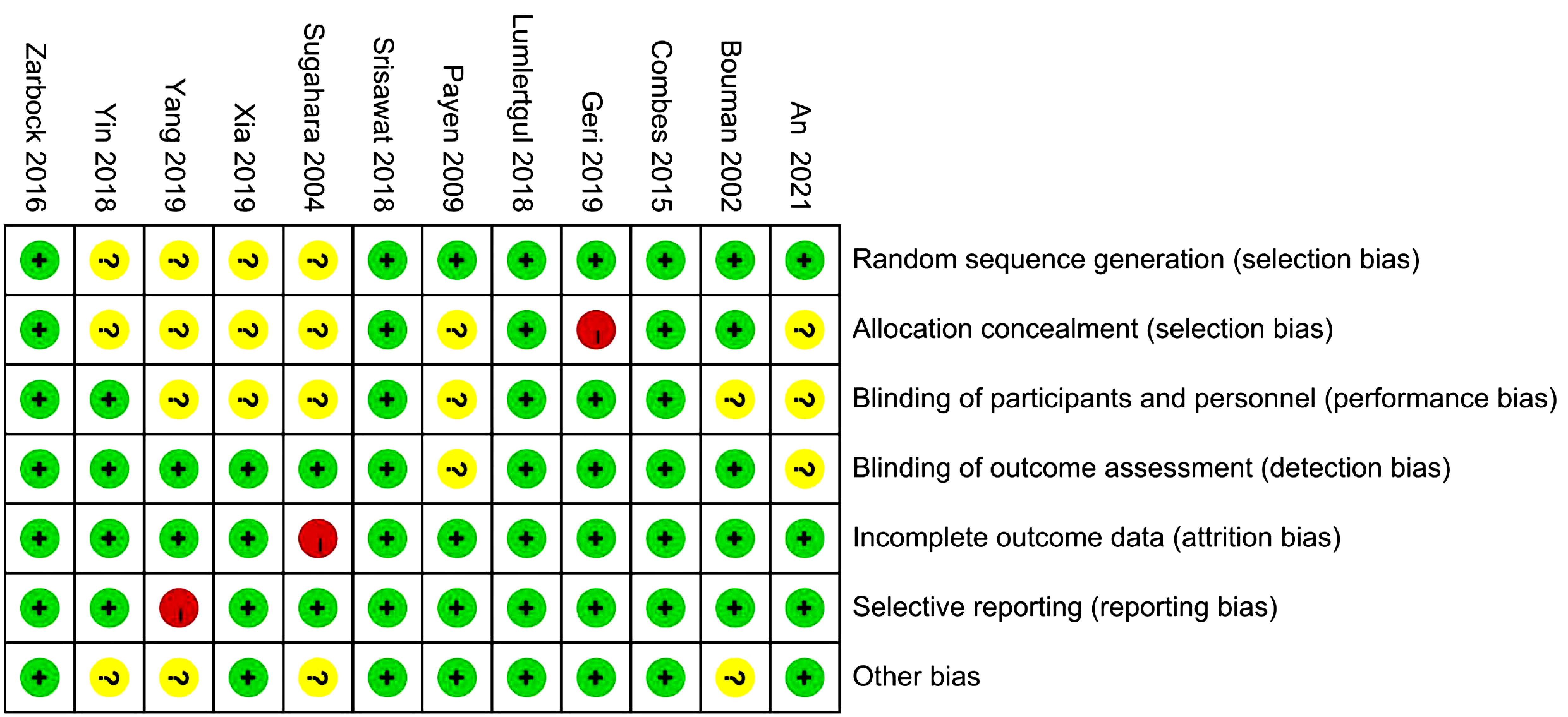

Supplement: S1 Fig — (TIF) [file pone.0320351.s001.tif]

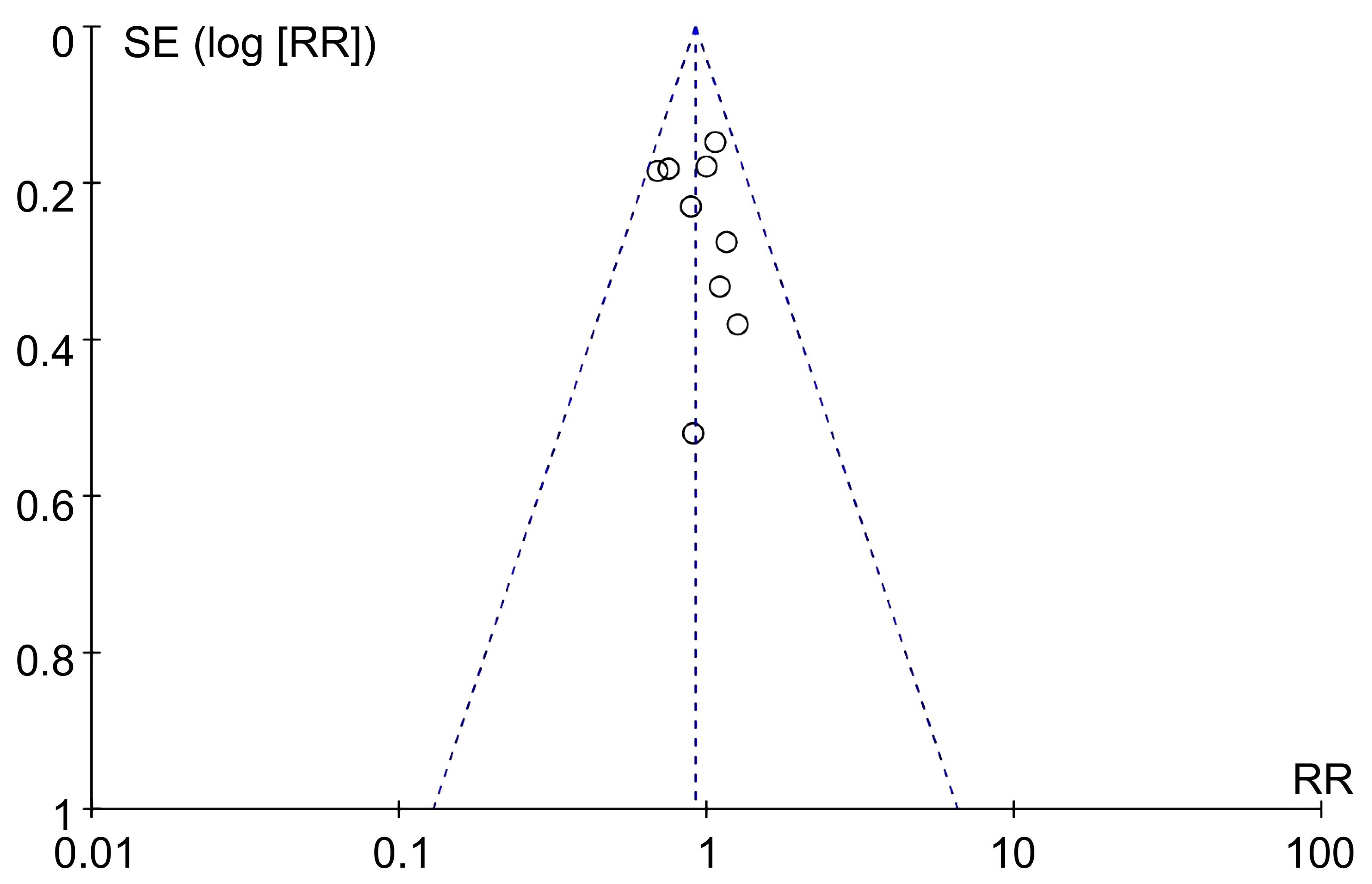

Supplement: S2 Fig — (TIF) [file pone.0320351.s002.tif]

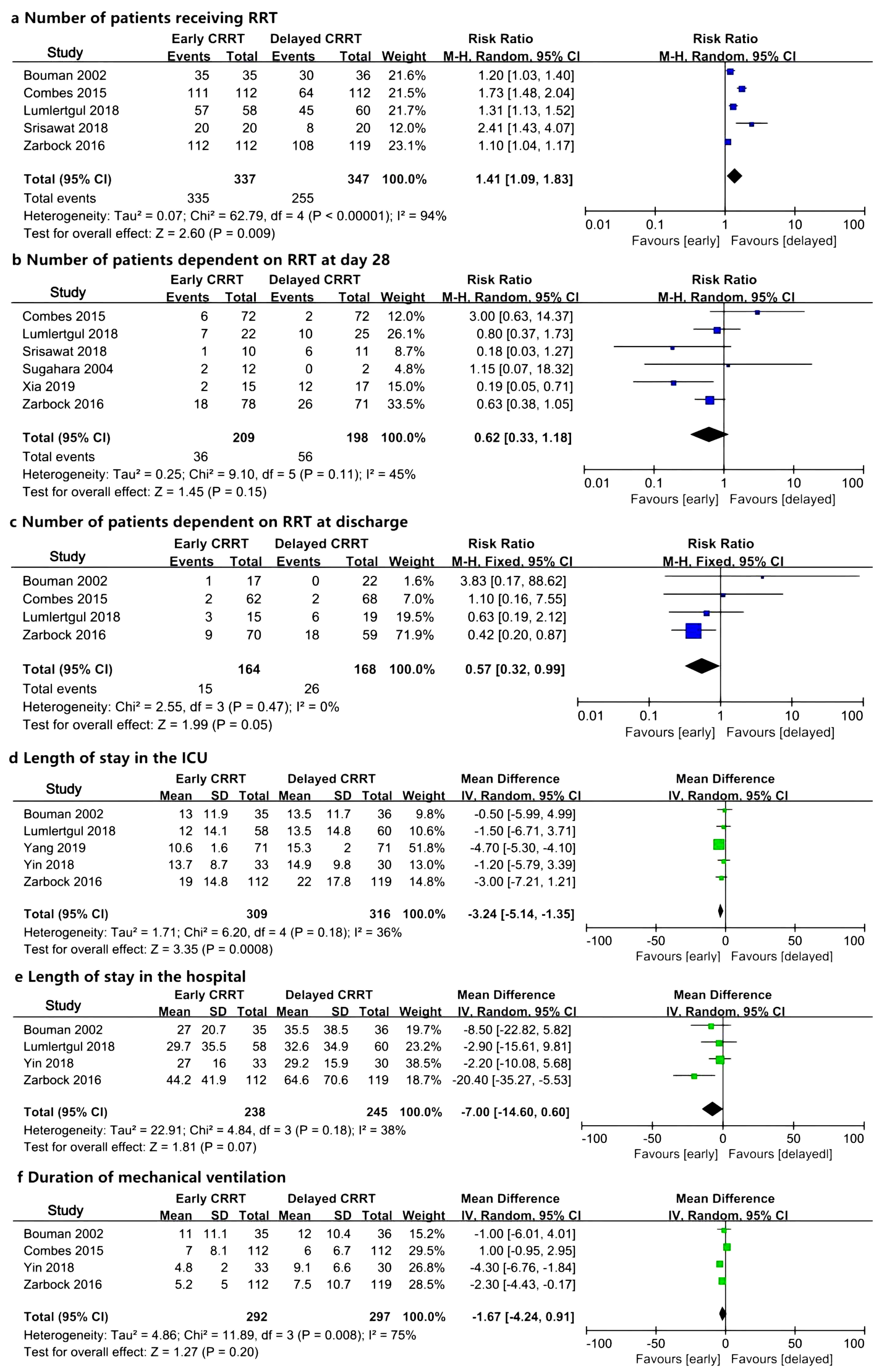

Supplement: S3 Fig — (TIF) [file pone.0320351.s003.tif]

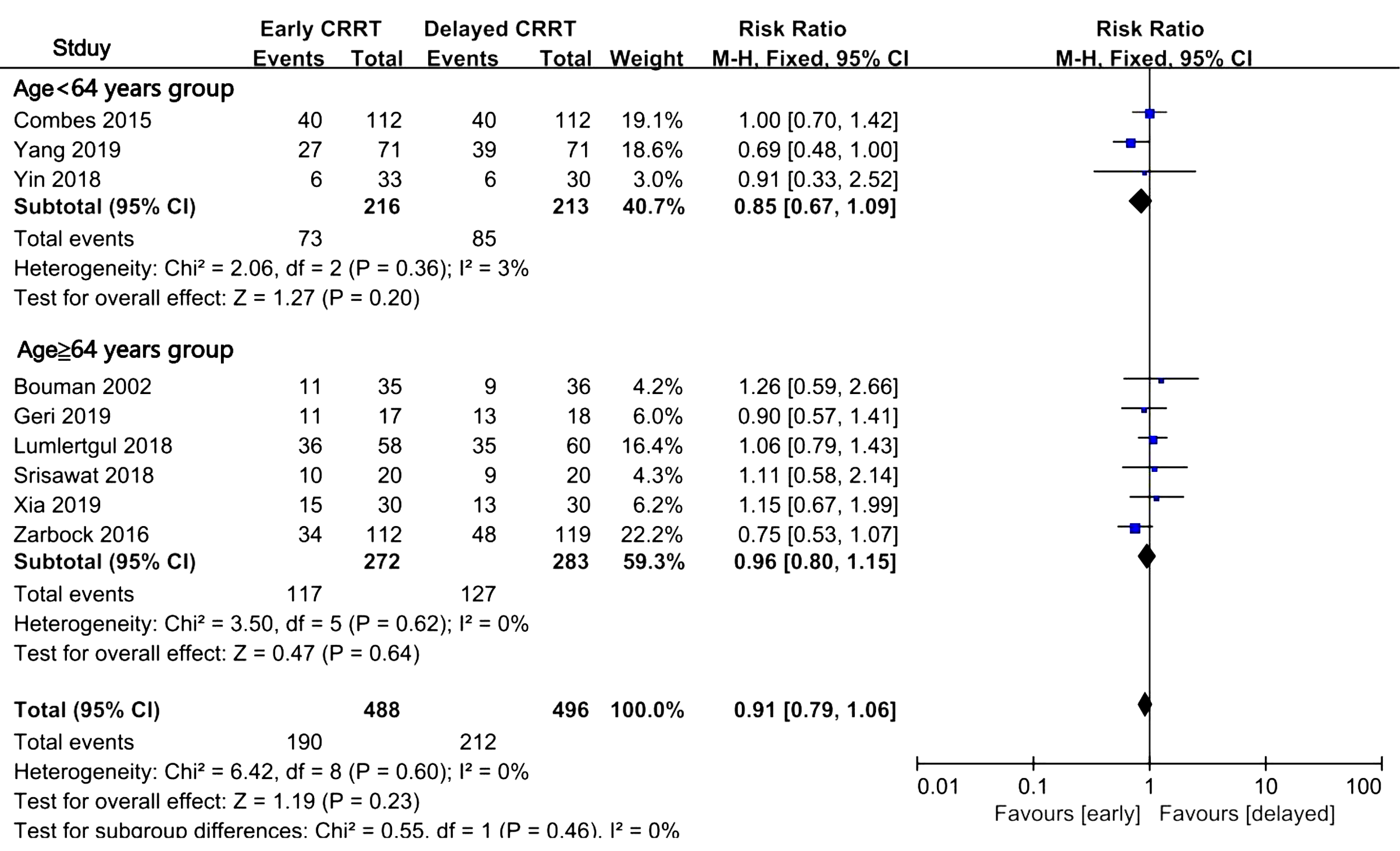

Supplement: S4 Fig — (TIF) [file pone.0320351.s004.tif]

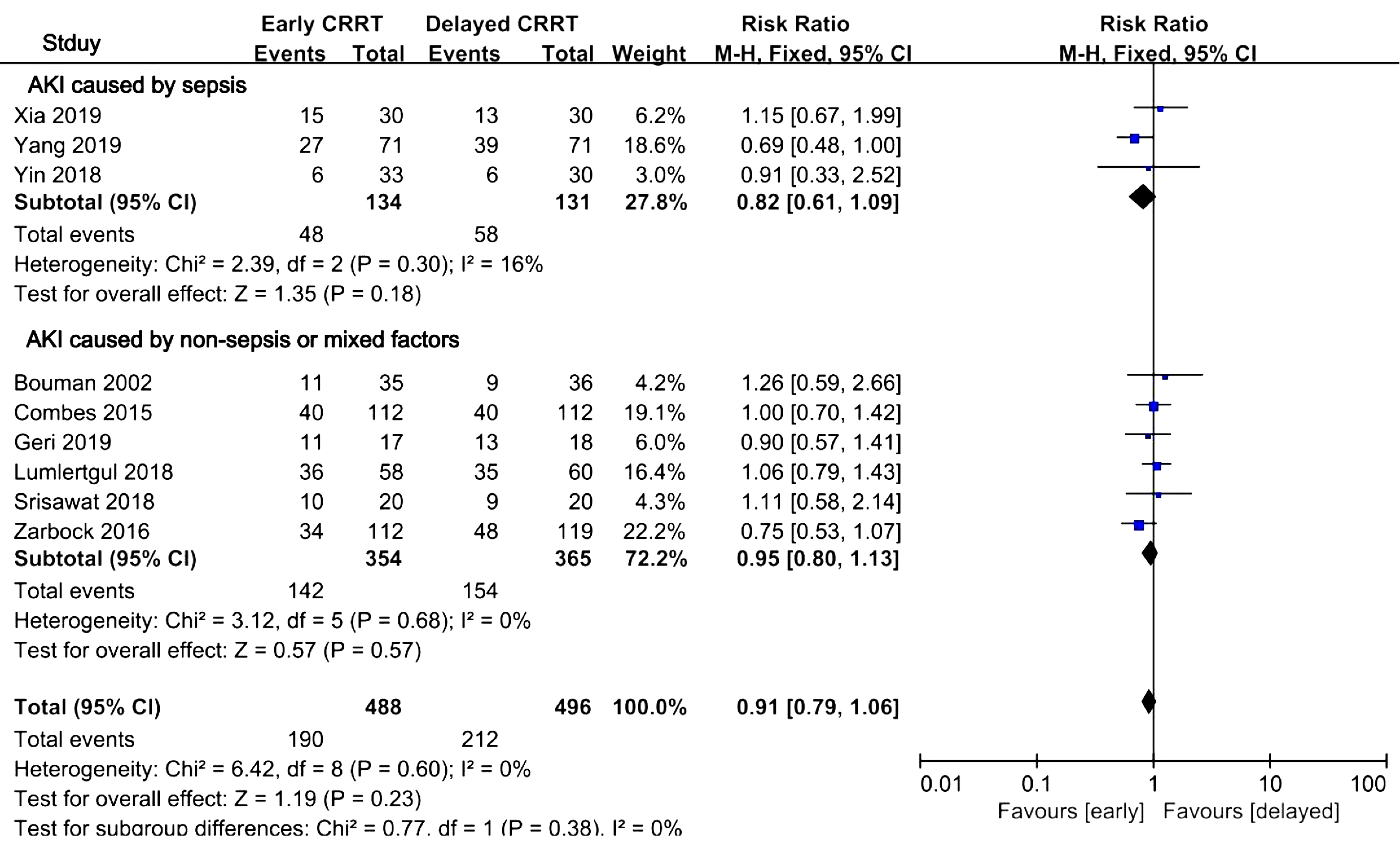

Supplement: S5 Fig — (TIF) [file pone.0320351.s005.tif]

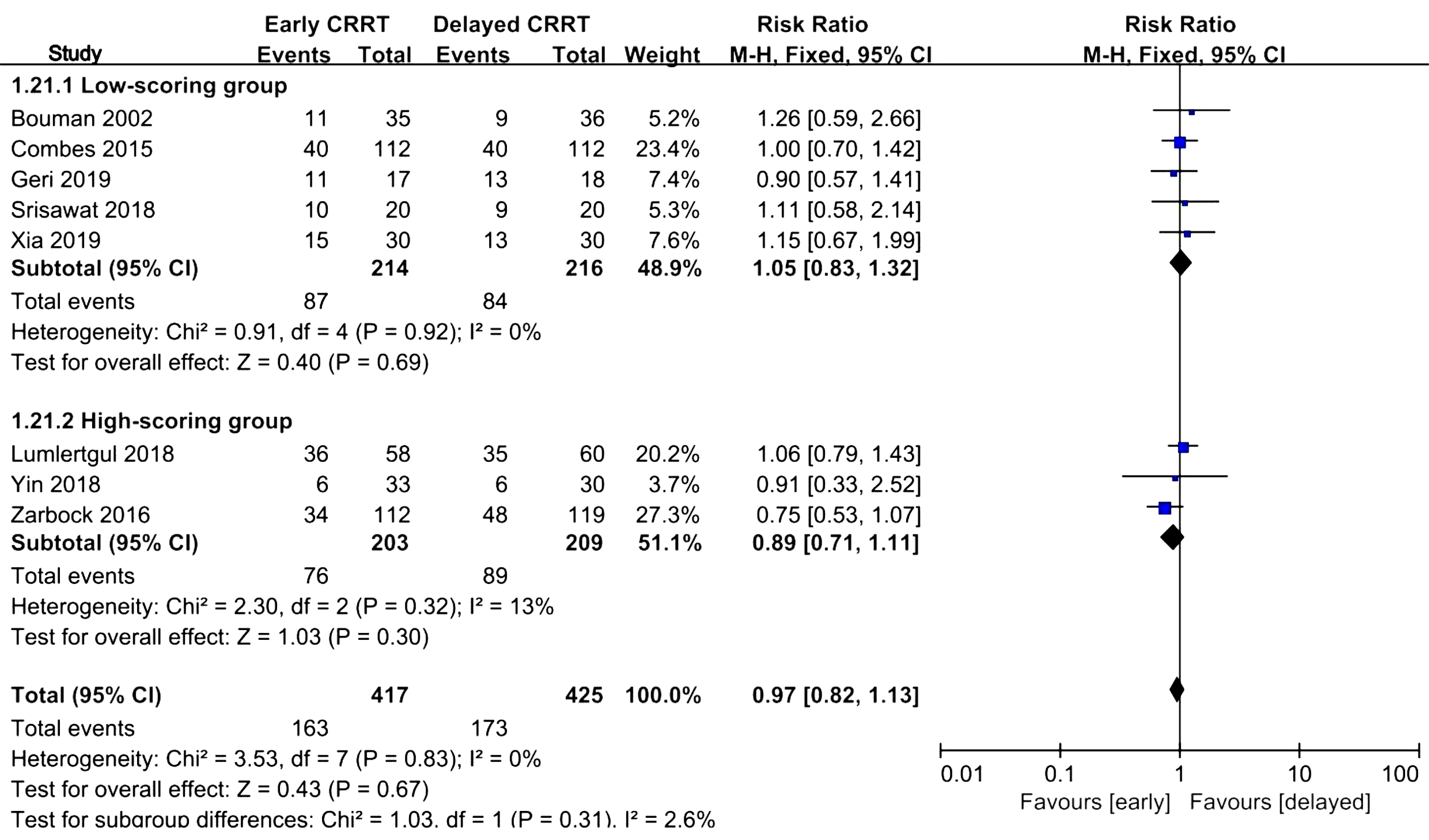

Supplement: S6 Fig — (TIF) [file pone.0320351.s006.tif]

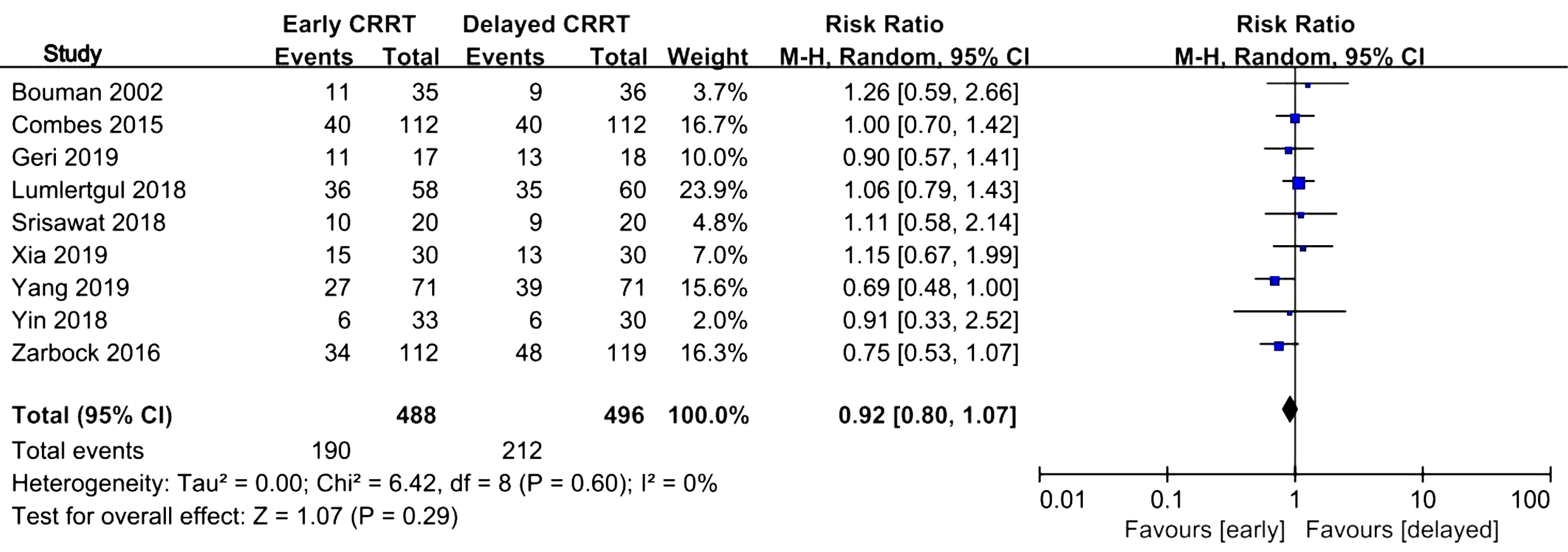

Supplement: S7 Fig — (TIF) [file pone.0320351.s007.tif]
