# Supplementary material for: Selection of timing of continuous renal replacement therapy in patients with acute kidney injury: A meta-analysis of randomized controlled trials
Source: PLoS One. 2025 Mar 25;20(3):e0320351. doi: 10.1371/journal.pone.0320351 (PMC11936205; doi:10.1371/journal.pone.0320351)
Supplement: S1 Table — (DOCX) [file pone.0320351.s009.docx]

**S1 Table. Search strategy**

**a:** Search strategy in PubMed

| **PubMed** | (((((((("Acute Kidney Injury"[Mesh]) OR (Acute Kidney Injury[Title/Abstract])) OR (acute renal injury[Title/Abstract])) OR (acute kidney failure[Title/Abstract])) OR (acute renal failure[Title/Abstract])) OR ("Kidney Tubular Necrosis, Acute"[Mesh])) AND (((((("Renal Replacement Therapy"[Mesh]) OR (Renal Replacement Therapy[Title/Abstract])) OR (hemofiltration[Title/Abstract])) OR (hemodialysis[Title/Abstract])) OR ("Renal Dialysis"[Mesh])) OR (dialysis[Title/Abstract]))) AND (((((((((early[Title/Abstract]) OR (timing[Title/Abstract])) OR ("Time-to-Treatment"[Mesh])) OR (time[Title/Abstract])) OR (accelerated[Title/Abstract])) OR (accelerating[Title/Abstract])) OR (earlier-start[Title/Abstract])) OR (late[Title/Abstract])) OR (delayed[Title/Abstract]))) AND ((((((randomized controlled trial[Publication Type]) OR (controlled clinical trial[Publication Type])) OR (randomized[Title/Abstract])) OR (randomly[Title/Abstract])) OR (random[Title/Abstract])) OR (placebo[Title/Abstract])) |
| --- | --- |

**b:** Search strategy in Cochrane Library

| **Cochrane** | #1 MeSH descriptor: [Acute Kidney Injury] explode all trees  #2 (acute kidney injury):ti,ab,kw (Word variations have been searched)  #3 (acute renal injury):ti,ab,kw (Word variations have been searched)  #4 (acute kidney failure):ti,ab,kw (Word variations have been searched)  #5 (acute renal failure):ti,ab,kw (Word variations have been searched)  #6 (acute kidney insufficiency):ti,ab,kw (Word variations have been searched)  #7 (acute renal insufficiency):ti,ab,kw (Word variations have been searched)  #8 (acute tubular necrosis):ti,ab,kw (Word variations have been searched)  #9 #1 or #2 or #3 or #4 or #5 or #6 or #7 or #8  #10 MeSH descriptor: [Renal Replacement Therapy] explode all trees  #11 MeSH descriptor: [Renal Dialysis] explode all trees  #12 (Renal Replacement Therapy):ti,ab,kw (Word variations have been searched)  #13 (Renal Dialysis):ti,ab,kw (Word variations have been searched)  #14 (hemofiltration):ti,ab,kw (Word variations have been searched)  #15 (hemodialysis):ti,ab,kw (Word variations have been searched)  #16 MeSH descriptor: [Dialysis] explode all trees  #17 #10 or #11 or #12 or #13 or #14 or #15 or #16  #18 #9 and #17  #19 (early):ti,ab,kw (Word variations have been searched)  #20 MeSH descriptor: [Time-to-Treatment] explode all trees  #21 (timing):ti,ab,kw (Word variations have been searched)  #22 (time):ti,ab,kw (Word variations have been searched)  #23 (accelerated):ti,ab,kw (Word variations have been searched)  #24 (accelerating):ti,ab,kw (Word variations have been searched)  #25 (earlier-start):ti,ab,kw (Word variations have been searched)  #26 (late):ti,ab,kw (Word variations have been searched)  #27 (delayed):ti,ab,kw (Word variations have been searched)  #28 #19 or #20 or #21 or #22 or #23 or #24 or #25 or #26 or #27  #29 #18 and #28 |
| --- | --- |

**c:** Search strategy in Embase

| **Embase** | #33. #7 AND #23 AND #31 AND #32  #32. #8 OR #9 OR #10 OR #11 OR #12 OR #13  #31. #24 OR #25 OR #26 OR #27 OR #28 OR #29 OR #30  #30. 'random':ti,ab  #29. 'double-blind':ti,ab  #28. 'randomly':ti,ab  #27. 'placebo':ti,ab  #26. 'randomized':ti,ab  #25. 'controlled clinical trial':ti,ab  #24. 'randomized controlled trial':ti,ab  #23. #14 OR #15 OR #16 OR #17 OR #18 OR #19 OR #20 OR  #21 OR #22  #22. 'delayed':ti,ab  #21. 'late':ti,ab  #20. 'earlier-start':ti,ab  #19. 'accelerating':ti,ab  #18. 'accelerated':ti,ab  #17. 'time':ti,ab  #16. 'timing':ti,ab  #15. 'early':ti,ab  #14. 'time to treatment'/exp OR 'time to treatment'  #13. 'dialysis':ti,ab  #12. 'renal dialysis':ti,ab  #11. 'hemodialysis'/exp OR 'hemodialysis'  #10. 'hemofiltration':ti,ab  #9. 'renal-replacement therapy':ti,ab  #8. 'renal replacement therapy'/exp OR 'renal  replacement therapy'  #7. #1 OR #2 OR #3 OR #4 OR #5 OR #6  #6. 'kidney tubule necrosis'/exp  #5. 'acute renal failure':ti,ab  #4. 'acute renal injury':ti,ab  #3. 'acute kidney injury':ti,ab  #2. 'acute kidney failure':ti,ab  #1. 'acute kidney failure'/exp |
| --- | --- |
