# Supplementary material for: Selection of timing of continuous renal replacement therapy in patients with acute kidney injury: A meta-analysis of randomized controlled trials
Source: PLoS One. 2025 Mar 25;20(3):e0320351. doi: 10.1371/journal.pone.0320351 (PMC11936205; doi:10.1371/journal.pone.0320351)
Supplement: S2 Table — (DOCX) [file pone.0320351.s010.docx]

**S2 Table. Studies included and excluded**

| **Studies included** | |
| --- | --- |
| 1. An N, Chen R, Bai Y, Xu M. Efficacy and prognosis of continuous renal replacement therapy at different times in the treatment of patients with sepsis-induced acute kidney injury. Am J Transl Res. 2021;13(6):7124-31. 2. Bouman CSC, Oudemans-van Straaten HM, Tijssen JGP, Zandstra DF, Kesecioglu J. Effects of early high-volume continuous venovenous hemofiltration on survival and recovery of renal function in intensive care patients with acute renal failure: A prospective, randomized trial. Crit Care Med. 2002;30(10):2205-11. doi: 10.1097/00003246-200210000-00005 3. Combes A, Bréchot N, Amour J, Cozic N, Lebreton G, Guidon C, et al. Early High-Volume Hemofiltration versus Standard Care for Post-Cardiac Surgery Shock The HEROICS Study. Am J Resp Crit Care. 2015;192(10):1179-90. doi: 10.1164/rccm.201503-0516OC 4. Geri G, Grimaldi D, Seguin T, Lamhaut L, Marin N, Chiche JD, et al. Hemodynamic efficiency of hemodialysis treatment with high cut-off membrane during the early period of post-resuscitation shock: the HYPERDIA trial. Resuscitation. 2019;140:170-7. doi: 10.1016/j.resuscitation.2019.03.045 5. Lumlertgul N, Peerapornratana S, Trakarnvanich T, Pongsittisak W, Surasit K, Chuasuwan A, et al. Early versus standard initiation of renal replacement therapy in furosemide stress test non-responsive acute kidney injury patients (the FST trial). Crit Care. 2018;22(1):101. doi: 10.1186/s13054-018-2021-1 6. Payen D, Mateo J, Cavaillon JM, Fraisse F, Floriot C, Vicaut E. Impact of continuous venovenous hemofiltration on organ failure during the early phase of severe sepsis: a randomized controlled trial. Crit Care Med 2009;37(3):803-10. 7. Srisawat N, Laoveeravat P, Limphunudom P, Lumlertgul N, Peerapornratana S, Tiranathanagul K, et al. The effect of early renal replacement therapy guided by plasma neutrophil gelatinase associated lipocalin on outcome of acute kidney injury: A feasibility study. J Crit Care. 2018;43:36-41. doi: 10.1016/j.jcrc.2017.08.029 8. Sugahara S, Suzuki H. Early start on continuous hemodialysis therapy improves survival rate in patients with acute renal failure following coronary bypass surgery. Hemodial Int. 2004;8(4):320-5. 9. Xia YM, Shi HP, Wu WD, Wang XZ. Effect of urinary NGAL on the timing of renal replacement therapy in patients with acute renal injury associated with sepsis. Medical Journal of Chinese People's Liberation Army. 2019;44(7):605-10. doi: 10.11855/j.issn.0577-7402.2019.07.12 10. Yang T, Zhou R, Yao H, Peng L. Effect of timing of continuous renal replacement therapy on prognosis of sepsis patients with acute renal injury. Mod Instrum Med Treat. 2019;25(05):11-5. 11. Yin JJ, Zheng RJ, Lin H, Shao J. Influence of initiation timing of continuous renal replacement therapy on prognosis of patients with septic shock and acute kidney injury. J Clin Med Pract. 2018;22(07):63-6. 12. Zarbock A, Kellum JA, Schmidt C, Van Aken H, Wempe C, Pavenstädt H, et al. Effect of early vs delayed initiation of renal replacement therapy on mortality in critically ill patients with acute kidney injury: The elain randomized clinical trial. JAMA. 2016;315(20):2190-9. doi: 10.1001/jama.2016.5828 | |
| **Reasons** | | **Studies excluded** | |
| Non-randomized  controlled trial  (n=6) | | 1. Bai L, Luo L, Gao W, Bu C, Huang J. Curative effects of early continuous renal replacement therapy in cardiac failure combined with acute kidney injury. Int J Clin Exp Med. 2020;13(3):1612-9. 2. Crescenzi G, Torracca L, Pierri MD, Rosica C, Munch C, Capestro F. 'Early' and 'late' timing for renal replacement therapy in acute kidney injury after cardiac surgery: a prospective, interventional, controlled, single-centre trial. Interact Cardiov Th. 2015;20(5):616-21. doi: 10.1093/icvts/ivv025. 3. Park JY, An JN, Jhee JH, Kim DK, Oh HJ, Kim S, et al. Early initiation of continuous renal replacement therapy improves survival of elderly patients with acute kidney injury: a multicenter prospective cohort study. Crit Care. 2016(1):260. [doi: 10.1186/s13054-016-1437-8](http://doi.org/10.1186/s13054-016-1437-8) 4. Liu H, Jeng E, Demos D, Vilaro J, Ahmed MM, Parker A, et al. Early versus standard renal replacement therapy after left ventricular assist device implantation. J Cardiac Surg. 2020;35(10):2529-38. [doi: 10.1111/jocs.14873](http://doi.org/10.1111/jocs.14873) 5. Jun M, Bellomo R, Cass A, Gallagher M, Lo S, Lee J. Timing of renal replacement therapy and patient outcomes in the randomized evaluation of normal versus augmented level of replacement therapy study. Crit Care Med. 2014;42(8):1756-65. [doi: 10.1097/CCM.0000000000000343](http://doi.org/10.1097/CCM.0000000000000343) 6. Jun M, Bellomo R, Cass A, Gallagher M, Lo S. Timing of renal replacement therapy and patient outcomes in the randomized evaluation of normal vs. Augmented level of replacement therapy trial. Nephrology. 2012;17:29-30. doi: 10.1111/j.1440-1797.2012.01631.x | |
| Non-continuous renal replacement therapy  (n=7) | | 1. Wald R, Adhikari NKJ, Smith OM, Weir MA, Pope K, Cohen A, et al. Comparison of standard and accelerated initiation of renal replacement therapy in acute kidney injury. Kidney Int. 2015;88(4):897-904. [doi: 10.1038/ki.2015.184](http://doi.org/10.1038/ki.2015.184) 2. Gaudry S, Hajage D, Martin-Lefevre L, Lebbah S, Louis G, Moschietto S, et al. Comparison of two delayed strategies for renal replacement therapy initiation for severe acute kidney injury (AKIKI 2): a multicentre, open-label, randomised, controlled trial. Lancet. 2021;397(10281):1293-300. [doi: 10.1016/S0140-6736(21)00350-0](http://doi.org/10.1016/S0140-6736(21)00350-0) 3. Jamale TE, Hase NK, Kulkarni M, Pradeep KJ, Keskar V, Jawale S, et al. Earlier-start versus usual-start dialysis in patients with community-acquired acute kidney injury: A randomized controlled trial. Am J Kidney Dis. 2013;62(6):1116-21. [doi: 10.1053/j.ajkd.2013.06.012](http://doi.org/10.1053/j.ajkd.2013.06.012) 4. Pursnani ML, Hazra DK, Singh B, Pandey DN. Early haemodialysis in acute tubular necrosis. J Assoc Physicians India. 1997;45(11):850-2. 5. Gaudry S, Hajage D, Schortgen F, Martin-Lefevre L, Pons B, Boulet E, et al. Initiation strategies for renal-replacement therapy in the intensive care unit. New Engl J Med. 2016;375(2):122-33. [doi: 10.1056/NEJMoa1603017](http://doi.org/10.1056/NEJMoa1603017) 6. Bagshaw SM, Wald R, Adhikari NKJ, Bellomo R, Da Costa BR, Dreyfuss D, et al. Timing of initiation of renal-replacement therapy in acute kidney injury. New Engl J Med. 2020;383(3):240-51. [doi: 10.1056/NEJMoa2000741](http://doi.org/10.1056/NEJMoa2000741) 7. Barbar SD, Clere-Jehl R, Bourredjem A, Hernu R, Montini F, Bruyère R, et al. Timing of renal-replacement therapy in patients with acute kidney injury and sepsis. New Engl J Med. 2018;379(15):1431-42. doi: 10.1056/NEJMoa1803213 | |
| No primary outcome  (n=2) | | 1. Jin B, Cao D, Yang N, Wang L, Li R, Liu X, et al. Early high-dose continuous veno-venous hemofiltration alleviates the alterations of CD4+ T lymphocyte subsets in septic patients combined with acute kidney injury. Artif Organs. 2022;46(7):1415-24. [doi: 10.1111/aor.14199](http://doi.org/10.1111/aor.14199) 2. Qu G, Chen F, Xu Q, He Z. Timing of continuous renal replacement therapy in patients with acute non-ST-segment elevation myocardial infarction complicated with cardiac and renal insufficiency. Am J Transl Res. 2021;13(4):3418-26. | |
| Irrelevant studies  (n=2295) | | 1. Tixagevimab-cilgavimab for treatment of patients hospitalised with COVID-19: a randomised, double-blind, phase 3 trial. Lancet Resp Med. 2022(10). http://doi.org/10.1016/S2213-2600(22)00215-6  2. 37th Vicenza Course on AKI and CRRT. Blood Purificat. 2021;50(3).  3. Urologic Society for Transplantation and Renal Surgery 2020 Annual Meeting. Canadian Urological Association Journal. 2020;14(5).  4. National Kidney Foundation 2017 Spring Clinical Meetings Late-Breaking Abstracts. Am J Kidney Dis. 2017;69(5).  5. French Intensive Care Society, International Congress - Reanimation 2016. Ann Intensive Care. 2016;6.  6. 8th International Conference on Pediatric Continuous Renal Replacement Therapy. Pediatr Nephrol. 2015;30(12).  7. 28th Annual Meeting of the European Association of Cardiothoracic Anaesthesiologists, EACTA 2013. Applied Cardiopulmonary Pathophysiology. 2014;17(2):233-4.  8. Abstracts of the 48th Annual Scientific Meeting of the Australian and New Zealand Society of Nephrology, ANZSN 2012. Nephrology. 2012;17.  9. Hemodialysis Abstracts from the Annual Dialysis Conference 30th Annual Conference on Peritoneal Dialysis, 16th International Symposium on Hemodialysis, and 21st Annual Symposium on Pediatric Dialysis. Hemodial Int. 2010;14(1).  10. Randomized, international study of cyclosporine microemulsion absorption profiling in renal transplantation with basiliximab immunoprophylaxis. Am J Transplant. 2002(2). http://doi.org/10.1034/j.1600-6143.2002.020207.x  11. Mycophenolate mofetil in renal transplantation: 3-year results from the placebo-controlled trial. European Mycophenolate Mofetil Cooperative Study Group. Transplantation. 1999(3).  12. Placebo-controlled study of mycophenolate mofetil combined with cyclosporin and corticosteroids for prevention of acute rejection. European Mycophenolate Mofetil Cooperative Study Group. Lancet (London, England). 1995(8961).  13. A A, H A, OF D. Oral prostacycline analog and clopidogrel combination provides early maturation and long-term survival after arteriovenous fistula creation: a randomized controlled study. Indian J Nephrol. 2015(3). http://doi.org/10.4103/0971-4065.139490  14. G A, T S. One litre-one hour exchange for acute peritoneal dialysis as a cost effective approach. Journal of College of Physicians and Surgeons Pakistan. 2007(7).  15. K A, M M, MN Z, W M, M I, T A, et al. Management of Plasma Cell-Rich Acute Rejection in Living-Related Kidney Transplant: role of Proteasome Inhibitor. Exp Clin Transplant. 2019(1). http://doi.org/10.6002/ect.2017.0154  16. TB A, M O, M C, I H, C K, S M, et al. The role of generics in kidney transplant: mycophenolate mofetil 500 versus mycophenolate: 2-year results. Exp Clin Transplant. 2010(4).  17. Abdelaziz TS, Lindenmeyer A, Baharani J, Mistry H, Sitch A, Temple RM, et al. Acute Kidney Outreach to Reduce Deterioration and Death (AKORDD) trial: the protocol for a large pilot study. Bmj Open. 2016;6(8):e012253. http://doi.org/10.1136/bmjopen-2016-012253  18. Abdel-Bakky MS, Aldakhili ASA, Ali HM, Babiker AY, Alhowail AH, Mohammed SAA. Evaluation of Cisplatin-Induced Acute Renal Failure Amelioration Using Fondaparinux and Alteplase. Pharmaceuticals-Base. 2023;16(7). http://doi.org/10.3390/ph16070910  19. MH A, FM F, T T, EL B, EP P. The haemodynamic effects of correction of anaemia in haemodialysis patients using recombinant human erythropoietin. Nephrology, Dialysis, Transplantation. 1990. http://doi.org/10.1093/ndt/5.suppl_1.102  20. Abdullah SS, Rostamzadeh N, Muanda FT, McArthur E, Weir MA, Sontrop JM, et al. High-Throughput Computing to Automate Population-Based Studies to Detect the 30-Day Risk of Adverse Outcomes After New Outpatient Medication Use in Older Adults with Chronic Kidney Disease: A Clinical Research Protocol. Canadian Journal of Kidney Health and Disease. 2024;11. http://doi.org/10.1177/20543581231221891  21. Abe M, Okada K, Suzuki M, Nagura C, Ishihara Y, Fujii Y, et al. Comparison of sustained hemodiafiltration with continuous venovenous hemodiafiltration for the treatment of critically ill patients with acute kidney injury. Artif Organs. 2010;34(4):331-8. http://doi.org/10.1111/j.1525-1594.2009.00861.x  22. Abendroth D, Schneeberger H, Schleibner S, Illner WD, Land W. [Status of treatment with free radical scavengers following kidney and pancreas transplantation]. Zbl Chir. 1992;117(9):502-8.  23. R A, M B, P L, PF W, Le Meur Y, I E, et al. CMV infections after two doses of daclizumab versus thymoglobulin in renal transplant patients receiving mycophenolate mofetil, steroids and delayed cyclosporine A. Nephrology, Dialysis, Transplantation. 2008(6). http://doi.org/10.1093/ndt/gfm873  24. Abraham S, Rameshkumar R, Chidambaram M, Soundravally R, Subramani S, Bhowmick R, et al. Trial of Furosemide to Prevent Acute Kidney Injury in Critically Ill Children: A Double-Blind, Randomized, Controlled Trial. Indian J Pediatr. 2021;88(11):1099-106. http://doi.org/10.1007/s12098-021-03727-3  25. Abu-Amer N, Beckerman P. [TREATMENT OF SEVERE ATHEROSCLEROTIC RENAL ARTERY STENOSIS WITH ACUTE KIDNEY INJURY REQUIRING HEMODIALYSIS BY PERCUTANEOUS TRANSLUMINAL RENAL ANGIOPLASTY AND STENT IMPLANTATION]. Harefuah. 2021;160(4):210-4.  26. Abuelazm MT, Ghanem A, Johanis A, Mahmoud A, Hassan AR, Katamesh BE, et al. Reno-protective effects of perioperative dexmedetomidine in kidney transplantation: a systematic review and meta-analysis of randomized controlled trials. Int Urol Nephrol. 2023;55(10):2545-56. http://doi.org/10.1007/s11255-023-03568-3  27. A A, A H, Y S. Evaluation of HVHF for the treatment of severe acute pancreatitis accompanying MODS. Medicine. 2018(1). http://doi.org/10.1097/MD.0000000000009417  28. A A, IH M. The outcomes of using fixed-dose nomogram-guided unfractionated heparin therapy in elderly patients in comparison with younger patients. Turk Geriatri Dergisi. 2020(3). http://doi.org/10.31086/tjgeri.2020.167  29. Acker CG, Flick R, Shapiro R, Scantlebury VP, Jordan ML, Vivas C, et al. Thyroid hormone in the treatment of post-transplant acute tubular necrosis (ATN). Am J Transplant. 2002;2(1):57-61. http://doi.org/10.1034/j.1600-6143.2002.020110.x  30. Acker CG, Singh AR, Flick RP, Bernardini J, Greenberg A, Johnson JP. A trial of thyroxine in acute renal failure. Kidney Int. 2000;57(1):293-8. http://doi.org/10.1046/j.1523-1755.2000.00827.x  31. ACTRN. A randomised comparative crossover study to assess the affect on circuit life of varying pre-dilution volumes associated with Continuous Veno-Venous Haemofiltration (CVVH) and Continuous Veno-Venous Haemodiafiltration (CVVHDF). Https://Trialsearch.Who.Int/Trial2.Aspx?TrialID=ACTRN12606000045516. 2006.  32. ACTRN. Early Intervention in Acute Renal Failure. Https://Trialsearch.Who.Int/Trial2.Aspx?TrialID=ACTRN12606000058572. 2006.  33. ACTRN. Prospective randomised cross-over trial of regional citrate with heparin for anticoagulation and additional albumin prime in continuous venovenous haemofiltration in children. Http://Www.Who.Int/Trialsearch/Trial2.Aspx?TrialID=ACTRN12608000462381. 2008.  34. ACTRN. Nephro-protective effects of L-amino acids in critically ill patients. A phase II multicentre randomised controlled trial. Https://Trialsearch.Who.Int/Trial2.Aspx?TrialID=ACTRN12609001015235. 2009.  35. ACTRN. A randomised controlled study comparing the effect of two different anticoagulation regimens on filter life during Continuous Renal Replacement Therapy (CRRT) – The Heparin Citrate (THC) Study. Https://Trialsearch.Who.Int/Trial2.Aspx?TrialID=ACTRN12609001079235. 2009.  36. ACTRN. A randomized trial to compare efficacy and safety of cyclosporin-prednisone versus mycophenolate mofetil-prednisone, 3 months after renal transplantation. Https://Trialsearch.Who.Int/Trial2.Aspx?TrialID=ACTRN12610000070033. 2006.  37. ACTRN. A multicenter open label study to compare efficacy and safety of induction treatment by ATG (thymoglobulin) versus anti-IL-2R (daclizumab) with a triple drug regimen (tacrolimus, mycophenolate mofetil, prednisone) in high risk renal transplant recipients. Http://Www.Who.Int/Trialsearch/Trial2.Aspx?TrialID=ACTRN12610000071022. 2006.  38. ACTRN. Efficacy and Safety of Alemtuzumab for prevention of graft rejection and preservation of renal function in patients receiving Kidney Transplant. Https://Trialsearch.Who.Int/Trial2.Aspx?TrialID=ACTRN12610000837022. 2010.  39. ACTRN. The Effect of Short Term Dialysis Catheter Position on Dialyser Lifespan. Unblinded, randomised, controlled trial to assess the effect of placing the proximal tip placement of a short term dialysis catheters on continuous renal replacement (CRRT) circuit lifespan in intensive care unit (ICU) patients with severe acute kidney injury (AKI). Https://Trialsearch.Who.Int/Trial2.Aspx?TrialID=ACTRN12611000759998. 2011.  40. ACTRN. Balanced fluid therapy and early kidney function in patients undergoing renal transplantation. Https://Trialsearch.Who.Int/Trial2.Aspx?TrialID=ACTRN12612000023853. 2012.  41. ACTRN. Filter Life In Renal Replacement Therapy. Http://Www.Who.Int/Trialsearch/Trial2.Aspx?TrialID=ACTRN12612000765820. 2012.  42. ACTRN. A pilot, randomized, blinded, multi-centre, feasibility, safety and biochemical and physiological study of normal saline versus plasmalyte in intensive therapy. Https://Trialsearch.Who.Int/Trial2.Aspx?TrialID=ACTRN12615000158561. 2015.  43. ACTRN. Factor concentrates versus Allogeneic blood in CardioThoracic Surgery trial. Http://Www.Who.Int/Trialsearch/Trial2.Aspx?TrialID=ACTRN12615000315516. 2015.  44. ACTRN. Conservative versus liberal fluid therapy in patients with liver disease admitted to the intensive care unit. Http://Www.Who.Int/Trialsearch/Trial2.Aspx?TrialID=ACTRN12615000377538. 2015.  45. ACTRN. Renal Replacement Therapy Intensity in Severe Acute Kidney Injury: an Individual Patient Data Meta-analysis of Randomized Trials. Https://Trialsearch.Who.Int/Trial2.Aspx?TrialID=ACTRN12615000394549. 2015.  46. ACTRN. The impact of prescribed blood flow rate on circuit life in critically ill patients receiving continuous renal replacement therapy (CRRT) in intensive care. Http://Www.Who.Int/Trialsearch/Trial2.Aspx?TrialID=ACTRN12615001353583. 2015.  47. ACTRN. A prospective randomised study conducted in an adult intensive care unit to compare the biochemical and acid-base effects of two solutions used during continuous renal replacement therapy (CRRT). Http://Www.Who.Int/Trialsearch/Trial2.Aspx?TrialID=ACTRN12616000045415. 2016.  48. ACTRN. REDUcing the burden of dialysis Catheter ComplicaTIOns in patients with kidney disease: a National approach. Http://Www.Who.Int/Trialsearch/Trial2.Aspx?TrialID=ACTRN12616000830493. 2016.  49. ACTRN. Paracetamol or Ibuprofen in the Primary Prevention of Asthma in Tamariki (PIPPA Tamariki). Https://Trialsearch.Who.Int/Trial2.Aspx?TrialID=ACTRN12618000303246. 2018.  50. ACTRN. A pilot randomized study comparing an approach of individualized blood pressure targets to standard care among critically ill patients with shock. Https://Trialsearch.Who.Int/Trial2.Aspx?TrialID=ACTRN12618000571279. 2018.  51. ACTRN. Can we reduce cerebral injury in patients undergoing combined aortic valve replacement and coronary artery bypass grafting? Http://Www.Who.Int/Trialsearch/Trial2.Aspx?TrialID=ACTRN12618001142224. 2018.  52. ACTRN. Australasian Follow-up Tracking and Enhancing Recovery after Acute Kidney Injury (AFTER-AKI). Https://Trialsearch.Who.Int/Trial2.Aspx?TrialID=ACTRN12618002044202. 2018.  53. ACTRN. comparing circuit life between modified membrane and standard membrane in liver failure patients. Https://Trialsearch.Who.Int/Trial2.Aspx?TrialID=ACTRN12619000788178. 2019.  54. ACTRN. A pilot clinical study of Vitamin C for the prevention of acute kidney injury in critically ill patients. Https://Trialsearch.Who.Int/Trial2.Aspx?TrialID=ACTRN12619000909123. 2019.  55. ACTRN. Comparison of three intravenous fluids used in the treatment of children admitted to the paediatric intensive care unit. Https://Trialsearch.Who.Int/Trial2.Aspx?TrialID=ACTRN12619001244190. 2019.  56. ACTRN. Albumin infusion for kidney protection after heart surgery. Https://Trialsearch.Who.Int/Trial2.Aspx?TrialID=ACTRN12619001355167. 2019.  57. ACTRN. What frozen elephant trunk prosthesis is safe and effective in the treatment thoracic aortic disease. Https://Trialsearch.Who.Int/Trial2.Aspx?TrialID=ACTRN12620000123943. 2020.  58. ACTRN. Haemofiltration with OXiris: multicentre Evaluation in Acute Kidney Injury (HOME-AKI). A pilot study comparing two devices used for renal replacement therapy in the intensive care unit in patients with acute kidney injury related to sepsis. Https://Trialsearch.Who.Int/Trial2.Aspx?TrialID=ACTRN12620000514909. 2020.  59. ACTRN. Evaluation of mega-dose Vitamin C for patients with severe infections who are admitted to the intensive care unit. Https://Trialsearch.Who.Int/Trial2.Aspx?TrialID=ACTRN12620000651987. 2020.  60. ACTRN. SODium BICarbonate for metabolic acidosis in the Intensive Care Unit: a pilot, multicentre, double-blind, placebo controlled randomized clinical trial. Https://Trialsearch.Who.Int/Trial2.Aspx?TrialID=ACTRN12620001361998. 2020.  61. ACTRN. NEt ultrafiltration Prescription and Targeting versus Usual NEt ultrafiltration during continuous renal replacement therapy. Https://Trialsearch.Who.Int/Trial2.Aspx?TrialID=ACTRN12621001566820. 2021.  62. ACTRN. Does conservative fluid therapy compared to usual care change the outcomes in critically ill patients with acute kidney injury. Https://Trialsearch.Who.Int/Trial2.Aspx?TrialID=ACTRN12622000030774. 2022.  63. ACTRN. Acute Kidney Injury in patients randomised to the ARISE FLUIDS Randomised Controlled Trial. Https://Trialsearch.Who.Int/Trial2.Aspx?TrialID=ACTRN12622000988752. 2022.  64. ACTRN. A Pilot, Safety, Feasibility and Efficacy Trial of Hemoperfusion during Continuous Renal Replacement Therapy in Critically Ill Patients with Combined Liver and Kidney Failure. Https://Trialsearch.Who.Int/Trial2.Aspx?TrialID=ACTRN12622001040752. 2022.  65. ACTRN. Investigating the effect of a new agent on heart disease and kidney function in people with type 2 diabetes who have recovered from acute kidney injury. Https://Trialsearch.Who.Int/Trial2.Aspx?TrialID=ACTRN12622001467729. 2022.  66. ACTRN. Individualising beta-lactam antibiotic dosing in septic intensive care unit patients based on plasma concentration measurements using therapeutic drug monitoring. Https://Trialsearch.Who.Int/Trial2.Aspx?TrialID=ACTRN12623000032651. 2023.  67. ACTRN. A pilot study of pulmonary artery catheters in low-risk heart surgery. Https://Trialsearch.Who.Int/Trial2.Aspx?TrialID=ACTRN12623000522617. 2023.  68. ACTRN. A randomised trial of blood pressure maintenance using angiotensin II versus noradrenaline in cardiac surgery patients to determine effect on length of hospital stay. Https://Trialsearch.Who.Int/Trial2.Aspx?TrialID=ACTRN12623000848606. 2023.  69. ACTRN. A single-centre feasibility study to evaluate the ability of Phoxilium to prevent hypophoshatemia compared to Hemosol B0 during continuous renal replacement therapy in the intensive care unit. Https://Trialsearch.Who.Int/Trial2.Aspx?TrialID=ACTRN12623001152617. 2023.  70. ACTRN. Intervention with concentrated albumin for resuscitation of undifferentiated sepsis - Multi Day. Https://Trialsearch.Who.Int/Trial2.Aspx?TrialID=ACTRN12624000327583. 2024.  71. M A, L J, K L, F H, F S, R K, et al. A prospective comparison of bladder versus enteric drainage in vascularized pancreas transplantation. Transpl P. 2004(4). http://doi.org/10.1016/j.transproceed.2004.05.027  72. MB A. Enisoprost in renal transplantation. The Enisoprost Renal Transplant Study Group. Transplantation. 1992(2).  73. Adelman MH, Iturrate E. IgA nephropathy presenting as a rapidly progressive glomerulonephritis in an elderly woman. J Gen Intern Med. 2014;29:S371.  74. D A, B S, TA A, WY K, ES A, CA M. Comparison of the Effects of Target-Controlled Infusion of Propofol and Sevoflurane as Maintenance of Anesthesia on Hemodynamic Profile in Kidney Transplantation. Anesthesiol Res Prac. 2019. http://doi.org/10.1155/2019/5629371  75. Adrie C, Monchi M. High volume hemofiltration in inflammatory shocks: Potential interests and hazards. Reanimation. 2005;14(6):528-33. http://doi.org/10.1016/j.reaurg.2005.09.005  76. B A, RB C, F S, MP B, DR T, D M, et al. Randomized, controlled clinical trial of the DIALIVE liver dialysis device versus standard of care in patients with acute-on- chronic liver failure. J Hepatol. 2023(1). http://doi.org/10.1016/j.jhep.2023.03.013  77. A A, S A, T K, Y G. Rifampicin and anti-hypertensive drugs in chronic kidney disease: pharmacokinetic interactions and their clinical impact. Indian J Nephrol. 2016(5). http://doi.org/10.4103/0971-4065.176145  78. Ahmed SS, Khan M. Acute Kidney Injury in Patients with Diabetic Ketoacidosis at National Institute of Child Health. Pakistan Journal of Medical and Health Sciences. 2022;16(12):499-501. http://doi.org/10.53350/pjmhs20221612499  79. N A, MJ H, MV J, MS R, HC Y. Limited dose monoclonal IL-2R antibody induction protocol after primary kidney transplantation. Am J Transplant. 2002(6). http://doi.org/10.1034/j.1600-6143.2002.20612.x  80. N A, D H, A M, S R, S T, A W, et al. Prednisone withdrawal in kidney transplant recipients on cyclosporine and mycophenolate mofetil--a prospective randomized study. Steroid Withdrawal Study Group. Transplantation. 1999(12). http://doi.org/10.1097/00007890-199912270-00009  81. Ahsan N, Johnson C, Gonwa T, Halloran P, Stegall M, Hardy M, et al. Randomized trial of tacrolimus plus mycophenolate mofetil or azathioprine versus cyclosporine oral solution (modified) plus mycophenolate mofetil after cadaveric kidney transplantation: results at 2 years. Transplantation. 2001;72(2):245-50. http://doi.org/10.1097/00007890-200107270-00014  82. Ai QJ, Liu LP, Shuai DK, Deng YY, Wang LJ, Zhou J. Effect of alprostadil on acute kidney injury after resuscitation to standard state for septic shock. Medical Journal of Chinese People's Liberation Army. 2018;43(6):499-503. http://doi.org/10.11855/j.issn.0577-7402.2018.06.09  83. D A, V A, T D, N G, TM L, G N, et al. Use of Remdesivir in Patients With COVID-19 on Hemodialysis: a Study of Safety and Tolerance. Kidney Int Rep. 2021(3). http://doi.org/10.1016/j.ekir.2020.12.003  84. C A, E K, T S, E A, T B, Y K, et al. Effects of chronic HCV infection on graft and patient survival in renal transplant recipients. Nephrol Dial Transpl. 2015. http://doi.org/10.1093/ndt/gfv202.4  85. Akil A, Ziegeler S, Rehers S, Ernst EC, Fischer S. Blood purification therapy in patients with severe COVID-19 requiring veno-venous ECMO therapy: A retrospective study. Int J Artif Organs. 2022;45(7):615-22. http://doi.org/10.1177/03913988221103287  86. T A, Y T, H H, Y W, H H, S N, et al. A prospective observational study of early intervention with erythropoietin therapy and renal survival in non-dialysis chronic kidney disease patients with anemia: JET-STREAM Study. Clin Exp Nephrol. 2016(6). http://doi.org/10.1007/s10157-015-1225-9  87. AM A, KD O, M M, Y Y, C C, K S, et al. Personalised recommendations for hospitalised patients with Acute Kidney Injury using a Kidney Action Team (KAT-AKI): protocol and early data of a randomised controlled trial. Bmj Open. 2023(4). http://doi.org/10.1136/bmjopen-2023-071968  88. EA A, Z K, A D, G M, G A. Early postoperative renal function following renal transplantation surgery: effect of anesthetic technique. J Anesth. 2002(2). http://doi.org/10.1007/s005400200004  89. E A, E S. Treatment of alcoholic hepatitis: is this a “dead-end”? Ann Gastroenterol. 2016(2). http://doi.org/10.20524/aog.2016.0023  90. Al AZ, Lodewyk K, Robinson D, Qureshi S, Kabbani LS, Sullivan B, et al. Contrast-induced nephropathy after peripheral vascular intervention: Long-term renal outcome and risk factors for progressive renal dysfunction. J Vasc Surg. 2019;69(3):913-20. http://doi.org/10.1016/j.jvs.2018.06.196  91. Alamartine E, De Filippis JP, Toulon J, Berthoux F. On-line continuous venovenous hemodiafiltration: A technique for the control of ultrafiltration and convection during continuous renal replacement therapy. Renal Failure. 1994;16(6):707-14.  92. L A, B B, F L, M G, O V, S S, et al. Outcomes with Tacrolimus-Based Immunosuppression After Kidney Transplantation from Standard- and Extended-Criteria Donors - A Post Hoc Analysis of the Prospective OSAKA Study. Ann Transpl. 2020. http://doi.org/10.12659/AOT.920041  93. BB A, AL B, JM A, D P. Dialysis complications in acute kidney injury patients treated with prolonged intermittent renal replacement therapy sessions lasting 10 versus 6 hours: results of a randomized clinical trial. Artif Organs. 2015(5). http://doi.org/10.1111/aor.12408  94. Albrecht F, Schunk S, Fuchs M, Volk T, Geisel J, Fliser D, et al. Rapid and Effective Elimination of Myoglobin with CytoSorb® Hemoadsorber in Patients with Severe Rhabdomyolysis. Blood Purificat. 2024;53(2):88-95. http://doi.org/10.1159/000534479  95. Aldemir M, Koca HB, Bakı ED, Çarşanba G, Kavrut NÖ, Kavaklı AS, et al. Effects of N-acetyl cysteine on renal functions evaluated by blood neutrophil gelatinase-associated lipocalin levels in geriatric patients undergoing coronary artery bypass grafting. Anatol J Cardiol. 2016;16(7):504-11. http://doi.org/10.5152/AnatolJCardiol.2015.6287  96. JW A, HR G, M C, J A, S G, S S, et al. Simultaneous corticosteroid avoidance and calcineurin inhibitor minimization in renal transplantation. Transpl Int. 2006(4). http://doi.org/10.1111/j.1432-2277.2006.00280.x  97. Alghamdi AM, Alghamdi AM, Alsemairi SAB, Alangari AA, Alabbad MA, Alebrahim EA, et al. Sepsis-associated Acute Kidney Injury, Diagnosis, and Management, Review Article. Int J Pharm Res Alli. 2021;10(3):129-36. http://doi.org/10.51847/A6DBTGLKCE  98. Al-Hwiesh A, Abdul-Rahman I, Finkelstein F, Divino-Filho J, Qutub H, Al-Audah N, et al. Acute Kidney Injury in Critically Ill Patients: A Prospective Randomized Study of Tidal Peritoneal Dialysis Versus Continuous Renal Replacement Therapy. Ther Apher Dial. 2018;22(4):371-9. http://doi.org/10.1111/1744-9987.12660  99. Alhwiesh A, Nasreldin MA, Saeed I. Comparing tidal peritoneal dialysis to crrt in critically ill patientswith acute kidney injury. A single centre experience. Nephrol Dial Transpl. 2014;29:iii226. http://doi.org/10.1093/ndt/gfu154  100. H A, A S, A M, L H, A A. Effect of low dose aspirin therapy on renal graft function and long term graft survival. Nephrol Dial Transpl. 2015. http://doi.org/10.1093/ndt/gfv202.18  101. Alkhatib Y, Dadla A, Malik D, Abdel-Rahman Z, Kuriakose P, Dabak VS, et al. Plasmapheresis and myeloma cast nephropathy: A meta-analysis and review of evidence. Blood. 2017;130.  102. Alkhunaizi AM, Schrier RW. Management of acute renal failure: New perspectives. Am J Kidney Dis. 1996;28(3):315-28. http://doi.org/10.1016/S0272-6386(96)90487-4  103. V A, L M, A V. Lipid and apolipoprotein patterns during erythropoietin therapy: roles of erythropoietin, route of administration, and diet. Nephrology, Dialysis, Transplantation. 1997(5). http://doi.org/10.1093/ndt/12.5.924  104. MJ A, J K, J W, C C, UG P, R H, et al. Early goal-directed nutrition in ICU patients (EAT-ICU): protocol for a randomised trial. Dan Med J. 2016(9).  105. F A, K Z, CS R, M A. Incidence and outcomes of early percutaneous coronary intervention after isolated valve surgery. Catheter Cardio Inte. 2019(4). http://doi.org/10.1002/ccd.27874  106. YS A, DW K, B D, MP K, JC P. Vancomycin Area under the Concentration-Time Curve Estimation Using Bayesian Modeling versus First-Order Pharmacokinetic Equations: a Quasi-Experimental Study. Antibiotics. 2022(9). http://doi.org/10.3390/antibiotics11091239  107. P A, G S, P B, E A, I L, M C, et al. Predilution haemofiltration--the Second Sardinian Multicentre Study: comparisons between haemofiltration and haemodialysis during identical Kt/V and session times in a long-term cross-over study. Nephrology, Dialysis, Transplantation. 2001(6). http://doi.org/10.1093/ndt/16.6.1207  108. Alves N, Mota M, Cunha M, Ribeiro JM. Impact of emergent coronary angiography after out-of-the-hospital cardiac arrest without ST-segment elevation – A systematic review and meta-analysis. Int J Cardiol. 2022;364:1-8. http://doi.org/10.1016/j.ijcard.2022.06.006  109. Amaechi P, Lartey KA. Paradigm shift: Sepsis-induced aki not due to hypotension. J Am Soc Nephrol. 2019;30:1084.  110. Amdisen C, Keller AK, Pedersen M, Nørrega Rd R, Birn H, Jespersen B. Danegaptide does not improve renal function after ischemia/reperfusion injury in a porcine model. Nephrol Dial Transpl. 2015;30:iii138-9. http://doi.org/10.1093/ndt/gfv172.44  111. Amendola CP, Silva-Jr JM, Carvalho T, Sanches LC, Silva UVAE, Almeida R, et al. Goal-directed therapy in patients with early acute kidney injury: a multicenter randomized controlled trial. Clinics (Sao Paulo, Brazil). 2018;73:e327. http://doi.org/10.6061/clinics/2018/e327  112. Amini S, Robabi HN, Tashnizi MA, Vakili V. Selenium, Vitamin C and N-Acetylcysteine do not Reduce the Risk of Acute Kidney Injury after Off-Pump CABG: a Randomized Clinical Trial. Braz J Cardiov Surg. 2018;33(2):129-34. http://doi.org/10.21470/1678-9741-2017-0071  113. An KR, Rahman IA, Tam DY, Ad N, Verma S, Fremes SE, et al. A Systematic Review and Meta-Analysis of del Nido Versus Conventional Cardioplegia in Adult Cardiac Surgery. Innovations: Technology and Techniques in Cardiothoracic and Vascular Surgery. 2019;14(5):385-93. http://doi.org/10.1177/1556984519863718  114. An MM, Liu CX, Gong P. Effects of continuous renal replacement therapy on inflammation-related anemia, iron metabolism and prognosis in sepsis patients with acute kidney injury. World J Emerg Med. 2023;14(3):136-42. http://doi.org/10.5847/wjem.j.1920-8642.2023.052  115. TJ A, YH S, J H, ME H, S V, L S, et al. Effects of folinic acid on forearm blood flow in patients with end-stage renal disease. Nephrology, Dialysis, Transplantation. 2006(7). http://doi.org/10.1093/ndt/gfl083  116. J A, AM C, E R, E C, JI C, E G, et al. Donor-specific antibodies development in renal living-donor receptors: effect of a single cohort. Int J Immunopath Ph. 2021. http://doi.org/10.1177/20587384211000545  117. Angeli P, Bezinover D, Biancofiore G, Bienholz A, Findlay J, Burtz CP, et al. Acute kidney injury in liver transplant candidates: A position paper on behalf of the Liver Intensive Care Group of Europe. Minerva Anestesiol. 2017;83(1):88-101. http://doi.org/10.23736/S0375-9393.16.11661-X  118. MS AK, M IS, K R, G M, N S, AM K, et al. Comparison of four different immunosuppression protocols without long-term steroid therapy in kidney recipients monitored by surveillance biopsy: five-year outcomes. Transpl Immunol. 2008(1‐2). http://doi.org/10.1016/j.trim.2008.08.005  119. C A, V C, A R. A Comparison between Two Dilute Citrate Solutions (15 vs. 18 mmol/l) in Continuous Renal Replacement Therapy: the Base Excess and Renal Substitution Solution Study. Blood Purificat. 2016(3). http://doi.org/10.1159/000446979  120. Antonic M. Effect of ascorbic acid on postoperative acute kidney injury in coronary artery bypass graft patients: A pilot study. Heart Surg Forum. 2017;20(5):E214-8. http://doi.org/10.1532/HSF98.20171811  121. Anwar F, Sroya HA, Al-Odat RT, Shaffi SK. Acute Kidney Injury due to Epstein-Barr Virus. J Am Soc Nephrol. 2016;27:1014A.  122. M A, G S, C B, E Y, K G, RM K, et al. Erythropoietin in Acute Kidney Injury (EAKI): a pragmatic randomized clinical trial. Bmc Nephrol. 2022(1). http://doi.org/10.1186/s12882-022-02727-5  123. H A, J M, WD I, A T, C D, R L, et al. Rejection after simultaneous pancreas-kidney transplantation. Nephrology, Dialysis, Transplantation. 2005. http://doi.org/10.1093/ndt/gfh1077  124. Arcangeli A, Rocca B, Salvatori G, Ciancia M, De Cristofaro R, Antonelli M. Heparin versus prostacyclin in continuous hemodiafiltration for acute renal failure: Effects on platelet function in the systemic circulation and across the filter. Thromb Res. 2010;126(1):24-31. http://doi.org/10.1016/j.thromres.2010.01.048  125. Arend J, Peters E, Mehta RL, Murray PT, Hummel J, Joannidis M, et al. Design of the STOP-AKI Trial: Safety, Tolerability, Efficacy and Quality of Life of Human Recombinant Alkaline Phosphatase in Patients with Sepsis-Associated Acute Kidney Injury. J Am Soc Nephrol. 2016;27:567A.  126. FA A, DA I, AA S, A A. An analysis of the effectiveness and benefits of peritoneal dialysis and haemodialysis using Nigerian made PD fluids. African Journal of Medicine and Medical Sciences. 2005(3).  127. Arora V, Maiwall R, Choudhury A, Jain P, Kumar G, Sarin SK. Terlipressin is superior to noradrenaline in management of acute kidney injury in acute on chronic liver failure. Indian J Gastroenter. 2017;36(1):A7-8. http://doi.org/10.1007/s12664-017-0798-5  128. Arrivi A, Morgantini A, Rosati R, Pucci G, Placanica A, Sordi M, et al. GLUTATHIONE INFUSION FOR COUNTERACTING CONTRASTASSOCIATED ACUTE KIDNEY INJURY IN PATIENTS WITH ST-ELEVATION MYOCARDIAL INFARCTION UNDERGOING PRIMARY PCI. G Ital Cardiol. 2023;24(10):e31.  129. Arrivi A, Pucci G, Sordi M, Dominici M, Barillà F, Carnevale R, et al. Repeated Glutathione Sodium Salt Infusion May Counteract Contrast-Associated Acute Kidney Injury Occurrence in ST-Elevation Myocardial Infarction Patients Undergoing Primary PCI: A Randomized Subgroup Analysis of the GSH 2014 Trial. Life-Basel. 2023;13(6). http://doi.org/10.3390/life13061391  130. Ashuntantang G, Miljeteig I, Luyckx VA. Bedside rationing and moral distress in nephrologists in sub- Saharan Africa. Bmc Nephrol. 2022;23(1). http://doi.org/10.1186/s12882-022-02827-2  131. Aslan AT, Pashayev T, Daǧ O, Akova M. Tecioplanin (TEI) vs. vancomycin (VAN) in combination with piperacillin-tazobactam (TZP) or meropenem (MER) as a cause of acute kidney injury (AKI). Open Forum Infect Di. 2018;5:S434-5. http://doi.org/10.1093/ofid/ofy210.1242  132. Atan R, May C, Bailey SR, Tanudji M, Visvanathan K, Skinner N, et al. Nucleosome levels and toll-like receptor expression during high cut-off haemofiltration: a pilot assessment. Crit Care Resusc. 2015;17(4):239-43.  133. Atan R, Peck L, Prowle J, Licari E, Eastwood GM, Storr M, et al. A double-blind randomized controlled trial of high cutoff versus standard hemofiltration in critically ill patients with acute kidney injury. Crit Care Med. 2018;46(10):E988-94. http://doi.org/10.1097/CCM.0000000000003350  134. Atan R, Peck L, Visvanathan K, Skinner N, Eastwood G, Bellomo R, et al. High cut-off hemofiltration versus standard hemofiltration: Effect on plasma cytokines. Int J Artif Organs. 2016;39(9):479-86. http://doi.org/10.5301/ijao.5000527  135. Atapour A, Shahidi S. Does tunneling the temporary vascular access extend its lifetime? J Res Med Sci. 2006;11(1):41-7.  136. Atreya MR, Cvijanovich NZ, Fitzgerald JC, Weiss SL, Bigham MT, Jain PN, et al. Revisiting Post-ICU Admission Fluid Balance Across Pediatric Sepsis Mortality Risk Strata: A Secondary Analysis of a Prospective Observational Cohort Study. Crit Care Explor. 2024;6(1):e1027. http://doi.org/10.1097/CCE.0000000000001027  137. M A, J W, A W, K R, M M, F H, et al. A randomized trial of three iron dextran infusion methods for anemia in EPO-treated dialysis patients. Am J Kidney Dis. 1998(1). http://doi.org/10.1053/ajkd.1998.v31.pm9428456  138. Augustine JJ, Sandy D, Seifert TH, Paganini EP. A randomized controlled trial comparing intermittent with continuous dialysis in patients with ARF. Am J Kidney Dis. 2004;44(6):1000-7. http://doi.org/10.1053/j.ajkd.2004.08.022  139. G B, H K, E S, FS T, S M. Mechanical chest compressions are associated with increased severity of post-cardiac arrest syndrome: a sub-study from the TTH48 trial. Intens Care Med Exp. 2020(SUPPL 2). http://doi.org/10.1186/s40635-020-00354-8  140. K B, GA J, A J, DR G, WA J. Molecular and structural consequences of early renal allograft injury. Kidney Int. 2002(2). http://doi.org/10.1046/j.1523-1755.2002.00149.x  141. M B, T H, J B, B T, B S, B T, et al. A prospective pilot trial to assess the efficacy of argatroban (argatra®) in critically ill patients with heparin resistance. J Clin Med. 2020(4). http://doi.org/10.3390/jcm9040963  142. S B, AB M, BS C, KR J, DL S. Machine learning to predict transplant outcomes: helpful or hype? A national cohort study. Transpl Int. 2020(11). http://doi.org/10.1111/tri.13695  143. CH B, CD K, DR L, YH K, J Y, BS K, et al. Randomized, Open-Label, Phase IV, Korean Study of Kidney Transplant Patients Converting From Cyclosporine to Prolonged-Release Tacrolimus Plus Standard- or Reduced-Dose Corticosteroids. Transpl P. 2019(3). http://doi.org/10.1016/j.transproceed.2019.01.057  144. Bagshaw SM. Epidemiology of renal recovery after acute renal failure. Curr Opin Crit Care. 2006;12(6):544-50. http://doi.org/10.1097/01.ccx.0000247444.63758.0b  145. Bagshaw SM, Bellomo R, Devarajan P, Johnson C, Karvellas CJ, Kutsiogiannis DJ, et al. Review article: Acute kidney injury in critical illness. Canadian Journal of Anesthesia. 2010;57(11):985-98. http://doi.org/10.1007/s12630-010-9375-4  146. Bagshaw SM, Berthiaume LR, Delaney A, Bellomo R. Continuous versus intermittent renal replacement therapy for critically ill patients with acute kidney injury: A meta-analysis. Crit Care Med. 2008;36(2):610-7. http://doi.org/10.1097/01.CCM.0B013E3181611F552  147. Bagshaw SM, Gibney RN, McAlister FA, Bellomo R. The SPARK Study: A phase II randomized blinded controlled trial of the effect of furosemide in critically ill patients with early acute kidney injury. Trials. 2010;11. http://doi.org/10.1186/1745-6215-11-50  148. Bagshaw SM, Gibney RTN, Kruger P, Hassan I, McAlister FA, Bellomo R. SPARK study-low-dose furosemide in critically ill patients with early acute kidney injury: A pilot randomized controlled trial. Am J Kidney Dis. 2017;69(5):A2. http://doi.org/10.1053/j.ajkd.2017.03.005  149. Bagshaw SM, Gibney RTN, Kruger P, Hassan I, McAlister FA, Bellomo R. The effect of low-dose furosemide in critically ill patients with early acute kidney injury: A pilot randomized blinded controlled trial (the SPARK study). J Crit Care. 2017;42:138-46. http://doi.org/10.1016/j.jcrc.2017.07.030  150. Bagshaw SM, Laupland KB, Boiteau PJE, Godinez-Luna T. Is regional citrate superior to systemic heparin anticoagulation for continuous renal replacement therapy? a prospective observational study in an adult regional critical care system. J Crit Care. 2005;20(2):155-61. http://doi.org/10.1016/j.jcrc.2005.01.001  151. Bagshaw SM, Neyra JA, Tolwani AJ, Wald R. Debate: Intermittent Hemodialysis versus Continuous Kidney Replacement Therapy in the Critically Ill Patient: The Argument for CKRT. Clin J Am Soc Nephro. 2023;18(5):647-60. http://doi.org/10.2215/CJN.0000000000000056  152. Bagshaw SM, Wald R, Barton J, Burns KEA, Friedrich JO, House AA, et al. Clinical factors associated with initiation of renal replacement therapy in critically ill patients with acute kidney injury-A prospective multicenter observational study. J Crit Care. 2012;27(3):268-75. http://doi.org/10.1016/j.jcrc.2011.06.003  153. Balachandran M, Banneheke P, Pakavakis A, Al-Bassam W, Sarode V, Rowland M, et al. Postoperative 20% albumin vs standard care and acute kidney injury after high-risk cardiac surgery (ALBICS): study protocol for a randomised trial. Trials. 2021;22(1). http://doi.org/10.1186/s13063-021-05519-8  154. Balbé H, Bastos-Bueno A, Luz A, Miyamoto M, Ferreira H, Zamprogna A, et al. WCN24-2144 THE BRAZILIAN RANDOMIZED CONTROLLED UNBLINDED PILOT TRIAL OF URGENT START PERITONEAL DIALYSIS – THE BRUTUS TRIAL INITIAL RESULTS. Kidney Int Rep. 2024;9(4):S603-4. http://doi.org/10.1016/j.ekir.2024.02.1268  155. I B, HK T, N B, R B. Possible strategies to prolong circuit life during hemofiltration: three controlled studies. Renal Failure. 2002(6). http://doi.org/10.1081/jdi-120015685  156. Baldwin I, Bellomo R, Naka T, Koch B, Fealy N. A pilot randomized controlled comparison of extended daily dialysis with filtration and continuous veno-venous hemofiltration: Fluid removal and hemodynamics. Int J Artif Organs. 2007;30(12):1083-9. http://doi.org/10.1177/039139880703001208  157. A B, MA C, L MB, I T, SM M, L B, et al. Erythropoietin in children with hemolytic uremic syndrome: a pilot randomized controlled trial. Pediatric Nephrology (Berlin, Germany). 2022(10). http://doi.org/10.1007/s00467-022-05474-9  158. J B. Thyroxine in ARF. Critical Care (London, England). 2000(1). http://doi.org/10.1186/ccf-2000-4857  159. Bank JR, Ruhaak R, Soonawala D, Mayboroda O, Romijn FP, van Kooten C, et al. Urinary TIMP-2 Predicts the Presence and Duration of Delayed Graft Function in Donation After Circulatory Death Kidney Transplant Recipients. Transplantation. 2019;103(5):1014-23. http://doi.org/10.1097/TP.0000000000002472  160. Baptista A, Banheiro B, Ávila A, Campello G. Renal replacement therapy with citrate in different doses (CiDoSes) study. Intens Care Med Exp. 2018;6. http://doi.org/10.1186/s40635-018-0201-6  161. JD B, PC D, C P, LV L, J S, De Broe ME. Low-dose (5 mg/kg) desferrioxamine treatment in acutely aluminium-intoxicated haemodialysis patients using two drug administration schedules. Nephrology, Dialysis, Transplantation. 1996(1).  162. M B, S G, P C, S I, C S, C T, et al. Acute Kidney Injury With the RenalGuard System in Patients Undergoing Transcatheter Aortic Valve Replacement: the PROTECT-TAVI Trial (PROphylactic effecT of furosEmide-induCed diuresis with matched isotonic intravenous hydraTion in Transcatheter Aortic Valve Implantation). JACC. Cardiovascular Interventions. 2015(12). http://doi.org/10.1016/j.jcin.2015.07.012  163. Barcia R, Nguyen S, O'Rourke B, Igo P, Tilles A, Miller B, et al. Immune Reprogramming in Human Subjects after Extracorporeal Mesenchymal Stromal Cell Therapy. Cytotherapy. 2020;22(5):S11. http://doi.org/10.1016/j.jcyt.2020.03.473  164. S B, R B, G C, A F, B G, U H, et al. Enoxaparin for primary thromboprophylaxis in ambulatory patients with coronavirus disease-2019 (the OVID study): a structured summary of a study protocol for a randomized controlled trial. Trials. 2020(1). http://doi.org/10.1186/s13063-020-04678-4  165. Barodka V, Silvestry S, Sharifi-Azad S, Diehl J, Grunwald Z, Sun J. Effects of preoperative diuretic therapy on renal function after cardiac surgery. Anesth Analg. 2010;110(3):S102. http://doi.org/10.1213/01.ANE.0000398215.59935.49  166. Baron D. Influence of anemia on outcome in patients undergoing orthotopic liver transplantation. Vox Sang. 2019;114:5. http://doi.org/10.1111/vox.12792  167. Barrio V, Quereda C, Zamora J, García López F. Does continuous veno-venous hemodiafiltration therapy reduce mortality due to acute renal failure as compared to intermittent hemodialysis? Nefrología : Publicación Oficial De La Sociedad Española Nefrologia. 2007;27 Suppl 1:42-8.  168. BA B, A B, AJ B, I A, MT O, M K, et al. Ultrafiltration versus usual care for hospitalized patients with heart failure: the Relief for Acutely Fluid-Overloaded Patients With Decompensated Congestive Heart Failure (RAPID-CHF) trial. J Am Coll Cardiol. 2005(11). http://doi.org/10.1016/j.jacc.2005.05.098  169. O B, S R, D B, N V, B B, M Z, et al. Predictive Value of the Residual SYNTAX Score in Patients With Cardiogenic Shock. J Am Coll Cardiol. 2021(2). http://doi.org/10.1016/j.jacc.2020.11.025  170. M B, B K, S F, K W, V D, JA S, et al. Renal function following conversion from Sandimmune to Neoral in stable renal transplant recipients. Journal of Transplant Coordination. 1997(2). http://doi.org/10.7182/prtr.1.7.2.56032476p80834l2  171. E B, A K, K G, E O, H O, M K, et al. Effects of late acute rejection on graft survival and outcome in pediatric renaltransplant recipients. Nephrol Dial Transpl. 2015. http://doi.org/10.1093/ndt/gfv203.11  172. Baskin E, Gulleroglu KS, Saygili A, Aslamaci S, Varan B, Tokel K. Peritoneal dialysis requirements following open-heart surgery in children with congenital heart disease. Renal Failure. 2010;32(7):784-7. http://doi.org/10.3109/0886022X.2010.493980  173. N B, L R, C M, S K, L E, J G, et al. Prospective monitoring of cytomegalovirus, Epstein-Barr virus, BK virus, and JC virus infections on belatacept therapy after a kidney transplant. Exp Clin Transplant. 2014(3).  174. Bateman RM, Sharpe MD, Jagger JE, Ellis CG, Solé-Violán J, López-Rodríguez M, et al. 36th International Symposium on Intensive Care and Emergency Medicine : Brussels, Belgium. 15-18 March 2016. Crit Care. 2016;20(Suppl 2):94. http://doi.org/10.1186/s13054-016-1208-6  175. CD B, R M, A K, H H, E A. Does Blood Transfusion Have an Effect on Outcomes After Aortic Valve Replacement Surgery? Heart, Lung & Circulation. 2021(6). http://doi.org/10.1016/j.hlc.2020.10.017  176. WO B, L P, L W, JP S, AJ Z. A comparative, randomized trial of concentration-controlled sirolimus combined with reduced-dose tacrolimus or standard-dose tacrolimus in renal allograft recipients. Transpl P. 2013(6). http://doi.org/10.1016/j.transproceed.2013.03.025  177. Beck-Schimmer B, Schadde E, Pietsch U, Filipovic M, Dübendorfer-Dalbert S, Fodor P, et al. Early sevoflurane sedation in severe COVID-19-related lung injury patients. A pilot randomized controlled trial. Ann Intensive Care. 2024;14(1). http://doi.org/10.1186/s13613-024-01276-4  178. N B, A B, W R, MI Q, R N. Caplacizumab as step-up therapy in refractory TTP utilizing adamts-13 activity and evidence of end-organ damage. Blood. 2019. http://doi.org/10.1182/blood-2019-123206  179. Bell S, Rennie T, Marwick CA, Davey P. Effects of peri-operative nonsteroidal anti-inflammatory drugs on post-operative kidney function for adults with normal kidney function. Cochrane Db Syst Rev. 2018;2018(11). http://doi.org/10.1002/14651858.CD011274.pub2  180. Bellizzi V, Signoriello S, Minutolo R, Di Iorio B, Nazzaro P, Conte G, et al. Effect of very low-protein diet versus standard low-protein diet on renal death in patients with chronic kidney disease: A pragmatic, randomized, controlled, multicenter trial. Nephrol Dial Transpl. 2015;30:iii476. http://doi.org/10.1093/ndt/gfv191.12  181. R B. Do we know the optimal dose for renal replacement therapy in the intensive care unit? Kidney Int. 2006(7). http://doi.org/10.1038/sj.ki.5001827  182. R B, H T, N B. Anticoagulant regimens in acute continuous hemodiafiltration: a comparative study. Intens Care Med. 1993(6). http://doi.org/10.1007/BF01694706  183. Bellomo R. Acute renal failure. Semin Resp Crit Care. 2011;32(5):639-50. http://doi.org/10.1055/s-0031-1287872  184. Bellomo R, Cass A, Cole L, Finfer S, Gallagher M, Lee J, et al. Calorie intake and patient outcomes in severe acute kidney injury: Findings from The Randomized Evaluation of Normal vs. Augmented Level of Replacement Therapy (RENAL) study trial. Crit Care. 2014;18(2). http://doi.org/10.1186/cc13767  185. Bellomo R, Cass A, Cole L, Finfer S, Gallagher M, Lee J, et al. Daily protein intake and patient outcomes in severe acute kidney injury: Findings of the randomized evaluation of normal versus augmented level of replacement therapy (RENAL) trial. Blood Purificat. 2014;37(4):325-34. http://doi.org/10.1159/000363175  186. Bellomo R, Lipcsey M, Calzavacca P, Haase M, Haase-Fielitz A, Licari E, et al. Early acid-base and blood pressure effects of continuous renal replacement therapy intensity in patients with metabolic acidosis. Intens Care Med. 2013;39(3):429-36. http://doi.org/10.1007/s00134-012-2800-0  187. Bellomo R, Palevsky PM, Bagshaw SM, Gibney N, McAlister FA, Honore PM, et al. Recent trials in critical care nephrology. Contrib Nephrol. 2010;165:299-309.  188. FJ B, de Maar EF, RR P, van Kan HJ, IJ TB, van der Heide JJ H, et al. Minimization of maintenance immunosuppression early after renal transplantation: an interim analysis. Transplantation. 2009(3). http://doi.org/10.1097/TP.0b013e3181af1df6  189. Ben-Assa E, Arbel Y, Rofe M, Shacham Y, Konigstein M, Havakuk O, et al. Forced diuresis with matched hydration using the renalguard system for the prevention of contrast induced acute kidney injury - A single center experience. JACC: Cardiovascular Interventions. 2014;7(2):S25. http://doi.org/10.1016/j.jcin.2014.01.057  190. U B, U G, S H, A L, D R, D B, et al. Donor Desmopressin Is Associated With Superior Graft Survival After Kidney Transplantation. Transplantation. 2011(11). http://doi.org/10.1097/TP.0b013e318236cd4c  191. Benichou N, Gaudry S, Placier S, Hadchouel J, Hertig A, Chatziantoniou C, et al. Development of a hemodialysis model in rats with septic acute kidney injury. Ann Intensive Care. 2020;10. http://doi.org/10.1186/s13613-020-0623-7  192. Benichou N, Lebbah S, Hajage D, Martin-Lefèvre L, Pons B, Boulet E, et al. Vascular access for renal replacement therapy among 459 critically ill patients: a pragmatic analysis of the randomized AKIKI trial. Ann Intensive Care. 2021;11(1). http://doi.org/10.1186/s13613-021-00843-3  193. A B, M F, P M, B C, B B, A M, et al. Canadian Association of Radiologists: consensus guidelines for the prevention of contrast-induced nephropathy. Journal L'Association Canadienne Des Radiologistes [Canadian Association of Radiologists Journal]. 2007(2).  194. MM B, A S, JP R, E R, MC C, M B, et al. Copper, selenium, zinc, and thiamine balances during continuous venovenous hemodiafiltration in critically ill patients. Am J Clin Nutr. 2004(2). http://doi.org/10.1093/ajcn/80.2.410  195. Berger ED, Bader BD, Bösker J, Risler T, Erley CM. [Contrast media-induced kidney failure cannot be prevented by hemodialysis]. Deut Med Wochenschr. 2001;126(7):162-6. http://doi.org/10.1055/s-2001-11198  196. Berlin N, Pawar R, Grossestreuer AV, Issa MS, Silverman J, Shekhar A, et al. Kidney-specific Biomarkers For Predicting Acute Kidney Injury Following Cardiac Arrest. Circulation. 2022;146. http://doi.org/10.1161/circ.146.suppl_1.125  197. Bermejo S, Bolufer M, Riveiro-Barciela M, Soler MJ. Immunotherapy and the Spectrum of Kidney Disease: Should We Individualize the Treatment? Front Med-Lausanne. 2022;9. http://doi.org/10.3389/fmed.2022.906565  198. T B, J M, P M, C T, S D, P M, et al. Impact of HLA matching on the outcome of simultaneous pancreas-kidney transplantation. Nephrology, Dialysis, Transplantation. 2005. http://doi.org/10.1093/ndt/gfh1082  199. Berni Wennekers A, Dourdil Sahun MV, Bonafonte Arruga E, Asensio Matas A, Martin Azara MP, Alvarez Lipe R, et al. High cut off hemodialysis for renal recovery in patients with multiple myeloma: Five years of experience. Haematologica. 2017;102:515.  200. Bernier-Jean A, Duca A, Madore F, Goupil R, Troyanov S, Bouchard J. Assessment of Methods Used to Substitute for Missing Preadmission Creatinine Values in the Diagnosis and Staging of Acute Kidney Injury. J Am Soc Nephrol. 2015;26:669A.  201. Berthelsen RE, Itenov TS, Perner A, Jensen JU, Ibsen M, Jensen AEK, et al. Forced fluid removal vs. Usual care in intensive care patients with high-risk acute kidney injury and severe fluid overload (FFAKI)-a randomized clinical pilot trial. Intens Care Med Exp. 2016;4. http://doi.org/10.1186/s40635-016-0100-7  202. Berthelsen RE, Itenov T, Perner A, Jensen JU, Ibsen M, Jensen A, et al. Forced fluid removal versus usual care in intensive care patients with high-risk acute kidney injury and severe fluid overload (FFAKI): study protocol for a randomised controlled pilot trial. Trials. 2017;18(1):189. http://doi.org/10.1186/s13063-017-1935-2  203. Besen BAMP, Romano TG, Mendes PV, Gallo CA, Zampieri FG, Nassar AP, et al. Early Versus Late Initiation of Renal Replacement Therapy in Critically Ill Patients: Systematic Review and Meta-Analysis. J Intensive Care Med. 2019;34(9):714-22. http://doi.org/10.1177/0885066617710914  204. AB B, CR F, P H, P S, MJ F, P R, et al. Prospective Randomized Trial Investigating the Influence of Pharmaceutical Care on the Intra-Individual Variability of Tacrolimus Concentrations Early after Kidney Transplant. Ther Drug Monit. 2016(4). http://doi.org/10.1097/FTD.0000000000000299  205. Bhandari S, Jain P. Management of acute coronary syndrome in chronic kidney disease. Journal of Association of Physicians of India. 2012;60(11):48-51.  206. Bhandary S, Ahn J, Shen W. Temporal trends in the inpatient mortality of patients with AKI after coronary revascularization in a nationwide study. J Am Soc Nephrol. 2018;29:117.  207. Bharathan VK, Chandran B, Gopalakrishnan U, Varghese CT, Menon RN, Balakrishnan D, et al. Perioperative prostaglandin e1 infusion in living donor liver transplantation: A double-blind, placebo-controlled randomized trial. Liver Transplant. 2016;22(8):1067-74. http://doi.org/10.1002/lt.24479  208. Bhuta S, Patel N, Burmeister C, Patel M, Ghazaleh S. IMMEDIATE VERSUS DELAYED CORONARY ANGIOGRAPHY FOR OUT-OF-HOSPITAL CARDIAC ARREST WITHOUT ST-SEGMENT ELEVATION: A SYSTEMATIC REVIEW AND META-ANALYSIS. J Am Coll Cardiol. 2022;79(9):934. http://doi.org/10.1016/S0735-1097(22)01925-8  209. Bianchi NA, Altarelli M, Monard C, Kelevina T, Chaouch A, Schneider AG. Identification of an optimal threshold to define oliguria in critically ill patients: an observational study. Crit Care. 2023;27(1):207. http://doi.org/10.1186/s13054-023-04505-7  210. Biancofiore G, Bindi ML, Miccoli M, Cerutti E, Lavezzo B, Pucci L, et al. Intravenous fenoldopam for early acute kidney injury after liver transplantation. J Anesth. 2015;29(3):426-32. http://doi.org/10.1007/s00540-014-1951-2  211. Bierer J, Horne D, Stanzel R, Henderson M, Boulos L, Hayden JA. Continuous Ultrafiltration Enhances Recovery After Adult Cardiac Surgery With Cardiopulmonary Bypass: A Systematic Review and Meta-analysis. CJC Open. 2023;5(7):494-507. http://doi.org/10.1016/j.cjco.2023.03.009  212. Birks P, Al-Zeer B, Holmes D, Elzayat R, Canney M, Djurdjev O, et al. Assessing Discharge Communication and Follow-up of Acute Kidney Injury in British Columbia: A Retrospective Chart Review. Canadian Journal of Kidney Health and Disease. 2024;11. http://doi.org/10.1177/20543581231222064  213. Birnbaum J, Spies CD, Klotz E, Hein OV, Morgera S, Schink T, et al. Iloprost for additional anticoagulation in continuous renal replacement therapy--a pilot study. Renal Failure. 2007;29(3):271-7. http://doi.org/10.1080/08860220601166222  214. Bishara A, Kothari R, Lituiev D, Hannon V, Bokoch M, Niemann C, et al. Predicting acute kidney injury after orthotopic liver transplantation using machine learning. Transplantation. 2019;103(8):75. http://doi.org/10.1097/01.tp.0000580472.17422.db  215. Bitker L, Biscarrat C, Chivot M, Chazot G, Deniel G, Richard JC. Hemodynamic determinants of urine output during continuous renal replacement therapy. Intens Care Med Exp. 2023;11. http://doi.org/10.1186/s40635-023-00546-y  216. Blinder JJ, Alten J, Bailly D, Buckley J, Clarke S, Diddle JW, et al. Diuretic response after neonatal cardiac surgery: a report from the NEPHRON collaborative. Pediatr Nephrol. 2024. http://doi.org/10.1007/s00467-024-06380-y  217. TD B, IW G, PE B. Histological progression of chronic renal allograft injury comparing sirolimus and mycophenolate mofetil-based protocols. A single-center, prospective, randomized, controlled study. Pediatr Transplant. 2010(7). http://doi.org/10.1111/j.1399-3046.2010.01374.x  218. IN B, SS G, EV S, AV S. Nephrological aspects of surgical weight correction in morbid obesity. Terapevt Arkh. 2018(6). http://doi.org/10.26442/terarkh201890698-104  219. Bock KR. Renal replacement therapy in pediatric critical care medicine. Curr Opin Pediatr. 2005;17(3):368-71. http://doi.org/10.1097/01.mop.0000163357.58651.d5  220. J B, MB M, FP A, FV V, FS T. Acute effects of intradialytic aerobic exercise on solute removal, blood gases and oxidative stress in patients with chronic kidney disease. Jornal Brasileiro De Nefrologia. 2017(2). http://doi.org/10.5935/0101-2800.20170022  221. Boldt J, Brosch C, Ducke M, Papsdorf M, Lehmann A. Influence of volume therapy with a modern hydroxyethylstarch preparation on kidney function in cardiac surgery patients with compromised renal function: A comparison with human albumin. Crit Care Med. 2007;35(12):2740-6. http://doi.org/10.1097/01.CCM.0000288101.02556.DE  222. M B, T L, Di Fulvio G, A G, Di Liberato L, R R, et al. Preliminary results of the first phase II clinical trial on the use of an innovative glucose-sparing PD solution (FIRST). Nephrol Dial Transpl. 2020(SUPPL 3). http://doi.org/10.1093/ndt/gfaa142.P1186  223. JM B, MH C, Van Duijnhoven EM, Van Suylen RJ, Van Hooff JP. Early steroid withdrawal in renal transplantation with tacrolimus dual therapy: a pilot study. Transplantation. 2002(12). http://doi.org/10.1097/00007890-200212270-00011  224. Borji R, Reza Khatami M, Reza Abbasi M, Meysamie A, Barkhordari K, Ayatollah Esfahani F, et al. Prophylactic dialysis in non-dialysis-dependent patients with renal failure after CABG. Tehran University Medical Journal. 2014;71(12):800-6.  225. R B, M L, Van Tromp J, T C, M G, N H, et al. Steroid sparing with tacrolimus and mycophenolate mofetil in renal transplantation. Am J Transplant. 2004(11). http://doi.org/10.1111/j.1600-6143.2004.00583.x  226. Borthwick EMJ, Hill CJ, Rabindranath KS, Maxwell AP, Mcauley DF, Blackwood B. High-volume haemofiltration for sepsis in adults. Cochrane Db Syst Rev. 2017;2017(1). http://doi.org/10.1002/14651858.CD008075.pub3  227. Borthwick EMJ, Hill CJ, Rabindranath KS, Maxwell AP, Mcauley DF, Blackwood B. High-volume haemofiltration for sepsis. Cochrane Db Syst Rev. 2013;2013(1). http://doi.org/10.1002/14651858.CD008075.pub2  228. Bouchard J, Weidemann C, Mehta RL. Renal Replacement Therapy in Acute Kidney Injury: Intermittent Versus Continuous? How Much Is Enough? Adv Chronic Kidney D. 2008;15(3):235-47. http://doi.org/10.1053/j.ackd.2008.04.004  229. Bounab R, Heming N, Maxime V, Kuperminc E, Carlos M, Moine P, et al. Continuous renal replacement therapy with oxiris filter in critically ill covid-19 patients. Ann Intensive Care. 2023;13. http://doi.org/10.1186/s13613-023-01131-y  230. Bove T, Monaco F, Covello RD, Zangrillo A. Acute renal failure and cardiac surgery. HSR Proc Intensive Care Cardiovasc Anesth. 2009;1(3):13-21.  231. Bove T, Zangrillo A, Guarracino F, Alvaro G, Persi B, Maglioni E, et al. Effect of fenoldopam on use of renal replacement therapy among patients with acute kidney injury after cardiac surgery: A randomized clinical trial. JAMA - Journal of the American Medical Association. 2014;312(21):2244-53. http://doi.org/10.1001/jama.2014.13573  232. Boyd CJ, Sharp CR, Claus MA, Raisis AL, Hosgood G, Smart L. Prospective randomized controlled blinded clinical trial evaluating biomarkers of acute kidney injury following 6% hydroxyethyl starch 130/0.4 or Hartmann's solution in dogs. J Vet Emerg Crit Car. 2021;31(3):306-14. http://doi.org/10.1111/vec.13056  233. Boyer A, Timsit JF, Klouche K, Canet E, Phan TN, Bohé J, et al. Aminoglycosides in Critically Ill Septic Patients With Acute Kidney Injury Receiving Continuous Renal Replacement Therapy: A Multicenter, Observational Study. Clin Ther. 2021;43(6):1116-24. http://doi.org/10.1016/j.clinthera.2021.04.011  234. Boyle M, Wyndham K, Jacobs S, Torda TA. Comparative clearance performance of two dialyser units used in the CVVHD mode. Aust Crit Care. 1995;8(2):20-5. http://doi.org/10.1016/s1036-7314(95)70258-2  235. Boys C, Saez-Rodriguez J. A FREE OPEN-SOURCE FRAMEWORK TO INFER PATIENT-LEVEL EGFR SLOPES FOR ASSOCIATION WITHMOLECULAR DATA. Nephrol Dial Transpl. 2023;38:i496. http://doi.org/10.1093/ndt/gfad063c_3434  236. Brain MJ, Roodenburg OS, Adams N, McCracken P, Hockings L, Musgrave S, et al. Randomised trial of software algorithm driven regional citrate anticoagulation versus heparin in continuous renal replacement therapy: the Filter Life in Renal Replacement Therapy pilot trial. Critical Care and Resuscitation : Journal of the Australasian Academy of Critical Care Medicine. 2014;16(2):131-7.  237. RA B, HM G, R T, ME R, M P, L Y, et al. De novo donor-specific antibodies in belatacept-treated vs cyclosporine-treated kidney-transplant recipients: post hoc analyses of the randomized phase III BENEFIT and BENEFIT-EXT studies. Am J Transplant. 2018(7). http://doi.org/10.1111/ajt.14721  238. Breilh D, Honore PM, De Bels D, Roberts JA, Gordien JB, Fleureau C, et al. Pharmacokinetics and pharmacodynamics of anti-infective agents during continuous veno-venous hemofiltration in critically ill patients: Lessons learned from an ancillary study of the IVOIRE trial. J Transl Intern Med. 2020;7(4):155-69. http://doi.org/10.2478/jtim-2019-0031  239. P B, M M, H R, B M, R M, P M, et al. Significance of prophylactic urodilatin (INN: ularitide) infusion for the prevention of acute renal failure in patients after heart transplantation. Eur J Med Res. 1995(3).  240. Brenner RM, Chertow GM. The rise and fall of atrial natriuretic peptide for acute renal failure. Curr Opin Nephrol Hy. 1997;6(5):474-6. http://doi.org/10.1097/00041552-199709000-00011  241. Briasoulis A, Pala M, Telila T, Merid O, Akintoye E, Vogiatzi G, et al. Statins and contrast-induced nephropathy: A systematic review and meta-analysis. Curr Pharm Design. 2018;23(46):7141-8. http://doi.org/10.2174/1381612823666170913170527  242. F B, CA H, N L. Myeloma cast nephropathy. Haematologica. 2011.  243. Bridoux F, Carron PL, Pegourie B, Alamartine E, Augeul-Meunier K, Karras A, et al. Effect of High-Cutoff Hemodialysis vs Conventional Hemodialysis on Hemodialysis Independence among Patients with Myeloma Cast Nephropathy: A Randomized Clinical Trial. JAMA - Journal of the American Medical Association. 2017;318(21):2099-110. http://doi.org/10.1001/jama.2017.17924  244. Bridoux F, Leung N, Belmouaz M, Royal V, Ronco P, Nasr SH, et al. Management of acute kidney injury in symptomatic multiple myeloma. Kidney Int. 2021;99(3):570-80. http://doi.org/10.1016/j.kint.2020.11.010  245. RA B, B J, C O, M W, G O, de Zoysa J, et al. A Novel Hypoxia-Inducible Factor-Prolyl Hydroxylase Inhibitor (GSK1278863) for Anemia in CKD: a 28-Day, Phase 2A Randomized Trial. Am J Kidney Dis. 2016(6). http://doi.org/10.1053/j.ajkd.2015.11.021  246. C B, C D, De Micco F, N S, G E, G V, et al. Left Ventricular End-Diastolic Pressure Versus Urine Flow Rate-Guided Hydration in Preventing Contrast-Associated Acute Kidney Injury. JACC. Cardiovascular Interventions. 2020(17). http://doi.org/10.1016/j.jcin.2020.04.051  247. C B, G V, B R, G C. Renal insufficiency following contrast media administration trial II (REMEDIAL II): renalGuard system in high-risk patients for contrast-induced acute kidney injury: rationale and design. Eurointervention. 2011(9). http://doi.org/10.4244/EIJV6I9A194  248. Briguori C, Donnarumma E, Quintavalle C, Fiore D, Condorelli G. Contrast-induced acute kidney injury: Potential new strategies. Curr Opin Nephrol Hy. 2015;24(2):145-53. http://doi.org/10.1097/MNH.0000000000000106  249. Briguori C, Mathew RO, Huang Z, Mavromatis K, Hickson LJ, Lau WL, et al. Dialysis Initiation in Patients With Chronic Coronary Disease and Advanced Chronic Kidney Disease in ISCHEMIA-CKD. J Am Heart Assoc. 2022;11(6). http://doi.org/10.1161/JAHA.121.022003  250. Brinker KR, Dickerman RM, Gonwa TA, Hull AR, Langley JW, Long DL, et al. A randomized trial comparing double-drug and triple-drug therapy in primary cadaveric renal transplants. Transplantation. 1990;50(1):43-9. http://doi.org/10.1097/00007890-199007000-00009  251. CE B, MN C, C C, BN C, D C, LM D, et al. Engagement in a Tailored Pain Coping Skills Training for Patients with Chronic Pain Receiving Maintenance Hemodialysis for Kidney Failure. J Pain. 2022(5). http://doi.org/10.1016/j.jpain.2022.03.127  252. Brivet F, Loirat P. Recent advances in management of acute renal failure. Presse Med. 1996;25(6):227-9.  253. Brivet F, Loirat P. [What are the advances in the treatment of acute kidney failure?]. Presse Med. 1996;25(6):227-9.  254. ME B, M B. Endotoxin and cytokin reduction function of the oXiris filter in a prospective double-blinded cross-over setting in patients with critical Gram-septic shock and continuous renal replacement therapy requiring acute kidney injury. Intens Care Med Exp. 2018(Supplement 2) (no pagination). http://doi.org/10.1186/s40635-018-0201-6  255. Bromberg JS, Weir MR, Gaber AO, Yamin MA, Goldberg ID, Mayne TJ, et al. Renal Function Improvement Following ANG-3777 Treatment in Patients at High Risk for Delayed Graft Function after Kidney Transplantation. Transplantation. 2021;105(2):443-50. http://doi.org/10.1097/TP.0000000000003255  256. Brown SM, Peltan I, Kumar N, Leither L, Webb BJ, Starr N, et al. Hydroxychloroquine versus azithromycin for hospitalized patients with COVID-19: Results of a randomized, active comparator trial. Ann Am Thorac Soc. 2021;18(4):590-7. http://doi.org/10.1513/AnnalsATS.202008-940OC  257. Brunkhorst FM, Engel C, Bloos F, Meier-Hellmann A, Ragaller M, Weiler N, et al. Intensive insulin therapy and pentastarch resuscitation in severe sepsis. New Engl J Med. 2008;358(2):125-39. http://doi.org/10.1056/NEJMoa070716  258. R B, A S, G C, C B, A N, S T, et al. Comparison of heparin to citrate as a catheter locking solution for non-tunneled central venous hemodialysis catheters in patients requiring renal replacement therapy for acute renal failure (VERROU-REA study): study protocol for a randomized controlled trial. Trials. 2014(1). http://doi.org/10.1186/1745-6215-15-449  259. A B, W L, B W. Insufficiency of arteriovenous fistulas in patients with end-stage renal failure--emergency surgical procedures. Przeglad Lekarski. 2003.  260. C B, A M, E C, K K, B C, MW T, et al. Intradialytic Cardiac Magnetic Resonance Imaging to Assess Cardiovascular Responses in a Short-Term Trial of Hemodiafiltration and Hemodialysis. Journal of the American Society of Nephrology : JASN. 2017(4). http://doi.org/10.1681/ASN.2016060686  261. K B, T B, W A, C S, P R, U E, et al. Everolimus-based, calcineurin-inhibitor-free regimen in recipients of de-novo kidney transplants: an open-label, randomised, controlled trial. Lancet (London, England). 2011(9768). http://doi.org/10.1016/S0140-6736(10)62318-5  262. K B, S B, JM G, K C, JE D, HT S, et al. Novel once-daily extended-release tacrolimus (LCPT) versus twice-daily tacrolimus in de novo kidney transplants: one-year results of Phase III, double-blind, randomized trial. Am J Transplant. 2014(12). http://doi.org/10.1111/ajt.12955  263. K B, J C, G K, L C, HH N, Y S, et al. Enteric-coated mycophenolate sodium can be safely administered in maintenance renal transplant patients: results of a 1-year study. Am J Transplant. 2004(2). http://doi.org/10.1046/j.1600-6143.2003.00321.x  264. K B, F L, C S, P R, W A, U E, et al. Five-year outcomes in kidney transplant patients converted from cyclosporine to everolimus: the randomized ZEUS study. Am J Transplant. 2015(1). http://doi.org/10.1111/ajt.12952  265. K B, T R, C S, H H, P R, O W, et al. Renal, efficacy and safety outcomes following late conversion of kidney transplant patients from calcineurin inhibitor therapy to everolimus: the randomized APOLLO study. Clin Nephrol. 2015(1). http://doi.org/10.5414/cn108444  266. K B, C S, T B, A A, F P, JM G, et al. Sotrastaurin, a novel small molecule inhibiting protein kinase C: first clinical results in renal-transplant recipients. Am J Transplant. 2010(3). http://doi.org/10.1111/j.1600-6143.2009.02980.x  267. K B, H T, W A, A S, M Z, M K, et al. Improved rejection prophylaxis with an initially intensified dosing regimen of enteric-coated mycophenolate sodium in de novo renal transplant recipients. Transplantation. 2011(3). http://doi.org/10.1097/TP.0b013e318223d7f3  268. K B, M Z, O W, W A, F L, M G, et al. Everolimus with cyclosporine withdrawal or low-exposure cyclosporine in kidney transplantation from Month 3: a multicentre, randomized trial. Nephrology, Dialysis, Transplantation. 2017(6). http://doi.org/10.1093/ndt/gfx075  269. S B, L R, RR A, P W, J D, S M, et al. LCPT once-daily extended-release tacrolimus tablets versus twice-daily capsules: a pooled analysis of two phase 3 trials in important de novo and stable kidney transplant recipient subgroups. Transpl Int. 2016(5). http://doi.org/10.1111/tri.12770  270. C. Open label multi-center randomized parallel two-group comparison test of the effects of hemoglobin levels on cardiovascular complications, prognosis, and quality of life in chronic hemodialysis patients. Https://Trialsearch.Who.Int/Trial2.Aspx?TrialID=JPRN-C000000386. 2006.  271. Cacho J, Burgos E, Molina M, Villegas A, Pérez M, Cañas L, et al. Remdesivir in kidney transplant patients with SARS-CoV-2 pneumonia. Nefrologia. 2022;42(3):311-7. http://doi.org/10.1016/j.nefro.2021.05.005  272. L C, F Z, B L, L W, Z C, J J. A single-centre, open-label, prospective study of an initially short-term intensified dosing regimen of enteric-coated mycophenolate sodium with reduced cyclosporine A exposure in Chinese live-donor kidney transplant recipients. International Journal of Clinical Practice. Supplement. 2014(181). http://doi.org/10.1111/ijcp.12403  273. Caironi P, Latini R, Struck J, Hartmann O, Bergmann A, Bellato V, et al. Circulating Proenkephalin, Acute Kidney Injury, and Its Improvement in Patients with Severe Sepsis or Shock. Clin Chem. 2018;64(9):1361-9. http://doi.org/10.1373/clinchem.2018.288068  274. D C, G K, M G, M H, J D, G B, et al. Randomized comparison of triple therapy and antithymocyte globulin induction treatment after simultaneous pancreas-kidney transplantation. Kidney Int. 1998(4). http://doi.org/10.1046/j.1523-1755.1998.00094.x  275. D C, Le Mauff B, M H, J D, R B, M D, et al. Prevention of acute rejection episodes with an anti-interleukin 2 receptor monoclonal antibody. I. Results after combined pancreas and kidney transplantation. Transplantation. 1994(2). http://doi.org/10.1097/00007890-199401001-00007  276. D C, L R, N K, D D, Y S, G M, et al. Early corticosteroid avoidance in kidney transplant recipients receiving ATG-F induction: 5-year actual results of a prospective and randomized study. Am J Transplant. 2014(11). http://doi.org/10.1111/ajt.12866  277. D C, L R, N K, Y S, D D, G M, et al. Corticosteroid avoidance in adult kidney transplant recipients under rabbit anti-T-lymphocyte globulin, mycophenolate mofetil and delayed cyclosporine microemulsion introduction. Transpl Int. 2010(3). http://doi.org/10.1111/j.1432-2277.2009.00971.x  278. F C, H L. More patients (pts) with acute renal failure (ARF) requiring hemodialysis (HD) and receiving high dose furosemide (HF) reach early two liter diuresis than pts on placebo (p) and may have shorter HD period. Journal of the American Society of Nephrology : JASN. 2000(Sept).  279. Cantarovich F, Galli C, Benedetti L, Chena C, Castro L, Correa C, et al. High dose frusemide in established acute renal failure. Br Med J. 1973;4(5890):449-50. http://doi.org/10.1136/bmj.4.5890.449  280. Cantarovich F, Rangoonwala B, Lorenz H, Verho M, Esnault VLM. High-dose furosemide for established ARF: A prospective, randomized, double-blind, placebo-controlled, multicenter trial. Am J Kidney Dis. 2004;44(3):402-9. http://doi.org/10.1053/j.ajkd.2004.05.021  281. M C, V G, Z W, M V, G R. Efficacy of everolimus with reduced-exposure cyclosporine in de novo kidney transplant patients at increased risk for efficacy events: analysis of a randomized trial. J Nephrol. 2015(5). http://doi.org/10.1007/s40620-015-0180-6  282. M C, P T, A S, S S, E M, L F, et al. Immediate introduction of everolimus does not affect wound healing and delayed graft function in kidney transplant recipients: 3-months results from NEVERWOUND study. Am J Transplant. 2016. http://doi.org/10.1111/ajt.13897  283. MP C, D M, G H, PJ W, M S, G M, et al. Impact of early parenteral nutrition to complete failing enteral nutrition in adult critically Ill patients: a randomized controlled trial. Endocr Rev. 2011(3).  284. AM C, JM G, C G, E F, S G, E A, et al. Update of our experience in long-term renal function of kidneys transplanted from non-heart-beating cadaver donors. Transpl P. 1993(1 Pt 2).  285. AM C, JM G, I S, D S, E A, S G, et al. Cyclosporin A (CsA) and azathioprine (AZA) combination in renal allografts with CsA nephrotoxicity. Transpl Int. 1992. http://doi.org/10.1007/978-3-642-77423-2_18  286. Castellano G, Intini A, Stasi A, Divella C, Gigante M, Pontrelli P, et al. Complement Modulation of Anti-Aging Factor Klotho in Ischemia/Reperfusion Injury and Delayed Graft Function. Am J Transplant. 2016;16(1):325-33. http://doi.org/10.1111/ajt.13415  287. Castellino S, Santoro D. [Renal replacement therapies in intensive care units: cost analysis]. Giornale Italiano Di Nefrologia : Organo Ufficiale Della Società Italiana Di Nefrologia. 2006;23 Suppl 36:S139-42.  288. A C, J Z, R C, J A. Renal Function Outcomes in Kidney Transplant Recipients After Conversion to Everolimus-Based Immunosuppression Regimen with CNI Reduction or Elimination. Transpl P. 2009(10). http://doi.org/10.1016/j.transproceed.2009.08.065  289. GR C, FW S, M M. Treatment of muscle cramps during maintenance haemodialysis. British Medical Journal. 1973(5876). http://doi.org/10.1136/bmj.3.5876.389  290. J C, S T, R S, M F, J M, D R, et al. Antimicrobial strategy for severe community-acquired Legionnaires' disease: a multicentre retrospective observational study. Ann Intensive Care. 2017(1). http://doi.org/10.1186/s13613-016-0223-8  291. Cerda J, Sheinfeld G, Ronco C. Fluid overload in critically ill patients with acute kidney injury. Blood Purificat. 2010;29(4):331-8. http://doi.org/10.1159/000287776  292. SJ C, JM E, J K, H P, PC L, SK L, et al. A randomized, controlled trial of everolimus-based dual immunosuppression versus standard of care in de novo kidney transplant recipients. Transpl Int. 2014(3). http://doi.org/10.1111/tri.12252  293. K C, F E, B P, L M, E B, A B, et al. Long-term outcomes after severe acute kidney injury in critically ill patients: the SALTO study. Ann Intensive Care. 2023(1). http://doi.org/10.1186/s13613-023-01108-x  294. RC C, ND A, AW M, CP H, KD H. Acute Post-operative Renal Failure and Mortality Risk Following Norwood Surgical Palliation: a Secondary Analysis of the Pediatric Heart Network Single Ventricle Reconstruction Trial Public Use Database. Circulation. 2020(SUPPL 3). http://doi.org/10.1161/circ.142.suppl_3.13533  295. A C, A D, E K, B B, B R. Open prospective study to evaluate cardiovascular risk factors and renal function in 2 dosage regimens of tacrolimus combined with mycophenolate mofetil and steroids in renal transplant patients: 5-year results. Transpl P. 2014(8). http://doi.org/10.1016/j.transproceed.2014.09.045  296. Chan KW, Yu KY, Lee PW, Lai KN, Tang SCW. Global REnal Involvement of CORonavirus Disease 2019 (RECORD): A Systematic Review and Meta-Analysis of Incidence, Risk Factors, and Clinical Outcomes. Front Med-Lausanne. 2021;8. http://doi.org/10.3389/fmed.2021.678200  297. GJ C, HD M, D Q, CE F, NL A, JP R, et al. Experience with the use of sirolimus in liver transplantation--use in patients for whom calcineurin inhibitors are contraindicated. Liver Transplant. 2000(6). http://doi.org/10.1053/jlts.2000.19023  298. Chang YT, Ling TC, Cheng YY, Sun CY, Wu JL, Tai CH, et al. Comparison of immunogenicity and safety between a single dose and one booster trivalent inactivated influenza vaccination in patients with chronic kidney disease: A 20-week, open-label trial. Vaccines-Basel. 2021;9(3):1-15. http://doi.org/10.3390/vaccines9030192  299. Chang Y, Gallon L, Jay CL, Ho B, Shetty K, Levitsky J, et al. Comparative effectiveness analysis of transplant strategies for liver transplant candidates with renal impairment. Hepatology. 2013;58(4):1011A. http://doi.org/10.1002/hep.26876  300. S C, E T, F P, M-V L, M D, LM F, et al. Bendamustine-based (BeEAM) conditioning before autologous stem cell transplantation: result of a french multicenter study of 386 patients from lysa centers. Blood. 2016(22).  301. B C, JO MP, M DCR, L R, J G, Y V, et al. Long-term exposure to belatacept in recipients of extended criteria donor kidneys. Am J Transplant. 2013(11). http://doi.org/10.1111/ajt.12459  302. DM C, M V, WB W, CA W, S K, F Z, et al. Novel Biomarkers Predict Progressive Nephropathy in Patients with Type 2 Diabetes after Acute Coronary Syndrome: the EXAMINE Trial. Journal of the American Society of Nephrology : JASN. 2016.  303. Chatzinikolaou I, Finkel K, Hanna H, Boktour M, Foringer J, Ho T, et al. Antibiotic-coated hemodialysis catheters for the prevention of vascular catheter-related infections: A prospective, randomized study. Am J Med. 2003;115(5):352-7. http://doi.org/10.1016/S0002-9343(03)00367-X  304. A C, M O, MJ E, R E, V D, M B, et al. The clinical impact of humoral immunity in pediatric renal transplantation. Journal of the American Society of Nephrology : JASN. 2013(4). http://doi.org/10.1681/ASN.2012070663  305. Chaudhuri D, Herritt B, Heyland D, Gagnon LP, Thavorn K, Kobewka D, et al. Early Renal Replacement Therapy Versus Standard Care in the ICU: A Systematic Review, Meta-Analysis, and Cost Analysis. J Intensive Care Med. 2019;34(4):323-9. http://doi.org/10.1177/0885066617698635  306. Chen IM, Chen PL, Weng SH, Hsu CP, Shih CC, Chang HH, et al. Clinical Outcomes of VasoRing Connector in Patients With Acute Type A Aortic Dissection. Ann Thorac Surg. 2018;106(3):764-70. http://doi.org/10.1016/j.athoracsur.2018.03.056  307. Chen LX, Demirjian S, Udani SM, Trevino SA, Koyner JL. Cytokine Clearance in Continuous Venovenous Hemofiltration and Continuous Venovenous Hemodialysis. J Am Soc Nephrol. 2016;27:914A.  308. Chen LX, Demirjian S, Udani SM, Trevino SA, Murray PT, Koyner JL. Cytokine Clearances in Critically Ill Patients on Continuous Renal Replacement Therapy. Blood Purificat. 2018;46(4):315-22. http://doi.org/10.1159/000492025  309. Chen L, Gong X. Efficacy and Safety of Chuan Huang Fang Combining Reduced Glutathione in Treating Acute Kidney Injury (Grades 1-2) on Chronic Kidney Disease (Stages 2-4): Study Protocol for a Multicenter Randomized Controlled Clinical Trial. Evid-Based Compl Alt. 2022;2022. http://doi.org/10.1155/2022/1099642  310. Chen SL, Zhang J, Yei F, Zhu Z, Liu Z, Lin S, et al. Clinical outcomes of contrast-induced nephropathy in patients undergoing percutaneous coronary intervention: a prospective, multicenter, randomized study to analyze the effect of hydration and acetylcysteine. Int J Cardiol. 2008;126(3):407-13. http://doi.org/10.1016/j.ijcard.2007.05.004  311. Chen X, Ma T. Sustained low-efficiency daily diafiltration for diabetic nephropathy patients with acute kidney injury. Med Prin Pract. 2014;23(2):119-24. http://doi.org/10.1159/000358172  312. Chen X, Xu J, Li Y, Xu X, Shen B, Zou Z, et al. Risk scoring systems including electrolyte disorders for predicting the incidence of acute kidney injury in hospitalized patients. Clin Epidemiol. 2021;13:383-96. http://doi.org/10.2147/CLEP.S311364  313. Chen YY, Liu CF, Shen YT, Kuo YT, Ko CC, Chen TY, et al. Development of real-time individualized risk prediction models for contrast associated acute kidney injury and 30-day dialysis after contrast enhanced computed tomography. Eur J Radiol. 2023;167. http://doi.org/10.1016/j.ejrad.2023.111034  314. Cheng X, Hu Q, Zhao H, Qin S, Zhang D. Transcatheter Versus Surgical Aortic Valve Replacement in Patients With Chronic Kidney Disease: A Meta-Analysis. J Cardiothor Vasc an. 2019;33(8):2221-30. http://doi.org/10.1053/j.jvca.2018.12.010  315. Cherney DZI, Charbonnel B, Cosentino F, Dagogo-Jack S, McGuire DK, Pratley R, et al. Effects of ertugliflozin on kidney composite outcomes, renal function and albuminuria in patients with type 2 diabetes mellitus: an analysis from the randomised VERTIS CV trial. Diabetologia. 2021;64(6):1256-67. http://doi.org/10.1007/s00125-021-05407-5  316. GM C, GA B, R C, TB D, J F, WG G, et al. Effect of cinacalcet on cardiovascular disease in patients undergoing dialysis. New Engl J Med. 2012(26). http://doi.org/10.1056/NEJMoa1205624  317. Chertow GM, Lazarus JM, Paganini EP, Allgren RL, Lafayette RA, Sayegh MH. Predictors of mortality and the provision of dialysis in patients with acute tubular necrosis. The Auriculin Anaritide Acute Renal Failure Study Group. J Am Soc Nephrol. 1998;9(4):692-8. http://doi.org/10.1681/ASN.V94692  318. Cheungpasitporn W, Thongprayoon C, Mitema DG, Mao MA, Sakhuja A, Kittanamongkolchai W, et al. The effect of aspirin on kidney allograft outcomes; a short review to current studies. Journal of Nephropathology. 2017;6(3):110-7. http://doi.org/10.15171/jnp.2017.19  319. ChiCTR. The efficacy and safety of argatroban for venous catheter lock solution: prospective, open-label, randomized controlled trial. Https://Trialsearch.Who.Int/Trial2.Aspx?TrialID=ChiCTR1800017105. 2018.  320. ChiCTR. The efficacy and safety of ischemia-free liver transplantation versus conventional liver transplantation in the treatment of end-stage liver diseases: a single-centre randomised controlled trial. Https://Trialsearch.Who.Int/Trial2.Aspx?TrialID=ChiCTR1900021158. 2019.  321. ChiCTR. A prospective, randomized, controlled, double-blind clinical trial for intravenous high-dose vitamin C on acute pancreatits. Https://Trialsearch.Who.Int/Trial2.Aspx?TrialID=ChiCTR1900022022. 2019.  322. ChiCTR. Clinical study for the effect of Continuous Renal Replacement Therapy on filter life in different models. Http://Www.Who.Int/Trialsearch/Trial2.Aspx?TrialID=ChiCTR2000029873. 2020.  323. ChiCTR. Effect of Recombinant Human Brain Natriuretic Peptide on Preventing Acute Kidney Injury (AKI) in high risk patients: a pilot randomized controlled trial. Https://Trialsearch.Who.Int/Trial2.Aspx?TrialID=ChiCTR2000035248. 2020.  324. ChiCTR. The value of biomarker combined with RRI guided CRRT in SA-AKI. Http://Www.Who.Int/Trialsearch/Trial2.Aspx?TrialID=ChiCTR2000039443. 2020.  325. ChiCTR. Preliminary study of caspofungin sequential posaconazole in prevention of invasive fungal infection in high-risk liver transplant recipients. Https://Trialsearch.Who.Int/Trial2.Aspx?TrialID=ChiCTR2100047646. 2021.  326. ChiCTR. Clinical study on early intervention of cluster optimization therapy based on inflammatory response theory in patients with sepsis acute renal injury. Https://Trialsearch.Who.Int/Trial2.Aspx?TrialID=ChiCTR2100053299. 2021.  327. ChiCTR. Methylprednisolone for acute type A aortic dissection patients ongoing total arch replacement trial(MEDAL trial). Https://Trialsearch.Who.Int/Trial2.Aspx?TrialID=ChiCTR2200059286. 2022.  328. ChiCTR. Efficacy of regional citrate anticoagulation in different models of continuous renal replacement therapy: a prospective, open, single-center randomized controlled trial. Https://Trialsearch.Who.Int/Trial2.Aspx?TrialID=ChiCTR2200061065. 2022.  329. ChiCTR. Effect of ultrasound-guided quadratus lumbar block on early rehabilitation of renal allograft patients. Https://Trialsearch.Who.Int/Trial2.Aspx?TrialID=ChiCTR2200062944. 2022.  330. ChiCTR. Efficacy and safety of anisodamine hydrobromide injection in sepsis: a prospective, open, randomized, controlled, multicenter clinical study. Https://Trialsearch.Who.Int/Trial2.Aspx?TrialID=ChiCTR2200063969. 2022.  331. ChiCTR. A prospective, multicenter, randomized, open-label, parallel-positive controlled non-inferiority clinical trial evaluating the safety and efficacy of hollow fibrous hemodialysis filters for hemodialysis therapy in patients with chronic renal failure. Https://Trialsearch.Who.Int/Trial2.Aspx?TrialID=ChiCTR2300075002. 2023.  332. ChiCTR. Optimization of blood transfusion strategies for ICU patients with acute kidney injury undergoing continuous renal replacement therapy. Https://Trialsearch.Who.Int/Trial2.Aspx?TrialID=ChiCTR2300077076. 2023.  333. ChiCTR. Effect of early intravenous use of the ß-blocker esmolol on the prognosis of acute pancreatitis patients to control heart rate. Https://Trialsearch.Who.Int/Trial2.Aspx?TrialID=ChiCTR2400080160. 2024.  334. ChiCTR-IOR-. Effect of different renal replacement therapy modalities in acute kidney injury following multiple wasp sting. Https://Trialsearch.Who.Int/Trial2.Aspx?TrialID=ChiCTR-IOR-15006166. 2014.  335. ChiCTR-IPR-. Therapeutic strategy of aortic arch for acute type A aortic dissection. Https://Trialsearch.Who.Int/Trial2.Aspx?TrialID=ChiCTR-IPR-16009372. 2016.  336. ChiCTR-IPR-. Effects of metformin on contrast-induced nephropathy in diabetes mellitus after cardiac catheterization procedures. Https://Trialsearch.Who.Int/Trial2.Aspx?TrialID=ChiCTR-IPR-17012793. 2017.  337. ChiCTR-IPR-. Effects of oral nicorandil on contrast-induced nephropathy in coronary artery disease with renal insufficiency after percutaneous coronary intervention. Https://Trialsearch.Who.Int/Trial2.Aspx?TrialID=ChiCTR-IPR-17012805. 2017.  338. ChiCTR-TRC-. Regional Citrate Anticoagulation Using a Calcium-containing Replacement Solution for Continuous Venovenous Hemofiltration. Https://Trialsearch.Who.Int/Trial2.Aspx?TrialID=ChiCTR-TRC-10001042. 2010.  339. Chiles JW, Colon CM, Barrios JG, Maddox WR, Mcdonald MN, Kim Y, et al. An Alabama Cohort of Early 2020 Critically Ill Covid Patients Managed with Supportive Care and Their 90-Day Outcomes. Am J Resp Crit Care. 2022;205(1). http://doi.org/10.1164/ajrccm-conference.2022.205.1_MeetingAbstracts.A2974  340. Chitalia VC, Fernandes Almeida A, Rai H, Bapat M, Chitalia KV, Acharya VN, et al. Is peritoneal dialysis adequate for hypercatabolic acute renal failure in developing countries? Kidney Int. 2002;61(2):747-57. http://doi.org/10.1046/j.1523-1755.2002.00177.x  341. Choi JS, Yang WS, Kim SB, Park SK, Lee SK, Park JS, et al. In spite of positive charge on polyethyleneimine, an69 st membrane does not tightly adsorb heparin during continuous renal replacement therapy. Int J Artif Organs. 2011;34(8):626-7. http://doi.org/10.5301/IJAO.2011.8701  342. Choi MJ, Yoon JW, Han SJ, Choi HH, Song YR, Kim SG, et al. The prevention of contrast-induced nephropathy by simultaneous hemofiltration during coronary angiographic procedures: a comparison with periprocedural hemofiltration. Int J Cardiol. 2014;176(3):941-5. http://doi.org/10.1016/j.ijcard.2014.08.095  343. E CV, J ER, A CS, E MM, A VG, D RE, et al. Comparative Study of 2 Extended-Release Tacrolimus Formulations in Kidney Transplantation. Transpl P. 2022(9). http://doi.org/10.1016/j.transproceed.2022.10.001  344. Chow WL, Tong SC, Aung KCY. Sustained low efficiency dialysis (SLED): A rapid review. Int J Technol Assess. 2017;33:218-9. http://doi.org/10.1017/S0266462317003932  345. T C, P P. Efficacy of 5%, 10% trisodium citrate and heparin as catheter-locking solution for central venous hemodialysis catheters: a prospective randomized controlled study. Chotmaihet Thangphaet [Journal of the Medical Association of Thailand]. 2020(11). http://doi.org/10.35755/jmedassocthai.2020.11.10190  346. Chung KK, Lundy JB, Matson JJ, Renz EM, White CE, King BT, et al. Continuous venovenous hemofiltration in severely burned patients with acute kidney injury: A cohort study. Crit Care. 2009;13(3). http://doi.org/10.1186/cc7801  347. G C, JJ G, G G, D R, L C, W K, et al. Randomized trial of 3 maintenance regimens (TAC/SRL vs. TAC/MMF vs. CSA/SRL) with low-dose corticosteroids in primary kidney transplantation: 18-year results. Clin Transplant. 2020(12). http://doi.org/10.1111/ctr.14123  348. G C, JJ G, G G, J S, L C, A M, et al. Randomized trial of three induction antibodies in kidney transplantation: long-term results. Transplantation. 2014(11). http://doi.org/10.1097/01.TP.0000441089.39840.66  349. G C, JJ G, G G, J S, D R, L C, et al. Antibody-mediated rejection implies a poor prognosis in kidney transplantation: results from a single center. Clin Transplant. 2018(10). http://doi.org/10.1111/ctr.13392  350. G C, JJ G, D R, W K, L H, L T, et al. Randomized trial of thymoglobulin versus alemtuzumab (with lower dose maintenance immunosuppression) versus daclizumab in living donor renal transplantation. Transpl P. 2010(9). http://doi.org/10.1016/j.transproceed.2010.08.045  351. G C, J S, E A, JJ G, L C, A Z, et al. A randomized pilot study of donor stem cell infusion in living-related kidney transplant recipients receiving alemtuzumab. Transplantation. 2013(9). http://doi.org/10.1097/TP.0b013e3182a0f68c  352. G C, P T, JJ G, G G, J S, D R, et al. Pilot Randomized Trial of Tacrolimus/Everolimus vs Tacrolimus/Enteric-Coated Mycophenolate Sodium in Adult, Primary Kidney Transplant Recipients at a Single Center. Transpl P. 2016(6). http://doi.org/10.1016/j.transproceed.2016.03.048  353. D C, HT S, A V, E L, C C, RG W, et al. Randomized trial of everolimus-facilitated calcineurin inhibitor minimization over 24 months in renal transplantation. Transplantation. 2013(7). http://doi.org/10.1097/TP.0b013e3182848e03  354. Cillo U, Saracino L, Vitale A, Bertacco A, Salizzoni M, Lupo F, et al. Very Early Introduction of Everolimus in De Novo Liver Transplantation: Results of a Multicenter, Prospective, Randomized Trial. Liver Transplant. 2019;25(2):242-51. http://doi.org/10.1002/lt.25400  355. WR C, BA M, KJ A, WL M. A comparison of metabolic control by continuous and intermittent therapies in acute renal failure. Journal of the American Society of Nephrology : JASN. 1994(7). http://doi.org/10.1681/ASN.V471413  356. Clark EG, McIntyre L, Ramsay T, Tinmouth A, Knoll G, Brown PA, et al. Saline versus albumin fluid for extracorporeal removal with slow lowefficiency dialysis (SAFER-SLED): Study protocol for a pilot trial. Pilot Feasibility St. 2019;5(1). http://doi.org/10.1186/s40814-019-0460-3  357. Clark WR, Letteri JJ, Uchino S, Bellomo R, Ronco C. Recent clinical advances in the management of critically ill patients with acute renal failure. Blood Purificat. 2006;24(5-6):487-98. http://doi.org/10.1159/000095929  358. PA C, SP M, JR C, SJ C. Mycophenolate versus azathioprine for kidney transplantation: a 15-year follow-up of a randomized trial. Transplantation. 2012(2). http://doi.org/10.1097/TP.0b013e31825475a3  359. Clec'H C, Darmon M, Lautrette A, Chemouni F, Azoulay E, Schwebel C, et al. Efficacy of renal replacement therapy in critically ill patients: A propensity analysis. Crit Care. 2012;16(6). http://doi.org/10.1186/cc11905  360. Clerico A, Galli C, Fortunato A, Ronco C. Neutrophil gelatinase-associated lipocalin (NGAL) as biomarker of acute kidney injury: A review of the laboratory characteristics and clinical evidences. Clin Chem Lab Med. 2012;50(9):1505-17. http://doi.org/10.1515/cclm-2011-0814  361. SA C, CE R, CM M, J S, AC S, L A. Hemodiafiltration decreases serum levels of inflammatory mediators in severe leptospirosis: a prospective study. Plos One. 2016(8). http://doi.org/10.1371/journal.pone.0160010  362. Cleto S, Malaque C, Rodrigues C, Sztajnbok J, Seguro A, Andrade L. Impact of different methods of renal replacement therapy in weil syndrome. Crit Care Med. 2015;43(12):246. http://doi.org/10.1097/01.ccm.0000474806.99688.e5  363. M C, T H, C R, L C, J T, G A, et al. The Effects of Preoperative Volume Replacement in Diabetic Patients Undergoing Coronary Artery Bypass Grafting Surgery: Protocol for a Randomized Controlled Trial (VeRDiCT Trial). Jmir Res Protoc. 2017(6). http://doi.org/10.2196/resprot.7386  364. Coates EC, Mann-Salinas EA, Caldwell NW, Chung KK. Challenges Associated with Managing a Multicenter Clinical Trial in Severe Burns. Journal of Burn Care and Research. 2020;41(3):681-9. http://doi.org/10.1093/JBCR/IRAA014  365. Cockwell P, Hutchison CA. Management options for cast nephropathy in multiple myeloma. Curr Opin Nephrol Hy. 2010;19(6):550-5. http://doi.org/10.1097/MNH.0b013e32833ef72c  366. AA C, R V, A N, S U, M H, G L, et al. Fenoldopam infusion for renal protection in high-risk cardiac surgery patients: a randomized clinical study. J Cardiothor Vasc an. 2007(6). http://doi.org/10.1053/j.jvca.2007.02.022  367. JB C, TC H, V C, P W, N R, NR R, et al. Randomized elimination and prolongation of ACE inhibitors and ARBs in coronavirus 2019 (REPLACE COVID) Trial Protocol. Journal of Clinical Hypertension (Greenwich, Conn.). 2020(10). http://doi.org/10.1111/jch.14011  368. Cole L, Bellomo R, Hart G, Journois D, Davenport P, Tipping P, et al. A phase II randomized, controlled trial of continuous hemofiltration in sepsis. Crit Care Med. 2002;30(1):100-6. http://doi.org/10.1097/00003246-200201000-00016  369. Collins MG, Fahim MA, Pascoe EM, Dansie KB, Hawley CM, Clayton PA, et al. Study Protocol for Better Evidence for Selecting Transplant Fluids (BEST-Fluids): A pragmatic, registry-based, multi-center, double-blind, randomized controlled trial evaluating the effect of intravenous fluid therapy with Plasma-Lyte 148 versus 0.9% saline on delayed graft function in deceased donor kidney transplantation. Trials. 2020;21(1). http://doi.org/10.1186/s13063-020-04359-2  370. Cornell TT, Selewski DT, Alten JA, Askenazi D, Fitzgerald JC, Topjian A, et al. Acute kidney injury after out of hospital pediatric cardiac arrest. Resuscitation. 2018;131:63-8. http://doi.org/10.1016/j.resuscitation.2018.07.362  371. Corrêa TD, Cavalcanti AB, Assunção MS. Balanced crystalloids for septic shock resuscitation. Rev Bras Ter Intensiva. 2016;28(4):463-71. http://doi.org/10.5935/0103-507X.20160079  372. Correction Bellomo R, Cass A, Cole L, Finfer S, Gallagher M, Lee J, et al. An observational study fluid balance and patient outcomes in the randomized evaluation of normal vs. augmented level of replacement therapy trial. Crit Care Med. 2012;40(6):1753-60. http://doi.org/10.1097/CCM.0b013e318246b9c6  373. Corteville D, Still RJ, Szabo G, Swaminathan M, Jayasankar V, Lamy A, et al. Efficacy and safety of QPI-1002 (QPI) for prevention of AKI following cardiac surgery. J Am Soc Nephrol. 2017;28:B1.  374. MR C, ME G, MT S, ML J, BA B, JR T, et al. Ultrafiltration versus intravenous diuretics for patients hospitalized for acute decompensated heart failure. J Am Coll Cardiol. 2007(6). http://doi.org/10.1016/j.jacc.2006.07.073  375. F C, De La Torre-Prados M-V, E C, P N, T T, A F, et al. Hemofiltration veno-venous continouos high and very high volume, pulmonary (paO2/FiO2) function and mortality in refractory septic shock patients. Intens Care Med Exp. 2017(2). http://doi.org/10.1186/s40635-017-0151-4  376. Côté JM, Pinard L, Cailhier JF, Lévesque R, Murray PT, Beaubien-Souligny W. Intermittent Convective Therapies in Patients with Acute Kidney Injury: A Systematic Review with Meta-Analysis. Blood Purificat. 2022;51(1):75-86. http://doi.org/10.1159/000515641  377. Coupes B, de Freitas DG, Roberts SA, Read I, Riad H, Brenchley PE, et al. rhErythropoietin-b as a tissue protective agent in kidney transplantation: a pilot randomized controlled trial. Bmc Res Notes. 2015;8:21. http://doi.org/10.1186/s13104-014-0964-0  378. M C, C A, S C, D P, JC S, JM T, et al. VITamin D supplementation in renAL transplant recipients (VITALE): a prospective, multicentre, double-blind, randomized trial of vitamin D estimating the benefit and safety of vitamin D3 treatment at a dose of 100,000 UI compared with a dose of 12,000 UI in renal transplant recipients: study protocol for a double-blind, randomized, controlled trial. Trials. 2014. http://doi.org/10.1186/1745-6215-15-430  379. J C, A S, D A, EV N, AC W. Antibiotics for asymptomatic bacteriuria in kidney transplant recipients. Cochrane Db Syst Rev. 2018(2). http://doi.org/10.1002/14651858.CD011357.pub2  380. Coutinho B, Chirita-Parker O, Okumura L, Riveros BS, Nita ME. PCV110 COST-EFFECTIVENESS OF SUTURELESS AORTIC VALVE REPLACEMENT VERSUS CONVENTIONAL AORTIC VALVE REPLACEMENT AND TRANSCATHETER AORTIC VALVE IMPLANTATION IN BRAZIL. Value Health. 2019;22:S562. http://doi.org/10.1016/j.jval.2019.09.834  381. Coville H, Wald R, Bagshaw S, Weist S, Dong Y, Banaei-Kashani K. Clinical decision patterns on renal replacement therapy among severe acute kidney injury. Crit Care Med. 2018;46:672. http://doi.org/10.1097/01.ccm.0000529380.86608.df  382. ZL C, HK S, LW S, B B, JH H, K H, et al. Randomized controlled trial of urinE chemiStry guided aCute heArt faiLure treATmEnt (ESCALATE): rationale and design. Am Heart J. 2023. http://doi.org/10.1016/j.ahj.2023.07.014  383. TR C, MJ D, M S, CM O, JS E, DF M. Results of the HARP study: a randomized double blind phase II trial of 80mg simvastatin in acute lung injury. Am J Resp Crit Care. 2010(1).  384. K C, M C, M L, Van Hoeck K, JC D, J N. Maintenance immunosuppression with mycophenolate mofetil and corticosteroids in pediatric kidney transplantation: temporary benefit but not without risk. Transplantation. 2007(8). http://doi.org/10.1097/01.tp.0000260146.57898.9c  385. Crosnier J, Kreis H, Descamps JM, Mansouri R. Are there non-steroid-dependent rejection episodes? Proc Eur Dial Transplant Assoc. 1980;17:391-5.  386. SA C, CM P. Tacrolimus once-daily formulation: in the prophylaxis of transplant rejection in renal or liver allograft recipients. Drugs. 2007(13). http://doi.org/10.2165/00003495-200767130-00012  387. ST C, GM C, J V, T O, J Z, RM S, et al. Lessons for successful study enrollment from the Veterans Affairs/National Institutes of Health Acute Renal Failure Trial Network Study. Clinical Journal of the American Society of Nephrology: CJASN. 2008(4).  388. CTIS. THINK - intensification of blood pressure lowering Therapeutics based on diuretics versus usual management for uncontrolled Hypertension IN patients with moderate to severe chronic Kidney disease: an open lapel, cluster randomized controlled, phase 3 trial. Https://Trialsearch.Who.Int/Trial2.Aspx?TrialID=CTIS2022-501494-39-00. 2022.  389. CTIS. Multicentre, open-label, randomised, two-arm, parallel-group, superiority trial to assess bioavailability and practicability of two once-daily tacrolimus formulations, Envarsus® compared with Advagraf™, administered in kidney transplant recipients. Https://Trialsearch.Who.Int/Trial2.Aspx?TrialID=CTIS2023-503531-18-00. 2023.  390. CTRI. Effect of giving lesser fluids during operation on the kidney functions in patients undergoing living donor renal transplant surgery. Https://Trialsearch.Who.Int/Trial2.Aspx?TrialID=CTRI/2013/12/004251. 2013.  391. CTRI. The outcome of liver infection by hepatitis C virus in kidney failure patients who undergo kidney transplantation. Https://Trialsearch.Who.Int/Trial2.Aspx?TrialID=CTRI/2015/02/005513. 2015.  392. CTRI. Effect of drug furosemide on progression of early onset of acute kidney failure in sick children- A comparative controlled study. Https://Trialsearch.Who.Int/Trial2.Aspx?TrialID=CTRI/2016/09/007321. 2016.  393. CTRI. A clinical trial comparing two different modes of blood purification in patients with recent onset kidney shut down due to some illness. Https://Trialsearch.Who.Int/Trial2.Aspx?TrialID=CTRI/2017/09/009675. 2017.  394. CTRI. Prevention of contrast induced acute kidney injury by Remote ischemic preconditioning. Https://Trialsearch.Who.Int/Trial2.Aspx?TrialID=CTRI/2017/12/011049. 2017.  395. CTRI. Role of Vitamin C hydrocortisone and thiamine in critically sick patients (sepsis and septic shock). Https://Trialsearch.Who.Int/Trial2.Aspx?TrialID=CTRI/2018/08/015193. 2018.  396. CTRI. Treatment of COVID19 : a randomised controlled trial. Https://Trialsearch.Who.Int/Trial2.Aspx?TrialID=CTRI/2020/04/024904. 2020.  397. CTRI. Remote Ischemic Conditioning For Prevention Of Contrast Induced Kidney Injury. Https://Trialsearch.Who.Int/Trial2.Aspx?TrialID=CTRI/2020/07/026548. 2020.  398. CTRI. Fluid management in diabetes ketoacidosis in children. Https://Trialsearch.Who.Int/Trial2.Aspx?TrialID=CTRI/2020/12/029450. 2020.  399. CTRI. Clinical study to assess the effect and safety of study product (Thymosin Alpha-1 and Ulinastatin) in sepsis patients. Https://Trialsearch.Who.Int/Trial2.Aspx?TrialID=CTRI/2021/04/032842. 2021.  400. CTRI. Ultrasound guided fluid removal in dialysis. Https://Trialsearch.Who.Int/Trial2.Aspx?TrialID=CTRI/2021/11/037766. 2021.  401. CTRI. Investigator Initiated Study to evaluate the safety and efficacy of Thymosin alpha-1 (T alpha-1). Https://Trialsearch.Who.Int/Trial2.Aspx?TrialID=CTRI/2021/12/038693. 2021.  402. CTRI. A study to determine if a new therapy(Plasma Exchange) can help in recovery of Acute on chronic Liver failure. Https://Trialsearch.Who.Int/Trial2.Aspx?TrialID=CTRI/2022/01/039094. 2022.  403. CTRI. Anti-fungal Strategies in Acute-on-Chronic Liver Failure Patients. Https://Trialsearch.Who.Int/Trial2.Aspx?TrialID=CTRI/2022/01/039674. 2022.  404. CTRI. Effect of lipid infusion during hemodialysis on renal recovery in patients with severe renal injury. Https://Trialsearch.Who.Int/Trial2.Aspx?TrialID=CTRI/2023/08/055983. 2023.  405. CTRI. Comparison of influence of three types of fluids on kidney function of children admitted with low blood pressures. Https://Trialsearch.Who.Int/Trial2.Aspx?TrialID=CTRI/2023/09/057723. 2023.  406. CTRI. Dialysis in acute kidney injury. Https://Trialsearch.Who.Int/Trial2.Aspx?TrialID=CTRI/2023/09/057935. 2023.  407. CTRI. Comparison of duration of hospital stay between patients receiving care under standard care protocol and Enhanced recovery after surgery protocol. Https://Trialsearch.Who.Int/Trial2.Aspx?TrialID=CTRI/2023/12/060932. 2023.  408. J C, Y K. Comparative analysis of nine COVID-19 convalescent plasma protocols registered by cochrane central register of controlled trials. Iran Red Crescent Me. 2020(8). http://doi.org/10.32592/ircmj.2020.22.8.47  409. Cui JW, Xu Y, Wang Y, Gao YX, Guo S, Wang M, et al. Efficacy of initial haemopurification strategy for acute paraquat poisoning in adults: Study protocol for a randomised controlled trial (HeSAPP). Bmj Open. 2018;8(6). http://doi.org/10.1136/bmjopen-2018-021964  410. Cui X, Zou Y, Gao W, Liu H, Wang S, Wei W, et al. Volume management of intermittent hemofiltration guided by critical care ultrasound in the treatment of acute kidney injury. Chinese Critical Care Medicine. 2023;35(3):310-5. http://doi.org/10.3760/cma.j.cn121430-20220809-00733  411. Curry MP, Vargas HE, Befeler AS, Pyrsopoulos NT, Patwardhan VR, Jamil K. Early treatment with terlipressin in patients with hepatorenal syndrome yields improved clinical outcomes in North American studies. Hepatol Commun. 2023;7(1). http://doi.org/10.1097/01.HC9.0000897228.91307.0c  412. Curry M, Vargas HE, Befeler A, Pyrsopoulos NT, Patwardhan V, Jamil K. Early treatment with terlipressin in patients with hepatorenal syndrome yields improved clinical outcomes in 3 phase III North American studies. J Hepatol. 2022;77:S634-5. http://doi.org/10.1016/S0168-8278(22)01585-9  413. A C, N K, B M, S N, M Z, D G, et al. The impact of different infusion solutions on postoperative recovery following colorectal surgery. Journal of B.U.ON. 2018(5).  414. D Alessandro S, Tuttolomondo D, Singh G, Hernandez-Vaquero D, Pattuzzi C, Gallingani A, et al. The early and long-term outcomes of coronary artery bypass grafting added to aortic valve replacement compared to isolated aortic valve replacement in elderly patients: a systematic review and meta-analysis. Heart Vessels. 2022;37(10):1647-61. http://doi.org/10.1007/s00380-022-02073-4  415. M D, A H, J C, C T, S M, Y P. Pharmacokinetics of serelaxin in patients with severe renal impairment or end-stage renal disease requiring hemodialysis: a single-dose, open-label, parallel-group study. J Clin Pharmacol. 2016(4). http://doi.org/10.1002/jcph.607  416. WB D, LK C, SY Q, ZY P, X Z, LL H, et al. Lactated Ringer's solution versus normal saline in pediatric living-donor liver transplantation: a matched retrospective cohort study. Paediatric Anaesthesia. 2021(6). http://doi.org/10.1111/pan.14181  417. Dai T, Cao S, Yang X. Comparison of clinical efficacy between continuous renal replacement therapy and intermittent haemodialysis for the treatment of sepsis-induced acute kidney injury. Chinese Critical Care Medicine. 2016;28(3):277-80. http://doi.org/10.3760/cma.j.issn.2095-4352.2016.03.017  418. A D, N V, A K, H S, S K, PE S. Comparing CVVH with CVVHD during citrate anticoagulation in ICU patients. Neth J Crit Care. 2017(4).  419. Damien R, Nicolas B, David H, Laurent ML, Nicolas DP, Nicolas L, et al. Impact of renal replacement therapy strategies on beta-lactamine plasma concentrations: The BETAKIKI study: An ancillary study of a randomized controlled trial. Ann Intensive Care. 2018;8(1). http://doi.org/10.1186/s13613-017-0345-7  420. J D, F B, MC M, L R, C L, R G, et al. Efficacy and safety of de novo or early everolimus with low cyclosporine in deceased-donor kidney transplant recipients at specified risk of delayed graft function: 12-month results of a randomized, multicenter trial. Transpl Int. 2010(11). http://doi.org/10.1111/j.1432-2277.2010.01094.x  421. B D, M S, G K, NI E, AA A, AA E. Fluid balance as an early indicator of acute kidney injury in CV surgery. Clin Nephrol. 2012(6). http://doi.org/10.5414/cn107278  422. Dass B, Beaver TM, Shimada M, Alquadan KF, Koratala A, Singhania G, et al. Natriuretic peptides in acute kidney injury – A sojourn on parallel tracks? Eur J Intern Med. 2020;71:39-44. http://doi.org/10.1016/j.ejim.2019.11.025  423. Datta D, Foley RJ, Wu R, Grady J, Scalise P. Renal Function, Weaning, and Survival in Patients With Ventilator-Dependent Respiratory Failure. J Intensive Care Med. 2019;34(3):212-7. http://doi.org/10.1177/0885066617696849  424. Datzmann T, Hoenicka M, Reinelt H, Liebold A, Gorki H. Influence of 6% Hydroxyethyl Starch 130/0.4 Versus Crystalloid Solution on Structural Renal Damage Markers After Coronary Artery Bypass Grafting: A Post Hoc Subgroup Analysis of a Prospective Trial. J Cardiothor Vasc an. 2018;32(1):205-11. http://doi.org/10.1053/j.jvca.2017.05.041  425. KM D, GB L, R V. Acute dialytic support for the critically ill: continuous venovenous haemodialysis versus continuous venovenous haemofiltration. International Medical Journal. 2006(1).  426. A D, EJ W, AM D. Continuous vs. intermittent forms of haemofiltration and/or dialysis in the management of acute renal failure in patients with defective cerebral autoregulation at risk of cerebral oedema. Contrib Nephrol. 1991. http://doi.org/10.1159/000420225  427. Davenport A, Honore PM. Continuous renal replacement therapy under special conditions like sepsis, burn, cardiac failure, neurotrauma, and liver failure. Semin Dialysis. 2021;34(6):457-71. http://doi.org/10.1111/sdi.13002  428. Davenport A, Will EJ, Davidson AM. Improved cardiovascular stability during continuous modes of renal replacement therapy in critically ill patients with acute hepatic and renal failure. Crit Care Med. 1993;21(3):328-38. http://doi.org/10.1097/00003246-199303000-00007  429. HT D, GD L. Intermittent versus Continuous Renal Replacement Therapy: a matter of controversy. Intensive & Critical Care Nursing. 2008(5). http://doi.org/10.1016/j.iccn.2008.02.001  430. L D, NR E, H I, S M, A R, Z Z, et al. Evaluation of the effectiveness of N-acetylcysteine on accelerating the recovery of renal failure in patients with leptospirosis, a randomized clinical trial study. Ann Med Surg. 2021. http://doi.org/10.1016/j.amsu.2021.102518  431. Dawidson I, Lu C, Palmer B, Peters P, Rooth P, Risser R, et al. Verapamil (VP) improves the outcome after renal transplantation (CRT). Transpl Int. 1992;5 Suppl 1:S60-2. http://doi.org/10.1007/978-3-642-77423-2_19  432. Dawood HFA, Abd Rabbih AAR, Fouad HA, El-Shafei MM, Hussein HW. Renal Restrictive Index (RRI) Versus Urinary Neutrophil Gelatinase-Associated Lipocalin (uNGAL) for early prediction of acute kidney injury (AKI) in adults undergoing elective cardiac surgeries with cardiopulmonary bypass: A prospective randomized observational study. Egypt J Anaesth. 2022;38(1):166-73. http://doi.org/10.1080/11101849.2022.2050042  433. De Bonis M, Lapenna E, Buzzatti N, La Canna G, Denti P, Pappalardo F, et al. Optimal results immediately after MitraClip therapy or surgical edge-to-edge repair for functional mitral regurgitation: Are they really stable at 4 years? Eur J Cardio-Thorac. 2016;50(3):488-94. http://doi.org/10.1093/ejcts/ezw093  434. De Fallois J, Weidhase L, Petros S. Myoglobin clearance in CVVHD using high cut-off filter versus CVVHDF with regional citrat anticoagulation: A prospective randomized controlled trial. Nephrol Dial Transpl. 2020;35(SUPPL 3):iii955. http://doi.org/10.1093/ndt/gfaa142.P0635  435. de Fijter JW, H H, O Ø, JS S, S S, FJ B, et al. Early Conversion From Calcineurin Inhibitor- to Everolimus-Based Therapy Following Kidney Transplantation: results of the Randomized ELEVATE Trial. Am J Transplant. 2017(7). http://doi.org/10.1111/ajt.14186  436. De Francisco A, H R, P L, B S, A Y, M J, et al. Achievement and maintenance of normokalaemia in patients with non-dialysis stage 5 chronic kidney disease. Nephrol Dial Transpl. 2015. http://doi.org/10.1093/ndt/gfv165.3  437. De Martin E, Londoño MC, Emamaullee J, Lerut J, Potts J, Aluvihare V, et al. The optimal immunosuppression management to prevent early rejection after liver transplantation: A systematic review of the literature and expert panel recommendations. Clin Transplant. 2022;36(10). http://doi.org/10.1111/ctr.14614  438. De Pietri L, Serra V, Preziosi G, Rompianesi G, Begliomini B. Perioperative effects of high doses of intraoperative thymoglobulin induction in liver transplantation. World J Transplant. 2015;5(4):320-8. http://doi.org/10.5500/wjt.v5.i4.320  439. De Souza Durão M, Dos Santos TOC, De Souza Oliveira MA, Monte JCM, Batista MC, Junior VGP, et al. Outcomes from a cohort of patients with acute kidney injury subjected to continuous venovenous hemodiafiltration: The role of negative fluid balance. Plos One. 2017;12(4). http://doi.org/10.1371/journal.pone.0175897  440. Delabranche X, Quenot JP, Lavigne T, Mercier E, François B, Severac F, et al. Early Detection of Disseminated Intravascular Coagulation during Septic Shock: A Multicenter Prospective Study. Crit Care Med. 2016;44(10):e930-9. http://doi.org/10.1097/CCM.0000000000001836  441. Á D, L T, R A, V B, M G, R A, et al. Glucocorticoids Decrease Longitudinal Bone Growth in Pediatric Kidney Transplant Recipients by Stimulating the FGF23/FGFR3 Signaling Pathway. J Bone Miner Res. 2019(10). http://doi.org/10.1002/jbmr.3761  442. A DB, M VA, M FB, AM LD, JL GG, E RS, et al. Early steroid withdrawal in pediatric renal transplantation. Rev Med Chile. 2006(11). http://doi.org/10.4067/s0034-98872006001100006  443. G D. Amphotericin B nephrotoxicity. J Antimicrob Chemoth. 2002(SUPL. S1). http://doi.org/10.1093/jac/49.suppl_1.37  444. Desai CS, Martin SS, Blumenthal RS. Non-cardiovascular effects associated with statins. BMJ (Online). 2014;349. http://doi.org/10.1136/bmj.g3743  445. G D, V P, S P, D G, R B, S B, et al. Soluble CD40 ligand is predictive of combined cardiovascular morbidity and mortality in patients on haemodialysis at a relatively short-term follow-up. Nephrology, Dialysis, Transplantation. 2011(9). http://doi.org/10.1093/ndt/gfq823  446. Dhifaoui K, Hajjej Z, Fatnassi A, Sellami W, Lab-Bene I, Ferjani M. On-line hemofltration versus conventional hemofltration in septic shock patients: Clinical safety and effectiveness. Ann Intensive Care. 2017;7(1):114. http://doi.org/10.1186/s13613-016-0224-7  447. Di Iorio B, Torraca S, Gustaferro P, Fazeli G, Heidland A. High-frequency external muscle stimulation in acute kidney injury (AKI): Potential shortening of its clinical course. Clin Nephrol. 2013;79(SUPPL. 13):S37-45. http://doi.org/10.5414/CNX77S101  448. Di Maio F, A F, C R, N P, G G, G S, et al. The short and long-term effects of hyperthermic intraperitoneal chemotherapy on renal function in platinum-sensitive recurrent ovarian cancer. Nephrol Dial Transpl. 2020(SUPPL 3). http://doi.org/10.1093/ndt/gfaa142.P0563  449. Diegeler A, Börgermann J, Kappert U, Breuer M, Böning A, Ursulescu A, et al. Off-pump versus on-pump coronary-artery bypass grafting in elderly patients. New Engl J Med. 2013;368(13):1189-98. http://doi.org/10.1056/NEJMoa1211666  450. Diekmann L. [Treatment of the hemolytic-uremic syndrome with streptokinase and heparin (author's transl)]. Klin Padiatr. 1980;192(5):430-5. http://doi.org/10.1055/s-2008-1035620  451. W D, M C, C T, A A, C M, LA H, et al. Testing the efficacy and safety of BIO101, for the prevention of respiratory deterioration, in patients with COVID-19 pneumonia (COVA study): a structured summary of a study protocol for a randomised controlled trial. Trials. 2021(1). http://doi.org/10.1186/s13063-020-04998-5  452. Dittrich S, Kurschat K, Dähnert I, Vogel M, Müller C, Alexi-Meskishvili V, et al. Renal function after cardiopulmonary bypass surgery in cyanotic congenital heart disease. Int J Cardiol. 2000;73(2):173-9. http://doi.org/10.1016/s0167-5273(00)00217-5  453. P D, P D, M D, J T, A C, A K. The effect of the use of a TNF-alpha inhibitor in hypothermic machine perfusion on kidney function after transplantation. Contemp Clin Trials. 2017. http://doi.org/10.1016/j.cct.2017.05.013  454. Dixon C, Rivera ET, Patel A, Pollack M. PREDICTION OF SHORT-TERM CREATININE CHANGE IN CRITICALLY ILL CHILDREN. Crit Care Med. 2024;52(1):S317. http://doi.org/10.1097/01.ccm.0001000928.24646.47  455. OS D, A E, A Y, Y A, A O, CB G. Analysis of liver function test abnormalities in kidney transplant recipients. Nephrol Dial Transpl. 2015. http://doi.org/10.1093/ndt/gfv202.88  456. A D, N P, JD P. Outcomes in kidney transplantation. Semin Nephrol. 2003(3). http://doi.org/10.1016/s0270-9295(03)00066-4  457. K D, M D, PF H, F E, K B, H R, et al. A Randomized Clinical Trial of Anti-IL-6 Antibody Clazakizumab in Late Antibody-Mediated Kidney Transplant Rejection. Journal of the American Society of Nephrology : JASN. 2021(3). http://doi.org/10.1681/ASN.2020071106  458. Doig GS, Simpson F, Bellomo R, Heighes PT, Sweetman EA, Chesher D, et al. Intravenous amino acid therapy for kidney function in critically ill patients: a randomized controlled trial. Intens Care Med. 2015;41(7):1197-208. http://doi.org/10.1007/s00134-015-3827-9  459. Dong Z, Shi L, Ye L, Xu Z, Zhou L. [Risk factors analysis of renal replacement therapy after liver transplantation and prognosis effect of initial treatment time]. Zhonghua Wei Zhong Bing Ji Jiu Yi Xue. 2018;30(11):1056-60. http://doi.org/10.3760/cma.j.issn.2095-4352.2018.011.009  460. S D, DFB W, JA R, SB D, JBW S, TL P, et al. The pharmacokinetics of meropenem and piperacillin-tazobactam during sustained low efficiency haemodiafiltration (SLED-HDF). Eur J Clin Pharmacol. 2020(2). http://doi.org/10.1007/s00228-019-02792-0  461. Dravid A, Kashiva R, Khan Z, Memon D, Kodre A, Potdar P, et al. Combination therapy of Tocilizumab and steroid for management of COVID-19 associated cytokine release syndrome: A single center experience from Pune, Western India. Medicine (United States). 2021;100(29):E26705. http://doi.org/10.1097/MD.0000000000026705  462. Drey M, Behnes M, Kob R, Lepiorz D, Hettwer S, Bollheimer C, et al. C-terminal agrin fragment (CAF) reflects renal function in patients suffering from severe sepsis or septic shock. Clin Lab. 2015;61(1-2):69-76. http://doi.org/10.7754/clin.lab.2014.140724  463. DRKS. Citrate based CVVH in Patients with Acute Renal Failure: comparison of a New Citrate Based Hemofiltration Solution vs. Standard Bicarbonate Based Hemofiltration Solution. Https://Trialsearch.Who.Int/Trial2.Aspx?TrialID=DRKS00000224. 2009.  464. DRKS. A prospective, multi-center, randomized, double blinded, placebo-controlled study for the evaluation of Iloprost in the early postoperative period after liver transplantation. Https://Trialsearch.Who.Int/Trial2.Aspx?TrialID=DRKS00003514. 2012.  465. DRKS. Early diagnosis and Treatment of acute kidney injury. Https://Trialsearch.Who.Int/Trial2.Aspx?TrialID=DRKS00010530. 2016.  466. DRKS. Cytokine adsorption in patients with acute on chronic liver failure (CYTOHEP) – a single center, open-label, randomized, controlled intervention trial. Https://Trialsearch.Who.Int/Trial2.Aspx?TrialID=DRKS00026082. 2021.  467. DRKS. Hemadsorption through supportive establishment of an Oxiris® filter in septic shock with suspected cytokine release syndrome - a randomized controlled intervention study. Https://Trialsearch.Who.Int/Trial2.Aspx?TrialID=DRKS00027287. 2022.  468. DRKS. Xerostomia in Dialysis Patients – Oral Care to Reduce Hyposalivation, Dental Biofilms and Gingivitis in Patients with Terminal Renal Insufficiency: a Randomized Clinical Study. Https://Trialsearch.Who.Int/Trial2.Aspx?TrialID=DRKS00029636. 2022.  469. DRKS. Efficacy and safety of Tiprelestat for treatment of severe COVID-19 (COMCOVID trial). Https://Trialsearch.Who.Int/Trial2.Aspx?TrialID=DRKS00031463. 2023.  470. Duan Z, Jijun L, Cai G, Xiangmei C, Fengkun C. Prevalence, outcome and modalities of renal replacement therapy in burn patients: A systematic review and meta-analysis. Nephrol Dial Transpl. 2019;34:a289. http://doi.org/10.1093/ndt/gfz106.FP710  471. Duggan KA, Macdonald GJ, Charlesworth JA, Pussell BA. Verapamil prevents post-transplant oliguric renal failure. Clin Nephrol. 1985;24(6):289-91.  472. GJ D, JH B, RA W. Renal support in critically ill patients: low-dose dopamine or low-dose dobutamine? Crit Care Med. 1994(12).  473. Düngen HD, Von Heymann C, Ronco C, Kox WJ, Spies CD. Renal replacement therapy: Physical properties of hollow fibers influence efficiency. Int J Artif Organs. 2001;24(6):357-66. http://doi.org/10.1177/039139880102400601  474. Durante-Mangoni E, Andini R, Signoriello S, Cavezza G, Murino P, Buono S, et al. Acute kidney injury during colistin therapy: a prospective study in patients with extensively-drug resistant Acinetobacter baumannii infections. Clin Microbiol Infec. 2016;22(12):984-9. http://doi.org/10.1016/j.cmi.2016.08.004  475. Durmaz I, Yagdi T, Calkavur T, Mahmudov R, Apaydin AZ, Posacioglu H, et al. Prophylactic dialysis in patients with renal dysfunction undergoing on-pump coronary artery bypass surgery. Ann Thorac Surg. 2003;75(3):859-64. http://doi.org/10.1016/S0003-4975(02)04635-0  476. A D, JM P, S F, M DCR, L R, D K, et al. Long-Term Outcomes in Belatacept- Versus Cyclosporine-Treated Recipients of Extended Criteria Donor Kidneys: final Results From BENEFIT-EXT, a Phase III Randomized Study. Am J Transplant. 2016(11). http://doi.org/10.1111/ajt.13830  477. JC D, C M, MN P, JM L, C G, F M, et al. Inhibition of neutral endopeptidase stimulates renal sodium excretion in patients with chronic renal failure. Clinical Science (London, England : 1979). 1993(1). http://doi.org/10.1042/cs0840031  478. A D, M J, IC E, B S, C A, D G, et al. Lassa fever clinical course and setting a standard of care for future randomized trials: a protocol for a cohort study of Lassa-infected patients in Nigeria (LASCOPE). Travel Med Infect Di. 2020. http://doi.org/10.1016/j.tmaid.2020.101557  479. Eastburg L, Russler-Germain DA, Abboud R, Westervelt P, DiPersio JF, Becker MW, et al. Increased Early Mortality after Fludarabine and Melphalan Conditioning with Peripheral Blood Grafts in Haploidentical SCT with Post-Transplant Cyclophosphamide. Blood. 2019;134:4496. http://doi.org/10.1182/blood-2019-129872  480. FY E, S K, JD B, A V. A Pilot Study Evaluating the Effect of Cooler Dialysate Temperature on Hemodynamic Stability During Prolonged Intermittent Renal Replacement Therapy in Acute Kidney Injury. Crit Care Med. 2019(2). http://doi.org/10.1097/CCM.0000000000003508  481. T E, J H, S H, I L, F K, G V, et al. Clearance of Selected Plasma Cytokines with Continuous Veno-Venous Hemodialysis Using Ultraflux EMiC2 versus Ultraflux AV1000S. Blood Purificat. 2017(4). http://doi.org/10.1159/000478965  482. ES EAH, AM K, W EDHE. Clinical outcomes of Mechanical circulatory support with Impella versus intra-aortic balloon pump in cardiogenic shock complicating acute myocardial infarction. Journal of Cardiovascular Disease Research. 2022(5). http://doi.org/10.31838/jcdr.2022.13.05.121  483. MI E, SA H. Single-dose linezolid pharmacokinetics in critically ill patients with impaired renal function especially chronic hemodialysis patients. Biopharm Drug Dispos. 2014(7). http://doi.org/10.1002/bdd.1910  484. G E, K G, I G, S T, NT O, H S, et al. The Effects of Genistein as Supplement to Oral/ Enteral Nutrition on Inflammatory Cytokines in Septic ICU patients: a Prospective, Single-center, Controlled Pilot Study. Erciyes Med J. 2023(2). http://doi.org/10.14744/etd.2022.55492  485. Elbadawi A, Elgendy IY, Saad M, Megaly M, Mentias A, Abuzaid AS, et al. Meta-Analysis of Trials on Prophylactic Use of Levosimendan in Patients Undergoing Cardiac Surgery. Ann Thorac Surg. 2018;105(5):1403-10. http://doi.org/10.1016/j.athoracsur.2017.11.027  486. Elbatanouny AM, Ragheb AM, Abdelbary AM, Fathy H, Massoud AM, Abd El Latif A, et al. Percutaneous nephrostomy versus JJ ureteric stent as the initial drainage method in kidney stone patients presenting with acute kidney injury: A prospective randomized study. Int J Urol. 2020;27(10):916-21. http://doi.org/10.1111/iju.14331  487. Elder J, McComb J, Lirette S, Herndon B, Yancey G, Mohammed A, et al. Pre-emptive intraoperative administration of PCC4 in cardiac surgery patients at high risk of bleeding: A pilot study. J Cardiac Surg. 2022;37(12):5130-4. http://doi.org/10.1111/jocs.17224  488. A E, A G, S M, K M, H I, S P, et al. NON-INVASIVE VENTILATION DECREASES THE NEED FOR INVASIVE MECHANICAL VENTILATION AND HOSPITAL ACQUIRED PNEUMONIA IN ACUTE HYPOXEMIC RESPIRATORY FAILURE DUE TO ACUTE PANCREATITIS (BIPAP TRIAL): a RANDOMIZED CONTROLLED TRIAL. Gastroenterology. 2023(6). http://doi.org/10.1016/S0016-5085(23)03419-4  489. Eliaschewitz FG, Canani LH. Advances in GLP-1 treatment: focus on oral semaglutide. Diabetology and Metabolic Syndrome. 2021;13(1). http://doi.org/10.1186/s13098-021-00713-9  490. Elitok S, Haase-Fielitz A, Ernst M, Haase M. Urinary ngal:hepcidin-25 ratio versus urinary ngal for early identification of patients at risk for acute renal replacement therapy and death after cardiac surgery. Nephrol Dial Transpl. 2021;36(SUPPL 1):i270. http://doi.org/10.1093/ndt/gfab083.002  491. Elitok S, Kuppe H, Devarajan P, Bellomo R, Isermann B, Westphal S, et al. Urinary Neutrophil Gelatinase-Associated Lipocalin/Hepcidin-25 Ratio for Early Identification of Patients at Risk for Renal Replacement Therapy after Cardiac Surgery: A Substudy of the BICARBONATE Trial. Anesth Analg. 2021;133(6):1510-9. http://doi.org/10.1213/ANE.0000000000005741  492. ElSharkawy M, Metaal M, Bishary W. Impact of ingestion paraphenylenediamine (PPD) on kidney function in upper Egypt. Nephrol Dial Transpl. 2015;30:iii454. http://doi.org/10.1093/ndt/gfv190.44  493. MS E, AM S, AI S, S S, DM S, AM Y, et al. Surgical complications and graft function following live-donor extraperitoneal renal transplantation in children 20 kg or less. J Pediatr Urol. 2014(4). http://doi.org/10.1016/j.jpurol.2013.12.013  494. ElSheemy MS, Shouman AM, Shoukry AI, ElShenoufy A, Aboulela W, Daw K, et al. Ureteric stents vs percutaneous nephrostomy for initial urinary drainage in children with obstructive anuria and acute renal failure due to ureteric calculi: a prospective, randomised study. Bju Int. 2015;115(3):473-9. http://doi.org/10.1111/bju.12768  495. Ely S, Gologorsky RC, Huyser MR, Beattie G, Chang CK. NSQIP database analysis does not support preoperative antibiotic use for outpatient laparoscopic cholecystectomy. Surgical Endoscopy. 2019;33:S231. http://doi.org/10.1007/s00464-019-06704-2  496. Emrich IE, Tokcan M, Al Ghorani H, Schwenger V, Mahfoud F. Current aspects of heart-kidney interactions: Summary of important clinical studies from 2020. Herz. 2022;47(2):150-7. http://doi.org/10.1007/s00059-021-05043-0  497. ZH E, JW P, RJ W, P D, CL E, JV B, et al. Improved performance of urinary biomarkers of acute kidney injury in the critically ill by stratification for injury duration and baseline renal function. Kidney Int. 2011(10). http://doi.org/10.1038/ki.2010.555  498. Endre ZH, Walker RJ, Pickering JW, Shaw GM, Frampton CM, Henderson SJ, et al. Early intervention with erythropoietin does not affect the outcome of acute kidney injury (the EARLYARF trial). Kidney Int. 2010;77(11):1020-30. http://doi.org/10.1038/ki.2010.25  499. Eremenko AA, Minbolatova NM, Kaabak MM, Babenko NN. Neutrophil gelatinase-associated lipocalin (u-NGAL) in the assessment of renal function in patients after kidney allotransplantation. Anesteziologiia I Reanimatologiia. 2014;59(5):10-5.  500. JW E, MH A, JK B, BG D, MR D, JC E, et al. Recombinant human erythropoietin in anemic patients with end-stage renal disease. Results of a phase III multicenter clinical trial. Ann Intern Med. 1989(12). http://doi.org/10.7326/0003-4819-111-12-992  501. FS E, AL I, DJ C. Sirolimus immunotherapy: the Albany Medical Center experience. Transpl P. 2003(3 Suppl). http://doi.org/10.1016/S0041-1345(03)00242-2  502. Esposito C, Torreggiani M, Castoldi F, Migotto C, Serpieri N, Grosjean F, et al. Effect of erythropoietin on short term I/R reperfusion injury in rats. Nephrol Dial Transpl. 2012;27:ii346-7. http://doi.org/10.1093/ndt/gfs234  503. Esson ML, Schrier RW. Diagnosis and treatment of acute tubular necrosis. Ann Intern Med. 2002;137(9):744-52. http://doi.org/10.7326/0003-4819-137-9-200211050-00010  504. I E, O T, J B, A T, A AN, de Ligny B H, et al. A 50% reduction in cyclosporine exposure in stable renal transplant recipients: renal function benefits. Nephrology, Dialysis, Transplantation. 2010(9). http://doi.org/10.1093/ndt/gfq135  505. DE E. The Effects of Nitric Oxide for Inhalation during Left Ventricular Assist Device (LVAD) Implantation. - INOT41. Https://Trialsearch.Who.Int/Trial2.Aspx?TrialID=EUCTR2005-000326-24-DE. 2005.  506. AT E. Anticoagulation with Prostaglandin E1 and unfractioned Heparin versus Placebo and unfractioned Heparin during continuous venovenous hemofiltration. Https://Trialsearch.Who.Int/Trial2.Aspx?TrialID=EUCTR2005-003211-69-at. 2005.  507. SE E. A multicentre, randomised, double-blind, placebo-controlled study of the effects of KW-3902 Injectable Emulsion on heart failure signs and symptoms and renal function in subjects with acute heart failure syndrome and renal impairment who are hospitalised for volume overload and require intravenous diuretic therapy. - PROTECT {CKI-302}. Https://Trialsearch.Who.Int/Trial2.Aspx?TrialID=EUCTR2006-001637-18-SE. 2006.  508. NL E. A multicentre, randomized, double-blind, placebo controlled study of the effects of KW-3902 Injectable Emulsion on heart failure signs and symptoms, diuresis, renal function, and clinical outcomes in subjects hospitalized with worsening renal function and heart failure requiring intravenous therapy. - REACH UP. Https://Trialsearch.Who.Int/Trial2.Aspx?TrialID=EUCTR2006-006843-29-NL. 2007.  509. SE E. A 24 month study to test everolimus in patients who have had a liver transplant. Https://Trialsearch.Who.Int/Trial2.Aspx?TrialID=EUCTR2007-001821-85-SE. 2007.  510. SK E. Randomized, controlled, double-blind multicenter safety study to evaluate the safety and immunogenicity of subcutaneous EPO HEXAL vs. ERYPO® in the treatment of anemia associated with chronic renal insufficiency in predialysis patients. Https://Trialsearch.Who.Int/Trial2.Aspx?TrialID=EUCTR2007-001906-26-SK. 2008.  511. BE E. A PHASE 2 RANDOMIZED, MULTICENTER, ACTIVE COMPARATOR-CONTROLLED TRIAL TO EVALUATE THE SAFETY AND EFFICACY OF COADMINISTRATION OF CP-690,550 AND MYCOPHENOLATE MOFETIL / MYCOPHENOLATE SODIUM IN DE NOVO KIDNEY ALLOGRAFT RECIPIENTS - N/A. Https://Trialsearch.Who.Int/Trial2.Aspx?TrialID=EUCTR2007-001984-31-be. 2007.  512. DE E. A multicentre, double-blind, randomised placebo-controlled trial of sodium bicarbonate to prevent acute kidney injury in patients undergoing cardiopulmonary bypass surgery. - Natriumbikarbonat-Studie. Https://Trialsearch.Who.Int/Trial2.Aspx?TrialID=EUCTR2007-002223-32-DE. 2008.  513. BE E. A Phase-II, double-blind, randomized, placebo-controlled proof of concept study on the safety and early efficacy of Alkaline Phosphatase in sepsis patients with Renal Failure. - A phase-II db plb-ctrld RCT on Safety and early efficacy of AP in sepsis patients with renal failure. Https://Trialsearch.Who.Int/Trial2.Aspx?TrialID=EUCTR2007-003866-16-be. 2009.  514. GR E. A MULTICENTER, FOUR ARM, RANDOMIZED, OPEN LABEL CLINICAL STUDY INVESTIGATING OPTIMIZED DOSING IN A PROGRAF®-/ ADVAGRAF®-BASED IMMUNOSUPPRESSIVE REGIMEN IN KIDNEY TRANSPLANT SUBJECTS. Protocol for Phase IIIb Study of Advagraf® (FK506E (MR4)) - Short Title: OSAKA Study. Https://Trialsearch.Who.Int/Trial2.Aspx?TrialID=EUCTR2007-005376-13-GR. 2008.  515. IT E. ?Fenoldopam end Renal failure? (FENO-HSR). Https://Trialsearch.Who.Int/Trial2.Aspx?TrialID=EUCTR2008-002077-12-IT. 2012.  516. GB E. A randomised study to assess the effects of Epoetin Beta on renal function in patients with critical illness(with or without multi-organ failure). Https://Trialsearch.Who.Int/Trial2.Aspx?TrialID=EUCTR2008-003733-24-GB. 2008.  517. CZ E. Lenalidomide and Dexamethasone for treatment of patients with acute myeloma (light chain)-induced renal failure - LD. Https://Trialsearch.Who.Int/Trial2.Aspx?TrialID=EUCTR2008-006497-15-CZ. 2009.  518. DE E. A Therapeutic Exploratory Study to Determine the Efficacy and Safety of Calcineurin-Inhibitor-Free de-novo Immunosuppression after Liver Transplantation. Https://Trialsearch.Who.Int/Trial2.Aspx?TrialID=EUCTR2008-008754-23-DE. 2009.  519. GB E. Preoperative volume replacement vs. usual care in diabetic patients having CABG surgery: a randomised controlled trial - VeRDiCT: volume replacement vs. usual care in diabetics having CABG. Https://Trialsearch.Who.Int/Trial2.Aspx?TrialID=EUCTR2009-013159-31-GB. 2010.  520. GB E. A large, international, placebo-controlled, factorial trial to assess the impact of low-dose clonidine and acetyl-salicyclic acid (ASA) in patients undergoing noncardiac surgery who are at risk of a perioperative cardiovascular event. PeriOperative ISchemic Evaluation-2 Trial. - PeriOperative ISchemic Evaluation-2 Trial. Https://Trialsearch.Who.Int/Trial2.Aspx?TrialID=EUCTR2009-018173-31-GB. 2011.  521. DE E. Controlled, Randomized, Prospective, Double-Blind, Multicenter, Phase I/II, Dose-Escalation Study in order to assess the Safety, the behaviour of the investigational product in the human body and the Clinical Activity of I5NP to prevent Delayed Graft Function in Patients Undergoing Kidney Transplantation from deceased donors. Https://Trialsearch.Who.Int/Trial2.Aspx?TrialID=EUCTR2010-020989-20-DE. 2010.  522. FI E. Dexmedetomidine pharmacokinetics during continuous venovenous hemofiltration and hemodiafiltration in critically ill patients - Dexmedetomidine study. Https://Trialsearch.Who.Int/Trial2.Aspx?TrialID=EUCTR2010-023455-28-FI. 2010.  523. ES E. The Effect the Study Drug has on the Heart in Patients with Type 2 Diabetes. Https://Trialsearch.Who.Int/Trial2.Aspx?TrialID=EUCTR2010-023799-21-ES. 2011.  524. DE E. Efficacy and Safety of Everolimus in Liver Transplant Recipients of Living Donor Liver Transplants. Https://Trialsearch.Who.Int/Trial2.Aspx?TrialID=EUCTR2010-024527-25-DE. 2013.  525. GB E. Treatment of a low blood count before heart surgery comparing iron supplements taken by mouth and the combination of iron given by intravenous drip and a drug to increase the blood count. Https://Trialsearch.Who.Int/Trial2.Aspx?TrialID=EUCTR2011-003695-36-GB. 2012.  526. ES E. A pilot clinical study in kidney transplant to assess the efficacy and safety of cardiotrophin-1. Https://Trialsearch.Who.Int/Trial2.Aspx?TrialID=EUCTR2011-004223-12-ES. 2012.  527. GB E. A clinical study (Phase II) in kidney transplant patients who are at high risk of developing clinical complications, immediately post transplant, requiring renal dialysis. The 3 part study is to assess an appropriate dose and the safety, tolerability and effiacy of OPN-305 as a treatment to prevent the clinical complication, when compared against placebo in a randomised, double blind manner. Https://Trialsearch.Who.Int/Trial2.Aspx?TrialID=EUCTR2012-001455-39-GB. 2012.  528. DE E. A clinical study on the application of physostigminsalicylate (Anticholium®) as auxiliary measure in perioperative blood poisoning/its life-threatening form. Https://Trialsearch.Who.Int/Trial2.Aspx?TrialID=EUCTR2012-001650-26-DE. 2012.  529. GB E. LeoPARDS - An efficacy and mechanism evaluation study of Levosimendan for the Prevention of Acute oRgan Dysfunction in Sepsis. Https://Trialsearch.Who.Int/Trial2.Aspx?TrialID=EUCTR2012-005159-18-GB. 2013.  530. DE E. 6 month, multi-center, open-label, prospective, randomized trial, investigating a standard regimen of an advagraf based immunosuppressive regimen in de-novo renal transplant patients versus a slower dose tapering and lower starting dose of Avagraf. Https://Trialsearch.Who.Int/Trial2.Aspx?TrialID=EUCTR2013-001770-19-DE. 2014.  531. GB E. Investigation of Zibotentan for kidney disease in scleroderma. Https://Trialsearch.Who.Int/Trial2.Aspx?TrialID=EUCTR2013-003200-39-GB. 2014.  532. DE E. A Phase 2b study of CSL112 in subjects with acute myocardial infarction. Https://Trialsearch.Who.Int/Trial2.Aspx?TrialID=EUCTR2013-003458-26-DE. 2014.  533. DE E. Study of Eculizumab in Adult Subjects at Risk for Developing Delayed Graft Function. Https://Trialsearch.Who.Int/Trial2.Aspx?TrialID=EUCTR2013-004650-25-DE. 2014.  534. DK E. Fluid optimization in urgent abdominal surgery. Https://Trialsearch.Who.Int/Trial2.Aspx?TrialID=EUCTR2015-000563-14-DK. 2015.  535. DE E. A phase II randomized, placebo-controlled, double-blind, dose-escalation study to evaluate safety, pharmacokinetics and pharmacodynamic dose response relationship of IFX-1 in patients undergoing complex cardiac surgery (CARDIAC). Https://Trialsearch.Who.Int/Trial2.Aspx?TrialID=EUCTR2015-003036-12-DE. 2015.  536. DE E. Phase 2 Study to see if QPI-1002 is effective and safe of for the Prevention of Acute Kidney Injury in Subjects at High Risk for AKI following Cardiac Surgery. Https://Trialsearch.Who.Int/Trial2.Aspx?TrialID=EUCTR2015-003113-15-DE. 2016.  537. FR E. Safety profile evaluation of TICagrelor Alone compared to a combination of lysine acetylsalicylate - Clopidogrel in the context of Transcatheter Aortic Valve Implantation (TAVI). Https://Trialsearch.Who.Int/Trial2.Aspx?TrialID=EUCTR2015-004144-20-FR. 2015.  538. NL E. Study on the effects of EA-230 on the immune system following heart surgery. Https://Trialsearch.Who.Int/Trial2.Aspx?TrialID=EUCTR2015-005600-28-NL. 2016.  539. GB E. Eculizumab in Shiga-toxin producing E. Coli Haemolytic Uraemic Syndrome (ECUSTEC): a Randomised, Double-Blind, Placebo-Controlled Trial. Https://Trialsearch.Who.Int/Trial2.Aspx?TrialID=EUCTR2016-000997-39-GB. 2018.  540. SE E. Spironolactone Initiation Registry Randomized Interventional Trial in Heart Failure with Preserved Ejection Fraction. Https://Trialsearch.Who.Int/Trial2.Aspx?TrialID=EUCTR2016-002019-16-SE. 2016.  541. NL E. Controlled, international study conducted in several centres on the safety and therapeutic effect of a Hydroxylethyl-starch (HES) solution versus an electrolyte solution in patients undergoing elective abdominal surgery. Https://Trialsearch.Who.Int/Trial2.Aspx?TrialID=EUCTR2016-002162-30-NL. 2017.  542. DE E. Pragmatic, controlled, international study conducted in several centres on the safety and therapeutic effect of a Hydroxylethyl starch (HES) solution versus an electrolyte solution in trauma patients. Https://Trialsearch.Who.Int/Trial2.Aspx?TrialID=EUCTR2016-002176-27-DE. 2016.  543. DE E. Liberal transfusion strategy to prevent mortality and anaemia-associated, ischaemic events in elderly non-cardiac surgical patients. Https://Trialsearch.Who.Int/Trial2.Aspx?TrialID=EUCTR2016-004446-29-DE. 2017.  544. ES E. Efficacy and safety of human plasma-derived C1-esterase inhibitor as add-on to standard of care for the treatment of refractory antibody mediated rejection (AMR) in adult renal transplant recipients. Https://Trialsearch.Who.Int/Trial2.Aspx?TrialID=EUCTR2017-000348-17-ES. 2017.  545. AT E. The influence of the medication Willfact on the blood loss of patient during the use of a heart-lung machine. Https://Trialsearch.Who.Int/Trial2.Aspx?TrialID=EUCTR2017-003036-37-at. 2017.  546. ES E. Comparative study of two types of cardioplegia during cardiac surgery in the adult patient. Https://Trialsearch.Who.Int/Trial2.Aspx?TrialID=EUCTR2017-005144-14-ES. 2018.  547. DK E. DAN-WAR-D Danish Warfarin-Dialysis Study Anticoagulation for prevention of stroke in patients with end-stage renal disease on dialysis with atrial fibrillation: a national randomized trial. Https://Trialsearch.Who.Int/Trial2.Aspx?TrialID=EUCTR2018-000484-86-DK. 2018.  548. ES E. Phase 3 Study to see if QPI-1002 is effective and safe for the Prevention of Acute Kidney Injury in Subjects at High Risk for AKI following Cardiac Surgery. Https://Trialsearch.Who.Int/Trial2.Aspx?TrialID=EUCTR2018-000757-49-ES. 2018.  549. FR E. Ilomedin for treatment of septic shock with persistent microperfusion defects. Https://Trialsearch.Who.Int/Trial2.Aspx?TrialID=EUCTR2018-001709-10-FR. 2018.  550. GB E. Transplant Antibody-mediated Rejection: guiding Effective Treatments-1 (TAR: GET-1). Https://Trialsearch.Who.Int/Trial2.Aspx?TrialID=EUCTR2018-002882-20-GB. 2019.  551. ES E. A study to measure how safe and successful Clazakizumab is in preventing kidney transplant rejection. Https://Trialsearch.Who.Int/Trial2.Aspx?TrialID=EUCTR2018-003682-34-ES. 2019.  552. GR E. BENEFIT OF ANTI-COAGULATION THERAPY IN PATIENTS WITH SEPTIC SHOCK. Https://Trialsearch.Who.Int/Trial2.Aspx?TrialID=EUCTR2018-003726-93-GR. 2019.  553. AT E. Comparison of two substitution fluids (BiphozylÂ® and PhoxiliumÂ® ) during continuous renal replacement therapy in adult critically ill patients with acute kidney injury and their effects on acid-base status and respiratory situation. â€“ A prospective, randomized, controlled, open, cross-over, Phase II, single-center pilot study. Https://Trialsearch.Who.Int/Trial2.Aspx?TrialID=EUCTR2019-001262-15-at. 2020.  554. HU E. A study to test the effect of empagliflozin in patients who are in hospital for acute heart failure. Https://Trialsearch.Who.Int/Trial2.Aspx?TrialID=EUCTR2019-002946-19-HU. 2020.  555. GB E. PlasmaLyte Usage and assessment of kidney Transplant Outcomes in children: the PLUTO trial. Https://Trialsearch.Who.Int/Trial2.Aspx?TrialID=EUCTR2019-003025-22-GB. 2019.  556. FR E. Lessening Organ Dysfunction with VITamin C (LOVIT). Https://Trialsearch.Who.Int/Trial2.Aspx?TrialID=EUCTR2019-003350-80-FR. 2019.  557. IT E. Comparison of two different volumes of colonoscopy fluid in hemodialysis patients: randomized non-inferiority study. Https://Trialsearch.Who.Int/Trial2.Aspx?TrialID=EUCTR2019-004840-30-IT. 2020.  558. FR E. Patients Admitted to ICU with proven or suspected infection as the main diagnosis. Https://Trialsearch.Who.Int/Trial2.Aspx?TrialID=EUCTR2020-000296-21-FR. 2020.  559. NL E. A phase II/III study of IFX-1 in patients with severe COVID-19 Pneumonia. Https://Trialsearch.Who.Int/Trial2.Aspx?TrialID=EUCTR2020-001335-28-NL. 2020.  560. IE E. A randomized double-blind placebo-controlled trial of intravenous plasma-purified alpha-1 antitrypsin for severe COVID-19 illness. Https://Trialsearch.Who.Int/Trial2.Aspx?TrialID=EUCTR2020-001391-15-IE. 2020.  561. DE E. Evaluation of efficacy and safety of an tocilizumab treatment in patients with severe COVID-19 pneumonia. Https://Trialsearch.Who.Int/Trial2.Aspx?TrialID=EUCTR2020-001408-41-DE. 2020.  562. DK E. Senicapoc treatment of COVID-19 positive patients in intensive care. Https://Trialsearch.Who.Int/Trial2.Aspx?TrialID=EUCTR2020-001420-34-DK. 2020.  563. FR E. Dexamethasone and oxygen support strategies in ICU patients with Covid-19 pneumonia. Https://Trialsearch.Who.Int/Trial2.Aspx?TrialID=EUCTR2020-001457-43-FR. 2020.  564. FR E. HYdrocortisone and VAsopressin in Post-RESuscitation Syndrome. Https://Trialsearch.Who.Int/Trial2.Aspx?TrialID=EUCTR2020-001620-33-FR. 2020.  565. FR E. X06 to rescue acute respiratory distress syndrome during Covid-19 pneumonia : FX-COVID. Https://Trialsearch.Who.Int/Trial2.Aspx?TrialID=EUCTR2020-002056-20-FR. 2020.  566. FR E. Opioid-Free Anesthesia in Cardiac Surgery. Https://Trialsearch.Who.Int/Trial2.Aspx?TrialID=EUCTR2020-002126-90-FR. 2020.  567. RO E. Open-label, pharmacokinetic, pharmacodynamic, ascending dose safety lead-in followed by a single-center, placebo-controlled, double-blind, adaptive, safety and efficacy, pilot study of Trans Sodium Crocetinate (TSC) in SARS-CoV-2 infected subjects. Https://Trialsearch.Who.Int/Trial2.Aspx?TrialID=EUCTR2020-002369-32-RO. 2020.  568. RO E. A PHASE 2 RANDOMIZED, DOUBLE-BLIND, PLACEBO-CONTROLLED, PROOF-OF-CONCEPT STUDY TO EVALUATE THE EFFICACY AND SAFETY OF RECOMBINANT HUMAN PLASMA GELSOLIN (RHU-PGSN) ADDED TO STANDARD OF CARE FOR TREATMENT OF SUBJECTS WITH SEVERE COVID-19 PNEUMONIA. Https://Trialsearch.Who.Int/Trial2.Aspx?TrialID=EUCTR2020-002460-31-RO. 2020.  569. GR E. Treatment of patients with coronavirus infection with immunoglobulin. Https://Trialsearch.Who.Int/Trial2.Aspx?TrialID=EUCTR2020-002542-16-GR. 2020.  570. FR E. Lessening Organ Dysfunction with VITamin C in septic ARDS. Https://Trialsearch.Who.Int/Trial2.Aspx?TrialID=EUCTR2020-003923-40-FR. 2020.  571. GR E. ZEUS – A research study to look at how ziltivekimab works compared to placebo in people with cardiovascular disease, chronic kidney disease and inflammation. Https://Trialsearch.Who.Int/Trial2.Aspx?TrialID=EUCTR2020-004853-59-GR. 2021.  572. DE E. An unblinded clinical trial investigating the active substance infliximab in the treatment of severe COVID-19 at multiple study centers. Https://Trialsearch.Who.Int/Trial2.Aspx?TrialID=EUCTR2021-002098-25-DE. 2021.  573. NL E. Rescue alkaline posphatase as a treatment for acute kidney injury. Https://Trialsearch.Who.Int/Trial2.Aspx?TrialID=EUCTR2021-002505-10-NL. 2021.  574. DE E. Phase 2 Study to see if RMC-035 is effective and safe in Subjects at High Risk for Acute Kidney Injury following Cardiac Surgery. Https://Trialsearch.Who.Int/Trial2.Aspx?TrialID=EUCTR2021-004040-19-DE. 2021.  575. DK E. Outcomes for people with cardiogenic shock after myocardial infarction by comparing two blood pressure targets. Https://Trialsearch.Who.Int/Trial2.Aspx?TrialID=EUCTR2021-005551-36-DK. 2023.  576. DK E. Dexamethasone, olanzapine, hemodynamics, and ventilation in cardiac surgery. Https://Trialsearch.Who.Int/Trial2.Aspx?TrialID=EUCTR2021-005618-32-DK. 2021.  577. PL E. Efficacy comparison of two doses of vitamin D3 in critically ill patients undergoing continuous renal replacement therapy - NephroD. Https://Trialsearch.Who.Int/Trial2.Aspx?TrialID=EUCTR2021-006789-19-PL. 2022.  578. Fabbri LP, Nucera M, Al Malyan M, Becchi C. Regional anticoagulation and antiaggregation for CVVH in critically ill patients: A prospective, randomized, controlled pilot study. Acta Anaesth Scand. 2010;54(1):92-7. http://doi.org/10.1111/j.1399-6576.2009.02031.x  579. Faguer S, Del Bello A, Danet C, Renaudineau Y, Izopet J, Kamar N. Apolipoprotein-A-I for severe COVID-19-induced hyperinflammatory states: A prospective case study. Front Pharmacol. 2022;13. http://doi.org/10.3389/fphar.2022.936659  580. Fajardo C, Sanchez CP, Cutler D, Sahney S, Sheth R. Inpatient citrate-based hemodialysis in pediatric patients. Pediatr Nephrol. 2016;31(10):1667-72. http://doi.org/10.1007/s00467-016-3403-8  581. Fakhari S, Bavil FM, Bilehjani E, Abolhasani S, Mirinazhad M, Naghipour B. Prophylactic furosemide infusion decreasing early major postoperative renal dysfunction in on-pump adult cardiac surgery: A randomized clinical trial. Res Rep Urol. 2017;9:5-13. http://doi.org/10.2147/RRU.S126134  582. SJ F, WR P, GC O. Early or Late Conversion From Tac-BD to Tac-BD in Renal Transplantation: when is the Right Time? Transpl P. 2015(6). http://doi.org/10.1016/j.transproceed.2015.05.025  583. Fang Q, Zhao X. Clinical effect of combined ulinastatin and continuous renal replacement therapy on management of severe sepsis with acute kidney injury. Trop J Pharm Res. 2017;16(4):925-30. http://doi.org/10.4314/tjpr.v16i4.26  584. Farag M, Badowski D, Koschny R, Skopp G, Brcic A, Szabo GB. Extracorporeal life support and digoxin-specific Fab fragments for successful management of Taxus baccata intoxication with low output and ventricular arrhythmia. Am J Emerg Med. 2017;35(12):1983-7. http://doi.org/10.1016/j.ajem.2017.09.031  585. Farese S, Jakob SM, Kalicki R, Frey FJ, Uehlinger DE. Treatment of acute renal failure in the intensive care unit: Lower costs by intermittent dialysis than continuous venovenous hemodiafiltration. Artif Organs. 2009;33(8):634-40. http://doi.org/10.1111/j.1525-1594.2009.00794.x  586. S F, WF P, M G, PD L. Impact of initial blood pressure on antihypertensive response in patients with acute hypertension. Am J Emerg Med. 2014(8). http://doi.org/10.1016/j.ajem.2014.03.021  587. Fatima R, Gowrishankar S. Myoglobinuria-induced acute kidney injury secondary to Covishield TM vaccination. Indian J Nephrol. 2022;32(5):484-7. http://doi.org/10.4103/ijn.ijn_257_21  588. IG F, I D, S P, P P, MG N, A C, et al. Effects of L-carnitine on oxidative stress responses in patients with renal disease. Medicine and Science in Sports and Exercise. 2010(10). http://doi.org/10.1249/MSS.0b013e3181dbacab  589. IG F, P P, A S, A C, S P, D S, et al. Acute exercise may exacerbate oxidative stress response in hemodialysis patients. Nephron. Clinical Practice. 2008(2). http://doi.org/10.1159/000139990  590. E F, G S, MP S, JA P, J R, F C. Tacrolimus plus mycophenolate mofetil vs. cyclosporine plus everolimus in deceased donor kidney transplant recipients: three-yr results of a single-center prospective clinical trial. Clin Transplant. 2013(4). http://doi.org/10.1111/ctr.12141  591. N F, I B, M J, M E, R B. A pilot randomized controlled crossover study comparing regional heparinization to regional citrate anticoagulation for continuous venovenous hemofiltration. Int J Artif Organs. 2007(4). http://doi.org/10.1177/039139880703000404  592. Fealy N, Aitken L, du Toit E, Lo S, Baldwin I. Faster Blood Flow Rate Does Not Improve Circuit Life in Continuous Renal Replacement Therapy: A Randomized Controlled Trial. Crit Care Med. 2017;45(10):e1018-25. http://doi.org/10.1097/CCM.0000000000002568  593. Feitosa MPM, Lima EG, Abizaid AAC, Mehran R, Lopes NHM, de Assis Fischer Ramos T, et al. The safety of SGLT-2 inhibitors in diabetic patients submitted to elective percutaneous coronary intervention regarding kidney function: SAFE-PCI pilot study. Diabetology and Metabolic Syndrome. 2023;15(1). http://doi.org/10.1186/s13098-023-01107-9  594. H F. The efficacy of colon dialysis therapy with integrated traditional Chinese and Western medicine and artificial liver support system in treating patients with acute-on-chronic liver failure. Hepatol Int. 2017(1). http://doi.org/10.1007/s12072-016-9783-9  595. Feng F, Chen Y, Chen W, Yang H, Yang W, Du J, et al. [Application of a risk stratification-based model for prediction of acute kidney injury combined with hemoperfusion in patients with sepsis: a prospective, observational, pilot study]. Zhonghua Wei Zhong Bing Ji Jiu Yi Xue. 2020;32(7):814-8. http://doi.org/10.3760/cma.j.cn121430-20200326-00239  596. Feng J, Zhang S, Ai T, Wang L, Gao Y, Li W, et al. Effect of CRRT with oXiris filter on hemodynamic instability in surgical septic shock with AKI: A pilot randomized controlled trial. Int J Artif Organs. 2022;45(10):801-8. http://doi.org/10.1177/03913988221107947  597. Feng J, Zhou S, Xie Q, Huang T, Xu X, Zhou D, et al. Traditional Chinese medicine shenhuang granule in patients with severe/critical COVID-19: A randomized controlled multicenter trial. Phytomedicine. 2021;89. http://doi.org/10.1016/j.phymed.2021.153612  598. Fergany A, O'Hara J, Campbell S, Kaple K, Bonilla A. The effect of fenoldopam on renal function in solitary kidney partial nephrectomy. J Urology. 2011;185(4):e280. http://doi.org/10.1016/j.juro.2011.02.067  599. Fergany A, O'Hara J, Campbell S, Kaple K, Bonilla A, Mahboobi R. The effect of fenoldopam on renal function in solitary partial nephrectomy surgery. European Urology, Supplements. 2011;10(2):199. http://doi.org/10.1016/S1569-9056(11)60600-3  600. TW F, AX G, MM S, C R, E C, P K, et al. Association Between the Publication of the Initiating Dialysis Early and Late Trial and the Timing of Dialysis Initiation in Canada. Jama Intern Med. 2019(7). http://doi.org/10.1001/jamainternmed.2019.0489  601. Fernández Lucas M, Ruíz-Roso G, Merino JL, Sánchez R, Bouarich H, Herrero JA, et al. Initiating renal replacement therapy through incremental haemodialysis: Protocol for a randomized multicentre clinical trial. Trials. 2020;21(1). http://doi.org/10.1186/s13063-020-4058-0  602. C FR, A AH, P VV, J OG, F VC. Basiliximab (Simulect) in renal transplantation with high risk for delayed graft function. Transpl P. 2005(3). http://doi.org/10.1016/j.transproceed.2005.02.007  603. Fernández A, Royuela A, Quintás A, Zamora J. [Are IL2 receptor antagonist useful in high risk acute tubular necrosis kidney recipients?]. Nefrologia. 2007;27(5):534-6.  604. Ferrari F, Husain-Syed F, Milla P, Lorenzin A, Scudeller L, Sartori M, et al. Clinical Assessment of Continuous Hemodialysis with the Medium Cutoff EMiC ® 2 Membrane in Patients with Septic Shock. Blood Purificat. 2022;51(11):912-22. http://doi.org/10.1159/000522321  605. Ferrario F, Barone MT, Landoni G, Genderini A, Heidemperger M, Trezzi M, et al. Acetylcysteine and non-ionic isosmolar contrast-induced nephropathy - A randomized controlled study. Nephrol Dial Transpl. 2009;24(10):3103-7. http://doi.org/10.1093/ndt/gfp306  606. Ferraro S, Mocka S, Traverso GB. Use of adsorption in waterhouse-friderichsen syndrome due to meningococcal sepsis. Blood Purificat. 2021;50(3):435-6. http://doi.org/10.1159/000500752  607. AN F, CR F, M C, L V, J M, de Paula M, et al. Prospective randomized study comparing everolimus and mycophenolate sodium in de novo kidney transplant recipients from expanded criteria deceased donor. Transpl Int. 2019(11). http://doi.org/10.1111/tri.13478  608. AN F, PG M, CR F, SA M, BH H, MK T, et al. Concentration-controlled use of sirolimus associated with reduced exposure of cyclosporine in black recipients of primarily living renal allograft donors: 12-month results. Clin Transplant. 2005(5). http://doi.org/10.1111/j.1399-0012.2005.00331.x  609. JP F, P R, G B, C M, WB W, F Z. Blood and Urine Biomarkers Predicting Worsening Kidney Function in Patients with Type 2 Diabetes Post-Acute Coronary Syndrome: an Analysis from the EXAMINE Trial. Am J Nephrol. 2021(12). http://doi.org/10.1159/000519436  610. F F, E G, C F, G A, La Verde G, A T, et al. A new strategy to remove serum free light chains (SFLC)in patients with multiple myeloma (MM) and acute kidney injury (AKI). Nephrol Dial Transpl. 2015. http://doi.org/10.1093/ndt/gfv190.65  611. E F, U M, C R, R G, E P, E P, et al. Effects of different energy intakes on nitrogen balance in patients with acute renal failure: a pilot study. Nephrology, Dialysis, Transplantation. 2005(9). http://doi.org/10.1093/ndt/gfh956  612. Filiberto AC, Ozrazgat-Baslanti T, Loftus TJ, Peng YC, Datta S, Efron P, et al. Optimizing predictive strategies for acute kidney injury after major vascular surgery. Surgery (United States). 2021;170(1):298-303. http://doi.org/10.1016/j.surg.2021.01.030  613. G F, NJ W, DV M, AR W, J G, G T, et al. Four-year data after pediatric renal transplantation: a randomized trial of tacrolimus vs. cyclosporin microemulsion. Pediatr Transplant. 2005(4). http://doi.org/10.1111/j.1399-3046.2005.00334.x  614. Fioccola A, Edo MT, Gori A, Ursitti M, Villa G, Ricci Z, et al. Association between convective CKRT dose and SOFA score improvement in septic patients treated with an acrylonitrile based membranes: an observational study. Blood Purificat. 2022;51:41. http://doi.org/10.1159/000527376  615. Fioccola A, Gori A, Teresa Edo M, Ursitti M, Villa G, Romagnoli S. Extracorporeal blood purification performed in patients with fluid overload allows to increase net ultrafiltrations over time. Blood Purificat. 2022;51:42. http://doi.org/10.1159/000527376  616. S F, BS S, WA W, NE M. Randomized Controlled Trial of Subcutaneous Epoetin Alfa-epbx Versus Epoetin Alfa in End-Stage Kidney Disease. Kidney Int Rep. 2019(9). http://doi.org/10.1016/j.ekir.2019.05.010  617. SM F, D G, C M, J F, V K, B M, et al. Kidney transplantation without calcineurin inhibitor drugs: a prospective, randomized trial of sirolimus versus cyclosporine. Transplantation. 2002(8). http://doi.org/10.1097/00007890-200210270-00002  618. JN F, DJ T, NA P, JW M, TR S, PK B, et al. A randomized, prospective comparison of transition to sirolimus-based CNI-minimization or withdrawal in African American kidney transplant recipients. Clin Transplant. 2016(5). http://doi.org/10.1111/ctr.12718  619. Fleuren L, Hoeijmakers A, Fornasa M, Roggeveen L, Guo T, Thoral PJ, et al. Richt dose, right now: Machine learning to predict creatinine values in intensive care patients. Intens Care Med Exp. 2019;7. http://doi.org/10.1186/s40635-019-0265-y  620. S F, T B, B B, A C, D C, G G, et al. Efficacy and Safety Outcomes of Extended Criteria Donor Kidneys by Subtype: subgroup Analysis of BENEFIT-EXT at 7 Years After Transplant. Am J Transplant. 2017(1). http://doi.org/10.1111/ajt.13886  621. Fomin A, Mitina T, Belousov K. Effectiveness of hemadsorption in reducing free light chains in patients with multiple myeloma. Hemasphere. 2020;4:939. http://doi.org/10.1097/HS9.0000000000000404  622. Fong KY, Low CHX, Chan YH, Ho KW, Keh YS, Chin CT, et al. Role of Invasive Strategy for Non–ST-Elevation Myocardial Infarction in Patients With Chronic Kidney Disease: A Systematic Review and Meta-Analysis. Am J Cardiol. 2023;205:369-78. http://doi.org/10.1016/j.amjcard.2023.07.178  623. Forni LG. Blood Purification Studies in the ICU: What Endpoints Should We Use? Blood Purificat. 2022;51(12):990-6. http://doi.org/10.1159/000523761  624. M F, H A, SA H, M H, M M, M B, et al. Lack of renal protection of ultrafiltration during cardiac surgery: a randomized clinical trial. J Cardiovasc Surg. 2014(3).  625. BJ F, ALH P, N Z, S A, L B, VR D, et al. A Randomized Trial of a Multicomponent Intervention to Promote Medication Adherence: the Teen Adherence in Kidney Transplant Effectiveness of Intervention Trial (TAKE-IT). Am J Kidney Dis. 2018(1). http://doi.org/10.1053/j.ajkd.2017.12.012  626. D F, L J, Y L, A T, M L, MO J, et al. Acute leptin regulation in end-stage renal failure: the role of growth hormone and IGF-1. Kidney Int. 1998(3). http://doi.org/10.1046/j.1523-1755.1998.00041.x  627. França AF, BAstos LSB, Rocha ER, Maccariello EM, Lapa E Silva JRLS, Salluh JIFS. Clinical profile and outcomes of patients with COVID-19 respiratory failure requiring renal replacement therapy: a machine learning analysis of 13,576 patients. Crit Care. 2023;27. http://doi.org/10.1186/s13054-023-04377-x  628. França ARM, Rocha E, Bastos LSL, Bozza FA, Kurtz P, Maccariello E, et al. Development and validation of a machine learning model to predict the use of renal replacement therapy in 14,374 patients with COVID-19. J Crit Care. 2024;80. http://doi.org/10.1016/j.jcrc.2023.154480  629. Franca A, Bastos L, Rocha E, Maccarielo E, Lapa ESJR, Salluh J. Clinical profile and outcomes of 13,575 patients with COVID19 respiratory failure requiring Renal Replacement Therapy: A Machine Learning analysis. Intens Care Med Exp. 2022;10. http://doi.org/10.1186/s40635-022-00468-1  630. H F, D W, V L, L K, WG D, U K, et al. Simultaneous hemodialysis during coronary angiography fails to prevent radiocontrast-induced nephropathy in chronic renal failure. Clin Nephrol. 2003(3). http://doi.org/10.5414/cnp60176  631. W FP, J V, R E, L D, CV P. Clevidipine for severe hypertension in patients with renal dysfunction: a VELOCITY trial analysis. Blood Pressure. Supplement. 2011(Suppl 1). http://doi.org/10.3109/08037051.2010.539317  632. Franklin SC, Moulton M, Sicard GA, Hammerman MR, Miller SB. Insulin-like growth factor I preserves renal function postoperatively. American Journal of Physiology - Renal Physiology. 1997;272(2 41-2):F257-9. http://doi.org/10.1152/ajprenal.1997.272.2.f257  633. Frei U, Harms A, Bakovic-Alt R, Pichlmayr R, Koch KM. Calcium channel blockers for kidney protection. J Cardiovasc Pharm. 1990;16(SUPPL. 6):S11-5.  634. French LK, McKeown NJ, Hendrickson RG. Cefepime induced neurotoxicity. Clin Toxicol. 2011;49(6):564. http://doi.org/10.3109/15563650.2011.598695  635. Freund A, van Royen N, Kern KB, Jobs A, Thiele H, Lemkes JS, et al. Early coronary angiography in patients after out-of-hospital cardiac arrest without ST-segment elevation: Meta-analysis of randomized controlled trials. Catheter Cardio Inte. 2022;100(3):330-7. http://doi.org/10.1002/ccd.30355  636. Friedrich JO, Wald R, Bagshaw SM, Burns KEA, Adhikari NKJ. Hemofiltration compared to hemodialysis for acute kidney injury: systematic review and meta-analysis. Crit Care. 2012;16(4). http://doi.org/10.1186/cc11458  637. S F, W A, B N, F V, B B, K B, et al. Sotrastaurin, a novel small molecule inhibiting protein-kinase C: randomized phase II study in renal transplant recipients. Am J Transplant. 2011(7). http://doi.org/10.1111/j.1600-6143.2011.03538.x  638. Froghi F, Gopalan V, Anastasiou Z, Koti R, Gurusamy K, Eastgate C, et al. Effect of post-operative goal-directed fluid therapy (GDFT) on organ function after orthotopic liver transplantation: Secondary outcome analysis of the COLT randomised control trial. Int J Surg. 2022;99:106265. http://doi.org/10.1016/j.ijsu.2022.106265  639. P F, XQ H, AH Y, G Y, XB M, RL C. Effects of salvianolate combined with alprostadil and reduced glutathione on progression of chronic renal failure in patients with chronic kidney diseases: a long-term randomized controlled trial. Zhong XI Yi Jie He Xue Bao [Journal of Chinese Integrative Medicine]. 2012(6). http://doi.org/10.3736/jcim20120607  640. RA F, MF P, AN P, GE M, MA H. Impact of telmisartan on glomerular filtration in laparoscopic surgery. A double blinded randomised controlled study. Cir Cir. 2017(1). http://doi.org/10.1016/j.circir.2016.05.010  641. Fuernau G, Poess J, Denks D, Desch S, Heine GH, Eitel I, et al. Fibroblast growth factor 23 in acute myocardial infarction complicated by cardiogenic shock-a biomarker substudy of the IABP-shock ii-trial. Circulation. 2013;128(22).  642. Fuernau G, Traeder F, Lele S, Rajapurkar MM, Mukhopadhyay B, Desch S, et al. Catalytic iron in acute myocardial infarction complicated by cardiogenic shock: A biomarker substudy of the IABP-SHOCK II-trial. J Am Coll Cardiol. 2015;65(10):A10.  643. Fuiano G, Di Filippo S, Memoli B, Cioffi M, Caglioti A, Mazza G. Guidelines for dialysis. Replacement therapy for acute renal failure in critically ill patients. Giornale Italiano Di Nefrologia : Organo Ufficiale Della Società Italiana Di Nefrologia. 2004;21 Suppl 28:S1-10.  644. T F, S F, Y K, Y A, T K, M F, et al. A randomized, open-label trial of edoxaban in Japanese patients with severe renal impairment undergoing lower-limb orthopedic surgery. Thromb J. 2015(1). http://doi.org/10.1186/s12959-014-0034-9  645. Funke B, Jackson KE, Siew ED, Saunders CT, Blume JD, Self WH, et al. Intravenous crystalloid composition does not affect urinary kidney injury molecule-1 levels among critically ill adults. Am J Resp Crit Care. 2019;199(9).  646. AO G, BD K, Van Buren C, SL S, J S, JF N. Comparison of sirolimus plus tacrolimus versus sirolimus plus cyclosporine in high-risk renal allograft recipients: results from an open-label, randomized trial. Transplantation. 2008(9). http://doi.org/10.1097/TP.0b013e318187bab0  647. AO G, LW M, RR A, ES W, J P, F S, et al. Acute rejection characteristics from a prospective, randomized, double-blind, placebo-controlled multicenter trial of early corticosteroid withdrawal. Transplantation. 2013(4). http://doi.org/10.1097/TP.0b013e3182777efb  648. DP G, JT C, LC M, P B, AL B. Continuous peritoneal dialysis compared with daily hemodialysis in patients with acute kidney injury. Periton Dialysis Int. 2009.  649. Gabriel A, Müller E, Tarnow J. [Treatment of acute renal failure--concepts and controversies. 2. Extracorporeal renal replacement and peritoneal dialysis]. Anasth Intensiv Notf. 2001;36(4):195-204. http://doi.org/10.1055/s-2001-12746  650. Gabriel A, Müller E, Tarnow J. Rational pharmacotheraphy and experimental strategies in the treatment of acute renal failure: Part II - Acute renal failure: Extracorporal renal replacement therapy and peritoneal dialysis. Anasth Intensiv Notf. 2001;36(4):195-204. http://doi.org/10.1055/s-2001-12746  651. Gabriel DP, Caramori JT, Martim LC, Barretti P, Balbi AL. High volume peritoneal dialysis vs daily hemodialysis: A randomized, controlled trial in patients with acute kidney injury. Kidney Int. 2008;73(SUPPL. 108):S87-93. http://doi.org/10.1038/sj.ki.5002608  652. E G, T C, J B, C C, M B, E C, et al. PRODIG (Prevention of new onset diabetes after transplantation by a short term treatment of Vildagliptin in the early renal post-transplant period) study: study protocol for a randomized controlled study. Trials. 2019(1). http://doi.org/10.1186/s13063-019-3392-6  653. M G, A C, R B, S F, D G, J L, et al. Long-term survival and dialysis dependency following acute kidney injury in intensive care: extended follow-up of a randomized controlled trial. Plos Med. 2014(2). http://doi.org/10.1371/journal.pmed.1001601  654. Gallagher M, Bellomo R, Cass A, Cole L, Finfer S, Lee J, et al. Fluid balance and patient outcomes in AKI: Analysis of the renal study participants. Nephrology. 2011;16:57. http://doi.org/10.1111/j.1440-1797.2011.01491.x  655. Galli V, Cecchin E, Trevisanello A, Trento L, Galuppi E, Scarsini R, et al. IMPACT OF RENAL DENERVATION ON BLOOD PRESSURE CONTROL IN A REAL POPULATION WITH MODERATE-TO-SEVERE CHRONIC KIDNEY DISEASE. G Ital Cardiol. 2023;24(10):e10-1.  656. Gandhi A, Husain M, Salhiyyah K, Raja SG. Does perioperative furosemide usage reduce the need for renal replacement therapy in cardiac surgery patients? Interact Cardiov Th. 2012;15(4):750-5. http://doi.org/10.1093/icvts/ivs208  657. J G, F W, Y W, D J, L T, K P. A mode of CVVH with regional citrate anticoagulation compared to no anticoagulation for acute kidney injury patients at high risk of bleeding. Sci Rep-Uk. 2019(1). http://doi.org/10.1038/s41598-019-42916-1  658. Gao W, Wang J, Zhou L, Luo Q, Lao Y, Lyu H, et al. Prediction of acute kidney injury in ICU with gradient boosting decision tree algorithms. Comput Biol Med. 2022;140. http://doi.org/10.1016/j.compbiomed.2021.105097  659. Garcés EO, Victorino JA, Thomé FS, Röhsig LM, Dornelles E, Louzada M, et al. Enoxaparin versus unfractioned heparin as anticoagulant for continuous venovenous hemodialysis: A randomized open-label trial. Renal Failure. 2010;32(3):320-7. http://doi.org/10.3109/08860221003606281  660. IG G, M R, AR M, de Soto LM, LD G, JM V, et al. A randomized multicenter clinical trial to evaluate the efficacy of melatonin in the prophylaxis of SARS-CoV-2 infection in high-risk contacts (MeCOVID Trial): a structured summary of a study protocol for a randomised controlled trial. Trials. 2020(1). http://doi.org/10.1186/s13063-020-04436-6  661. R G, PG P, CR F, SI P, LA S, MF F, et al. Conversion from azathioprine to mycophenolate mofetil followed by calcineurin inhibitor minimization or elimination in patients with chronic allograft dysfunction. Transpl P. 2006(9). http://doi.org/10.1016/j.transproceed.2006.10.001  662. S G, DL B, M G, H J, J K, PM P, et al. Strategies to Reduce Acute Kidney Injury and Improve Clinical Outcomes Following Percutaneous Coronary Intervention: a Subgroup Analysis of the PRESERVE Trial. JACC. Cardiovascular Interventions. 2018(22). http://doi.org/10.1016/j.jcin.2018.07.044  663. VD G, DB C, RT G, RL C, HH C, M A, et al. Randomized trial of early corticosteroid reduction vs. regular-dose corticosteroid maintenance in combination with tacrolimus and mycophenolate mofetil in living donor kidney transplant recipients: the Brazilian CORRETA trial. Clin Transplant. 2010(4). http://doi.org/10.1111/j.1399-0012.2009.01185.x  664. García-Fernández N, Lavilla FJ, Rocha E, Purroy A. Haemostatic changes in systemic inflammatory response syndrome during continuous renal replacement therapy. J Nephrol. 2000;13(4):282-9.  665. Garg AX, Al-Jaishi AA, Dixon SN, Sontrop JM, Anderson SJ, Bagga A, et al. Personalised cooler dialysate for patients receiving maintenance haemodialysis (MyTEMP): a pragmatic, cluster-randomised trial. The Lancet. 2022;400(10364):1693-703. http://doi.org/10.1016/S0140-6736(22)01805-0  666. Garg AX, Vincent J, Cuerden M, Parikh C, Devereaux PJ, Teoh K, et al. Steroids in caRdiac Surgery (SIRS) trial: Acute kidney injury substudy protocol of an international randomised controlled trial. Bmj Open. 2014;4(3). http://doi.org/10.1136/bmjopen-2014-004842  667. G G, A S, A C, S S, P S, MR S, et al. Acute effects of peritoneal dialysis with dialysates containing dextrose or dextrose and amino acids on muscle protein turnover in patients with chronic renal failure. Journal of the American Society of Nephrology : JASN. 2001(3). http://doi.org/10.1681/ASN.V123557  668. P G, H S, F M, D C, J L, G N, et al. Results of the multicenter, prospective, randomised STRENGTH (Study To evaluate the use of RENalGuard to proTect patients at High risk of acute kidney injury) study. Eur Heart J. 2020(SUPPL 2). http://doi.org/10.1093/ehjci/ehaa946.2555  669. F G, M O, ME H, D M, L C, R R, et al. The (doreMIFA) dose response multicenter investigation fluid assesment: fluid accumulation and CRRT initiation. Blood Purificat. 2015(1). http://doi.org/10.1159/000431262  670. Gastaldello K, Melot C, Kahn RJ, Vanherweghem JL, Vincent JL, Tielemans C. Comparison of cellulose diacetate and polysulfone membranes in the outcome of acute renal failure. A prospective randomized study. Nephrol Dial Transpl. 2000;15(2):224-30.  671. Gaudry S, Grolleau F, Barbar S, Martin-Lefevre L, Pons B, Boulet É, et al. Continuous renal replacement therapy versus intermittent hemodialysis as first modality for renal replacement therapy in severe acute kidney injury: a secondary analysis of AKIKI and IDEAL-ICU studies. Crit Care. 2022;26(1). http://doi.org/10.1186/s13054-022-03955-9  672. Gaviria SP, Zapata N, Villa P, Agudelo CA, Molina FJ, González MA, et al. HIV/AIDS infection in critical care: epidemiological profile and risk factors for mortality in a Colombian cohort. J Infect Dev Countr. 2023;17(1):102-10. http://doi.org/10.3855/jidc.15859  673. Gawarammana IB, Kularatne SA, Dissanayake WP, Kumarasiri RPV, Senanayake N, Ariyasena H. Parallel infusion of hydrocortisone ± chlorpheniramine bolus injection to prevent acute adverse reactions to antivenom for snakebites: A randomised, double-blind, placebo-controlled study. Med J Australia. 2004;180(1):20-3. http://doi.org/10.5694/j.1326-5377.2004.tb05768.x  674. Gawrieh S, Dasarathy S, Tu W, Kamath PS, Chalasani NP, McClain CJ, et al. Randomized trial of anakinra plus zinc vs. prednisone for severe alcohol-associated hepatitis. J Hepatol. 2024;80(5):684-93. http://doi.org/10.1016/j.jhep.2024.01.031  675. Gayle L, Otuonye G, Sittol R, Imran M, Williams K, Fein D. SEVERE ACUTE KIDNEY INJURY IN COVID-19: NOT IRRECOVERABLE. Chest. 2020;158(4):A793. http://doi.org/10.1016/j.chest.2020.08.738  676. JJ G, G C, G G, J S, L H, D R, et al. Single-centre study of 628 adult, primary kidney transplant recipients showing no unfavourable effect of new-onset diabetes after transplant. Diabetologia. 2015(2). http://doi.org/10.1007/s00125-014-3428-0  677. Ge C, Deng F, Chen W, Ye Z, Zhang L, Ai Y, et al. Machine learning for early prediction of sepsis-associated acute brain injury. Front Med-Lausanne. 2022;9. http://doi.org/10.3389/fmed.2022.962027  678. MA G, MH C, van Heurn EL, van den Berg-Loonen EP, CJ P, van Hooff JP. High rejection rate during calcineurin inhibitor-free and early steroid withdrawal immunosuppression in renal transplantation. Transplantation. 2006(9). http://doi.org/10.1097/01.tp.0000232688.76018.19  679. JL G, C K, RH A, LL S. The Role of Thromboelastography in Pediatric Patients with Sinusoidal Obstructive Syndrome Receiving Defibrotide. Biol Blood Marrow Tr. 2017(4). http://doi.org/10.1016/j.bbmt.2017.01.074  680. Genga KR, Russell JA. Update of Sepsis in the Intensive Care Unit. J Innate Immun. 2017;9(5):441-55. http://doi.org/10.1159/000477419  681. George J, Varma S, Kumar S, Thomas J, Gopi S, Pisharody R. Comparing continuous venovenous hemodiafiltration and peritoneal dialysis in critically ill patients with acute kidney injury: A pilot study. Periton Dialysis Int. 2011;31(4):422-9. http://doi.org/10.3747/pdi.2009.00231  682. Gernone G, Detomaso F, Partipilo F, Montemurro M, Procino F, Diele C. POS-853 AKI due to COVID-19 disease requiring Renal Replacement Therapy: role of Expanded HaemoDialisys (HDx) on inflammation and outcome. Kidney Int Rep. 2022;7(2):S368-9. http://doi.org/10.1016/j.ekir.2022.01.891  683. Ghadimi K, Cappiello JL, Wright MC, Levy JH, Bryner BS, Devore AD, et al. Inhaled Epoprostenol Compared With Nitric Oxide for Right Ventricular Support After Major Cardiac Surgery. Circulation. 2023;148(17):1316-29. http://doi.org/10.1161/CIRCULATIONAHA.122.062464  684. Ghahramani N, Shadrou S, Hollenbeak C. A systematic review of continuous renal replacement therapy and intermittent haemodialysis in management of patients with acute renal failure. Nephrology. 2008;13(7):570-8. http://doi.org/10.1111/j.1440-1797.2008.00966.x  685. N G, A R, S B, S B, S B, M M, et al. Atorvastatin and Aspirin as Adjuvant Therapy in Patients with SARS-CoV-2 Infection: a structured summary of a study protocol for a randomised controlled trial. Trials. 2020(1). http://doi.org/10.1186/s13063-020-04840-y  686. Ghorbani A, Masoumi K, Forouzan A, Rahmani AH, Rahim F, Taherinezhad Taeybi B, et al. Effect of pulse therapy with glucocorticoids and cyclophosphamide in patients with paraquat poisoning. Hong Kong J Emerg Me. 2015;22(4):235-40. http://doi.org/10.1177/102490791502200405  687. Gianello P, Carlier M, Jamart J, Hulhoven R, Bernheim J, Bernard A, et al. Effect of 1-28 alpha-h atrial natriuretic peptide on acute renal failure in cadaveric renal transplantation. Clin Transplant. 1995;9(6):481-9.  688. ML G, AM T, J H, A E, PM H, DJ A, et al. Radiotherapy plus cetuximab or cisplatin in human papillomavirus-positive oropharyngeal cancer (NRG Oncology RTOG 1016): a randomised, multicentre, non-inferiority trial. Lancet (London, England). 2019(10166). http://doi.org/10.1016/S0140-6736(18)32779-X  689. Giovini M, Barbera M, Antonucci E. Can dexmedetomidine reduce the incidence of AKI? Intens Care Med Exp. 2020;8(SUPPL 2). http://doi.org/10.1186/s40635-020-00354-8  690. S G, L F, D D, Le Meur Y, C M, B M, et al. EPURE Transplant (Eplerenone in Patients Undergoing Renal Transplant) study: study protocol for a randomized controlled trial. Trials. 2018(1). http://doi.org/10.1186/s13063-018-2956-1  691. Göcze I, Jauch D, Götz M, Kennedy P, Graf BM, Banas B, et al. Biomarker-guided intervention to prevent acute kidney injury after major surgery: The prospective randomized BigpAK study. Intens Care Med Exp. 2017;5(2). http://doi.org/10.1186/s40635-017-0151-4  692. Göcze I, Jauch D, Götz M, Kennedy P, Jung B, Zeman F, et al. Biomarker-guided Intervention to Prevent Acute Kidney Injury after Major Surgery. Ann Surg. 2018;267(6):1013-20. http://doi.org/10.1097/SLA.0000000000002485  693. G G, JJ L, G J, A S, De Castro V, P C. Safety of HES 130/0.4 (Voluven(R)) in patients with preoperative renal dysfunction undergoing abdominal aortic surgery: a prospective, randomized, controlled, parallel-group multicentre trial. Eur J Anaesth. 2008(12). http://doi.org/10.1017/S026502150800447X  694. WC G, MA P, JA P, C M, N T, ML F, et al. A prospective, randomized, clinical trial of intraoperative versus postoperative Thymoglobulin in adult cadaveric renal transplant recipients. Transplantation. 2003(5). http://doi.org/10.1097/01.TP.0000081042.67285.91  695. M G, de Vinuesa SG, B Q, E V, C B, E M, et al. Aspirin for Primary Prevention of Cardiovascular Disease and Renal Disease Progression in Chronic Kidney Disease Patients: a Multicenter Randomized Clinical Trial (AASER Study). Cardiovascular Drugs and Therapy / Sponsored by the International Society of Cardiovascular Pharmacotherapy. 2018(3). http://doi.org/10.1007/s10557-018-6802-1  696. Gojo I, Walker A, Cooper M, Feldman EJ, Padmanabhan S, Baer MR, et al. Phase II study of the Cyclin-Dependent Kinase (CDK) inhibitor dinaciclib (SCH 727965) in patients with advanced acute Leukemias. Blood. 2010;116(21).  697. MH G, B D, E C, P M, SL S, MB F, et al. Effects of azathioprine withdrawal in kidney recipients with stable function two years after transplant. Clin Transplant. 1996(6 Pt 2).  698. SR G, F C, MA F, E B, A G, B C, et al. Duvelisib for Critically-Ill Patients with COVID-19 Disease: an Investigator-Initiated, Randomized, Placebo-Controlled Double-Blind Pilot Trial. Blood. 2022. http://doi.org/10.1182/blood-2022-157605  699. Goldstein SL. Fluid management in acute kidney injury. J Intensive Care Med. 2014;29(4):183-9. http://doi.org/10.1177/0885066612465816  700. Goldstein SL, Askenazi DJ, Basu RK, Selewski DT, Paden ML, Krallman KA, et al. Use of the Selective Cytopheretic Device in Critically Ill Children. Kidney Int Rep. 2021;6(3):775-84. http://doi.org/10.1016/j.ekir.2020.12.010  701. Goldstein SL, Ollberding NJ, Askenazi DJ, Basu RK, Selewski DT, Krallman KA, et al. Use of the Selective Cytopheretic Device to Support Critically Ill Children Requiring Continuous Renal Replacement Therapy: A Probable Benefit-Risk Assessment.; 2023.  702. Golla R, Kumar S, Dhibhar DP, Bhalla A, Sharma N. 0.9% saline V/S Ringer’s lactate for fluid resuscitation in adult sepsis patients in emergency medical services: An open-label randomized controlled trial. Hong Kong J Emerg Me. 2022;29(5):271-80. http://doi.org/10.1177/1024907920948983  703. Gomes C, Mendes R, Suassuna J, Soares D. WCN24-979 ULTRAPORTABLE MICROCHIP VERSUS CONVENTIONAL PIEZOELECTRIC CRYSTAL US FOR EVALUATING PULMONARY AND INFERIOR VENA CAVA PARAMETERS IN AKI PATIENTS REQUIRING RRT. Kidney Int Rep. 2024;9(4):S27-8. http://doi.org/10.1016/j.ekir.2024.02.042  704. Gonçalves AM, Gonçalves J, Marinho A. The role of vitamin d in patients with severe sars cov2 pneumonia admitted to a polyvalent intensive care unit. Prospective study on clinical and laboratory evolution and prognosis. Clin Nutr Espen. 2023;54:618. http://doi.org/10.1016/j.clnesp.2022.09.470  705. TA G, DE H, K B, JM G, FP S. Improved renal function in sirolimus-treated renal transplant patients after early cyclosporine elimination. Transplantation. 2002(11). http://doi.org/10.1097/00007890-200212150-00013  706. E G, E G, Y H, G R, MJ G, EG M, et al. Anti-CD25 monoclonal antibody sequential immunosuppressive induction therapy in renal transplants with high risk of delayed graft function. Transpl P. 2005(9). http://doi.org/10.1016/j.transproceed.2005.09.176  707. Goodall G, Lamotte M, Ramos M, Maunoury F, Pejchalova B, de Pouvourville G. Cost-effectiveness analysis of the SAPIEN 3 TAVI valve compared with surgery in intermediate-risk patients. J Med Econ. 2019;22(4):289-96. http://doi.org/10.1080/13696998.2018.1559600  708. Gordon AC, Santhakumaran S, Al-Beidh F, Orme R, Perkins GD, Singer M, et al. Levosimendan to prevent acute organ dysfunction in sepsis: the LeoPARDS RCT. Southampton (UK): NIHR Journals Library; 2018.  709. Gouda S, Govindaiah HK, Solanke PV. A study of acute renal failure in snake bite patients at Bengaluru. Indian Journal of Public Health Research and Development. 2016;7(2):189-94. http://doi.org/10.5958/0976-5506.2016.00091.7  710. Gouveia R, Bravo P, Santos C, Ramos A. Contrast-induced acute kidney injury - A review focusing on prophylactic strategies. Angiologia E Cirurgia Vascular. 2015;11(2):68-78. http://doi.org/10.1016/j.ancv.2015.01.009  711. Goyal V, Gupta P, Baj B, Garg V. A randomized comparison between pulse pressure variation and central venous pressure in patients undergoing renal transplantation. J Anaesth Clin Pharm. 2021;37(4):628-32. http://doi.org/10.4103/joacp.JOACP_23_20  712. Grams ME, Coresh J, Matsushita K, Ballew SH, Sang Y, Surapaneni A, et al. Estimated Glomerular Filtration Rate, Albuminuria, and Adverse Outcomes: An Individual-Participant Data Meta-Analysis. Jama-J Am Med Assoc. 2023;330(13):1266-77. http://doi.org/10.1001/jama.2023.17002  713. Grande L, Rimola A, Cugat E, Alvarez L, García-Valdecasas JC, Taurá P, et al. Effect of venovenous bypass on perioperative renal function in liver transplantation: results of a randomized, controlled trial. Hepatology. 1996;23(6):1418-28. http://doi.org/10.1002/hep.510230618  714. R G, A W, R T, B T, J J, M F, et al. A randomized trial to assess the impact of early steroid withdrawal on growth in pediatric renal transplantation: the TWIST study. Am J Transplant. 2010(4). http://doi.org/10.1111/j.1600-6143.2010.03047.x  715. PJ G, RH M, H K, N F, JR S. Is there an optimal time for the first cyclosporin dose in renal transplantation? Transpl Int. 1993(4). http://doi.org/10.1007/BF00337104  716. Griffin BR, Ten EP, Faubel S, Jalal D, Gallagher M, Bellomo R. Platelet Decreases following Continuous Renal Replacement Therapy Initiation as a Novel Risk Factor for Renal Nonrecovery. Blood Purificat. 2022;51(7):559-66. http://doi.org/10.1159/000517232  717. Griffith GL, Maull KI, Coleman C, Baehler RW. Acute reversible intrinsic renal failure. Surgery Gynecology and Obstetrics. 1978;146(4):631-40.  718. JM G, J A, R S, AM C, S G, E A, et al. Antilymphoblast globulin, cyclosporine, and steroids in cadaveric renal transplantation. Transplantation. 1990(6). http://doi.org/10.1097/00007890-199006000-00017  719. J G, M A, M G, R M, J M, C L, et al. Likelihood of improving or sustaining renal function over three years with belatacept or CsA: insights from the BENEFIT study. Am J Transplant. 2011(Suppl 2). http://doi.org/10.1111/j.1600-6143.2011.03534.x  720. J G, M A, M G, R M, J M, C L, et al. Improving or sustaining renal function over 3 years with belatacept or cyclosporine A (CsA): insights from the BENEFIT study. Transpl Int. 2011(Suppl 2). http://doi.org/10.1111/j.1432-2277.2011.01351.x  721. JM G, M DCR, J A, SM S, RC M, G N, et al. Safety and Efficacy Outcomes 3 Years After Switching to Belatacept From a Calcineurin Inhibitor in Kidney Transplant Recipients: results From a Phase 2 Randomized Trial. Am J Kidney Dis. 2017(5). http://doi.org/10.1053/j.ajkd.2016.09.021  722. J G, I K, P L, M M, S D, P L, et al. Renal recovery after conversion to a calcineurin inhibitor-free immunosuppression in late cardiac transplant recipients. Eur J Cardio-Thorac. 2004(3). http://doi.org/10.1016/j.ejcts.2003.11.030  723. J G, I K, U S, E S, K K, T W, et al. Mycophenolate and sirolimus as calcineurin inhibitor-free immunosuppression improves renal function better than calcineurin inhibitor-reduction in late cardiac transplant recipients with chronic renal failure. Transplantation. 2009(5). http://doi.org/10.1097/TP.0b013e3181963371  724. J G, B M, P L, L B, M M, I K, et al. Mycophenolate mofetil and sirolimus as calcineurin inhibitor-free immunosuppression for late cardiac-transplant recipients with chronic renal failure. Transplantation. 2004(4). http://doi.org/10.1097/01.tp.0000103740.98095.14  725. Grolleau F, Petit F, Gaudry S, Diard É, Quenot JP, Dreyfuss D, et al. Personalizing renal replacement therapy initiation in the intensive care unit: a reinforcement learning-based strategy with external validation on the AKIKI randomized controlled trials.; 2023.  726. Groover J, Londoño LA, Tapia-Ruano K, Iacovetta C. Extracorporeal blood purification in acutely intoxicated veterinary patients: A multicenter retrospective study (2011–2018): 54 cases. J Vet Emerg Crit Car. 2022;32(1):34-41. http://doi.org/10.1111/vec.13100  727. EA G, YJ W, N P, JD G, CF S, VA S, et al. Outcomes of coronary artery bypass grafting and reduction annuloplasty for functional ischemic mitral regurgitation: a prospective multicenter study (Randomized Evaluation of a Surgical Treatment for Off-Pump Repair of the Mitral Valve). J Thorac Cardiov Sur. 2011(1). http://doi.org/10.1016/j.jtcvs.2010.08.057  728. Grundmann F, Kubacki T, Mueller RU, Scherner M, Faust M, Becker I, et al. Pilot Trial of Dietary Restriction for Protection from Acute Kidney Injury in Cardiac Surgery. J Am Soc Nephrol. 2015;26:108A.  729. Guan M, Wang H, Tang X, Zhao Y, Wang F, Zhang L, et al. Continuous Renal Replacement Therapy With Adsorbing Filter oXiris in Acute Kidney Injury With Septic Shock: A Retrospective Observational Study. Front Med-Lausanne. 2022;9. http://doi.org/10.3389/fmed.2022.789623  730. M G, J P, C H, BK K, C N, J B, et al. Renal function, efficacy, and safety of sirolimus and mycophenolate mofetil after short-term calcineurin inhibitor-based quadruple therapy in de novo renal transplant patients: one-year analysis of a randomized multicenter trial. Transplantation. 2010(2). http://doi.org/10.1097/TP.0b013e3181e11798  731. Guerin C, Girard R, Selli JM, Perdrix JP, Ayzac L. Initial versus delayed acute renal failure in the intensive care unit. A multicenter prospective epidemiological study. Rhône-Alpes Area Study Group on Acute Renal Failure. Am J Resp Crit Care. 2000;161(3 Pt 1):872-9. http://doi.org/10.1164/ajrccm.161.3.9809066  732. Guérin C, Girard R, Selli J, Ayzac L. Intermittent versus continuous renal replacement therapy for acute renal failure in intensive care units: Results from a multicenter prospective epidemiological survey. Intens Care Med. 2002;28(10):1411-8. http://doi.org/10.1007/s00134-002-1433-0  733. B G, O M, T B, F P, JF P, J M, et al. Assessment of hemodynamic efficacy and safety of 6% hydroxyethylstarch 130/0.4 vs. 0.9% NaCl fluid replacement in patients with severe sepsis: the CRYSTMAS study. Critical Care (London, England). 2012(3). http://doi.org/10.1186/cc11358  734. Guirguis JK, Sher SJ, Doshi S. Postpartum thrombotic microangiopathy of unknown etiology: Is it too late to save the kidneys? J Am Soc Nephrol. 2020;31:111.  735. Guirguis JK, Yenebere P, Sher SJ, Kelly KJ. Lower continuous venovenous hemodialysis replacement rate and its effect on patient outcome in the COVID crisis time. J Am Soc Nephrol. 2020;31:258.  736. Gunst J, Debaveye Y, Güiza F, Dubois J, De Bruyn A, Dauwe D, et al. Tight Blood-Glucose Control without Early Parenteral Nutrition in the ICU. New Engl J Med. 2023;389(13):1180-90. http://doi.org/10.1056/NEJMoa2304855  737. Gunst J, Vanhorebeek I, Casaer MP, Hermans G, Wouters PJ, Dubois J, et al. Impact of early parenteral nutrition on metabolism and kidney injury. J Am Soc Nephrol. 2013;24(6):995-1005. http://doi.org/10.1681/ASN.2012070732  738. J G, W H, XN Y, T J, K A, MA J, et al. Short-term continuous high-volume hemofiltration on clinical outcomes of severe acute pancreatitis. Pancreas. 2014(2). http://doi.org/10.1097/01.mpa.0000437321.06857.fc  739. N G, MJ H, RD H, A B, B L, V K, et al. A pharmacokinetics and safety phase 1/1b study of oral ixazomib in patients with multiple myeloma and severe renal impairment or end-stage renal disease requiring haemodialysis. Brit J Haematol. 2016(5). http://doi.org/10.1111/bjh.14125  740. Gupta S, Wu S, Chung M, Konczal L, Patil SP. Severe Hyperammonemia and Coma in a Post-partum Patient Secondary to a Urea Cycle Disorder. Am J Resp Crit Care. 2023;207(1). http://doi.org/10.1164/ajrccm-conference.2023.C53  741. Guru PK, Singh TD, Passe M, Kashani KB, Schears GJ, Kashyap R. Derivation and Validation of a Search Algorithm to Retrospectively Identify CRRT Initiation in the ECMO Patients. Appl Clin Inform. 2016;7(2):596-603. http://doi.org/10.4338/ACI-2015-12-RA-0183  742. Haase M, Bellomo R, Devarajan P, Haase-Fielitz A. Neutrophil Gelatinase-Associated Lipocalin (NGAL) in acute kidney injury: A systematic review and meta-analysis. Intens Care Med. 2009;35:S215.  743. Haase M, Haase-Fielitz A, Plass M, Kuppe H, Hetzer R, Hannon C, et al. Prophylactic Perioperative Sodium Bicarbonate to Prevent Acute Kidney Injury Following Open Heart Surgery: A Multicenter Double-Blinded Randomized Controlled Trial. Plos Med. 2013;10(4). http://doi.org/10.1371/journal.pmed.1001426  744. Haase-Fielitz A, Mertens PR, Plaß M, Kuppe H, Hetzer R, Westerman M, et al. Urine hepcidin has additive value in ruling out cardiopulmonary bypass-associated acute kidney injury: An observational cohort study. Crit Care. 2011;15(4). http://doi.org/10.1186/cc10339  745. E H, V M, E R, R M, MS K, LL G. Cyclosporin versus tacrolimus for liver transplanted patients. Cochrane Db Syst Rev. 2006(4). http://doi.org/10.1002/14651858.CD005161.pub2  746. S H, WW L, R R, SA N. Candidemia as a cause of septic shock and multiple organ failure in nonimmunocompromised patients. Crit Care Med. 2002(8). http://doi.org/10.1097/00003246-200208000-00023  747. RW H, AJ F, YI W, L F, DK H, A D, et al. Catabolism in Critical Illness: a Reanalysis of the REducing Deaths due to OXidative Stress (REDOXS) Trial. Crit Care Med. 2022(7). http://doi.org/10.1097/CCM.0000000000005499  748. Haines RW, Kirwan CJ, Prowle JR. Continuous renal replacement therapy: individualization of the prescription. Curr Opin Crit Care. 2018;24(6):443-9. http://doi.org/10.1097/MCC.0000000000000546  749. LA H, JP A, JT F, A R, JL V, EA O, et al. High lactate levels are predictors of major complications after cardiac surgery. J Thorac Cardiov Sur. 2013(2). http://doi.org/10.1016/j.jtcvs.2013.02.003  750. Hakeam HA, Sarkhi KA, Iansavichene A. Tigecycline and Hypoglycemia, When and How? J Pharm Technol. 2024;40(1):37-44. http://doi.org/10.1177/87551225231211737  751. Halderman AK, Kandarpa M. AKI induced by oral semaglutide leading to metformin-induced lactic acidosis. J Am Soc Nephrol. 2021;32:139.  752. MA H, OA G, Y M, AM N, AF A, M E, et al. Extended Efficacy of Low-Dose Valganciclovir for Prevention of Cytomegalovirus Disease in Intermediate-Risk Kidney Transplant Recipients: two-Year Follow-Up. Exp Clin Transplant. 2019(3). http://doi.org/10.6002/ect.2018.0020  753. J-M H, D J, C C, G C, B D, J-P F, et al. Blood pressure and proteinuria control remains a challenge in patients with type 2 diabetes mellitus and chronic kidney disease: experience from the prospective observational ALICE-PROTECT study. Bmc Nephrol. 2016(1). http://doi.org/10.1186/s12882-016-0336-1  754. Hall BM, Tiller DJ, Duggin GG, Horvath JS, Farnsworth A, May J, et al. Post-transplant acute renal failure in cadaver renal recipients treated with cyclosporine. Kidney Int. 1985;28(2):178-86. http://doi.org/10.1038/ki.1985.138  755. Hambley B, Gehrie E, Webster J, Showel MM, Gondek LP, Dalton WB, et al. Coagulopathy, Hypoxemia, and Mortality Outcomes in Newly Diagnosed Acute Myeloid Leukemia with Hyperleukocytosis Treated with Large Volume Leukapheresis. Blood. 2019;134:3841. http://doi.org/10.1182/blood-2019-123229  756. S H, V K, D S, D J, RD B, M B, et al. Single-center, real-world experience with granulocyte colony-stimulating factor for management of leukopenia following kidney transplantation. Clin Transplant. 2019(6). http://doi.org/10.1111/ctr.13541  757. DJ H, JB P, YS K, SJ K, J H, HC K, et al. A 39-month follow-up study to evaluate the safety and efficacy in kidney transplant recipients treated with modified-release tacrolimus (FK506E)-based immunosuppression regimen. Transpl P. 2012(1). http://doi.org/10.1016/j.transproceed.2011.12.070  758. Han H, Zachariah T, Rosen RJ, Santoriello D. An unusual presentation of type 1 cryoglobulinemic gn in monoclonal gammopathy of undetermined significance (MGUS). J Am Soc Nephrol. 2021;32:138. http://doi.org/10.1681/asn.2020030323  759. Hang C, Liu LJ, Huang ZY, Zhu JL, Zhou BC, Li XZ. Optimal indicator for changing the filter during the continuous renal replacement therapy in intensive care unit patients with acute kidney injury: A crossover randomized trial. World J Emerg Med. 2022;13(3):136-41. http://doi.org/10.5847/WJEM.J.1920-8642.2022.046  760. S H, R B, D S, E-O E, P T, T S, et al. Molecular adsorbent recirculating system (mars) effectively replaces hepatic function in severe acute liver failure. Am J Transplant. 2017. http://doi.org/10.1111/ajt.14306  761. Hansen TG. Propofol infusion syndrome in critically ill children: An update. Acta Anaesth Scand. 2009;53:5-6. http://doi.org/10.1111/j.1399-6576.2009.02001.x  762. Hanson G, Moist L. Acute renal failure in the ICU: Assessing the utility of continuous renal replacement. J Crit Care. 2003;18(1):48-51. http://doi.org/10.1053/jcrc.2003.YJCRC10  763. DW H, MD J, SK S, CS M, TM R, SM M, et al. Induction immunosuppression with antilymphocyte globulin or OKT3 in cadaver kidney transplantation. Results of a single institution prospective randomized trial. Transplantation. 1994(3). http://doi.org/10.1097/00007890-199402150-00011  764. KL H, DC B, MA S. Rabbit antithymocyte globulin is more beneficial in standard kidney than in extended donor recipients. Transplantation. 2009(9). http://doi.org/10.1097/TP.0b013e3181a2475f  765. Harel Z, Wald R, Bargman JM, Mamdani M, Etchells E, Garg AX, et al. Nephrologist follow-up improves all-cause mortality of severe acute kidney injury survivors. Kidney Int. 2013;83(5):901-8. http://doi.org/10.1038/ki.2012.451  766. Harenski K. Adjuvant sepsis therapy in renal failure. Inflamm Res. 2010;59:s63. http://doi.org/10.1007/s00011-010-0170-z  767. Hassanpour R, Ziaie S, Kobarfard F, Kouchek M, Miri M, Ahmadi Koomleh A, et al. Evaluation of pharmacokinetic and pharmacodynamic parameters of meropenem in critically ill patients with acute kidney disease. Eur J Clin Pharmacol. 2021;77(6):831-40. http://doi.org/10.1007/s00228-020-03062-0  768. R H, P H, P J, L B, J E, MJ L, et al. Alemtuzumab-based induction treatment versus basiliximab-based induction treatment in kidney transplantation (the 3C Study): a randomised trial. Lancet (London, England). 2014(9955). http://doi.org/10.1016/S0140-6736(14)61095-3  769. S H, M G, R W, J J, C M, E E, et al. Blood pressure response to acute and chronic exercise in chronic kidney disease. Nephrology (Carlton, Vic.). 2017(1). http://doi.org/10.1111/nep.12730  770. D H, M C, B G, S O, D H, F S, et al. Preconditioning Shields Against Vascular Events in Surgery (SAVES), a multicentre feasibility trial of preconditioning against adverse events in major vascular surgery: study protocol for a randomised control trial. Trials. 2015. http://doi.org/10.1186/s13063-015-0678-1  771. M H, A L, N N, LM S. Impact of early quantitative morbidity on 1-year outcomes in coronary artery bypass graft surgery. Interact Cardiov Th. 2022(4). http://doi.org/10.1093/icvts/ivab316  772. Hébert M, Lamy A, Noiseux N, Stevens L. EARLY MORBIDITY IN ON-PUMP VS. OFF-PUMP CORONARY ARTERY BYPASS GRAFT SURGERY - A QUANTITATIVE MORBIDITY ANALYSIS OF THE CORONARY TRIAL. Can J Cardiol. 2020;36(10):S94-5. http://doi.org/10.1016/j.cjca.2020.07.186  773. Heering P, Ivens K, Aker S, Grabensee B. Distal tubular acidosis induced by FK506. Clin Transplant. 1998;12(5):465-71.  774. Heerspink HJL, Furtado RHM, Berwanger O, Koch GG, Martinez F, Mukhtar O, et al. Dapagliflozin and Kidney Outcomes in Hospitalized Patients with COVID-19 Infection: An Analysis of the DARE-19 Randomized Controlled Trial. Clin J Am Soc Nephro. 2022;17(5):643-54. http://doi.org/10.2215/CJN.14231021  775. Hein OV, Staegemann M, Wagner D, Von Heymann C, Martin M, Morgera S, et al. Torsemide versus furosemide after continuous renal replacement therapy due to acute renal failure in cardiac surgery patients. Renal Failure. 2005;27(4):385-92. http://doi.org/10.1081/JDI-65298  776. Hein OV, von Heymann C, Diehl T, Ziemer S, Ronco C, Morgera S, et al. Intermittent hirudin versus continuous heparin for anticoagulation in continuous renal replacement therapy. Renal Failure. 2004;26(3):297-303. http://doi.org/10.1081/jdi-120039529  777. Hene IZ, Wiertz NR, Van Schilfgaarde M, Wester JPJ, Leyte A, Oudemans-Van Straaten HM. Nadroparin anticoagulation in continuous venovenous hemofiltration (CVVH): Kinetics and extracorporeal removal. Intens Care Med. 2009;35:S44.  778. S H, Y P, N L, Y K, YN K, HS S, et al. Lack of Efficacy and Safety of Eculizumab for Treatment of Antibody-Mediated Rejection Following Renal Transplantation. Transpl P. 2022(8). http://doi.org/10.1016/j.transproceed.2022.08.008  779. Herget-Rosenthal S, Marggraf G, Hüsing J, Göring F, Pietruck F, Janssen O, et al. Early detection of acute renal failure by serum cystatin C. Kidney Int. 2004;66(3):1115-22. http://doi.org/10.1111/j.1523-1755.2004.00861.x  780. Hermine O, Mariette X, Tharaux PL, Resche-Rigon M, Porcher R, Ravaud P. Effect of Tocilizumab vs Usual Care in Adults Hospitalized with COVID-19 and Moderate or Severe Pneumonia: A Randomized Clinical Trial. Jama Intern Med. 2021;181(1):32-40. http://doi.org/10.1001/jamainternmed.2020.6820  781. Hermite L, Quenot JP, Nadji A, Barbar SD, Charles PE, Hamet M, et al. Sodium citrate versus saline catheter locks for non-tunneled hemodialysis central venous catheters in critically ill adults: A randomized controlled trial. Intens Care Med. 2012;38(2):279-85. http://doi.org/10.1007/s00134-011-2422-y  782. D H, T V, E S, V L, P R, A C, et al. Treatment of early borderline lesions in low immunological risk kidney transplant patients: a Spanish multicenter, randomized, controlled parallel-group study protocol: the TRAINING study. Bmc Nephrol. 2022(1). http://doi.org/10.1186/s12882-022-02989-z  783. Hernández D, Lacalzada J, Salido E, Linares J, Barragán A, Lorenzo V, et al. Regression of left ventricular hypertrophy by lisinopril after renal transplantation: role of ACE gene polymorphism. Kidney Int. 2000;58(2):889-97. http://doi.org/10.1046/j.1523-1755.2000.00239.x  784. Herrera-Gutiérrez ME, Seller-Pérez G, Lebrón-Gallardo M, De La Cruz-Cortés JP, González-Correa JA. [Use of isolated epoprostenol or associated to heparin for the maintenance of the patency of the continuous renal replacement technical circuits]. Med Intensiva. 2006;30(7):314-21. http://doi.org/10.1016/s0210-5691(06)74536-3  785. Herrington WG, Staplin N, Haynes R. Kidney disease trials for the 21st century: innovations in design and conduct. Nat Rev Nephrol. 2020;16(3):173-85. http://doi.org/10.1038/s41581-019-0212-x  786. Heung M, Bagshaw SM, House AA, Juncos LA, Piazza R, Goldstein SL. CRRTnet: a prospective, multi-national, observational study of continuous renal replacement therapy practices. Bmc Nephrol. 2017;18(1):222. http://doi.org/10.1186/s12882-017-0650-2  787. Heydari-Kamjani M, Rios J, Calderon A, Goldman Gollan Y, Machin V, Perry K. A REPORT OF MYCOTIC ANEURYSM AND RUPTURE IN VIRIDANS-GROUP STREPTOCOCCAL INFECTIVE ENDOCARDITIS OF THE NATIVE AORTIC VALVE. Chest. 2023;164(4):A2542-3. http://doi.org/10.1016/j.chest.2023.07.1694  788. Heyland DK, Patel J, Compher C, Rice TW, Bear DE, Lee ZY, et al. The effect of higher protein dosing in critically ill patients with high nutritional risk (EFFORT Protein): an international, multicentre, pragmatic, registry-based randomised trial. The Lancet. 2023;401(10376):568-76. http://doi.org/10.1016/S0140-6736(22)02469-2  789. LB H, AJ H, RA K. Medication compliance after renal transplantation. Transplantation. 1995(9).  790. DM H, JA R, JK A, WL H, KK C. Continuous Venovenous Hemofiltration is Associated with Improved Survival in Burn Patients with Shock: a Subset Analysis of a Multicenter Observational Study. Blood Purificat. 2021(4‐5). http://doi.org/10.1159/000512101  791. Hill M, Mor MK, Travis L, Ward T, Palevsky PM, Ramkumar M, et al. Renal function following fistulography in patients with advanced chronic kidney disease. Renal Failure. 2013;35(6):791-5. http://doi.org/10.3109/0886022X.2013.794432  792. Himmelfarb J, Tolkoff RN, Chandran P, Parker RA, Wingard RL, Hakim R. A multicenter comparison of dialysis membranes in the treatment of acute renal failure requiring dialysis. J Am Soc Nephrol. 1998;9(2):257-66. http://doi.org/10.1681/ASN.V92257  793. Hinden SE, Schweighauser A, Francey T. Evaluation of a novel non-surgical post-pyloric feeding technique in dogs with severe acute kidney injury. J Vet Emerg Crit Car. 2020;30(4):384-95. http://doi.org/10.1111/vec.12955  794. R H, J K, P L, E B, J M, T A, et al. Multicenter clinical trial of recombinant human insulin-like growth factor I in patients with acute renal failure. Kidney Int. 1999(6). http://doi.org/10.1046/j.1523-1755.1999.00463.x  795. P H, J H, S S, H H, T M, L S, et al. Randomized trial to assess the clinical utility of renal allograft monitoring by urine CXCL10 chemokine. Journal of the American Society of Nephrology : JASN. 2023(8). http://doi.org/10.1681/ASN.0000000000000160  796. K H, K E. Multicenter trial of carperitide in patients with renal dysfunction undergoing cardiovascular surgery. Gen Thorac Cardiovas. 2012(1). http://doi.org/10.1007/s11748-011-0846-5  797. Hjortrup PB, Haase N, Treschow F, Møller MH, Perner A. Predictive value of neutrophil gelatinase-associated lipocalin (NGAL) for use of renal replacement therapy in patients with severe sepsis. Intens Care Med. 2013;39:S215-6. http://doi.org/10.1007/s00134-013-3095-5  798. Hladunewich MA, Corrigan G, Derby GC, Ramaswamy D, Kambham N, Scandling JD, et al. A randomized, placebo-controlled trial of IGF-1 for delayed graft function: A human model to study postischemic ARF. Kidney Int. 2003;64(2):593-602. http://doi.org/10.1046/j.1523-1755.2003.00100.x  799. Hlavicka J, Straka Z, Jelinek S, Budera P, Vanek T, Maly M, et al. Off-pump versus on-pump coronary artery bypass grafting surgery in high-risk patients: PRAGUE-6 trial at 30 days and 1 year. Biomedical Papers of the Medical Faculty of the University Palacky, Olomouc, Czechoslovakia. 2016;160(2):263-70. http://doi.org/10.5507/bp.2015.059  800. J H, A S, K K, R C, De Serres S, IW G, et al. Multicentre randomised controlled trial protocol of urine CXCL10 monitoring strategy in kidney transplant recipients. Bmj Open. 2019(4). http://doi.org/10.1136/bmjopen-2018-024908  801. Hoareau GL, Beyer CA, Kashtan HW, Walker LE, Wilson C, Wishy A, et al. Improvised Field Expedient Method for Renal Replacement Therapy in a Porcine Model of Acute Kidney Injury. Disaster Med Public. 2021;15(6):741-9. http://doi.org/10.1017/dmp.2020.107  802. H H, A M, E K, K R, M J, E F. Pentoxifylline decreases the incidence of multiple organ failure in patients after major cardio-thoracic surgery. Shock (Augusta, Ga.). 1998(4). http://doi.org/10.1097/00024382-199804000-00001  803. Hoffmann H, Markewitz A, Reichert K, Jochum M, Faist E, Reichart B. Acute lung injury and renal failure in high risk patients after major cardio-thoracic surgery: Therapeutic effects of Xanthines. Zeitschrift Fur Herz-, Thorax- Und Gefasschirurgie. 1998;12(2):87-93. http://doi.org/10.1007/s003980050021  804. AJ H, P R, JG K, van Lier HJ, PJ C, RA K. Treatment of acute rejection of cadaveric renal allografts with rabbit antithymocyte globulin. Transplantation. 1982(1). http://doi.org/10.1097/00007890-198201000-00003  805. AC H, T S, de Jong RC, RA D, JW G, ED W, et al. A placebo-controlled, double-blind trial of growth hormone treatment in prepubertal children after renal transplant. Kidney International. Supplement. 1996.  806. H H, AG J, DC W, IB B, PJ C, B F, et al. Effect of fluvastatin on acute renal allograft rejection: a randomized multicenter trial. Kidney Int. 2001(5). http://doi.org/10.1046/j.1523-1755.2001.00010.x  807. AA H, RJ H, J H, van Es LA, van der Woude FJ. Late prednisone withdrawal in cyclosporine-treated kidney transplant patients: a randomized study. Journal of the American Society of Nephrology : JASN. 1997(2). http://doi.org/10.1681/ASN.V82294  808. E H, S S, R K, A N, KB L. Nitric Oxide Inhalation is Associated with Decreased Incidence of Acute Rejection after Cardiac Transplantation. J Heart Lung Transpl. 2020(4). http://doi.org/10.1016/j.healun.2020.01.915  809. Hölscher B, Heitmeyer C, Fobker M, Breithardt G, Schaefer RM, Reinecke H. Predictors for contrast media-induced nephropathy and long-term survival: prospectively assessed data from the randomized controlled Dialysis-Versus-Diuresis (DVD) trial. Can J Cardiol. 2008;24(11):845-50. http://doi.org/10.1016/s0828-282x(08)70193-4  810. Holt BG, White JJ, Kuthiala A, Fall P, Szerlip HM. Sustained low-efficiency daily dialysis with hemofiltration for acute kidney injury in the presence of sepsis. Clin Nephrol. 2008;69(1):40-6. http://doi.org/10.5414/CNP69040  811. Holtermann W, Kramer M, Lukasewitz P, Wickern VM. Comparative clinical trial with two 10% medium-molecular weight hydroxyethyl solutions of different starches in patients on continuous veno-venous haemofiltration. Anasthesiologie Und Intensivmedizin. 1995;36(10):282-6.  812. Honore PM, Jacobs R, Joannes-Boyau O, De Regt J, Boer W, De Waele E, et al. Septic AKI in ICU patients. diagnosis, pathophysiology, and treatment type, dosing, and timing: a comprehensive review of recent and future developments. Ann Intensive Care. 2011;1(1):32. http://doi.org/10.1186/2110-5820-1-32  813. Honore PM, Joannes-Boyau O, Boer W, Collin V. High-volume hemofiltration in sepsis and SIRS: Current concepts and future prospects. Blood Purificat. 2009;28(1):1-11. http://doi.org/10.1159/000210031  814. Honore PM, Joannes-Boyau O, Boer W, Janvier G, Gressens B. Acute kidney injury in the ICU: time has come for an early biomarker kit!. Acta Clin Belg. 2007;62 Suppl 2:318-21. http://doi.org/10.1179/acb.2007.072  815. Honoré PM, Joannes-Boyau O, Gressens B. Blood and plasma treatments: High-volume hemofiltration - A global view.; 2007. p. 371-86.  816. Honore PM, Joannes-Boyau O, Merson L, Boer W, Piette V, Galloy AC, et al. The big bang of hemofiltration: The beginning of a new era in the third millennium for extra-corporeal blood purification!. Int J Artif Organs. 2006;29(7):649-59. http://doi.org/10.1177/039139880602900702  817. S H, R K, J R, A P, M C, M L. Impact of resuscitation fluid bag size availability on volume of fluid administration in the intensive care unit. Acta Anaesth Scand. 2018(9). http://doi.org/10.1111/aas.13161  818. T H, R K, A D, C Z, W J, M B, et al. Pharmacokinetics of ganciclovir during continuous venovenous hemodiafiltration in critically Ill patients. Antimicrob Agents Ch. 2014(1). http://doi.org/10.1128/AAC.00892-13  819. S H, C C, C W, G O, B P, L B, et al. A Randomised Trial of Normothermic Machine Perfusion versus Static Cold Storage in Donation After Circulatory Death Renal Transplantation. Am J Transplant. 2022. http://doi.org/10.1111/ajt.17072  820. SA H, K S, C W, C C, D C, ML N. Protocol of a randomised controlled, open-label trial of ex vivo normothermic perfusion versus static cold storage in donation after circulatory death renal transplantation. Bmj Open. 2017(1). http://doi.org/10.1136/bmjopen-2016-012237  821. Hoste EAJ, Vanholder RC, Lameire NH, Roosens CDVK, Decruyenaere JMA, Blot SI, et al. No early respiratory benefit with CVVHDF in patients with acute renal failure and acute lung injury. Nephrol Dial Transpl. 2002;17(12):2153-8. http://doi.org/10.1093/ndt/17.12.2153  822. Hou X, Fu C, Zhang P, Wang X, Yi K, You T. Effect of continuous blood purification for acute renal injury after acute Stanford type A aortic dissection. National Medical Journal of China. 2021;101(5):333-8. http://doi.org/10.3760/cma.j.cn112137-20200527-01682  823. AA H, M E, K D, PP L, N M, F R, et al. Apparent low absorbers of cyclosporine microemulsion have higher requirements for tacrolimus in renal transplantation. Clin Transplant. 2007(4). http://doi.org/10.1111/j.1399-0012.2007.00680.x  824. Hovaguimian F, Schläpfer M, Beck-Schimmer B. Organ protection in allograft recipients: anesthetic strategies to reduce postoperative morbidity and mortality. Curr Opin Organ Tran. 2014;19(2):121-30. http://doi.org/10.1097/MOT.0000000000000062  825. Howitt SH, Oakley J, Caiado C, Goldstein M, Malagon I, Mccollum C, et al. A Novel Patient-Specific Model for Predicting Severe Oliguria; Development and Comparison With Kidney Disease: Improving Global Outcomes Acute Kidney Injury Classification. Crit Care Med. 2020;48(1):E18-25. http://doi.org/10.1097/CCM.0000000000004074  826. DE H, B A, T A, DC B, JS B, S B, et al. Infliximab Induction Lacks Efficacy and Increases BK Virus Infection in Deceased Donor Kidney Transplant Recipients: results of the CTOT-19 Trial. Journal of the American Society of Nephrology : JASN. 2023(1). http://doi.org/10.1681/ASN.2022040454  827. Hsu WT, Galm B, Schrank G, Lee WC, Hsu TC, Lee CC, et al. Renin-angiotensin-aldosterone system inhibition and outcome of sepsis. Pharmacoepidem Dr S. 2018;27:41-2. http://doi.org/10.1002/pds.4629  828. ZJ H, H I, R S, S K, I A. Time course of activated coagulation time at various sites during continuous haemodiafiltration using nafamostat mesilate. Intens Care Med. 1999(5). http://doi.org/10.1007/s001340050892  829. Hu J, Francesco Z, Edward AB, Chong L, Rezoagli E, Lorenzo B. Free hemoglobin ratio as a novel predictive biomarker of acute kidney injury after cardiac surgery: Secondary analysis of a randomized controlled trial. Intens Care Med Exp. 2020;8(SUPPL 2). http://doi.org/10.1186/s40635-020-00354-8  830. Hu J, Rezoagli E, Zadek F, Bittner EA, Lei C, Berra L. Free Hemoglobin Ratio as a Novel Biomarker of Acute Kidney Injury after On-Pump Cardiac Surgery: Secondary Analysis of a Randomized Controlled Trial. Anesth Analg. 2021;132(6):1548-58. http://doi.org/10.1213/ANE.0000000000005381  831. Hu P, Liang X, Chen Y, Li R, Jiang F, Li Z, et al. Development and validation of a severe a acute kidney injury risk index for chinese elderly patients with cardiac surgery. Nephrol Dial Transpl. 2012;27:ii349. http://doi.org/10.1093/ndt/gfs235  832. Hu Y, Liu K, Ho K, Riviello D, Brown J, Chang AR, et al. A Simpler Machine Learning Model for Acute Kidney Injury Risk Stratification in Hospitalized Patients. J Clin Med. 2022;11(19). http://doi.org/10.3390/jcm11195688  833. Huang CT, Wang TJ, Kuo LK, Tsai MJ, Cia CT, Chiang DH, et al. Federated machine learning for predicting acute kidney injury in critically ill patients: a multicenter study in Taiwan. Health Inf Sci Syst. 2023;11(1). http://doi.org/10.1007/s13755-023-00248-5  834. Huang CY, Guiza Grandas F, Schetz M, Gunst J, Casaer M, Van Den Berghe G, et al. Clinical prediction model for acute kidney injury recovery in the intensive care unit. Intens Care Med Exp. 2019;7. http://doi.org/10.1186/s40635-019-0265-y  835. Huang CY, Güiza Grandas F, Schetz M, Gunst J, Casaer M, Van Den Berghe G, et al. Development and internal validation of a model to predict acute kidney injury recovery at hospital discharge. Crit Care. 2020;24. http://doi.org/10.1186/s13054-020-2772-3  836. Huang H, Wu C, Shen Q, Xu H, Fang Y, Mao W. The effect of early vasopressin use on patients with septic shock: A systematic review and meta-analysis. Am J Emerg Med. 2021;48:203-8. http://doi.org/10.1016/j.ajem.2021.05.007  837. Huang JJ, Cai JZ, Zhou ZP, Liu Y, Yang ZJ, Li DZ, et al. Impact of early heparin therapy on outcomes in patients with solid malignancy associated sepsis: a marginal structural model causal analyse. Front Pharmacol. 2023;14. http://doi.org/10.3389/fphar.2023.1281235  838. Huang TM, Chen YT, Chang FC, Ko WJ, Wu KD, Wu VC. Diuretics on hospital mortality in postoperative patients receiving acute dialysis. Blood Purificat. 2009;28(4):324. http://doi.org/10.1159/000235632  839. Huei Hern KK. A complicated course of infective endocarditis. Irish J Med Sci. 2014;183(4):S176-8. http://doi.org/10.1007/s11845-014-1153-9  840. K H, F G, JH B, M G, M-S J, F L, et al. Early renal perfusion impairment quantified functional MRI is predictive for delayed renal graft function and renal outcome one year after kidney transplantation. Nephrol Dial Transpl. 2015. http://doi.org/10.1093/ndt/gfv202.3  841. K H, AA K, JH B, VD VC, M G, S W, et al. Diffusion-Weighted imaging and diffusion tensor imaging detect delayed graft function and correlate with allograft fibrosis in patients early after kidney transplantation. J Magn Reson Imaging. 2016(1). http://doi.org/10.1002/jmri.25158  842. KH H, JG L, J H, CK O, MK J, CD K, et al. De novo low-dose sirolimus versus mycophenolate mofetil in combination with extended-release tacrolimus in kidney transplant recipients: a multicentre, open-label, randomized, controlled, non-inferiority trial. Nephrology, Dialysis, Transplantation. 2017(8). http://doi.org/10.1093/ndt/gfx093  843. L H, A Z, X Z, K Z, F S. Evaluation of the therapeutic effect of hemopurification in hyperlipidemic severe acute pancreatitis. Int J Clin Exp Med. 2019(1).  844. Hummel M, Kuhn M, Bub A, Bittner H, Kleefeld D, Marxen P, et al. Urodilatin: a new peptide with beneficial effects in the postoperative therapy of cardiac transplant recipients. Clin Investig. 1992;70(8):674-82. http://doi.org/10.1007/BF00180284  845. P H, C B, I J, C K, L D, L M, et al. Oxygenated End-Hypothermic Machine Perfusion in Expanded Criteria Donor Kidney Transplant: a Randomized Clinical Trial. Jama Surg. 2021(6). http://doi.org/10.1001/jamasurg.2021.0949  846. D H, F H, S N, J S, Y Z, K W, et al. Prognostic relevance of tissue oxygen saturation in patients in the early stage of multiple organ dysfunction syndrome. Medizinische Klinik, Intensivmedizin Und Notfallmedizin. 2019(2). http://doi.org/10.1007/s00063-018-0438-6  847. Hutchison CA, Cockwell P, Reid S, Chandler K, Mead GP, Harrison J, et al. Efficient removal of immunoglobulin free light chains by hemodialysis for multiple myeloma: In vitro and in vivo studies. J Am Soc Nephrol. 2007;18(3):886-95. http://doi.org/10.1681/ASN.2006080821  848. Hutchison CA, Xiong F, Mollee P. The treatment of paraprotein-related kidney disease. Curr Opin Nephrol Hy. 2017;26(6):477-83. http://doi.org/10.1097/MNH.0000000000000369  849. M I, M B, M E, AE M. Cardiovascular risk of circulating endotoxin level in prevalent hemodialysis patients. Egypt Heart J. 2018(1). http://doi.org/10.1016/j.ehj.2017.06.003  850. Ibrahim M, Sany D, Elsaid H. Acute renal failure: Prognostic value of RIFLE Criteria in acute renal failure in early postrenal transplant phase. Hemodial Int. 2010;14(1):97. http://doi.org/10.1111/j.1542-4758.2009.00434.x  851. TA I, JD M, LJ R, JA E, B B, RM H, et al. Plasma F2-isoprostane levels are elevated in chronic hemodialysis patients. Clin Nephrol. 2002(3). http://doi.org/10.5414/cnp58190  852. Imamura R, Isaka Y, Sandoval RM, Kakuta Y, Abe T, Okumi M, et al. Systemic administration of siRNA targeting p53 protects kidneys from ischemia-reperfusion injury in experimental rat kidney transplantation model. J Urology. 2009;181(4):743.  853. N I, T S, V P, M V, M B, J W, et al. Noninterventional follow-up vs fluid bolus in RESPONSE to oliguria-The RESPONSE trial protocol and statistical analysis plan. Acta Anaesth Scand. 2020(8). http://doi.org/10.1111/aas.13599  854. Inthorn D, Storck M, Hartl WH, Zimmerer E. [Improved survival rate of postoperative renal failure caused by high volume hemofiltration]. Zbl Chir. 1991;116(16):961-8.  855. N I. The effect of curcumin in the prevention of kidney failure following heart angiography. Https://Trialsearch.Who.Int/Trial2.Aspx?TrialID=IRCT138706261256N1. 2017.  856. N I. The effect of zinc supplementation in chronic hemodialysis patients. Https://Trialsearch.Who.Int/Trial2.Aspx?TrialID=IRCT138902033777N1. 2003.  857. N I. The effect of remote ischemic preconditioning on kidney and cardiac injury after CABG. Https://Trialsearch.Who.Int/Trial2.Aspx?TrialID=IRCT138903123646N3. 2016.  858. N I. Prophylactic Dialysis in CABG Patients with Moderate Renal Failure. Https://Trialsearch.Who.Int/Trial2.Aspx?TrialID=IRCT201011065113N1. 2012.  859. N I. Effects of magnesium on insomnia. Https://Trialsearch.Who.Int/Trial2.Aspx?TrialID=IRCT201109057479N1. 2011.  860. N I. The effect of Lasix in preventing acute kidney injury. Https://Trialsearch.Who.Int/Trial2.Aspx?TrialID=IRCT201205092582N8. 2012.  861. N I. Effect of three heparinization methods of hemodialysis set on coagulation condition and dialysis efficacy in patients under hemodialysis. Https://Trialsearch.Who.Int/Trial2.Aspx?TrialID=IRCT2013021712498N1. 2013.  862. N I. The comparison of the effects of three therapeutic methods (oatmeal (Avena Sativa) , diluted vinegar sponge bath and Routine Protocol) in reducing uremic pruritus. Https://Trialsearch.Who.Int/Trial2.Aspx?TrialID=IRCT2013021912525N1. 2013.  863. N I. The Effect of aromatherapy on Sleep Quality of Patients Undergoing Hemodialysis. Https://Trialsearch.Who.Int/Trial2.Aspx?TrialID=IRCT2013042111572N2. 2013.  864. N I. Evaluation of Co-Enzyme Q10 in cardiac pre-procedural injury of ischemic heart diseases patients undergoing angioplasty. Https://Trialsearch.Who.Int/Trial2.Aspx?TrialID=IRCT201311278307N3. 2014.  865. N I. Evaluation of pentoxifylline in pre-procedural cardiac injury following angioplasty in patients with ischemic heart diseases. Https://Trialsearch.Who.Int/Trial2.Aspx?TrialID=IRCT201402068307N4. 2014.  866. N I. Evaluation of Vitamin D in pre-procedural cardiac injury following angioplasty in patients with ischemic heart diseases. Https://Trialsearch.Who.Int/Trial2.Aspx?TrialID=IRCT201402078307N6. 2015.  867. N I. The effect of oil massage therapy on severity of restless legs syndrome. Https://Trialsearch.Who.Int/Trial2.Aspx?TrialID=IRCT20140708018395N3. 2020.  868. N I. Comparative evaluation of the effect of two different fluid therapy regimen on the bowel motility return after surgery. Https://Trialsearch.Who.Int/Trial2.Aspx?TrialID=IRCT2014090711398N7. 2014.  869. N I. Allopurinol in the prevention of pre-procedural myocardial injury following angioplasty. Https://Trialsearch.Who.Int/Trial2.Aspx?TrialID=IRCT201412088307N11. 2017.  870. N I. Comparing the High Flux versus Low Flux membranes. Https://Trialsearch.Who.Int/Trial2.Aspx?TrialID=IRCT2014121712789N10. 2015.  871. N I. Effect of acupressure on constipation of hemodialysis patients. Https://Trialsearch.Who.Int/Trial2.Aspx?TrialID=IRCT2015043022027N1. 2015.  872. N I. Cold hemodialysis effect on quality of life and anemia. Https://Trialsearch.Who.Int/Trial2.Aspx?TrialID=IRCT2015062722930N1. 2017.  873. N I. Clinical trial of effectiveness of education based on BASNEF model on adherence to dietary in hemodialysis clients. Https://Trialsearch.Who.Int/Trial2.Aspx?TrialID=IRCT2016020626388N1. 2016.  874. N I. Evaluation of the effects of Calcitriol on acute kidney injury. Https://Trialsearch.Who.Int/Trial2.Aspx?TrialID=IRCT2016022326716N1. 2016.  875. N I. Evaluation of curcumin in pre-procedural cardiac injury following angioplasty in patients with ischemic heart diseases. Https://Trialsearch.Who.Int/Trial2.Aspx?TrialID=IRCT201603038307N13. 2017.  876. N I. Effect of N-Acetyl Cysteine on liver and kidney function tests after surgical bypass in obstructive jaundice patients. Https://Trialsearch.Who.Int/Trial2.Aspx?TrialID=IRCT2016041016473N7. 2016.  877. N I. The effects of protein whey, lipoic acid and protein whey in combination with lipoic acid among acute ischemic stroke patients. Https://Trialsearch.Who.Int/Trial2.Aspx?TrialID=IRCT2016061428450N1. 2016.  878. N I. The effect of music on comfort and vital signs of hemodialysis patients. Https://Trialsearch.Who.Int/Trial2.Aspx?TrialID=IRCT2016092323446N8. 2016.  879. N I. the effect of topical application of rosemary and menthol on musculoskeletal pain in hemodialysis patients. Https://Trialsearch.Who.Int/Trial2.Aspx?TrialID=IRCT2016101430296N1. 2016.  880. N I. Effects of Melatonin on injury caused by transplant process in renal transplant patients. Https://Trialsearch.Who.Int/Trial2.Aspx?TrialID=IRCT201610203812N5. 2016.  881. N I. The effect of the comfort-oriented nursing care on comfort and quality of life. Https://Trialsearch.Who.Int/Trial2.Aspx?TrialID=IRCT2016103130607N1. 2017.  882. N I. Effect of coenzyme Q10 supplementation in dialysis patients. Https://Trialsearch.Who.Int/Trial2.Aspx?TrialID=IRCT2017060734370N1. 2017.  883. N I. The effect of preoperative and postoperative vitamin D medication therapy on postoperative urinary IL18,KIM1levels in modarres hospital CABG patients in 1397 year. Https://Trialsearch.Who.Int/Trial2.Aspx?TrialID=IRCT20180131038578N1. 2019.  884. N I. Effect of Stepwise profile of Fluid flow rate and increase Blood flow rate in Adequacy of Hemodialysis. Https://Trialsearch.Who.Int/Trial2.Aspx?TrialID=IRCT20180407039218N1. 2018.  885. N I. The effect of Bio-impedance Analysis in fluid management of patients on Continuous Renal Replacement Therapy. Https://Trialsearch.Who.Int/Trial2.Aspx?TrialID=IRCT20181108041595N1. 2019.  886. N I. ?Effect of Empagliflozin in ?heart failure preserved ejection fraction. Https://Trialsearch.Who.Int/Trial2.Aspx?TrialID=IRCT20190122042450N2. 2019.  887. N I. The effect of education on Self - efficacy in patients under hemodialysis. Https://Trialsearch.Who.Int/Trial2.Aspx?TrialID=IRCT20191103045314N1. 2020.  888. N I. The effect of curcumin supplementaion on clinical symptoms , acute phase reactants and inflamatory cytokines in patients with covid-19 hospitalization. Https://Trialsearch.Who.Int/Trial2.Aspx?TrialID=IRCT20211126053183N1. 2021.  889. N I. Investigating the effect of foot massage on comfort, muscle cramps and nausea. Https://Trialsearch.Who.Int/Trial2.Aspx?TrialID=IRCT20230122057181N1. 2023.  890. Ishibashi M, Kokado Y, Takahara S, Okuyama A, Kurita T, Amemiya H, et al. Randomized multicenter study for comparison of University of Wisconsin solution vs Euro-Collins solution on early renal allograft function in the non-heart-beating cadaver donor. Transpl P. 1994;26(4):2405-8.  891. H I, S T, N A, S T, T C, K T, et al. A Prospective Randomized, Comparative Trial of High-Dose Mizoribine Versus Mycophenolate Mofetil in Combination With Tacrolimus and Basiliximab for Living Donor Renal Transplant: a Multicenter Trial. Exp Clin Transplant. 2016(5).  892. ISRCTN. Evaluation of lifespan in AN69ST with two different heparinization strategies. Https://Trialsearch.Who.Int/Trial2.Aspx?TrialID=ISRCTN01121161. 2008.  893. ISRCTN. Pre-operative volume replacement versus usual care in diabetic patients having coronary artery bypass graft (CABG) surgery: a randomised controlled trial. Https://Trialsearch.Who.Int/Trial2.Aspx?TrialID=ISRCTN02159606. 2008.  894. ISRCTN. Study to determine the pharmacokinetic profile, safety and tolerability of Sildenafil in cardiac surgery. Https://Trialsearch.Who.Int/Trial2.Aspx?TrialID=ISRCTN06134609. 2013.  895. ISRCTN. Investigating the effect of adequate energy and protein intake for children after cardiopulmonary bypass surgery. Https://Trialsearch.Who.Int/Trial2.Aspx?TrialID=ISRCTN10130540. 2020.  896. ISRCTN. Does giving kidney donors intravenous fluids the night before kidney donation make any difference? Https://Trialsearch.Who.Int/Trial2.Aspx?TrialID=ISRCTN10199225. 2015.  897. ISRCTN. Can a drug with the potential to boost the immune system (interferon gamma) prevent infection in patients who are critically ill and at particularly high risk of developing new infections during their stay in an intensive care unit? Https://Trialsearch.Who.Int/Trial2.Aspx?TrialID=ISRCTN10449048. 2023.  898. ISRCTN. Statins for improving organ outcome in transplantation. Https://Trialsearch.Who.Int/Trial2.Aspx?TrialID=ISRCTN11440354. 2021.  899. ISRCTN. Infant kidney dialysis and filtration: the I-KID study. Https://Trialsearch.Who.Int/Trial2.Aspx?TrialID=ISRCTN13787486. 2018.  900. ISRCTN. Multicentre evaluation of sodium bicarbonate in acute kidney injury in critical care (MOSAICC). Https://Trialsearch.Who.Int/Trial2.Aspx?TrialID=ISRCTN14027629. 2021.  901. ISRCTN. Understanding how COVID-19 leads to respiratory failure in COVID-19 positive patients. Https://Trialsearch.Who.Int/Trial2.Aspx?TrialID=ISRCTN14212905. 2020.  902. ISRCTN. Prepare for kidney care. Https://Trialsearch.Who.Int/Trial2.Aspx?TrialID=ISRCTN17133653. 2017.  903. ISRCTN. A feasibility study of octreotide infusion during liver transplant. Https://Trialsearch.Who.Int/Trial2.Aspx?TrialID=ISRCTN17573492. 2021.  904. ISRCTN. Cold oxygenated machine preservation of aged renal donation after cardiovascular death transplants. Https://Trialsearch.Who.Int/Trial2.Aspx?TrialID=ISRCTN32967929. 2013.  905. ISRCTN. Mortality and recovery of renal function in acute kidney injury patients treated with extended dialysis. Https://Trialsearch.Who.Int/Trial2.Aspx?TrialID=ISRCTN33774458. 2014.  906. ISRCTN. Ward based goal-directed fluid therapy (GDFT) in acute pancreatitis. Https://Trialsearch.Who.Int/Trial2.Aspx?TrialID=ISRCTN36077283. 2018.  907. ISRCTN. Hospital at home as a model of early discharge from hospital. Https://Trialsearch.Who.Int/Trial2.Aspx?TrialID=ISRCTN36662318. 2009.  908. ISRCTN. Pre- versus post-dilution haemofiltration: a prospective randomised cross-over study. Https://Trialsearch.Who.Int/Trial2.Aspx?TrialID=ISRCTN38768865. 2005.  909. ISRCTN. Work Package 2 (WP2) - Normothermic Liver Perfusion Vs Cold Storage in Liver Transplants. Https://Trialsearch.Who.Int/Trial2.Aspx?TrialID=ISRCTN39731134. 2014.  910. ISRCTN. BK Viremia: kinase Inhibition to Decrease Nephropathy Intervention Trial. Https://Trialsearch.Who.Int/Trial2.Aspx?TrialID=ISRCTN40228609. 2010.  911. ISRCTN. Preeclampsia prevention by timed birth at term. Https://Trialsearch.Who.Int/Trial2.Aspx?TrialID=ISRCTN41632964. 2022.  912. ISRCTN. An investigation into the treatment of the donor kidney to see if this improves the recovery of the kidney after transplantation. Https://Trialsearch.Who.Int/Trial2.Aspx?TrialID=ISRCTN49958194. 2012.  913. ISRCTN. CArdiac Death kidney Machine Perfusion trial. Https://Trialsearch.Who.Int/Trial2.Aspx?TrialID=ISRCTN50082383. 2011.  914. ISRCTN. HydroCortisone in Severe Acute Pancreatitis. Https://Trialsearch.Who.Int/Trial2.Aspx?TrialID=ISRCTN53054042. 2008.  915. ISRCTN. Comparison of haemodialysis and peritoneal dialysis in babies. Http://Www.Who.Int/Trialsearch/Trial2.Aspx?TrialID=ISRCTN55761337. 2011.  916. ISRCTN. Effect of ischaemic pre-conditioning on cardiac function during elective open abdominal aortic aneurysm repair. Https://Trialsearch.Who.Int/Trial2.Aspx?TrialID=ISRCTN58848790. 2006.  917. ISRCTN. Goal-directed therapy: to determine a systematic approach in reducing complications after a cardiac surgery. Https://Trialsearch.Who.Int/Trial2.Aspx?TrialID=ISRCTN72151906. 2013.  918. ISRCTN. A randomized multicenter trial to assess the efficacy of a combined therapy with Sirolimus (Rapamune®), MMF (Cellsept®) and corticosteroids after early elimination of cyclosporin compared to a standard immunosuppression with cyclosporin, MMF and corticosteroids in patients after kidney transplantation. Https://Trialsearch.Who.Int/Trial2.Aspx?TrialID=ISRCTN74429508. 2005.  919. ISRCTN. Comparison of the efficacy of two dialysis filters in removing larger molecules that accumulate in acute renal failure. Https://Trialsearch.Who.Int/Trial2.Aspx?TrialID=ISRCTN88486733. 2012.  920. ISRCTN. A study of the benefit of a medicine called eculizumab in Shiga-Toxin producing E. Coli Haemolytic Syndrome. Https://Trialsearch.Who.Int/Trial2.Aspx?TrialID=ISRCTN89553116. 2016.  921. ISRCTN. Quality assessment of kidneys by ex-vivo warm perfusion prior to transplantation. Https://Trialsearch.Who.Int/Trial2.Aspx?TrialID=ISRCTN91315246. 2016.  922. ISRCTN. PROthrombin complex concentrate versus fresh frozen Plasma for bleeding in adults undergoing HEart SurgerY (PROPHESY-2 trial). Https://Trialsearch.Who.Int/Trial2.Aspx?TrialID=ISRCTN92114384. 2024.  923. N I, F O, SA R, A K, BL K, M M, et al. The renin-aldosterone axis in kidney transplant recipients and its association with allograft function and structure. Kidney Int. 2014(2). http://doi.org/10.1038/ki.2013.278  924. T I, N F, E I, K D, M F, T M, et al. The effect of an L/N-type calcium channel blocker on intradialytic blood pressure in intradialytic hypertensive patients. Clinical and Experimental Hypertension (New York, N.Y. : 1993). 2019(1). http://doi.org/10.1080/10641963.2018.1445753  925. E JZ, MI SL, JM P, CN P, R V, HG C, et al. Worsening renal function in patients hospitalized for acute decompensated heart failure. Eur J Heart Fail. 2017. http://doi.org/10.1002/ejhf.833  926. M J, J A, S J, JY L, JA B, JM C, et al. The radiographic assessment of lung Edema (RALE) score is associated with survival and may be useful to identify focal and non-focal lung imaging phenotypes in patients with ARDS. Am J Resp Crit Care. 2020(1).  927. Jabayeva N, Bekishev B, Lesbekov T, Kaliyev R, Nurmykhametova Z, Li T, et al. EXTRACORPOREAL BLOOD PURIFICATION DURING OPEN HEART SURGERY WITH PROLONGED CPB: CYTOSORB 300 VS JAFRON HA330. Nephrol Dial Transpl. 2023;38:i1121-2. http://doi.org/10.1093/ndt/gfad063c_3285  928. Jacob M, Sahu S, Singh YP, Mehta Y, Yang KY, Kuo SW, et al. A prospective observational study of rational fluid therapy in asian intensive care units: Another puzzle piece in fluid therapy. Indian J Crit Care M. 2020;24(11):1028-36. http://doi.org/10.5005/jp-journals-10071-23653  929. PA J, D S, A I, WS O, YC L, R L, et al. Genetic and clinical determinants of early, acute calcineurin inhibitor-related nephrotoxicity: results from a kidney transplant consortium. Transplantation. 2012(6). http://doi.org/10.1097/TP.0b013e3182461288  930. V J, Y M, A G, R S, A R, N T. The role of neutrophil gelatinase-associated lipocalin in predicting acute kidney injury in patients undergoing off-pump coronary artery bypass graft: a pilot study. Ann Card Anaesth. 2016(2). http://doi.org/10.4103/0971-9784.179590  931. Jain A, Deval N, Paul L. A RECOVERED CASE OF COVID-19 MYOCARDITIS TREATED WITH IV IMMUNOGLOBULIN. Chest. 2020;158(4):A281. http://doi.org/10.1016/j.chest.2020.08.282  932. Jamale TE, Hase NK, Iqbal AM. Laparoscopic donor nephrectomy versus open donor nephrectomy: recipient's perspective. Saudi J Kidney Dis T. 2012;23(6):1175-80. http://doi.org/10.4103/1319-2442.103556  933. James MT, Har BJ, Tyrrell BD, Faris PD, Tan Z, Spertus JA, et al. Effect of Clinical Decision Support With Audit and Feedback on Prevention of Acute Kidney Injury in Patients Undergoing Coronary Angiography: A Randomized Clinical Trial. Jama-J Am Med Assoc. 2022;328(9):839-49. http://doi.org/10.1001/jama.2022.13382  934. Jannati M, Shahbazi S, Lotfi HR. EFFECT of INTRAOPERATIVE DEXMEDETOMIDINE on RENAL FUNCTION in PATIENTS UNDERGOING CARDIOPULMONARY BYPASS GRAFT SURGERY under CARDIOPULMONARY BYPASS. Bulletin of Pharmaceutical Sciences. Assiut. 2021;44(2):467-76. http://doi.org/10.21608/BFSA.2021.207174  935. JapicCTI-. An open-label study of FCU-08 in patients with acute kidney injury requiring continuous blood purification therapy. Https://Trialsearch.Who.Int/Trial2.Aspx?TrialID=JPRN-JapicCTI-152797. 2015.  936. JapicCTI-. A randomized study of FCU-08 in patients with acute kidney injury requiring continuous blood purification therapy. Https://Trialsearch.Who.Int/Trial2.Aspx?TrialID=JPRN-JapicCTI-163262. 2016.  937. AG J, IA H, C G, K B, E B, A H. The impact study: 100-day vs 200-day prophylaxis with valganciclovir (Valcyte®) to reduce CMV disease post-transplant is cost-effective within us health care setting. Transplantation. 2010.  938. M J, E C, E M, A G, F M, L G, et al. Cellular Immunity to Predict the Risk of Cytomegalovirus Infection in Kidney Transplantation: a Prospective, Interventional, Multicenter Clinical Trial. Clin Infect Dis. 2020(9). http://doi.org/10.1093/cid/ciz1209  939. F J, Z S, M R, R A, F H. The effect of N-acetyl cysteine injection on renal function after coronary artery bypass graft surgery: a randomized double blind clinical trial. J Cardiothorac Surg. 2021(1). http://doi.org/10.1186/s13019-021-01550-7  940. JC J, E J, HY K, MG K, HJ I, HJ J, et al. Desensitization using bortezomib and high-dose immunoglobulin increases rate of deceased donor kidney transplantation. Medicine. 2016(5). http://doi.org/10.1097/MD.0000000000002635  941. JH J, A B, B F, KR W. Effects of acute intradialytic exercise on cardiovascular responses in hemodialysis patients. Hemodialysis International. International Symposium On Home Hemodialysis. 2018(4). http://doi.org/10.1111/hdi.12664  942. Jeppesen KK, Rasmussen SB, Kjaergaard J, Schmidt H, Mølstrøm S, Beske RP, et al. Acute kidney injury after out-of-hospital cardiac arrest. Crit Care. 2024;28(1). http://doi.org/10.1186/s13054-024-04936-w  943. A J, D A, B B, A B, C C, SM C, et al. Five-year study of tacrolimus as secondary intervention versus continuation of cyclosporine in renal transplant patients at risk for chronic renal allograft failure. Transplantation. 2008(7). http://doi.org/10.1097/TP.0b013e318186dd0c  944. B J, S R, S B. Role of aminophylline in the management of neonatal acute renal failure - a prospective study. Nephrology (Carlton, Vic.). 2005.  945. P J, W J, J T, H Z, J Z, Z L, et al. Acute Effects of Hemodiafiltration Versus Conventional Hemodialysis on Endothelial Function and Inflammation: a Randomized Crossover Study. Medicine. 2016(16). http://doi.org/10.1097/MD.0000000000003440  946. Jiang J, Liu X, Cheng Z, Liu Q, Xing W. Effect of preoperative moderate-dose statin and duration on acute kidney injury after cardiac surgery: a retrospective cohort study. Curr Med Res Opin. 2024;40(2):229-38. http://doi.org/10.1080/03007995.2023.2286317  947. Jiang L, Zeng R, Yang K, Mi DH, Tian JH, Ma B, et al. Tidal versus other forms of peritoneal dialysis for acute kidney injury. Cochrane Database of Systematic Reviews (Online). 2012;6:CD007016.  948. T J, L L, L D, S W, R Z, N S, et al. Hemoconcentration is associated with early faster fluid rate and increased risk of persistent organ failure in acute pancreatitis patients. JGH Open: An Open Access Journal of Gastroenterology and Hepatology. 2020(4). http://doi.org/10.1002/jgh3.12320  949. ZC J, ZX Y, MS J, H Z. Application of continuous veno-venous hemofiltration in patients with acute respiratory distress syndrome. Zhonghua Yi Xue Za Zhi. 2008(32).  950. JMA-IIA. Safety and efficacy of atrial natriuretic peptide for preventing contrast-induced nephropathy in patients with renal dysfunction. Https://Trialsearch.Who.Int/Trial2.Aspx?TrialID=JPRN-JMA-IIA00379. 2018.  951. HA J, S P, CD K, HY J, JH C, RH C, et al. Efficacy and safety of a balanced salt solution versus a 0.9% saline infusion for the prevention of contrast-induced acute kidney injury (BASIC trial): a study protocol for a randomized controlled trial. Trials. 2017(1). http://doi.org/10.1186/s13063-017-2202-2  952. Joannes-Boyau O, Honoré PM, Perez P, Bagshaw SM, Grand H, Canivet JL, et al. High-volume versus standard-volume haemofiltration for septic shock patients with acute kidney injury (IVOIRE study): A multicentre randomized controlled trial. Intens Care Med. 2013;39(9):1535-46. http://doi.org/10.1007/s00134-013-2967-z  953. Jodele S, Dandoy CE, Aguayo-Hiraldo P, Lane A, Teusink-Cross A, Sabulski A, et al. A prospective multi-institutional study of eculizumab to treat high-risk stem cell transplantation–associated TMA. Blood. 2024;143(12):1112-23. http://doi.org/10.1182/blood.2023022526  954. WB J, EF E. Management of end stage renal disease in persons living with hiv: A cost-effectiveness analysis. Open Forum Infect Di. 2017. http://doi.org/10.1093/ofid/ofx163.1103  955. P J, SD L, C C, LT J, L H. Acute effect of oral, intraperitoneal, and intravenous 1 alpha-hydroxycholecalciferol on markers of bone metabolism. Nephrology, Dialysis, Transplantation. 1994(5). http://doi.org/10.1093/ndt/9.5.524  956. Johansen KL, Smith MW, Unruh ML, Siroka AM, O'Connor TZ, Palevsky PM. Predictors of health utility among 60-day survivors of acute kidney injury in the Veterans Affairs/National Institutes of Health Acute Renal Failure Trial Network Study. Clin J Am Soc Nephro. 2010;5(8):1366-72. http://doi.org/10.2215/CJN.02570310  957. SG J, NM S, CW M. Effects of peritoneal dialysis fluid biocompatibility on baroreflex sensitivity. Kidney International. Supplement. 2008(108). http://doi.org/10.1038/sj.ki.5002612  958. Jordan SC, Choi J, Aubert O, Haas M, Loupy A, Huang E, et al. A phase I/II, double-blind, placebo-controlled study assessing safety and efficacy of C1 esterase inhibitor for prevention of delayed graft function in deceased donor kidney transplant recipients. Am J Transplant. 2018;18(12):2955-64. http://doi.org/10.1111/ajt.14767  959. VJ J, JK C, D D, RC P, NR P, RW J. Evidence that late perfusion does not influence delayed allograft function. Transplantation. 1994(3).  960. Journois D, Safran D, Castelain MH, Chanu D, Drévillon C, Barrier G. [Comparison of the antithrombotic effects of heparin, enoxaparin and prostacycline in continuous hemofiltration]. Ann Fr Anesth Reanim. 1990;9(4):331-7. http://doi.org/10.1016/s0750-7658(05)80244-8  961. JPRN-jRCTs. A Randomized Controlled Trial to Compare The Sedative Efficacy and Safety of Isoflurane and Dexmedemomidine in Patients after Cardiovascular Surgery. Https://Trialsearch.Who.Int/Trial2.Aspx?TrialID=JPRN-JRCTs021220016. 2022.  962. JPRN-UMIN. Evaluation of the efficacy of neuromuscular electrical stimulation in ICU patients undergoing continuous renal replacement therapy. Https://Trialsearch.Who.Int/Trial2.Aspx?TrialID=JPRN-UMIN000049622. 2022.  963. jRCT. Prevention of acute kidney injury by cardiopulmonary bypass: role of haptoglobin administration for hemolysis. Https://Trialsearch.Who.Int/Trial2.Aspx?TrialID=JPRN-JRCT1031190038. 2019.  964. jRCTs. The efficacy and safety of empagliflozin in patients with acute heart failure. Https://Trialsearch.Who.Int/Trial2.Aspx?TrialID=JPRN-JRCTs031210682. 2022.  965. jRCTs. Goreisan for Heart Failure Trial. Https://Trialsearch.Who.Int/Trial2.Aspx?TrialID=JPRN-JRCTs051200101. 2020.  966. Judge C, Murphy R, Reddin C, Cormican S, Smyth A, O'Halloran M, et al. Adaptive design methods in dialysis clinical trials-a systematic review. Nephrol Dial Transpl. 2021;36(SUPPL 1):i469. http://doi.org/10.1093/ndt/gfab098.0032  967. Judge C, Murphy R, Reddin C, Cormican S, Smyth A, O'Halloran M, et al. Trends in Adaptive Design Methods in Dialysis Clinical Trials: A Systematic Review. Kidney Med. 2021;3(6):925-41. http://doi.org/10.1016/j.xkme.2021.08.001  968. Jun M, Lambers Heerspink HJ, Ninomiya T, Gallagher M, Bellomo R, Myburgh J, et al. Intensities of renal replacement therapy in acute kidney injury: A systematic review and meta-analysis. Clin J Am Soc Nephro. 2010;5(6):956-63. http://doi.org/10.2215/CJN.09111209  969. Jung S, Jhee J, Park J, Yoo T, Kang S, Kim D, et al. Electrolyte and mineral disturbances in septic acute kidney injury patients undergoing continuous renal replacement therapy. Nephrology. 2016;21:66. http://doi.org/10.1111/nep.12887  970. MdZ K, S A, F Z, MR B, NN K, MdH R. Abortion related acute renal failure - A study in Dhaka Medical College Hospital. Bangladesh Renal Journal. 2001(2).  971. VS K, L F, LP M, SA J, DF M. The effect of dopamine on graft function in patients undergoing renal transplantation. Anesth Analg. 1993(2).  972. BD K. Two-year results of multicenter phase III trials on the effect of the addition of sirolimus to cyclosporine-based immunosuppressive regimens in renal transplantation. Transpl P. 2003(3 Suppl). http://doi.org/10.1016/S0041-1345(03)00353-1  973. BD K. Efficacy of sirolimus compared with azathioprine for reduction of acute renal allograft rejection: a randomised multicentre study. The Rapamune US Study Group. Lancet (London, England). 2000(9225). http://doi.org/10.1016/s0140-6736(00)02480-6  974. Kail D, Arif F, Sultan-Ali I, Velamuri SR, Hill DM. A Retrospective Chart Review to Determine Hypophosphatemia Incidence and Phosphorus Supplementation Requirements in Patients With Severe Thermal Cutaneous Injuries Receiving High-Volume Hemofiltration. Journal of Burn Care and Research. 2023;44(2):234-9. http://doi.org/10.1093/jbcr/irac047  975. A K, J W, C M, M H, C B, P P, et al. Steroid pretreatment of organ donors to prevent postischemic renal allograft failure: a randomized, controlled trial. Ann Intern Med. 2010(4). http://doi.org/10.7326/0003-4819-153-4-201008170-00003  976. Kaja Kamal RM, Farrington K, Busby AD, Wellsted D, Chandna H, Mawer LJ, et al. Initiating haemodialysis twice-weekly as part of an incremental programme may protect residual kidney function. Nephrol Dial Transpl. 2019;34(6):1017-25. http://doi.org/10.1093/ndt/gfy321  977. Kama K, Pospiech L, Hahn D, Kitterer D, Illerhaus G. Hematopoietic stem cell transplantation for relapsed hemophagocytic syndrome-associated intravascular large B-cell lymphoma with concomitant AA amyloidosis and end stage renal disease. Oncol Res Treat. 2020;43:66. http://doi.org/10.1159/000510995  978. MD K, YA H, SM Z. Role of Topical Mupirocin as a Prophylaxis in Catheter Related Infection in Haemodialysis Patients. International Medical Journal. 2004(1).  979. N K, V G, A K, G M, N L, B C, et al. Impact of early or delayed cyclosporine on delayed graft function in renal transplant recipients: a randomized, multicenter study. Am J Transplant. 2006(5 Pt 1). http://doi.org/10.1111/j.1600-6143.2006.01291.x  980. D K, K K, P C, A H, O M, A G, et al. The influence of warm ischemia elimination on kidney injury during transplantation - clinical and molecular study. Sci Rep-Uk. 2016. http://doi.org/10.1038/srep36118  981. T K, F K, M S, S H, B Z, von Dossow V, et al. Comparison of 6% hydroxyethyl starch and 5% albumin for volume replacement therapy in patients undergoing cystectomy (CHART): study protocol for a randomized controlled trial. Trials. 2015. http://doi.org/10.1186/s13063-015-0866-z  982. Kanduri SR, Cheungpasitporn W, Kovvuru K, Vaitla P, Thongprayoon C, Kashani K. Recovery of renal function among left ventricular assist device patients with severe AKI requiring renal replacement therapy: A meta-analysis. J Am Soc Nephrol. 2020;31:86.  983. Kanitra JJ, Power AD, Hayward RD, Haouilou JC, Edhayan E. Malfunctioning temporary hemodialysis catheters in patients with novel coronavirus disease 2019. J Vasc Surg. 2021;73(6):1881-8. http://doi.org/10.1016/j.jvs.2020.11.033  984. Karacan H, Valentin A, Carl P. Mild therapeutic hypothermia after cardiac arrest through continuous dialysis. Ugeskrift for Laeger. 2009;171(17):1396-400.  985. Karkouti K, Callum J, Wijeysundera DN, Rao V, Crowther M, Grocott HP, et al. Point-of-Care Hemostatic Testing in Cardiac Surgery: A Stepped-Wedge Clustered Randomized Controlled Trial. Circulation. 2016;134(16):1152-62. http://doi.org/10.1161/CIRCULATIONAHA.116.023956  986. KM K, GS T, GD W. Calcineurin inhibitor withdrawal or tapering for kidney transplant recipients. Cochrane Db Syst Rev. 2017(7). http://doi.org/10.1002/14651858.CD006750.pub2  987. Karvellas CJ, Taylor S, Bigam D, Kneteman NM, Shapiro AMJ, Romanovsky A, et al. Intraoperative continuous renal replacement therapy during liver transplantation: a pilot randomized-controlled trial (INCEPTION). Canadian Journal of Anesthesia. 2019;66(10):1151-61. http://doi.org/10.1007/s12630-019-01454-0  988. Karvellas CJ, Taylor S, Özelsel T, Bishop E, Cave D, Bigam D, et al. A pilot randomized controlled trial of intra-operative continuous renal replacement therapy during liver transplantation: The inception study. Hepatology. 2018;68:7A. http://doi.org/10.1002/hep.30256  989. Karvellas C, Taylor S, Ozelzel T, Bishop E, Cave D, Bigam D, et al. Intraoperative continuous renal replacement therapy in liver transplantation: A pilot randomized controlled trial (INCEPTION study). Crit Care. 2018;22. http://doi.org/10.1186/s13054-018-1973-5  990. BL K, HJ J, PJ G, KL H, VK R, DC D, et al. A randomized trial comparing cyclosporine induction with sequential therapy in renal transplant recipients. Am J Kidney Dis. 1997(5). http://doi.org/10.1016/s0272-6386(97)90487-x  991. T K, A Q, A D, EG R, I R, LL N, et al. A double-blind randomised controlled investigation into the efficacy of Mirococept (APT070) for preventing ischaemia reperfusion injury in the kidney allograft (EMPIRIKAL): study protocol for a randomised controlled trial. Trials. 2017(1). http://doi.org/10.1186/s13063-017-1972-x  992. E K, E T, A S, S D, M C, E Z, et al. Daratumumab with dexamethasone in patients with relapsed/refractory multiple myeloma and severe renal impairment: results on efficacy and safety of the phase 2 dare study. Blood. 2020(SUPPL 1). http://doi.org/10.1182/blood-2020-140322  993. Katulka R, Al Saadon A, Sebanstianski M, Featherston R, Vandermeer B, Gibney N, et al. Determining the optimal time for liberation from renal replacement therapy in critically ill patients: A systematic review and meta-analysis (done RRT). Canadian Journal of Anesthesia. 2019;66(1):S30-1. http://doi.org/10.1007/s12630-019-01292-0  994. HM K, D S, PS F, AT S. High dose (bolus) intravenous methylprednisolone at the time of kidney homotransplantation. Ann Surg. 1977(5). http://doi.org/10.1097/00000658-197711000-00015  995. DB K, ES W, AR S, J L, A M, A W, et al. Belatacept for Simultaneous Calcineurin Inhibitor and Chronic Corticosteroid Immunosuppression Avoidance: two-Year Results of a Prospective, Randomized Multicenter Trial. Clin J Am Soc Nephro. 2021(9). http://doi.org/10.2215/CJN.13100820  996. GA K, NW L, WE M, AL C, L K, JP E. Evidence that C-reactive protein or IL-6 are not surrogates for all inflammatory cardiovascular risk factors in hemodialysis patients. Blood Purificat. 2006(5‐6). http://doi.org/10.1159/000096471  997. KCT. Effect of exogenous albumin on the incidence of postoperative acute kidney injury in patients with hypoalbuminemia undergoing off-pump coronary bypass surgery. Http://Www.Who.Int/Trialsearch/Trial2.Aspx?TrialID=KCT0000210. 2011.  998. KCT. Immediate Versus Delayed Coronary Angiography in Patients With Non-ST-Segment Acute Coronary Syndrome with Acute Decompensated Heart Failure. Http://Www.Who.Int/Trialsearch/Trial2.Aspx?TrialID=KCT0006035. 2021.  999. KCT. REvaSCUlarization StratEgy of Multivessel Coronary Artery Disease for Patients with Acute Myocardial Infarction Complicated by Cardiogenic SHOCK Undergoing Veno-arterial Extracorporeal Membrane Oxygenator: randomized-Controlled Trial. Https://Trialsearch.Who.Int/Trial2.Aspx?TrialID=KCT0007715. 2022.  1000. KCT. Tailored Antiplatelet Therapy for High-Risk Patients At 12 Months after Drug-Eluting Stent implantation. Https://Trialsearch.Who.Int/Trial2.Aspx?TrialID=KCT0008489. 2023.  1001. CK K, L A, A B, AS B, G K, AK V, et al. Combination of granulocyte colony-stimulating factor and erythropoietin improves outcomes of patients with decompensated cirrhosis. Gastroenterology. 2015(7). http://doi.org/10.1053/j.gastro.2015.02.054  1002. Keith P, Eyadiel L, Hodges J, Sands K, Watkins J. Therapeutic plasma exchange for thrombocytopenia-associated multiorgan failure in septic shock. Crit Care Med. 2018;46:696. http://doi.org/10.1097/01.ccm.0000529425.30564.64  1003. JA K. Impact of Consensus Papers versus Randomized Trials in Critical Care Nephrology. Blood Purificat. 2020(6). http://doi.org/10.1159/000507422  1004. JA K. Renal replacement therapy in critically ill patients with acute renal failure: does a greater dose improve survival? Commentary. Nature Clinical Practice Nephrology. 2007(3). http://doi.org/10.1038/ncpneph0398  1005. Kellum JA, Angus DC, Johnson JP, Leblanc M, Griffin M, Ramakrishnan N, et al. Continuous versus intermittent renal replacement therapy: A meta-analysis. Intens Care Med. 2002;28(1):29-37. http://doi.org/10.1007/s00134-001-1159-4  1006. Kellum JA, Chawla LS, Keener C, Singbartl K, Palevsky PM, Pike FL, et al. The Effects of Alternative Resuscitation Strategies on Acute Kidney Injury in Patients with Septic Shock. Am J Resp Crit Care. 2016;193(3):281-7. http://doi.org/10.1164/rccm.201505-0995OC  1007. Kellum JA, Johnson JP, Kramer D, Palevsky P, Brady JJ, Pinsky MR. Diffusive vs. convective therapy: Effects on mediators of inflammation in patients with severe systemic inflammatory response syndrome. Crit Care Med. 1998;26(12):1995-2000. http://doi.org/10.1097/00003246-199812000-00027  1008. Kelly YP, Waikar SS, Mendu ML. When to stop renal replacement therapy in anticipation of renal recovery in AKI: The need for consensus guidelines. Semin Dialysis. 2019;32(3):205-9. http://doi.org/10.1111/sdi.12773  1009. AL K, GD W, SH J, GS T. Interventions for treating central venous haemodialysis catheter malfunction. Cochrane Db Syst Rev. 2017(10). http://doi.org/10.1002/14651858.CD011953.pub2  1010. P K, R B, S K, M C, C M, Z K, et al. Meta-analysis of basiliximab for immunoprophylaxis in renal transplantation. Biodrugs. 2003(4). http://doi.org/10.2165/00063030-200317040-00006  1011. Kerrie JP, Bagshaw SM, Brindley PG. Early versus late parenteral nutrition in the adult ICU: Feeding the patient or our conscience? Canadian Journal of Anesthesia. 2012;59(5):494-8. http://doi.org/10.1007/s12630-012-9674-z  1012. Kes P. Biocompatibility of dialysis membrane: Fact or fiction. Acta Clin Croat. 1999;38(1):45-50.  1013. Khader A, Zelnick L, Sathe NA, Kestenbaum BR, Himmelfarb J, Johnson N, et al. The Effect of Fluid Resuscitation Strategy on Risk for Renal Replacement Therapy Differs Based on Presence of Acute Kidney Injury in the CLOVERS Trial. Am J Resp Crit Care. 2023;207(1). http://doi.org/10.1164/ajrccm-conference.2023.C74  1014. Khalafallah AA, Loi SW, Love S, Mohamed M, Mace R, Khalil R, et al. Early application of high cut-off haemodialysis for de-novo myeloma nephropathy is associated with long-term dialysis-independency and renal recovery. Mediterr J Hematol I. 2013;5(1). http://doi.org/10.4084/MJHID.2013.007  1015. Khalil MA, El Tahan MR, Khidr AM, Fallatah S, Abohamar AD, Amer MM, et al. Effects of norepinephrine infusion during cardiopulmonary bypass on perioperative changes in lactic acid level (Norcal). Perfusion (United Kingdom). 2023;38(8):1584-99. http://doi.org/10.1177/02676591221122350  1016. B K, L N. Allopurinol-induced drug reaction with eosinophilia and systemic symptoms (DRESS) syndrome complicated by acute renal failure. Rheumatology (United Kingdom). 2019. http://doi.org/10.1093/rheumatology/kez110.028  1017. M K, YH K, M T, W M, G B. Caloric requirements of critically ill hemodialysis patients. Crit Care Med. 2021(1 SUPPL 1). http://doi.org/10.1097/01.ccm.0000730420.25486.68  1018. RB K, Y E, M H, FJ D, W M, A H, et al. A controlled sequential evaluation of open donor nephrectomy versus classical and modified laparoscopic donor nephrectomy: an update. Transpl P. 2005(7). http://doi.org/10.1016/j.transproceed.2005.08.016  1019. Khositrangsikun K, Srisawa N, Parapiboon W, Tatiyanupanwong S, Lumlertgul N, Peerapornratana S. The study comparison the efficacy and outcome of acute peritoneal dialysis and intermittent haemodialysis in criticallyill patients with acute kidney injury. Nephrology. 2020;25(SUPPL 4):39. http://doi.org/10.1111/nep.v25.S4  1020. Khoundabi B, Behzadnia N, Ahmadi ZH, Chitsazan M, Tabarsi P, Jamaati H, et al. A randomized controlled clinical trial on therapeutic effects of teicoplanin and vancomycin after cardiac surgery due to MRSA infective endocarditis in ICU patients. Iran Red Crescent Me. 2020;23(1). http://doi.org/10.32592/IRCMJ.2021.23.1.69  1021. JT K, D C, T S, C H, SM B, E K, et al. Pharmacokinetics and total elimination of meropenem and vancomycin in intensive care unit patients undergoing extended daily dialysis. Crit Care Med. 2006(1).  1022. Kielstein JT, Kretschmer U, Ernst T, Hafer C, Bahr MJ, Haller H, et al. Efficacy and Cardiovascular Tolerability of Extended Dialysis in Critically Ill Patients: A Randomized Controlled Study. Am J Kidney Dis. 2004;43(2):342-9. http://doi.org/10.1053/j.ajkd.2003.10.021  1023. Kierdorf HP. Renal replacement therapy in intensive care medicine. Procedure selection, indications and dosage. Nephrologe. 2011;6(2):135-42. http://doi.org/10.1007/s11560-010-0454-x  1024. Kierdorf HP. Renal replacement therapy in acute renal failure. Deut Med Wochenschr. 2010;135(47):2341-6. http://doi.org/10.1055/s-0030-1267520  1025. Kiessling AH, Dietz J, Reyher C, Stock UA, Beiras-Fernandez A, Moritz A. Early postoperative serum cystatin C predicts severe acute kidney injury following cardiac surgery: a post-hoc analysis of a randomized controlled trial. J Cardiothorac Surg. 2014;9(1):10. http://doi.org/10.1186/1749-8090-9-10  1026. Kiessling AH, Isgro F, Lehmann A, Bergner R, Neher M, Saggau W. Continuous venovenous hemodialysis (CVVHD) with citrate calcium reduces postoperative bleeding complications after cardiac surgery. Thorac Cardiov Surg. 2011;58. http://doi.org/10.1055/s-0029-1246805  1027. J K, J P, S H, H Y, K K, JB P, et al. Ten-year observational follow-up of a randomized trial comparing cyclosporine and tacrolimus therapy combined with steroid withdrawal in living-donor renal transplantation. Clin Transplant. 2018(9). http://doi.org/10.1111/ctr.13372  1028. SB K, SK L, JS P, HS C, CD H, WS Y. Effects of fixed low-dose warfarin on hemostatic factors in continuous ambulatory peritoneal dialysis patients. Am J Kidney Dis. 2001(2). http://doi.org/10.1053/ajkd.2001.21303  1029. WH K, JH L, GS K, HY S, SJ K. The effect of remote ischemic postconditioning on graft function in patients undergoing living donor kidney transplantation. Transplantation. 2014(5). http://doi.org/10.1097/TP.0000000000000098  1030. Kim MJ, Heim M, Mayr M. Effect of corticosteroids during ongoing drug exposure in pantoprazole-induced interstitial nephritis. Nephrol Dial Transpl. 2010;25(5):1716-9. http://doi.org/10.1093/ndt/gfp751  1031. Kim WH, Lee SM, Choi JW, Kim EH, Lee JH, Jung JW, et al. Simplified clinical risk score to predict acute kidney injury after aortic surgery. J Cardiothor Vasc an. 2013;27(6):1158-66. http://doi.org/10.1053/j.jvca.2013.04.007  1032. AD K, LM J, DM H, NF R, JD P, HW S. Clinically stable human renal allografts contain histological and RNA-based findings that correlate with deteriorating graft function. Transplantation. 1999(10). http://doi.org/10.1097/00007890-199911270-00024  1033. A K, S K, R Y, M A, B A, Y E, et al. The effects of maintenance recombinant human erythropoietin therapy on ambulatory blood pressure recordings: conventional, Doppler, and tissue Doppler echocardiographic parameters. Artif Organs. 2005(12). http://doi.org/10.1111/j.1525-1594.2005.00166.x  1034. Kiser TH, MacLaren R, Fish DN, Hassell KL, Teitelbaum I. Bivalirudin versus unfractionated heparin for prevention of hemofilter occlusion during continuous renal replacement therapy. Pharmacotherapy. 2010;30(11):1117-26. http://doi.org/10.1592/phco.30.11.1117  1035. Kiser T, MacLaren R, Fish D, Hassell K, Teitelbaum I. Evaluation of predictive factors associated with hemofilter survival in patients receiving heparin or bivalirudin during continuous renal replacement therapy. Crit Care Med. 2009;37(12):A199. http://doi.org/10.1097/01.ccm.0000365439.11849.a2  1036. K K, SR M, S S. Comparison of OKT3 and antithymocyte globulin as induction immunosuppressive agents in renal transplantation. Transpl P. 1996(3).  1037. T K, JL G, YH K, WH T. Impact of Ultrafiltration on Serum Sodium Homeostasis and its Clinical Implication in Patients With Acute Heart Failure, Congestion, and Worsening Renal Function. Circulation. Heart Failure. 2017(2). http://doi.org/10.1161/CIRCHEARTFAILURE.116.003603  1038. Kitchlu A, Adhikari N, Burns KE, Friedrich JO, Garg AX, Klein D, et al. Outcomes of sustained low efficiency dialysis versus continuous renal replacement therapy in critically ill adults with acute kidney injury: a cohort study. Bmc Nephrol. 2015;16:127. http://doi.org/10.1186/s12882-015-0123-4  1039. M K, J P, M H, Z Y, F L, J Y, et al. Anti-thymocyte globulin induction to prevent renal dysfunction: is it necessary today? American Journal of Transplantation. Conference: 2014 World Transplant Congress. United States. 2014(Supplement 3). http://doi.org/10.1111/ajt.12878  1040. K K, B N, E K, M M. Effect of unfractionated and low-molecular-weight heparin on OPG, sRANKL, and von Willebrand factor concentrations during hemodialysis. Clinical and Applied Thrombosis/Hemostasis. 2014(4). http://doi.org/10.1177/1076029612463424  1041. Klingele M, Fliser D. Nephrology in the intensive care unit. Procedure selection. Nephrologe. 2014;9(4):278-83. http://doi.org/10.1007/s11560-013-0841-1  1042. Klinger M. Treatment of cancer in patients with chronic kidney disease: the nephrologist's point of view. Polish Archives of Internal Medicine. 2019;129:9-10. http://doi.org/10.20452/pamw.14821  1043. Klouche K, Amigues L, Deleuze S, Beraud JJ, Canaud B. Complications, effects on dialysis dose, and survival of tunneled femoral dialysis catheters in acute renal failure. Am J Kidney Dis. 2007;49(1):99-108. http://doi.org/10.1053/j.ajkd.2006.09.014  1044. P K, P S, T S, WR S, C S. Treatment of infection-associated purpura fulminans with protein C zymogen is associated with a high survival rate. Blood. 2013(21).  1045. GA K, A H, D F, O J, AA H, SJ K, et al. Levofloxacin for BK virus prophylaxis following kidney transplantation: a randomized clinical trial. Jama-J Am Med Assoc. 2014(20). http://doi.org/10.1001/jama.2014.14721  1046. Ko SH, Nan Z, Soh S, Shim JK, Lee HW, Kwak YL, et al. Effect of Retrograde Autologous Priming on Coagulation Assessed by Rotation Thromboelastometry in Patients Undergoing Valvular Cardiac Surgery. J Cardiothor Vasc an. 2024;38(4):939-45. http://doi.org/10.1053/j.jvca.2023.12.038  1047. MM K, A G, U S, A EB, R K, G T. Normothermic ex vivo allograft blood perfusion in clinical heart transplantation. Heart Surg Forum. 2014(3). http://doi.org/10.1532/HSF98.2014332  1048. Kohen JA, Whitley KY, Kjellstrand CM. Continuous arteriovenous hemofiltration: a comparison with hemodialysis in acute renal failure. Transactions - American Society for Artificial Internal Organs. 1985;31:169-75.  1049. JA K, GM G, DM P, CW K, MM M, SM S. N-acetylcysteine for patients with prolonged hypotension as prophylaxis for acute renal failure (NEPHRON). Crit Care Med. 2007(2). http://doi.org/10.1097/01.CCM.0000253816.83011.DB  1050. P K, R C, A G, LI PL, M B, S A, et al. Fit for Dialysis: a prospective 2-site parallel intervention trial of a filmed research-based drama to increase exercise amongst older hemodialysis patients. Int Urol Nephrol. 2021(6). http://doi.org/10.1007/s11255-020-02745-y  1051. J K, YW S, van Buren M, JH G, SR R, van der Molen AJ, et al. Randomised trial of no hydration vs. sodium bicarbonate hydration in patients with chronic kidney disease undergoing acute computed tomography-pulmonary angiography. Journal of Thrombosis and Haemostasis : JTH. 2014(10). http://doi.org/10.1111/jth.12701  1052. J K, YW S, Van Buren M, MJ G, S R, Van Der Molen AJ, et al. Hydration prior to ct-pulmonary angiography is not required for prevention of contrast induced-acute kidney injury: the randomized nefros trial. Circulation. 2013(22).  1053. S K, A S, A K, E P, O L, O Y, et al. Evaluation of the efficiency of allogenic adipose tissue mesenchymal stem cells therapy for tacrolimus dose minimisation in patients with acute kidney injury after liver transplantation. Transplantation. 2018(7).  1054. M K, A L, M F, M B, RM S, KH R, et al. Dialysis filter type determines the acute effect of haemodialysis on endothelial function and oxidative stress. Nephrology, Dialysis, Transplantation. 2003(7). http://doi.org/10.1093/ndt/gfg169  1055. Kovacic D, Saremian J, Aysola A. Comparison of reduction in triglyceride levels with or without therapeutic plasma exchange in the treatment of hypertriglyceridemia induced acute pancreatitis. J Clin Apheresis. 2015;30(2):110-1. http://doi.org/10.1002/jca.21385  1056. Kovacs B, Sullivan KJ, Hiremath S, Patel RV. Effect of sustained low efficient dialysis versus continuous renal replacement therapy on renal recovery after acute kidney injury in the intensive care unit: A systematic review and meta-analysis. Nephrology. 2017;22(5):343-53. http://doi.org/10.1111/nep.13009  1057. Koyner JL, Garg AX, Shlipak MG, Patel UD, Sint K, Hong K, et al. Urinary cystatin C and acute kidney injury after cardiac surgery. Am J Kidney Dis. 2013;61(5):730-8. http://doi.org/10.1053/j.ajkd.2012.12.006  1058. Kozek-Langenecker SA, Spiss CK, Gamsjäger T, Domenig C, Zimpfer M. Anticoagulation with prostaglandins and unfractionated heparin during continuous venovenous haemofiltration: A randomized controlled trial. Wien Klin Wochenschr. 2002;114(3):96-101.  1059. Kozlov B, Panfilov D, Lukinov V. Frozen Elephant Trunk for Aortic Dissection Using Different Hybrid Grafts: Preliminary Results from a Prospective Study. J Pers Med. 2023;13(5). http://doi.org/10.3390/jpm13050784  1060. BK K, L A, B B, B C, L B, H T, et al. Efficacy of Prolonged- and Immediate-release Tacrolimus in Kidney Transplantation: a Pooled Analysis of Two Large, Randomized, Controlled Trials. Transpl P. 2017(9). http://doi.org/10.1016/j.transproceed.2017.07.011  1061. BK K, C Z, MC K, C S, C H, M F, et al. Cardiovascular risk factors and estimated risk for CAD in a randomized trial comparing calcineurin inhibitors in renal transplantation. Am J Transplant. 2003(8). http://doi.org/10.1034/j.1600-6143.2003.00156.x  1062. Krastins J, Straume Z, Auzins J. Renal functions in children after open heart surgery. Intens Care Med. 2010;36:S175. http://doi.org/10.1007/s00134-010-1999-x  1063. Kreis H, Chkoff N, Droz D, Noel LH, Tolani M, Descamps JM, et al. Nonsteroid antiinflammatory agents as a substitute treatment for steroids in ATGAM-treated cadaver kidney recipients. Transplantation. 1984;37(2):139-45. http://doi.org/10.1097/00007890-198402000-00005  1064. Kremer D, Pieters TT, Verhaar MC, Berger SP, Bakker SJL, van Zuilen AD, et al. A systematic review and meta-analysis of COVID-19 in kidney transplant recipients: Lessons to be learned. Am J Transplant. 2021;21(12):3936-45. http://doi.org/10.1111/ajt.16742  1065. A K, A T, D C, A C, G D, N B, et al. Impact of early conversion from cyclosporin to everolimus on left ventricular mass index: a randomized controlled trial. Clin Transplant. 2017(10). http://doi.org/10.1111/ctr.13043  1066. Krogstrup NV, Oltean M, Bibby BM, Nieuwenhuijs-Moeke GJ, Dor FJ, Birn H, et al. Remote ischaemic conditioning on recipients of deceased renal transplants, effect on immediate and extended kidney graft function: a multicentre, randomised controlled trial protocol (CONTEXT). Bmj Open. 2015;5(8):e007941. http://doi.org/10.1136/bmjopen-2015-007941  1067. Krouzecky A, Chvojka J, Sykora R, Radej J, Karvunidis T, Novak I, et al. Regional cooling of the extracorporeal blood circuit: a novel anticoagulation approach for renal replacement therapy? Intens Care Med. 2009;35(2):364-70. http://doi.org/10.1007/s00134-008-1271-9  1068. Y K, J S, J F, AM EN, A F, JL H. Lack of effect of IGF-I on the glomerular filtration rate in non-diabetic patients with advanced chronic kidney disease. Growth Horm Igf Res. 2009(3). http://doi.org/10.1016/j.ghir.2008.10.002  1069. LU K, R B, FC R. Comparison of intraoperative versus intraoperative plus postoperative hemoadsorption therapy in cardiac surgery patients with endocarditis. Int J Artif Organs. 2019(4). http://doi.org/10.1177/0391398819831301  1070. S K, NC K, YH D, RN F, G M, V B, et al. Eculizumab Therapy for Chronic Antibody-Mediated Injury in Kidney Transplant Recipients: a Pilot Randomized Controlled Trial. Am J Transplant. 2017(3). http://doi.org/10.1111/ajt.14001  1071. M K, C M, M O, S C, N GN, G T, et al. Biomarker-guided implementation of the KDIGO guidelines to reduce the occurrence of acute kidney injury in patients after cardiac surgery (PrevAKI-multicentre): protocol for a multicentre, observational study followed by randomised controlled feasibility trial. Bmj Open. 2020(4). http://doi.org/10.1136/bmjopen-2019-034201  1072. A K, GS G, D D, DV B, A J, N T, et al. Common Inflammatory Markers and Outcome After Pediatric Cardiac Surgery With High Thoracic Epidural Anesthesia: a Randomized Controlled Study. World Journal for Pediatric & Congenital Heart Surgery. 2023(3). http://doi.org/10.1177/21501351221151053  1073. MS K, SG X, B F, D S, M H, MJ M, et al. Steroid avoidance in renal transplantation using basiliximab induction, cyclosporine-based immunosuppression and protocol biopsies. Clin Transplant. 2005(1). http://doi.org/10.1111/j.1399-0012.2004.00298.x  1074. Kumar D, Shaikh F, Warsha F, Haider S. Fluid management in diabetic ketoacidosis; comparing balanced fluids to normal saline. Am J Resp Crit Care. 2021;203(9). http://doi.org/10.1164/ajrccm-conference.2021.203.1_MeetingAbstracts.A2833  1075. Kumar S, Jordan S. Distinct delayed classical complement pathway activation highlights transition of ischemic acute kidney injury to chronic kidney disease with interstitial fibrosis/tubular atrophy. Am J Transplant. 2019;19:630-1. http://doi.org/10.1111/ajt.15406  1076. Kumar VA, Yeun JY, Depner TA, Don BR. Extended daily dialysis vs. continuous hemodialysis for ICU patients with acute renal failure: a two-year single center report. Int J Artif Organs. 2004;27(5):371-9. http://doi.org/10.1177/039139880402700505  1077. Kutlay S, Kurultak I, Nergizoglu G, Erturk S, Karatan O. Optimal treatment in critically ILL patients with acute kidney injury: Comparison of intermittant hemofiltration and hemodialysis in a randomized controlled trial. Nephrol Dial Transpl. 2013;28:i331. http://doi.org/10.1093/ndt/gft129  1078. Kutsogiannis DJ, Gibney RTN, Stollery D, Gao J. Regional citrate versus systemic heparin anticoagulation for continuous renal replacement in critically ill patients. Kidney Int. 2005;67(6):2361-7. http://doi.org/10.1111/j.1523-1755.2005.00342.x  1079. Kutsogiannis DJ, Mayers I, Chin WDN, Gibney RTN. Regional citrate anticoagulation in continuous venovenous hemodiafiltration. Am J Kidney Dis. 2000;35(5):802-11. http://doi.org/10.1016/S0272-6386(00)70248-4  1080. DR K, J M, K C, P E, B M, W C, et al. Secondary effects of immunosuppressive drugs after simultaneous pancreas-kidney transplantation. Nephrology, Dialysis, Transplantation. 2005. http://doi.org/10.1093/ndt/gfh1080  1081. DR K, HH N, L F, K B, JL R, Y V. Calcium channel blockade and preservation of renal graft function in cyclosporine-treated recipients: a prospective randomized placebo-controlled 2-year study. Transplantation. 2004(8). http://doi.org/10.1097/01.tp.0000137793.23371.42  1082. DR K, Y V. Time to reach tacrolimus maximum blood concentration,mean residence time, and acute renal allograft rejection: an open-label, prospective, pharmacokinetic study in adult recipients. Clin Ther. 2004(11). http://doi.org/10.1016/j.clinthera.2004.11.004  1083. Kwiatkowski DM, Goldstein SL, Cooper DS, Nelson DP, Morales DLS, Krawczeski CD. Peritoneal Dialysis vs Furosemide for prevention of fluid overload in infants after cardiac surgery a randomized clinical trial. Jama Pediatr. 2017;171(4):357-64. http://doi.org/10.1001/jamapediatrics.2016.4538  1084. Kwiatkowski DM, Goldstein SL, Cooper DS, Nelson DP, Morales DL, Krawczeski CD. Peritoneal dialysis vs. furosemide for the treatment of oliguria in infants after cardiopulmonary bypass. Circulation. 2015;132.  1085. Kwon TH, Kim YL, Cho DK. Ultrasound-guided cannulation of the femoral vein for acute haemodialysis access. Nephrol Dial Transpl. 1997;12(5):1009-12. http://doi.org/10.1093/ndt/12.5.1009  1086. Kwong AJ, Cheng X, Kwo PY, Wang U, Kim WR. Feasibility and efficacy of norepinephrine in the non-ICU setting for treatment of hepatorenal syndrome. Hepatology. 2020;72(1 SUPPL):1106A. http://doi.org/10.1002/hep.31579  1087. G L, F T, L B, JL D, D O, G M, et al. Nicotine patches in patients on mechanical ventilation for severe COVID-19: a randomized, double-blind, placebo-controlled, multicentre trial. Intens Care Med. 2022(7). http://doi.org/10.1007/s00134-022-06721-1  1088. SL L, JM B. Effect of furosemide on dialysis requirement following cadaveric kidney transplantation. J Urology. 1985(6). http://doi.org/10.1016/S0022-5347(17)49326-8  1089. Lachance K, White M, Denus SD. Risk factors for chronic renal insufficiency following cardiac transplantation. Ann Transpl. 2015;20:576-87. http://doi.org/10.12659/AOT.893788  1090. M L, O J, PM H, B G, H G, C F, et al. Acquired deficit of antithrombin and role of supplementation in septic patients during continuous veno-venous hemofiltration. ASAIO Journal (American Society for Artificial Internal Organs : 1992). 2008(1). http://doi.org/10.1097/MAT.0b013e31815cc3bf  1091. MR L, R S, B S, R K, F D, M A, et al. Randomized prospective trial of early steroid withdrawal compared with low-dose steroids in renal transplant recipients using serial protocol biopsies to assess efficacy and safety. Surgery. 2005(3). http://doi.org/10.1016/j.surg.2004.10.013  1092. Lai K, Lin G, Chen C, Xu Y. Development and Validation of a Predictive Model for Acute Kidney Injury in Sepsis Patients Based on Recursive Partition Analysis. J Intensive Care Med. 2024;39(5):465-76. http://doi.org/10.1177/08850666231214243  1093. H L, J J, DS S, FY A. Oseltamivir, an influenza neuraminidase inhibitor drug, does not affect the steady-state pharmacokinetic characteristics of cyclosporine, mycophenolate, or tacrolimus in adult renal transplant patients. Ther Drug Monit. 2011(6). http://doi.org/10.1097/FTD.0b013e3182399448  1094. Lamy A, Devereaux PJ, Prabhakaran D, Taggart DP, Hu S, Paolasso E, et al. Off-pump or on-pump coronary-artery bypass grafting at 30 days. New Engl J Med. 2012;366(16):1489-97. http://doi.org/10.1056/NEJMoa1200388  1095. W L, H S, S S, WD I, D A, G R, et al. The beneficial effect of human recombinant superoxide dismutase on acute and chronic rejection events in recipients of cadaveric renal transplants. Transplantation. 1994(2). http://doi.org/10.1097/00007890-199401001-00010  1096. G L, GG B, G M, T B, O F, G M, et al. Fenoldopam Reduces the Need for Renal Replacement Therapy and In-Hospital Death in Cardiovascular Surgery: a Meta-Analysis. J Cardiothor Vasc an. 2008(1). http://doi.org/10.1053/j.jvca.2007.07.015  1097. G L, L P, AA M, P G, A Z, E B, et al. A Multicenter Phase 2 Randomized Controlled Study on the Efficacy and Safety of Reparixin in the Treatment of Hospitalized Patients with COVID-19 Pneumonia. Infect Dis Ther. 2022(4). http://doi.org/10.1007/s40121-022-00644-6  1098. Landoni G, Bove T, Pasero D, Comis M, Orando S, Pinelli F, et al. Fenoldopam to prevent renal replacement therapy after cardiac surgery. Design of the FENO-HSR study. HSR Proc Intensive Care Cardiovasc Anesth. 2010;2(2):111-7.  1099. Langenecker SA, Felfernig M, Werba A, Mueller CM, Chiari A, Zimpfer M. Anticoagulation with prostacyclin and heparin during continuous venovenous hemofiltration. Crit Care Med. 1994;22(11):1774-81. http://doi.org/10.1097/00003246-199422110-00012  1100. J L, A V, T G, M R, F K. Pharmacokinetics of meropenem during intermittent and continuous intravenous application in patients treated by continuous renal replacement therapy. Intens Care Med. 2008(6). http://doi.org/10.1007/s00134-008-1034-7  1101. I L, AR M, P B, S P, E C, Z C, et al. Can tamoxifen prevent encapsulating sclerosing peritonitis in peritoneal dialysis? A single center experience and literature review. Nephrol Dial Transpl. 2017. http://doi.org/10.1093/ndt/gfx176  1102. CP L, J G, J M, Y V, F V, B B, et al. Belatacept-based regimens versus a cyclosporine a-based regimen in kidney transplant recipients: 2-year results from the benefit and benefit-EXT studies. Transplantation. 2010(12). http://doi.org/10.1097/TP.0b013e3181ff87cd  1103. Laubach CM, Janssen KP, Johannes W, Bogdanski R, Kapfer B. Cytokine and DAMP adsorption in septic acute kidney injury. Intens Care Med Exp. 2018;6. http://doi.org/10.1186/s40635-018-0201-6  1104. Lazarus B, Kotwal S, Gallagher M, Gray NA, Coggan S, Rogers K, et al. Effect of a Multifaceted Intervention on the Incidence of Hemodialysis Catheter Dysfunction in a National Stepped-Wedge Cluster Randomized Trial. Kidney Int Rep. 2023;8(10):1941-50. http://doi.org/10.1016/j.ekir.2023.07.013  1105. Le Bricon T, E T, M B, B B, C L, D E. Changes in plasma cystatin C after renal transplantation and acute rejection in adults. Clin Chem. 1999(12).  1106. Le Meur Y, E M, JP R, A T, M B, S D, et al. Oxyop study: first use in humans of the oxygen carrier (HEMO2life®) for organ preservation. Transpl Int. 2019. http://doi.org/10.1111/tri.13379  1107. Le PH, Yu PC, Patel P, Brateanu A, Imrey P, Rothberg MB. Association between antibiotic use and acute kidney injury in patients hospitalized with community-acquired pneumonia. J Gen Intern Med. 2019;34(2):S135. http://doi.org/10.1007/11606.1525-1497  1108. Leandro G. How to interpret a randomized controlled study stopped early. Intens Care Med. 2013;39(9):1642-3. http://doi.org/10.1007/s00134-013-3000-2  1109. Leano DR, Danguilan R, Arakama MH, Chua E, Apelin V, Alamillo PP. EFFICACY OF ADJUNCT HEMOPERFUSION COMPARED TO STANDARD MEDICAL THERAPY ON 28-DAY MORTALITY IN LEPTOSPIROSIS PATIENTS WITH RENAL FAILURE AND SHOCK: A SINGLE CENTER RANDOMIZED CONTROLLED TRIAL. Blood Purificat. 2023;52:4. http://doi.org/10.1159/000532122  1110. Leano DR, Danguilan R, Arakama M, Chua E, Apelin V, Alamillo PP. EFFICACY OF ADJUNCT HEMOPERFUSION VERSUS STANDARD MEDICAL THERAPY ON 28-DAYMORTALITY IN LEPTOSPIROSIS PATIENTS WITH RENAL FAILURE AND SHOCK. Nephrol Dial Transpl. 2023;38:i1119. http://doi.org/10.1093/ndt/gfad063c_3436  1111. M L, L M, OP R, M T, EP P. Bicarbonate dialysate for continuous renal replacement therapy in intensive care unit patients with acute renal failure. Am J Kidney Dis. 1995(6). http://doi.org/10.1016/0272-6386(95)90055-1  1112. Leblanc M, Fedak S, Mokris G, Paganini EP. Blood recirculation in temporary central catheters for acute hemodialysis. Clin Nephrol. 1996;45(5):315-9.  1113. A L, F M, MS U, De Serres SA, P D, S D, et al. Impact of dialysate calcium concentration on the progression of aortic stiffness in patients on haemodialysis. Nephrology, Dialysis, Transplantation. 2011(11). http://doi.org/10.1093/ndt/gfr138  1114. Lebon JS, Belda M, Laliberté E, Deschamps A, Couture P, Ayoub C, et al. An hydroxyethyl starch prime to improve perfusion in cardiac surgery. Canadian Journal of Anesthesia. 2014;61:S61-2. http://doi.org/10.1007/s12630-014-0239-1  1115. Y L, F B, M B, Le Meur Y, I E, O T, et al. Immunoprophylaxis with basiliximab compared with antithymocyte globulin in renal transplant patients receiving MMF-containing triple therapy. Am J Transplant. 2002(1). http://doi.org/10.1034/j.1600-6143.2002.020109.x  1116. Y L, A T, E T, M B, I E, PF W, et al. Efficacy and safety of early cyclosporine conversion to sirolimus with continued MMF-four-year results of the Postconcept study. Am J Transplant. 2011(8). http://doi.org/10.1111/j.1600-6143.2011.03637.x  1117. Y L, A T, O T, PF W, I E, E T, et al. Efficacy on renal function of early conversion from cyclosporine to sirolimus 3 months after renal transplantation: concept study. Am J Transplant. 2009(5). http://doi.org/10.1111/j.1600-6143.2009.02615.x  1118. A L, MG C, JC C, JF K, JP K. Effects of nonsteroidal anti‐inflammatory drugs on postoperative renal function in adults with normal renal function. Cochrane Db Syst Rev. 2007(2). http://doi.org/10.1002/14651858.CD002765.pub3  1119. SH L, CD K, KH H, BH C, MK J, DR L, et al. Low-dose mycophenolate mofetil in tablet form or capsule form combined with tacrolimus in the early period after kidney transplantation: a prospective randomized trial  . Clin Nephrol. 2016(12). http://doi.org/10.5414/CN108945  1120. SH L, JB P, CK O, MS K, SJ K, J H. Cyclosporine Sparing Effect of Enteric-Coated Mycophenolate Sodium in De Novo Kidney Transplantation. Yonsei Med J. 2017(1). http://doi.org/10.3349/ymj.2017.58.1.217  1121. Lee JY, Kim Y, Hyun YY, Lee KB, Yang J. WCN24-1782 THE IMPACT OF HIGHER PROTEIN PROVISION ON CONTINUOUS KIDNEY REPLACEMENT THERAPY. Kidney Int Rep. 2024;9(4):S535. http://doi.org/10.1016/j.ekir.2024.02.1124  1122. Lee P, Mason C, Ahmad-Hossain H, Lam KY, Germain E, Munjal I. Evaluation of a pharmacy directed vancomycin and monitoring pilot program at an academic pediatric hospital. Open Forum Infect Di. 2016;3. http://doi.org/10.1093/ofid/ofw172.1471  1123. C L, D C, M Z, L R, K B. Efficacy and Safety of Enteric-Coated Mycophenolate Sodium in De Novo Renal Transplant Recipients: pooled Data From Three 12-Month Multicenter, Open-Label, Prospective Studies. Transpl P. 2007(5). http://doi.org/10.1016/j.transproceed.2007.03.064  1124. Legrand M, Darmon M, Joannidis M, Zeni F, Payen D. Management of renal replacement therapy: An international survey among intensivists. Intens Care Med. 2011;37:S208.  1125. Lehr AR, Rached-D'Astous S, Parker M, McIntyre L, Sampson M, Hamid J, et al. Impact of balanced versus unbalanced fluid resuscitation on clinical outcomes in critically ill children: Protocol for a systematic review and meta-analysis. Syst Rev-London. 2019;8(1). http://doi.org/10.1186/s13643-019-1109-2  1126. Lehr A, Rached-D'Astous S, Hamid J, Parker M, McIntyre L, Sampson M, et al. Balanced versus unbalanced fluid in critically-ill children: Systematic review and meta-analysis. Pediatr Crit Care Me. 2021;22(SUPPL 1):116. http://doi.org/10.1097/01.pcc.0000739068.70964.52  1127. LeMaire SA, Jones MM, Conklin LD, Carter SA, Criddell MD, Wang XL, et al. Randomized comparison of cold blood and cold crystalloid renal perfusion for renal protection during thoracoabdominal aortic aneurysm repair. J Vasc Surg. 2009;49(1):11-9. http://doi.org/10.1016/j.jvs.2008.08.048  1128. P L, S HV, S G, X V, E R, J B, et al. High-Fidelity Simulation Nurse Training Reduces Unplanned Interruption of Continuous Renal Replacement Therapy Sessions in Critically Ill Patients: the SimHeR Randomized Controlled Trial. Anesth Analg. 2019(1). http://doi.org/10.1213/ANE.0000000000003581  1129. Lemkes JS, Janssens GN, Straaten HMOV, Elbers PW, van der Hoeven NW, Tijssen JGP, et al. Coronary angiography after cardiac arrest: Rationale and design of the COACT trial. Am Heart J. 2016;180:39-45. http://doi.org/10.1016/j.ahj.2016.06.025  1130. Lemkes JS, Janssens GN, Van Der Hoeven NW, Jewbali LSD, Dubois EA, Meuwissen M, et al. Coronary angiography after cardiac arrest without ST-segment elevation. New Engl J Med. 2019;380(15):1397-407. http://doi.org/10.1056/NEJMoa1816897  1131. KL L, MA S, H X, DC B. Long-term safety and efficacy of antithymocyte globulin induction: use of integrated national registry data to achieve ten-year follow-up of 10-10 Study participants. Trials. 2015. http://doi.org/10.1186/s13063-015-0891-y  1132. CD L, RG L, RR S. Parenteral essential amino acids in acute renal failure. Urology. 1975(2). http://doi.org/10.1016/0090-4295(75)90702-5  1133. Leoncini M, Toso A, Maioli M, Tropeano F, Villani S, Bellandi F. Early high-dose rosuvastatin for contrast-induced nephropathy prevention in acute coronary syndrome: Results from the PRATO-ACS study (protective effect of rosuvastatin and antiplatelet therapy on contrast-induced acute kidney injury and myocardial damage in patients with acute coronary syndrome). J Am Coll Cardiol. 2014;63(1):71-9. http://doi.org/10.1016/j.jacc.2013.04.105  1134. Lertjitbanjong P, Thongprayoon C, Cheungpasitporn W, Mao M, Kashani K. INCIDENCE AND IMPACT OF ACUTE KIDNEY INJURY IN PATIENTS RECEIVING EXTRACORPOREAL MEMBRANE OXYGENATION: A META-ANALYSIS. Chest. 2019;156(4):A1139. http://doi.org/10.1016/j.chest.2019.08.1040  1135. Leslie GD, Jacobs IG, Clarke GM. Proximally delivered dilute heparin does not improve circuit life in continuous venovenous haemodiafiltration. Intens Care Med. 1996;22(11):1261-4. http://doi.org/10.1007/BF01709346  1136. Levy MS, Creager MA. Revascularization versus medical therapy for renal-artery stenosis. The ASTRAL investigators. The New England Journal of Medicine 2009; 361: 1953-1962. Vasc Med. 2010;15(4):343-5. http://doi.org/10.1177/1358863X10372007  1137. M L, I N, AG S. HMG CoA reductase inhibitors (statins) for preventing acute kidney injury after surgical procedures requiring cardiac bypass. Cochrane Db Syst Rev. 2015(3). http://doi.org/10.1002/14651858.CD010480.pub2  1138. VD L, J M, S R, ZM D, G BJ, DM R, et al. Conversion of azathioprine to mycophenolate mofetil and chronic graft failure progression. Transpl P. 2005(2). http://doi.org/10.1016/j.transproceed.2004.12.142  1139. H L, SX W. Improvement of hypertension and LVH in maintenance hemodialysis patients treated with sustained-release isosorbide mononitrate. J Nephrol. 2011(2). http://doi.org/10.5301/jn.2011.6252  1140. P L, KJ H, DJ T, RE C, L K, D L, et al. Inflammatory biomarkers, glycemic variability, hypoglycemia, and renal transplant outcomes: results of a randomized controlled trial. Transplantation. 2014(6). http://doi.org/10.1097/TP.0000000000000123  1141. Li C, Wang H, Liu N, Jia M, Zhang H, Xi X, et al. Early negative fluid balance is associated with lower mortality after cardiovascular surgery. Perfusion (United Kingdom). 2018;33(8):630-7. http://doi.org/10.1177/0267659118780103  1142. Li WX, Chen HD, Wang XW, Zhao S, Chen XK, Zheng Y, et al. Predictive value of RIFLE classification on prognosis of critically ill patients with acute kidney injury treated with continuous renal replacement therapy. Chinese Med J-Peking. 2009;122(9):1020-5. http://doi.org/10.3760/cma.j.issn.0366-6999.2009.09.004  1143. Li X, Li X, Chi X, Luo G, Yuan D, Sun G, et al. Ulinastatin ameliorates acute kidney injury following liver transplantation in rats and humans. Exp Ther Med. 2015;9(2):411-6. http://doi.org/10.3892/etm.2014.2088  1144. Li Y, Xiong Y, Zhang H, Li J, Wang D, Chen W, et al. Ginkgo biloba extract EGb761 attenuates brain death-induced renal injury by inhibiting pro-inflammatory cytokines and the SAPK and JAK-STAT signalings. Sci Rep-Uk. 2017;7:45192. http://doi.org/10.1038/srep45192  1145. Liang S, Liu Y, Zhang B, Dun Y, Guo H, Qian X, et al. Postoperative Hepatic Dysfunction After Frozen Elephant Trunk for Type A Aortic Dissection. Front Cardiovasc Med. 2021;8. http://doi.org/10.3389/fcvm.2021.739606  1146. Libetta C, Esposito P, Sepe V, Rampino T, Zucchi M, Canevari M, et al. Acute kidney injury: effect of hemodialysis membrane on Hgf and recovery of renal function. Clin Biochem. 2013;46(1-2):103-8. http://doi.org/10.1016/j.clinbiochem.2012.10.014  1147. A L, T T, E M. Renaloutcomes in critically ill patients receiving propofolor midazolam a propensity scoring analysis. Nephrol Dial Transpl. 2015. http://doi.org/10.1093/ndt/gfv190.42  1148. Lim JY, Kim YS, Kim JB. Impact of 6% balanced hydroxyethyl starch following cardiopulmonary bypass on renal function: a retrospective study. J Cardiothorac Surg. 2020;15(1):237. http://doi.org/10.1186/s13019-020-01286-w  1149. Lim JY, Kim YW, Kang PJ, Park EJ, Kim JB, Choo SJ. Effect of saline-liberal vs restrictive administration on acute kidney injury after cardiac surgery. Crit Care Med. 2016;44(12):127. http://doi.org/10.1097/01.ccm.0000508881.46851.5c  1150. Lima EQ, Silva RG, Donadi ELS, Fernandes AB, Zanon JR, Pinto KRD, et al. Prevention of intradialytic hypotension in patients with acute kidney injury submitted to sustained low-efficiency dialysis. Renal Failure. 2012;34(10):1238-43. http://doi.org/10.3109/0886022X.2012.723581  1151. Limjoco LL, Teh-Yap JG, David A, Abrantes RO, Parilla KD, Cañones A, et al. T-tube in Moderation: T-tube Drainage versus Primary Closure after Open Common Bile Duct Exploration for Common Bile Duct Stone: A Single Institution Experience. Hpb. 2021;23:S373. http://doi.org/10.1016/j.hpb.2020.11.957  1152. G L, J L, X L. Down-regulation of miR-9 promotes VEGF expression in kidney, inhibits inflammatory factor release, and reduces acute kidney injury. Int J Clin Exp Med. 2020(9).  1153. J L, M G, R B, M D, K T, AY W. SOFA coagulation score and changes in platelet counts in severe acute kidney injury: analysis from the randomized evaluation of normal versus augmented level (RENAL) study. Nephrology (Carlton, Vic.). 2019(5). http://doi.org/10.1111/nep.13387  1154. J L, Z Q, X L, J X, Z W, Y G, et al. Retrograde inferior vena caval perfusion for total aortic arch replacement surgery: a randomized pilot study. Bmc Cardiovasc Disor. 2021(1). http://doi.org/10.1186/s12872-021-02002-9  1155. J L, B X, Q Z, L Y, F Z. Application of citric acid in continuous veno-venous hemofiltration of severe acute pancreatitis patients in ICU. Int J Clin Exp Med. 2020(9).  1156. Lin S, Lu W, Wang T, Wang Y, Leng X, Chi L, et al. Predictive model of acute kidney injury in critically ill patients with acute pancreatitis: a machine learning approach using the MIMIC-IV database. Renal Failure. 2024;46(1). http://doi.org/10.1080/0886022X.2024.2303395  1157. Lin WT, Lai CC, Chang SP, Wang JJ. Effects of early dialysis on the outcomes of critically ill patients with acute kidney injury: a systematic review and meta-analysis of randomized controlled trials. Sci Rep-Uk. 2019;9(1):18283. http://doi.org/10.1038/s41598-019-54777-9  1158. Lin YC. Effects of Sodium Bicarbonate on Clinical Outcomes in CKD Patients with Contrast-Associated AKI (CA-AKI): A Meta-Analysis on Randomized Controlled Trials (RCTs). J Am Soc Nephrol. 2023;34:433.  1159. Lin Y, Mok M, Harrison J, Battistella M, Farrell A, Leung M, et al. Use of sodium-glucose co-transporter 2 inhibitors in solid organ transplant recipients with pre-existing type 2 or post-transplantation diabetes mellitus: A systematic review. Transplant Rev-Orlan. 2023;37(1):100729. http://doi.org/10.1016/j.trre.2022.100729  1160. AM L, SJ N, JS R, PJ B, HB B, KAA F. Evacetrapib and cardiovascular outcomes in high-risk vascular disease. J Vasc Surg. 2017(4). http://doi.org/10.1016/j.jvs.2017.08.007  1161. K L, M R, JG H, HP H, BL P, BL J, et al. Effect of far infrared therapy on arteriovenous fistula maturation, survival and stenosis in hemodialysis patients, a randomized, controlled clinical trial: the FAITH on fistula trial. Bmc Nephrol. 2021(1). http://doi.org/10.1186/s12882-021-02476-x  1162. Lingegowda V, Van QC, Shimada M, Beaver TM, Dass B, Sood P, et al. Long-term outcome of patients treated with prophylactic nesiritide for the prevention of acute kidney injury following cardiovascular surgery. Clin Cardiol. 2010;33(4):217-21. http://doi.org/10.1002/clc.20750  1163. Lins RL, Elseviers MM, Van Der Niepen P, Hoste E, Malbrain ML, Damas P, et al. Intermittent versus continuous renal replacement therapy for acute kidney injury patients admitted to the intensive care unit: Results of a randomized clinical trial. Nephrol Dial Transpl. 2009;24(2):512-8. http://doi.org/10.1093/ndt/gfn560  1164. Litwak JJ, Cho N, Nguyen HB, Moussavi K, Bushell T. Vitamin C, hydrocortisone, and thiamine for the treatment of severe sepsis and septic shock: A retrospective analysis of real-world application. J Clin Med. 2019;8(4). http://doi.org/10.3390/jcm8040478  1165. Litwak J, Cho N, Moussavi K, Nguyen HB, Bushell T. Retrospective analysis of vitamin C, hydrocortisone, and thiamine in septic shock. Crit Care Med. 2019;47(1).  1166. B L, D L, J Q, P S. Combination of erythropoietin and alprostadil in treating acute kidney injury. Int J Clin Exp Med. 2016(9).  1167. BH L, HF L, Y L, SX Z, ML S. Clinical significance of dynamic monitoring of procalcitonin in guiding the use of antibiotics in patients with sepsis in ICU. Zhonghua Wei Zhong Bing Ji Jiu Yi Xue. 2013(11). http://doi.org/10.3760/cma.j.issn.2095-4352.2013.11.013  1168. F L, J Z, Y Z, L S, Y L, L H, et al. Acetate Ringer's solution versus 0.9% saline for septic patients: study protocol for a multi-center parallel controlled trial. Trials. 2021(1). http://doi.org/10.1186/s13063-020-05007-5  1169. KD L, BT T, M A, JS S, IS D, MA M, et al. Acute kidney injury in patients with acute lung injury: impact of fluid accumulation on classification of acute kidney injury and associated outcomes. Crit Care Med. 2011(12). http://doi.org/10.1097/CCM.0b013e318228234b  1170. Liu B, Sun YL. Protective effects of recombinant human erythropoietin combined with alprostadil in treatment of acute kidney injury. Hong Kong Journal of Nephrology. 2015;17(2):S98. http://doi.org/10.1016/j.hkjn.2015.09.070  1171. Liu C, Li M, Cao S, Wang J, Huang X, Zhong W. Effects of HV-CRRT on PCT, TNF-α, IL-4, IL-6, IL-8 and IL-10 in patients with pancreatitis complicated by acute renal failure. Exp Ther Med. 2017;14(4):3093-7. http://doi.org/10.3892/etm.2017.4843  1172. Liu H, Liu Y, Sun JK, Xu QL, Yan Y, Chen YM, et al. Extravascular lung water monitoring of renal replacement therapy in lung water scavenging for septic acute kidney injury. Int J Clin Exp Med. 2015;8(10):18907-16.  1173. Liu P, He YZ, Zhang XG, Li G, Wang HC, Lu XX, et al. [Studying the therapeutic effects of hemoperfusion with continuous venovenous hemofiltration on the patients with acute paraquat poisoning]. Zhonghua Lao Dong Wei Sheng Zhi Ye Bing Za Zhi = Zhonghua Laodong Weisheng Zhiyebing Zazhi = Chinese Journal of Industrial Hygiene and Occupational Diseases. 2011;29(4):266-9.  1174. Liu R, Nair D, Ix J, Moore DH, Bent S. N-acetylcysteine for the prevention of contrast-induced nephropathy: A systematic review and meta-analysis. J Gen Intern Med. 2005;20(2):193-200. http://doi.org/10.1111/j.1525-1497.2005.30323.x  1175. Liu Y, Hong D, Wang AY, Guo R, Smyth B, Liu J, et al. Effects of intravenous hydration on risk of contrast induced nephropathy and in-hospital mortality in STEMI patients undergoing primary percutaneous coronary intervention: a systematic review and meta-analysis of randomized controlled trials. Bmc Cardiovasc Disor. 2019;19(1):87. http://doi.org/10.1186/s12872-019-1054-y  1176. Liu Z, Jin Y, Feng C, Liu G, Wang Y, Zhao X, et al. Renoprotective Effect of Intraoperative Dexmedetomidine in Renal Transplantation. Comput Math Method M. 2022;2022:9275406. http://doi.org/10.1155/2022/9275406  1177. Liu Z, Zhao Y, Lei M, Zhao G, Li D, Sun R, et al. Remote Ischemic Preconditioning to Prevent Acute Kidney Injury After Cardiac Surgery: A Meta-Analysis of Randomized Controlled Trials. Front Cardiovasc Med. 2021;8. http://doi.org/10.3389/fcvm.2021.601470  1178. Llopart T, Lombardi R, Forselledo M, Andrade R. Acute renal failure in open heart surgery. Renal Failure. 1997;19(2):319-23. http://doi.org/10.3109/08860229709026296  1179. F L. Influence of membranes on morbidity. Nephrology, Dialysis, Transplantation. 1996(SUPPL. 2). http://doi.org/10.1093/ndt/11.supp2.116  1180. Lock AE, Gutierrez GC, Hand EO, Barthol CA, Attridge RL. Fludrocortisone Plus Hydrocortisone Versus Hydrocortisone Alone as Adjunctive Therapy in Septic Shock: A Retrospective Cohort Study. Ann Pharmacother. 2023;57(12):1375-88. http://doi.org/10.1177/10600280231164210  1181. TP L, DE L. Ceftaroline fosamil in the treatment of community-acquired bacterial pneumonia and acute bacterial skin and skin structure infections. Drugs. 2012(11). http://doi.org/10.2165/11635660-000000000-00000  1182. Lofgren L, Kuck K, Silverton N, Hoareau G. Urine oxygen tension monitoring as a measure of systemic perfusion. Anesth Analg. 2022;134(4 SUPPL):68-9.  1183. Logue J, Bell R, Hakeem A, Farid S, Upasani V, Attia M, et al. Favourable outcomes with hypothermic oxygenated machine perfusion in donation after circulatory death liver transplants - a single centre experience J. Walcott1. Transplantation. 2023;107(9):184.  1184. G L, O B, E B, M K, T P, L P, et al. Impact of renal failure on all-cause mortality and other outcomes in patients treated by percutaneous coronary intervention. Arch Cardiovasc Dis. 2015(11). http://doi.org/10.1016/j.acvd.2015.06.001  1185. Looareesuwan S, Wilairatana P, Vannaphan S, Wanaratana V, Wenisch C, Aikawa M, et al. Pentoxifylline as an ancillary treatment for severe falciparum malaria in Thailand. Am J Trop Med Hyg. 1998;58(3):348-53. http://doi.org/10.4269/ajtmh.1998.58.348  1186. Lopes RD, Macedo AVS, De Barros E Silva PGM, Moll-Bernardes RJ, Dos Santos TM, Mazza L, et al. Effect of Discontinuing vs Continuing Angiotensin-Converting Enzyme Inhibitors and Angiotensin II Receptor Blockers on Days Alive and out of the Hospital in Patients Admitted with COVID-19: A Randomized Clinical Trial. JAMA - Journal of the American Medical Association. 2021;325(3):254-64. http://doi.org/10.1001/jama.2020.25864  1187. Lopez-Solis RC, McCauley J, Mitric LA, Akavanheidari M, Hutnar A, Patzer JF. Renal insufficiency post liver transplant: Bound solute (albumin) dialysis. Liver Transplant. 2011;17:S247-8. http://doi.org/10.1002/lt.22457  1188. Louie JK, Yang S, Yen C, Acosta M, Schechter R, Uyeki TM. Use of intravenous peramivir for treatment of severe influenza A(H1N1)pdm09. Plos One. 2012;7(6). http://doi.org/10.1371/journal.pone.0040261  1189. C L, MJ N, F B, D C, P L, Y L, et al. Economic contribution of mofetil mycofenolate as preventive immunosuppressive treatment after renal transplantation from cadaver. Presse Medicale (Paris, France : 1983). 1996(33).  1190. R L, X Z, X C, X W, H L, L W, et al. Efficacy and safety of polysaccharide iron complex capsules compared with iron sucrose in hemodialysis patients: study protocol for a randomized, open-label, positive control, multicenter trial (IHOPE). Trials. 2021(1). http://doi.org/10.1186/s13063-021-05663-1  1191. TM L, SL Y, WZ W, JM T. Alemtuzumab induction therapy in highly sensitized kidney transplant recipients. Chinese Med J-Peking. 2011(5). http://doi.org/10.3760/cma.j.issn.0366-6999.2011.05.005  1192. XQ L. Clinical efficacy of TCM retention enema and sequential peritoneal dialysis on children with acute renal failure. Journal of Pediarics of Traditional Chinese Medicine [Zhong Yi ER Ke Za Zhi]. 2015(4).  1193. Y L, Q Z, J J. Development and validation of a prediction model for in-hospital mortality of patients with severe thrombocytopenia. Sci Rep-Uk. 2022(1). http://doi.org/10.1038/s41598-022-10438-y  1194. D L, O W, C S, P H, M K. Benefit of intrarenal gallopamil to acute renal failure. Internal Medicine (Tokyo, Japan). 1991(1).  1195. N L, N S. The haemodynamic effects of oXiris haemofilter in septic shock patients requiring renal support: a single-centre experience. Int J Artif Organs. 2021(1). http://doi.org/10.1177/0391398820917150  1196. Lumlertgul D, Wongmekiat O, Sirivanichai C, Hundagoon P, Keoplung M, Conger JD, et al. Intrarenal infusion of gallopamil in acute renal failure. A preliminary report. Drugs. 1991;42 Suppl 1:44-50. http://doi.org/10.2165/00003495-199100421-00009  1197. HT L, QM L, JJ T, YN Y, PH Z, ZD L, et al. Observation on hybrid bioartificial liver support systems in treating chronic severe hepatitis: a study of 60 cases. Zhonghua Gan Zang Bing Za Zhi [Chinese Journal of Hepatology]. 2006(3).  1198. Luo CC, Zhong YL, Qiao ZY, Li CN, Liu YM, Zheng J, et al. Development and validation of a nomogram for postoperative severe acute kidney injury in acute type A aortic dissection. J Geriatr Cardiol. 2022;19(10):734-42. http://doi.org/10.11909/j.issn.1671-5411.2022.10.003  1199. Luo XQ, Yan P, Duan SB, Kang YX, Deng YH, Liu Q, et al. Development and Validation of Machine Learning Models for Real-Time Mortality Prediction in Critically Ill Patients With Sepsis-Associated Acute Kidney Injury. Front Med-Lausanne. 2022;9. http://doi.org/10.3389/fmed.2022.853102  1200. Luo Y, Song Q, Li J, Fu S, Yu W, Shao X, et al. Effects of uric acid-lowering therapy (ULT) on renal outcomes in CKD patients with asymptomatic hyperuricemia: a systematic review and meta-analysis. Bmc Nephrol. 2024;25(1). http://doi.org/10.1186/s12882-024-03491-4  1201. C L, B Z, D Z, J L, L S, Z Z, et al. The effects of bicarbonated versus acetated Ringer's solutions on acid-base status and kidney injury following orthotopic liver transplantation: protocol for a single-centre, randomised controlled trial (The BETTER trial). Front Surg. 2022. http://doi.org/10.3389/fsurg.2022.1019570  1202. Lyndon WD, Wille KM, Tolwani AJ. Solute clearance in CRRT: Prescribed dose versus actual delivered dose. Nephrol Dial Transpl. 2012;27(3):952-6. http://doi.org/10.1093/ndt/gfr480  1203. Lyndon W, Wille K, Tolwani A. Solute clearance in CRRT: Comparing measured effluent volume to actual delivered dose. Am J Kidney Dis. 2011;57(4):A61. http://doi.org/10.1053/j.ajkd.2011.02.180  1204. M Turan ON, Driscol C, Cetin-Demirkaya B, Gabbay-Benziv R, Turan S, Kopelman J, et al. Early onset long term fetal indomethacin exposure does not cause neonatal renal complications. Reprod Sci. 2016;23(1):105A. http://doi.org/10.1177/1933719116641257  1205. Ma L, Guo J, Sun H, Li N, Lv M, Shang B. Nursing Countermeasures of Continuous Renal Replacement Treatment in End-Stage Renal Disease with Refractory Hypotension in the Context of Smart Health. Comput Math Method M. 2022;2022:2382458. http://doi.org/10.1155/2022/2382458  1206. Mabasa V, Khangura S, Wiens M, Reynolds S, Keenan S, Malyuk D, et al. Full vs. renal dosing of antibiotics in septic shock patients with acute renal failure: The fraser feasibility trial. Crit Care Med. 2009;37(12):A429. http://doi.org/10.1097/01.ccm.0000365439.11849.a2  1207. Macedo E, Karl B, Lee E, Mehta RL. A randomized trial of albumin infusion to prevent intradialytic hypotension in hospitalized hypoalbuminemic patients. Crit Care. 2021;25(1). http://doi.org/10.1186/s13054-020-03441-0  1208. S M, R D, A H, H H, K B, G M, et al. POS-038 ELECTRONIC ALERTS FOR ACUTE KIDNEY INJURY: EVALUATION OF EARLY RESPONSE BY A CLINICAL NURSE SPECIALIST. Kidney Int Rep. 2021(4). http://doi.org/10.1016/j.ekir.2021.03.044  1209. Mackinnon S, Aitken E, Ghita R, Clancy M. A comparison of the effects of oral vs. intravenous hydration on subclinical acute kidney injury in living kidney donors: a protocol of a randomised controlled trial. Bmc Nephrol. 2017;18(1):30. http://doi.org/10.1186/s12882-017-0447-3  1210. Madsen MB, Lange T, Hjortrup PB, Perner A. Immunoglobulin for necrotising soft tissue infections (INSTINCT): Protocol for a randomised trial. Dan Med J. 2016;63(7).  1211. KW M, B N, V P, de Zeeuw D, G F, N E, et al. Canagliflozin for Primary and Secondary Prevention of Cardiovascular Events: results From the CANVAS Program (Canagliflozin Cardiovascular Assessment Study). Circulation. 2018(4). http://doi.org/10.1161/CIRCULATIONAHA.117.032038  1212. Mahrous A, Elsammk M, Megahed M, Zytoun T. Renal effect of colloid versus crystalloid in septic neutropenic patients. Crit Care Med. 2013;41(12):A250. http://doi.org/10.1097/01.ccm.0000440233.20599.2e  1213. Mai Z, Tan Y, Zhu Y, Yang Z, Chen H, Cai S, et al. Effects of low-dose furosemide combined with aminophylline on the renal function in septic shock patients. Renal Failure. 2023;45(1). http://doi.org/10.1080/0886022X.2023.2185084  1214. R M, A K, SSR P, AK H, H T, G K, et al. A randomized-controlled trial comparing 20% albumin to plasmalyte in patients with cirrhosis and sepsis-induced hypotension. J Hepatol. 2022(3). http://doi.org/10.1016/j.jhep.2022.03.043  1215. R M, A S, M B, LG M, V S, P A, et al. High-volume plasma-exchange improves transplant-free survival in patients with acute liver failure-a prospective randomized controlled trial NCT02718079. Hepatology (Baltimore, Md.). 2018. http://doi.org/10.1002/hep.30256  1216. Maj G, Landoni G, Biondi-Zoccai G, Bignami E, Cabrini L, Buratti L, et al. Nesiritide and clinically relevant outcomes in cardiac surgery: A meta-analysis of randomized studies. Signa Vitae. 2011;6(2):17-23. http://doi.org/10.22514/SV62.102011.2  1217. SR M, CM K, WJ T, M H, DA T, KK T. Forced euvolemic diuresis with mannitol and furosemide for prevention of contrast-induced nephropathy in patients with CKD undergoing coronary angiography: a randomized controlled trial. Am J Kidney Dis. 2009(4). http://doi.org/10.1053/j.ajkd.2009.03.024  1218. J M, H A, WD I, A T, C D, R L, et al. Simultaneous pancreas-kidney transplantation: analysis of rejection. Transpl P. 2005(6). http://doi.org/10.1016/j.transproceed.2005.05.027  1219. Malhotra R, Katz R, Weiner DE, Levey AS, Cheung AK, Bostom AG, et al. Blood pressure, Chronic kidney disease progression, and kidney allograft failure in kidney transplant recipients: A secondary analysis of the favorit trial. Am J Hypertens. 2019;32(9):816-23. http://doi.org/10.1093/ajh/hpz095  1220. Malhotra R, Katz R, Weiner DE, Levey AS, Cheung AK, Bostom A, et al. Relationship of systolic blood pressure with GFR decline in kidney transplant recipients: The favorit trial. J Am Soc Nephrol. 2018;29:155. http://doi.org/10.1681/asn.2015060707  1221. Malki L, Gordien JB, Mariescu S, Rouault A, Joanes-Bouyau O, Janvier G, et al. Pharmacokinetics of Daptomycin during continuous Venovenous hemofiltration in septic patients. Int J Clin Pharm-Net. 2011;33(2):462. http://doi.org/10.1007/s11096-011-9481-6  1222. Malyszko J, Lukaszyk E, Glowinska I, Durlik M. Biomarkers of delayed graft function as a form of acute kidney injury in kidney transplantation. Sci Rep-Uk. 2015;5:11684. http://doi.org/10.1038/srep11684  1223. DA M, J A, A B, BA M, HT S, SM F, et al. Effect of Ramipril on Urinary Protein Excretion in Maintenance Renal Transplant Patients Converted to Sirolimus. Am J Transplant. 2015(12). http://doi.org/10.1111/ajt.13384  1224. AJ M, GL B. Angiotensin-Converting Enzyme Inhibitors and Angiotensin Receptor Blockers in Chronic Renal Disease: safety Issues. Semin Nephrol. 2004(2). http://doi.org/10.1016/j.semnephrol.2003.11.001  1225. HJ M, G A, DE W, H J, DC M, D J, et al. Multidisciplinary Medication Therapy Management and Hospital Readmission in Patients Undergoing Maintenance Dialysis: a Retrospective Cohort Study. Am J Kidney Dis. 2020(1). http://doi.org/10.1053/j.ajkd.2019.12.002  1226. Mann JFE, Ørsted DD, Brown-Frandsen K, Marso SP, Poulter NR, Rasmussen S, et al. Liraglutide and renal outcomes in type 2 diabetes. New Engl J Med. 2017;377(9):839-48. http://doi.org/10.1056/NEJMoa1616011  1227. Mann L, Eyck PT, Wu C, Story M, Jenigiri S, Patel J, et al. CVVHD results in longer filter life than pre-filter CVVH: Results of a quasi-randomized clinical trial. Plos One. 2023;18(1 January). http://doi.org/10.1371/journal.pone.0278550  1228. RB M, B A, PG S, AK M, AB F, N W, et al. Avoidance of CNI and steroids using belatacept-Results of the Clinical Trials in Organ Transplantation 16 trial. Am J Transplant. 2020(12). http://doi.org/10.1111/ajt.16152  1229. TM M, M C, P T, A S, S S, E M, et al. A 3-month, Multicenter, Randomized, Open-label Study to Evaluate the Impact on Wound Healing of the Early (vs Delayed) Introduction of Everolimus in De Novo Kidney Transplant Recipients, With a Follow-up Evaluation at 12 Months After Transplant (NEVERWOUND Study). Transplantation. 2020(2). http://doi.org/10.1097/TP.0000000000002851  1230. Mao HJ, Wang XY, Ying XM, Wang HJ, Shen X. Effect of bioartificial renal tubule on blood pressure, cytokine level and survival time in pig models of multiple organ dysfunction. Chinese Journal of Clinical Rehabilitation. 2004;8(24):5029-31.  1231. Mao HJ, Yu S, Yu XB, Zhang B, Zhang L, Xu XR, et al. Effects of coupled plasma filtration adsorption on immune function of patients with multiple organ dysfunction syndrome. Int J Artif Organs. 2009;32(1):31-8. http://doi.org/10.1177/039139880903200104  1232. Marathias KP, Vassili M, Robola A, Alivizatos PA, Palatianos GM, Geroulanos S, et al. Preoperative intravenous hydration confers renoprotection in patients with chronic kidney disease undergoing cardiac surgery. Artif Organs. 2006;30(8):615-21. http://doi.org/10.1111/j.1525-1594.2006.00270.x  1233. N M, JL S, GB S, M P, C K. Analysis of cytodiagnostic urinalysis findings in 77 patients with concurrent renal biopsies. Am J Kidney Dis. 1992(6). http://doi.org/10.1016/s0272-6386(12)70229-9  1234. Marenzi G, Bartorelli AL, Lauri G, Assanelli E, Grazi M, Campodonico J, et al. Continuous veno-venous hemofiltration for the treatment of contrast-induced acute renal failure after percutaneous coronary interventions. Catheter Cardio Inte. 2003;58(1):59-64. http://doi.org/10.1002/ccd.10373  1235. Marenzi G, Ferrari C, Assanelli E, Marana I, Lauri G, Campodonico J, et al. Furosemide-induced diuresis with matched hydration compared to standard hydration for contrast-induced nephropathy prevention: Preliminary results of the MYTHOS trial. Eur Heart J. 2009;30:526. http://doi.org/10.1093/eurheartj/ehp414  1236. C M, E A, N D, de Filippis JP, B L, F B. A randomized prospective study comparing low-dose OKT3 to low-dose ATG for the treatment of acute steroid-resistant rejection episodes in kidney transplant recipients. Transpl Int. 1998(3). http://doi.org/10.1007/s001470050133  1237. JF M, J L, SR K, L G, R V, J M, et al. Effect of repeated oral administrations of the oral adsorbent AST-120 on serum creatinine and other markers of renal function. A randomized controlled study in patients with chronic kidney disease. Am J Nephrol. 2006(2). http://doi.org/10.1159/000092242  1238. Marshall MR, Creamer JM, Foster M, Ma TM, Mann SL, Fiaccadori E, et al. Mortality rate comparison after switching from continuous to prolonged intermittent renal replacement for acute kidney injury in three intensive care units from different countries. Nephrol Dial Transpl. 2011;26(7):2169-75. http://doi.org/10.1093/ndt/gfq694  1239. WP M, J W, FJ L, NG D, le Roux CW. Metabolic Surgery to Treat Obesity in Diabetic Kidney Disease, Chronic Kidney Disease, and End-Stage Kidney Disease; What Are the Unanswered Questions? Front Endocrinol. 2020. http://doi.org/10.3389/fendo.2020.00289  1240. Martina Boscolo Berto M, Putzu A, Belletti A, Pasotti E, Cassina T, Moccetti T, et al. Prevention of contrast-induced acute kidney injury by furosemide with matched hydration in patients undergoing interventional procedures. A systematic review and Meta-Analysis of randomized trials. Eur J Heart Fail. 2017;19:102. http://doi.org/10.1002/ejhf.833  1241. F M, ER P, D P. Comparative pharmacological profile and cardiovascular impact of classic and emergent mineralocorticoid antagonist receptors. Rev Fed Argent Cardi. 2023.  1242. G M, PI M, LF B, MT M, CA A, LA J, et al. Low-dose Thymoglobulin vs Basiliximab Induction Therapy in Low-Risk Living Related Kidney Transplant Recipients: a Prospective Randomized Trial. Transpl P. 2021(3). http://doi.org/10.1016/j.transproceed.2020.01.054  1243. S M, L P, B T, L DS, R E, D M, et al. Efficacy and safety of everolimus with reduced tacrolimus in pediatric renal transplant recipients: design and baseline characteristics of cradle study. Pediatr Transplant. 2015.  1244. S M, C G, M H, de Molina FJ G, R C, S A. Preventive measures for development of contrast associated acute kidney injury in critical patients. Preliminary results of the nefrocon study. Intens Care Med Exp. 2015(no pagination). http://doi.org/10.1186/2197-425X-3-S1-A52  1245. Masich AM, Kalaria SN, Gonzales JP, Heil EL, Tata AL, Claeys KC, et al. Vancomycin Pharmacokinetics in Obese Patients with Sepsis or Septic Shock. Pharmacotherapy. 2020;40(3):211-20. http://doi.org/10.1002/phar.2367  1246. Masolitin SV, Protsenko DN, Tyurin DN, Magomedov MA, Kim TG, Grishina LA, et al. The Early Use of Selective Hemoadsorption Based on a Hyper-Crosslinked Styrene-Divinylbenzene Copolymer in Patients with Toxic Rhabdomyolysis Complicated by Acute Kidney Injury (Multicenter Randomized Clinical Trial). Obshchaya Reanimatologiya. 2022;18(6):22-9. http://doi.org/10.15360/1813-9779-2022-6-22-29  1247. J M, I V, P V, A H, V R, V B, et al. Prevention of contrast-induced acute kidney injury by theophylline in elderly patients with chronic kidney disease. Heart Vessels. 2010(6). http://doi.org/10.1007/s00380-010-0004-5  1248. TH M. A blinded, long-term, randomized multicenter study of mycophenolate mofetil in cadaveric renal transplantation: results at three years. Tricontinental Mycophenolate Mofetil Renal Transplantation Study Group. Transplantation. 1998(11). http://doi.org/10.1097/00007890-199806150-00007  1249. TH M, Van Buren C, BD K, K B, S H, JJ Z. A comparative study of sirolimus tablet versus oral solution for prophylaxis of acute renal allograft rejection. J Clin Pharmacol. 2006(1). http://doi.org/10.1177/0091270005282628  1250. Maxim K, Maria E, Svetlana A, Svetlana V, Zhanna K. Biomarkers in early diagnostic of acute kidney injury in patients with acute cardiorenal syndrome. Nephrol Dial Transpl. 2017;32:iii535-6. http://doi.org/10.1093/ndt/gfx167  1251. May HP, Griffin JM, Herges JR, Kashani KB, Kattah AG, Mara KC, et al. Comprehensive Acute Kidney Injury Survivor Care: Protocol for the Randomized Acute Kidney Injury in Care Transitions Pilot Trial. Jmir Res Protoc. 2023;12:e48109. http://doi.org/10.2196/48109  1252. T M, F P, M J. Recent developments in acute kidney injury: definition, biomarkers, subphenotypes, and management. Medizinische Klinik - Intensivmedizin Und Notfallmedizin. 2024. http://doi.org/10.1007/s00063-024-01142-y  1253. Mayumi K, Yamashita T, Hamasaki Y, Noiri E, Nangaku M, Yahagi N, et al. Impact of continuous renal replacement therapy intensity on septic acute kidney injury. Shock. 2016;45(2):133-8. http://doi.org/10.1097/SHK.0000000000000496  1254. Mazer CD, Whitlock RP, Fergusson DA, Thorpe KE, Shehata N. TRiCS III - An international multicenter randomized trial of transfusion requirements in cardiac surgery. Circulation. 2017;136:e449. http://doi.org/10.1161/CIR.0000000000000546  1255. McCausland FR, Asafu-Adjei J, Betensky RA, Palevsky PM, Waikar SS. Comparison of urine output among patients treated with more intensive versus less intensive RRT: Results from the acute renal failure trial network study. Clin J Am Soc Nephro. 2016;11(8):1335-42. http://doi.org/10.2215/CJN.10991015  1256. McCoy IE, Chertow GM. AKI—A Relevant Safety End Point? Am J Kidney Dis. 2020;75(4):508-12. http://doi.org/10.1053/j.ajkd.2019.11.010  1257. PA M. Prevention of cardiorenal syndromes. Contrib Nephrol. 2010. http://doi.org/10.1159/000313749  1258. McEvoy NL, Clarke JL, Mc Elvaney OJ, Mc Elvaney OF, Boland F, Hyland D, et al. A randomised, double-blind, placebo-controlled, pilot trial of intravenous plasma purified alpha-1 antitrypsin for SARS-CoV-2-induced Acute Respiratory Distress Syndrome: a structured summary of a study protocol for a randomised, controlled trial. Trials. 2021;22(1). http://doi.org/10.1186/s13063-021-05254-0  1259. P M, O D, D W, H H, K B, J GS. POS-335 COST-EFFECTIVENESS OF DAPAGLIFLOZIN AS A TREATMENT FOR CHRONIC KIDNEY DISEASE: a HEALTH-ECONOMIC ANALYSIS OF DAPA-CKD. Kidney Int Rep. 2021(4). http://doi.org/10.1016/j.ekir.2021.03.351  1260. McGee R, Nguyen B, Mitchell R, Craig J, Webster A. The number, coverage and quality of diagnostic test accuracy studies in nephrology. Nephrology. 2010;15:78. http://doi.org/10.1111/j.1440-1797.2010.01377.x  1261. McIntyre L, Fergusson DA, Rowe B, Cook DJ, Arabi Y, Bagshaw SM, et al. The PRECISE RCT: Evolution of an Early Septic Shock Fluid Resuscitation Trial. Transfus Med Rev. 2012;26(4):333-41. http://doi.org/10.1016/j.tmrv.2011.11.003  1262. McMahon BA, Jaggi K, Kraus ES, Novick TK, Menez S, Desai N, et al. The Impact of Donor Cannabinoid Intoxication on Outcomes of Kidney Transplantation. J Am Soc Nephrol. 2016;27:628A-629A.  1263. McNicholas B, Akcan Arikan A, Ostermann M. Quality of life after acute kidney injury. Curr Opin Crit Care. 2023;29(6):566-79. http://doi.org/10.1097/MCC.0000000000001090  1264. M M, L V, AM H, G R, S V, Y F, et al. Randomized controlled trial of mineralocorticoid receptor blockade in children with chronic kidney allograft nephropathy. Clin J Am Soc Nephro. 2017(8). http://doi.org/10.2215/CJN.05300516  1265. Meena J, Thomas CC, Kumar J, Mathew G, Bagga A. Biomarkers for prediction of acute kidney injury in pediatric patients: a systematic review and meta-analysis of diagnostic test accuracy studies. Pediatr Nephrol. 2023;38(10):3241-51. http://doi.org/10.1007/s00467-023-05891-4  1266. M M, M K, C W, D K, S K, T S, et al. Regional citrate versus systemic heparin anticoagulation for continuous renal replacement therapy in critically ill patients with acute kidney injury (RICH) trial: study protocol for a multicentre, randomised controlled trial. Bmj Open. 2019(1). http://doi.org/10.1136/bmjopen-2018-024411  1267. Meersch M, Schmidt C, Hoffmeier A, Van Aken H, Wempe C, Gerss J, et al. Prevention of cardiac surgery-associated AKI by implementing the KDIGO guidelines in high risk patients identified by biomarkers: the PrevAKI randomized controlled trial. Intens Care Med. 2017;43(11):1551-61. http://doi.org/10.1007/s00134-016-4670-3  1268. Mehdi M, Nicolas M, Mariat C. Economic impact of two modalities of cvvhdf on nurses working load. Nephrol Dial Transpl. 2012;27:ii361-2. http://doi.org/10.1093/ndt/gfs235  1269. Mehta RL, Mcdonald B, Gabbai FB, Pahl M, Pascual MTA, Farkas A, et al. A randomized clinical trial of continuous versus intermittent dialysis for acute renal failure. Kidney Int. 2001;60(3):1154-63. http://doi.org/10.1046/j.1523-1755.2001.0600031154.x  1270. Meloni C, Morosetti M, Meschini L, Palombo G, Latorre PC, Taccone-Gallucci M, et al. Blood purification procedures for acute renal failure: convenient strategy related to clinical conditions. Blood Purificat. 1996;14(3):242-8. http://doi.org/10.1159/000170267  1271. RA M, AI R, D S, de Castro-Filho A, T J, A DJ, et al. Prognostic value of renal function in patients with aortic stenosis treated with transcatheter aortic valve replacement. Catheter Cardio Inte. 2017(3). http://doi.org/10.1002/ccd.26693  1272. Menez S, Coca SG, Moledina DG, Wen Y, Chan L, Thiessen-Philbrook H, et al. Evaluation of Plasma Biomarkers to Predict Major Adverse Kidney Events in Hospitalized Patients With COVID-19. Am J Kidney Dis. 2023;82(3):322-32. http://doi.org/10.1053/j.ajkd.2023.03.010  1273. Menezes RRPP, Sampaio TL, Martins AMC, de Sousa Alves R, Magalhães EP. Prescription Drug Overdose, Depression, and Other Mental Disorders in the Context of Kidney Disease. Contrib Nephrol. 2021;199:155-61. http://doi.org/10.1159/000517700  1274. Meng JB, Lai ZZ, Xu XJ, Ji CL, Hu MH, Zhang G. Effects of early continuous venovenous hemofiltration on E-selectin, hemodynamic stability, and ventilatory function in patients with septic-shock-induced acute respiratory distress syndrome. Biomed Res Int. 2016;2016. http://doi.org/10.1155/2016/7463130  1275. FS M, M G, I V, F F, PL M, D U, et al. Cost-effectiveness analysis of Vaborem in Carbapenem-resistant Enterobacterales (CRE) -Klebsiella pneumoniae infections in Italy. Health Econ Rev. 2021(1). http://doi.org/10.1186/s13561-021-00341-z  1276. Menon S, Li Q, Van De Sompele DR, Doud AJ, Vong KL, Bourdrez H, et al. Early Identification of the Need for CRRT in Children: A Machine Learning Approach. J Am Soc Nephrol. 2023;34:94.  1277. TP M, KE W, van Brunschot DMD O, Van der Vliet DJA, MM R, MC W. Ischaemic preconditioning for the reduction of renal ischaemia reperfusion injury. Cochrane Db Syst Rev. 2017(3). http://doi.org/10.1002/14651858.CD010777.pub2  1278. RM M, ML H, JS M, HW S, DE S, RJ T. Randomized, prospective trial of mycophenolate mofetil versus azathioprine for prevention of acute renal allograft rejection after simultaneous kidney-pancreas transplantation. Transplantation. 2000(1).  1279. P M, F B, C D, D M, G C, D D, et al. Lower incidence of chronic allograft nephropathy at 1 year post-transplantation in patients treated with mycophenolate mofetil. Am J Transplant. 2004(11). http://doi.org/10.1111/j.1600-6143.2004.00533.x  1280. Meshram HS, Kute VB, Patel H, Banerjee S, Navadiya V, Desai S, et al. Feasibility and safety of remdesivir in SARS-CoV2 infected renal transplant recipients: A retrospective cohort from a developing nation. Transpl Infect Dis. 2021;23(4). http://doi.org/10.1111/tid.13629  1281. Metz D, Stiller M, Silber RE, Kroll H, Hofmann HS, Diez C. Prophylactic intraaortic balloon pumping in high-risk cardiac surgery patients. Medizinische Klinik - Intensivmedizin Und Notfallmedizin. 2011;106(2):125-31. http://doi.org/10.1007/s00063-011-0048-z  1282. M M, E P, G S, HJ U, K S, E B, et al. Therapeutic use of the natriuretic peptide ularitide in acute renal failure. Renal Failure. 1999(1). http://doi.org/10.3109/08860229909066972  1283. C MG, S K, P T, Y D, M Y, P J, et al. Safety and Tolerability of CSL112, a Reconstituted, Infusible, Plasma-Derived Apolipoprotein A-I, After Acute Myocardial Infarction: the AEGIS-I Trial (ApoA-I Event Reducing in Ischemic Syndromes I). Circulation. 2016(24). http://doi.org/10.1161/CIRCULATIONAHA.116.025687  1284. H M, A T, M N, P R, S ZV, S S, et al. The early start of hemoperfusion decreases the mortality rate among severe COVID-19 patients: a preliminary study. Hemodialysis International. International Symposium On Home Hemodialysis. 2022(2). http://doi.org/10.1111/hdi.12982  1285. Milanez T, Ocvirk J, Arnol M, Jaimes EA. Onco-nephrology: The current concept and future perspectives. Libri Oncologici. 2023;51:5-6.  1286. J M, R M, JD P, SC J. Safety and efficacy of tacrolimus in combination with mycophenolate mofetil (MMF) in cadaveric renal transplant recipients. FK506/MMF Dose-Ranging Kidney Transplant Study Group. Transplantation. 2000(5). http://doi.org/10.1097/00007890-200003150-00035  1287. Miller B. A study of cell therapy for subjects with AKI who are receiving continuous renal replacement therapy. J Am Soc Nephrol. 2017;28:700.  1288. Millis MA, Poli EC, Sherman SK, Dahdaleh FS, Kamm A, Turaga KK. Fluid optimal strategy during cytoreductive surgery (CRS) and hyperthermic intraperitoneal chemotherapy (HIPEC). Pleura Peritoneum. 2018;3:sA136-7. http://doi.org/10.1515/pap-2018-7016  1289. G M, L G, A C, O G, M G, D C, et al. Once-every-2-weeks and once-weekly epoetin beta regimens: equivalency in hemodialyzed patients. Am J Kidney Dis. 2006(3). http://doi.org/10.1053/j.ajkd.2006.05.030  1290. Mirhosseini SM, Fakhri M, Asadollahi S, Ahmadi ZH, Farokhi FR, Boloursaz MR, et al. Continuous renal replacement therapy versus furosemide for management of kidney impairment in heart transplant recipients with volume overload. Interact Cardiov Th. 2013;16(3):314-20. http://doi.org/10.1093/icvts/ivs492  1291. Mistry K. Dialysis disequilibrium syndrome prevention and management. 2019;12:69-77. http://doi.org/10.2147/IJNRD.S165925  1292. Mital D, Petruccione M, Brown J, Vasquez S, Wheaton S, Johnson W, et al. Sequential surgical strategy for heart-kidney transplantation. Am J Transplant. 2017;17:646. http://doi.org/10.1111/ajt.14306  1293. Mitra S, Ling RR, Tan CS, Shekar K, MacLaren G, Ramanathan K. Concurrent Use of Renal Replacement Therapy during Extracorporeal Membrane Oxygenation Support: A Systematic Review and Meta-Analysis. J Clin Med. 2021;10(2). http://doi.org/10.3390/jcm10020241  1294. L M, S SS, von Zur Mühlen B, B J, JM H, C B, et al. Renal function three years after early conversion from a calcineurin inhibitor to everolimus: results from a randomized trial in kidney transplantation. Transpl Int. 2015(1). http://doi.org/10.1111/tri.12437  1295. Modarresi A, Nafar M, Sahraei Z, Salamzadeh J, Chaibakhsh S, Ziaie S, et al. N-acetylcysteine decreases urinary level of neutrophil gelatinase-associated lipocalin in deceased-donor renal transplant recipients: a randomized clinical trial. Biomarkers. 2018;23(6):589-96. http://doi.org/10.1080/1354750X.2018.1468823  1296. C M, AC N, M G, E H, B M, J M. Long-term results with single pediatric donor kidney transplants in adult recipients. J Urology. 1996(3).  1297. C M, JM S, MH M, J T, van Gelder F, BP N, et al. Machine perfusion or cold storage in deceased-donor kidney transplantation. New Engl J Med. 2009(1). http://doi.org/10.1056/NEJMoa0802289  1298. Mohamed ZU, Varghese CT, Sudhakar A, Kumar L, Gopalakrishnan U, Balakrishnan D, et al. Prostaglandins for adult liver transplanted recipients. Cochrane Db Syst Rev. 2023;2023(8). http://doi.org/10.1002/14651858.CD006006.pub3  1299. Mohammad Al-Shibani BI, Kahaleq MAA, Abosaooda M, Mosa AK, Abdulhussein MA, Hadi NR. Potential nephroprotective effect of valsartan in renal ischemia reperfusion injury role of nf-kbp65 pathway in rat. International Journal of Pharmaceutical Research. 2020;12(1):928-36. http://doi.org/10.31838/IJPR/2020.12.01.176  1300. N M, A R, A W, N G, S D, D S, et al. Sodium bicarbonate for kidney transplant recipients with metabolic acidosis in Switzerland: a multicentre, randomised, single-blind, placebo-controlled, phase 3 trial. Lancet (London, England). 2023(10376). http://doi.org/10.1016/S0140-6736(22)02606-X  1301. DG M, IE H, H T, PP R, FL W, B S, et al. Performance of Serum Creatinine and Kidney Injury Biomarkers for Diagnosing Histologic Acute Tubular Injury. Am J Kidney Dis. 2017(6). http://doi.org/10.1053/j.ajkd.2017.06.031  1302. Molinari L, Heskia F, Peerapornratana S, Ronco C, Guzzi L, Toback S, et al. Limiting Acute Kidney Injury Progression In Sepsis: Study Protocol and Trial Simulation. Crit Care Med. 2021;49(10):1706-16. http://doi.org/10.1097/CCM.0000000000005061  1303. Monteiro A. Impact of contrast-induced acute kidney injury (CI-AKI) on long term outcomes of patients undergoing arterial procedures. Nephrol Dial Transpl. 2016;31:i147. http://doi.org/10.1093/ndt/gfw162.2  1304. N M, AC W, A R, J Z, M CB, J P. Steroid avoidance or withdrawal for pancreas and pancreas with kidney transplant recipients. Cochrane Db Syst Rev. 2014(9). http://doi.org/10.1002/14651858.CD007669.pub2  1305. Montomoli M, Molina P, Vizcaíno B, Beltrán S, Castro C, González-Moya M, et al. Effectiveness of the palindrome tunneled catheter in achieving an adequate diffusive and convective dialysis doses: A 3 year single-center prospective study. Blood Purificat. 2015;40(1):12-3. http://doi.org/10.1159/000431262  1306. P M, F T, M E, S L, T L, B V, et al. Risk factors of clinical failure during treatment of postoperative peritonitis: results of the DURAPOP trial. Intens Care Med Exp. 2017(2). http://doi.org/10.1186/s40635-017-0151-4  1307. MB M, A K, DE U, U E. Arterial stiffness depends on serum ionized calcium levels during dialysis with regional citrate anticoagulation. Artif Organs. 2013(5). http://doi.org/10.1111/aor.12037  1308. E M, T P, S G, P S, S E, S G, et al. Comparison of short and long-term renal function in liver transplant patients receiving cyclosporin or FK 506. Transpl Int. 1994. http://doi.org/10.1111/j.1432-2277.1994.tb01314.x  1309. Morabito S, Pistolesi V, Tritapepe L, Vitaliano E, Zeppilli L, Polistena F, et al. Continuous venovenous hemodiafiltration with a low citrate dose regional anticoagulation protocol and a phosphate-containing solution: effects on acid-base status and phosphate supplementation needs. Bmc Nephrol. 2013;14:232. http://doi.org/10.1186/1471-2369-14-232  1310. Moradi H, Igarashi M, Soohoo M, Streja E, Rhee C, Said HM, et al. Arachidonoylglycerol, a Major Activator of the Endocannabinoid System, Is Significantly Increased in Patients with Kidney Disease. J Am Soc Nephrol. 2015;26:549A-550A.  1311. C M, NA B, J B, AF B, D M. Effects of acute resistance exercise on acyl-ghrelin and obestatin levels in hemodialysis patients: a pilot study. Renal Failure. 2015(10). http://doi.org/10.3109/0886022X.2015.1087805  1312. LE M, JA O, R P, LA M, Y G, H J, et al. Spironolactone reduces oxidative stress in living donor kidney transplantation: a randomized controlled trial. American Journal of Physiology. Renal Physiology. 2019(3). http://doi.org/10.1152/ajprenal.00606.2018  1313. G M, MC Y, S S, I A, M H. Effect of routine insertion of a double-J stent after living related renal transplantation. Transpl P. 2005(2). http://doi.org/10.1016/j.transproceed.2005.01.083  1314. S M, C S, C M, T S, L L, G B, et al. A simple, safe and effective citrate anticoagulation protocol for the genius dialysis system in acute renal failure. Nephron. Clinical Practice. 2004(1). http://doi.org/10.1159/000079925  1315. Morgera S, Haase M, Kuss T, Vargas-Hein O, Zuckermann-Becker H, Melzer C, et al. Pilot study on the effects of high cutoff hemofiltration on the need for norepinephrine in septic patients with acute renal failure. Crit Care Med. 2006;34(8):2099-104. http://doi.org/10.1097/01.CCM.0000229147.50592.F9  1316. N M, C M, A S, G T, M C, F O, et al. Early intra-aortic balloon pump in acute decompensated heart failure complicated by cardiogenic shock: rationale and design of the randomized Altshock-2 trial. Am Heart J. 2021. http://doi.org/10.1016/j.ahj.2020.11.017  1317. Morillas JA, Marco Canosa F, Srinivas P, Asadi T, Calabrese C, Rajendram P, et al. Tocilizumab therapy in 5 solid and composite tissue transplant recipients with early ARDS due to SARS-CoV-2. Am J Transplant. 2020;20(11):3191-7. http://doi.org/10.1111/ajt.16080  1318. GJ M, DA O, SC S, WA J. Sequential changes in plasma selenium concentration after cadaveric renal transplantation. Brit J Surg. 2004(3). http://doi.org/10.1002/bjs.4427  1319. Mortazavi M, Ghasemi F, Seirafian S, Taheri S, Mansourian M, Abbasi S. The incidence rate of acute kidney injury and risk factors among the intensive care unit inpatients. Journal of Isfahan Medical School. 2021;39(613):86-92. http://doi.org/10.22122/jims.v39i613.13476  1320. Morzywołek P, Steen J, Van Biesen W, Decruyenaere J, Vansteelandt S. On estimation and cross-validation of dynamic treatment regimes with competing risks. Stat Med. 2022;41(26):5258-75. http://doi.org/10.1002/sim.9568  1321. Morzywołek P, Steen J, Vansteelandt S, Decruyenaere J, Sterckx S, Van Biesen W. Timing of dialysis in acute kidney injury using routinely collected data and dynamic treatment regimes. Crit Care. 2022;26(1). http://doi.org/10.1186/s13054-022-04252-1  1322. FH M, H B, N H, JN B. Prednisolone treatment in acute interstitial nephritis (PRAISE) - protocol for the randomized controlled trial. Bmc Nephrol. 2021(1). http://doi.org/10.1186/s12882-021-02372-4  1323. Moskowitz A, Andersen LW, Cocchi MN, Karlsson M, Patel PV, Donnino MW. Thiamine as a renal protective agent in septic shock a secondary analysis of a randomized, double-blind, placebo-controlled trial. Ann Am Thorac Soc. 2017;14(5):737-41. http://doi.org/10.1513/AnnalsATS.201608-656BC  1324. Moskowitz A, Berg KM, Grossestreuer AV, Balaji L, Liu X, Cocchi MN, et al. Thiamine for Renal Protection in Septic Shock (TRPSS): A Randomized, Placebo-controlled, Clinical Trial. Am J Resp Crit Care. 2023;208(5):570-8. http://doi.org/10.1164/rccm.202301-0034OC  1325. G M, M G, L A, O V, P M, G T, et al. Incidence of Posttransplantation Diabetes Mellitus in De Novo Kidney Transplant Recipients Receiving Prolonged-Release Tacrolimus-Based Immunosuppression With 2 Different Corticosteroid Minimization Strategies: ADVANCE, A Randomized Controlled Trial. Transplantation. 2017(8). http://doi.org/10.1097/TP.0000000000001453  1326. G M, L R, Ch L. Assessment of two strategies of neoral administration, early versus delayed, on renal function and efficacy in de novo renal transplant patients receiving myfortic, steroids, and anti-IL2R antibodies: 12-month results of a randomized, multicentre, open, prospective controlled study. Transpl P. 2005(2). http://doi.org/10.1016/j.transproceed.2004.12.263  1327. JS M, Jd H, van Zwet EW, MJ M, J D, de Fijter JW. Randomized trial comparing late concentration-controlled calcineurin inhibitor or mycophenolate mofetil withdrawal. Transplantation. 2012(9). http://doi.org/10.1097/TP.0b013e31824ad60a  1328. K M, B F, L P, J P, D L, A K, et al. 36-month follow-up of 75 renal allograft recipients treated with steroids, tacrolimus, and azathioprine or mycophenolate mofetil. Transpl P. 2003(6). http://doi.org/10.1016/s0041-1345(03)00815-7  1329. B M, H S, P S, R M, L S, KH O. A prospective randomized trial comparing traditional and fast-track patient care in elective open infrarenal aneurysm repair. World J Surg. 2009(3). http://doi.org/10.1007/s00268-008-9892-2  1330. F M, HH N, D DC, S S, AJ Z, K B. The efficacy and safety of cyclosporine reduction in de novo renal allograft patients receiving sirolimus and corticosteroids: results from an open-label comparative study. Transpl Int. 2014(2). http://doi.org/10.1111/tri.12228  1331. MA M, AK G, SN M, SA H, M K, A K, et al. Terlipressin in combination with albumin as a therapy for hepatorenal syndrome in patients aged 65 years or older. Ann Hepatol. 2023(5). http://doi.org/10.1016/j.aohep.2023.101126  1332. Mukhopadhyay P. MON-238 EVALUATION AND OUTCOME OF PREGNANCY INDUCED ACUTE KIDNEY INJURY AND SURROGATE PREDICTOR OF PATIENT NEEDED RENAL REPLACEMENT THERAPY. Kidney Int Rep. 2019;4(7):S398. http://doi.org/10.1016/j.ekir.2019.05.1040  1333. S M, H T, F O, R W, U K, G R, et al. FTY720/cyclosporine regimens in de novo renal transplantation: a 1-year dose-finding study. Am J Transplant. 2006(8). http://doi.org/10.1111/j.1600-6143.2006.01404.x  1334. Mulla H, Peek G, Upton D, Lin E, Loubani M. Plasma aluminum levels during sucralfate prophylaxis for stress ulceration in critically ill patients on continuous venovenous hemofiltration: A randomized, controlled trial. Crit Care Med. 2001;29(2):267-71. http://doi.org/10.1097/00003246-200102000-00008  1335. Muller M, Lefebvre F, Harlay ML, Glady L, Becker G, Muller C, et al. Impact of intravenous lidocaine on clinical outcomes of patients with ARDS during COVID-19 pandemia (LidoCovid): A structured summary of a study protocol for a randomised controlled trial. Trials. 2021;22(1):131. http://doi.org/10.1186/s13063-021-05095-x  1336. Müller RB, Haase N, Lange T, Wetterslev J, Perner A. Acute kidney injury with hydroxyethyl starch 130/0.42 in severe sepsis. Acta Anaesth Scand. 2015;59(3):329-36. http://doi.org/10.1111/aas.12453  1337. Muñoz B, Schobel SA, Lisboa FA, Khatri V, Grey SF, Dente CJ, et al. Clinical risk factors and inflammatory biomarkers of post-traumatic acute kidney injury in combat patients. Surgery (United States). 2020;168(4):662-70. http://doi.org/10.1016/j.surg.2020.04.064  1338. M M, S M, AJ F, DN J, HI S, G R, et al. Renal replacement treatment initiation with twice-weekly versus thrice-weekly haemodialysis in patients with incident dialysis-dependent kidney disease: rationale and design of the TWOPLUS pilot clinical trial. Bmj Open. 2021(5). http://doi.org/10.1136/bmjopen-2020-047596  1339. R M, X W, C K, F P, PM P, M U, et al. Associations between Intensity of RRT, Inflammatory Mediators, and Outcomes. Clin J Am Soc Nephro. 2015(6). http://doi.org/10.2215/CJN.04560514  1340. Murugan R, Kerti SJ, Chang CCH, Gallagher M, Neto AS, Clermont G, et al. Association between Net Ultrafiltration Rate and Renal Recovery among Critically Ill Adults with Acute Kidney Injury Receiving Continuous Renal Replacement Therapy: An Observational Cohort Study. Blood Purificat. 2022;51(5):397-409. http://doi.org/10.1159/000517281  1341. GS M, SS D, NN Y, GJ G, BA P, RI H, et al. Association of IL6 and IL10 with renal dysfunction and the use of haemofiltration during cardiopulmonary bypass. Eur J Cardio-Thorac. 2009(3). http://doi.org/10.1016/j.ejcts.2008.10.010  1342. M M, M M, Y Y, A B, B E, H F, et al. Electronic Alerts for Acute Kidney Injury Amelioration (ELAIA-1): a completely electronic, multicentre, randomised controlled trial: design and rationale. Bmj Open. 2019(5). http://doi.org/10.1136/bmjopen-2018-025117  1343. J M. Which fluid should i use for resuscitation? Intern Med J. 2010. http://doi.org/10.1111/j.1445-5994.2010.02186.x  1344. Myles PS, Bellomo R, Corcoran T, Forbes A, Peyton P, Story D, et al. Restrictive versus liberal fluid therapy for major abdominal surgery. New Engl J Med. 2018;378(24):2263-74. http://doi.org/10.1056/NEJMoa1801601  1345. Mylvaganam R, Szabo A, Cooper C, Makowski C, Wunderink R, Kruser J. PRAGMATIC EXPERIENCE IN THE USE OF ANGIOTENSIN II FOR THE TREATMENT OF SHOCK: A SINGLE-CENTER COHORT STUDY. Chest. 2020;158(4):A2414-20. http://doi.org/10.1016/j.chest.2020.09.012  1346. Z N, N A, RM R, ZU Z. Contrast Induced Nephropathy In High Risk Patients - Myth Or Reality. Journal of Ayub Medical College, Abbottabad. 2021(4).  1347. Nadeau-Fredette AC, Bouchard J. Fluid Management and Use of Diuretics in Acute Kidney Injury. Adv Chronic Kidney D. 2013;20(1):45-55. http://doi.org/10.1053/j.ackd.2012.09.005  1348. M N, L H, E L, K C, De Wever L, F C, et al. Intrarenal resistive index after renal transplantation. New Engl J Med. 2013(19). http://doi.org/10.1056/NEJMoa1301064  1349. M N, O S, M B, RB E, V D, W H, et al. Subclinical inflammation and chronic renal allograft injury in a randomized trial on steroid avoidance in pediatric kidney transplantation. Am J Transplant. 2012(10). http://doi.org/10.1111/j.1600-6143.2012.04144.x  1350. Nagamani S, Vinodh MP, Shoma RV. The rifle score in ARF: A unicenter observational study on the outcome of ARF in the critically ill population. Intens Care Med. 2010;36:S287. http://doi.org/10.1007/s00134-010-2000-8  1351. Naik D, Jebasingh FK, Thomas N, Raveendran S, Raj Pallapati SC, Prakash JJ, et al. Necrotizing soft tissue infection of the upper extremities in patients with diabetes mellitus in a tertiary care center-a retrospective study. Diabetes and Metabolic Syndrome: Clinical Research and Reviews. 2020;14(5):1071-5. http://doi.org/10.1016/j.dsx.2020.05.032  1352. R N, J M, Van Ophem D. A large, prospective, randomized, open-label, multicentre study of corticosteroid withdrawal in SPK transplantation: a 3-year report. Nephrology, Dialysis, Transplantation. 2005. http://doi.org/10.1093/ndt/gfh1081  1353. Namdev H. SODIUM BICARBONATE INFUSION: TO PREVENT CARDIAC SURGERY-ASSOCIATED ACUTE KIDNEY INJURY. International Journal of Academic Medicine and Pharmacy. 2022;4(5):27-30. http://doi.org/10.47009/jamp.2022.4.5.7  1354. Nana P, Kouvelos G, Brotis A, Spanos K, Giannoukas A, Matsagkas M. The effect of endovascular aneurysm repair on renal function in patients treated for abdominal aortic aneurysm. Curr Pharm Design. 2019;25(44):4675-85. http://doi.org/10.2174/1381612825666191129094923  1355. BJ N, M S, CH P, A S. The Clinical and Pathologic Phenotype of Antibody-Mediated Vascular Rejection Diagnosed Using Arterial C4d Immunoperoxidase. Kidney Int Rep. 2022(7). http://doi.org/10.1016/j.ekir.2022.04.097  1356. B N, S L, IR H, A L, JR J. Reduction of acute renal allograft rejection by daclizumab. Daclizumab Double Therapy Study Group. Transplantation. 1999(1). http://doi.org/10.1097/00007890-199901150-00019  1357. B N, CP S, M A, K A, G A, KE B, et al. BIOKID: randomized controlled trial comparing bicarbonate and lactate buffer in biocompatible peritoneal dialysis solutions in children. Bmc Nephrol. 2004. http://doi.org/10.1186/1471-2369-5-14  1358. Nayak S, Prabhahar A, Bahuguna P, Gupta KL, Kohli HS, Ramachandran R. SUN-003 ONLINE HEMODIAFILTRATION (POST-DILUTION) AS COMPARED TO HIGH FLUX HEMODIALYSIS DOES NOT IMPROVE OUTCOMES IN COMMUNITY ACQUIRED AKI WITH SIRS- A RANDOMISED CONTROLLED TRIAL. Kidney Int Rep. 2020;5(3):S205-6. http://doi.org/10.1016/j.ekir.2020.02.525  1359. NCT. Angiotensin II Blockade for Chronic Allograft Nephropathy. Https://Clinicaltrials.Gov/Show/NCT00067990. 2003.  1360. NCT. Acute Renal Failure Trial Network (ATN) Study. Https://Clinicaltrials.Gov/Show/NCT00076219. 2004.  1361. NCT. The Use of Nesiritide in Thoracic Aneurysm Repair to Prevent Acute Renal Failure. Https://Clinicaltrials.Gov/Show/NCT00110201. 2005.  1362. NCT. Donor Dopamine and Initial Graft Function. Https://Clinicaltrials.Gov/Show/NCT00115115. 2005.  1363. NCT. Trial of Plasma Exchange for Acute Renal Failure at the Onset of Myeloma. Https://Clinicaltrials.Gov/Show/NCT00120263. 2005.  1364. NCT. An Investigation of N-acetylcysteine and Fenoldopam as Renal Protection Agents for Cardiac Surgery. Https://Clinicaltrials.Gov/Show/NCT00122018. 2005.  1365. NCT. Ultrafiltration Versus Intravenous (IV) Diuretics for Patients Hospitalized for Acute Decompensated Heart Failure: (UNLOAD). Https://Clinicaltrials.Gov/Show/NCT00124137. 2005.  1366. NCT. Pilot Study on the Use of Sirolimus to Treat Chronic Allograft Nephropathy in Children After Kidney Transplant. Https://Clinicaltrials.Gov/Show/NCT00188955. 2005.  1367. NCT. Citrate Versus Heparin Anticoagulation in Continuous Venovenous Hemofiltration. Https://Clinicaltrials.Gov/Show/NCT00209378. 2005.  1368. NCT. Haemofiltration Study : IVOIRE (hIgh VOlume in Intensive Care). Https://Clinicaltrials.Gov/Show/NCT00241228. 2005.  1369. NCT. Safety and Efficacy of the Use of Regional Anticoagulation With Citrate in Continuous Venovenous Hemofiltration. Https://Clinicaltrials.Gov/Show/NCT00286273. 2006.  1370. NCT. Vasodilators and Anti-Oxidant Therapy in Early ATN. Https://Clinicaltrials.Gov/Show/NCT00286403. 2006.  1371. NCT. Is Spironolactone Safe and Effective in the Treatment of Cardiovascular Disease in Mild Chronic Renal Failure? Https://Clinicaltrials.Gov/Show/NCT00291720. 2006.  1372. NCT. Forced Diuresis Versus Observation in Resolving Renal Failure After Haemofiltration in Critically Ill Patients. Https://Clinicaltrials.Gov/Show/NCT00298454. 2006.  1373. NCT. Dopamine and Norepinephrine in Shock Patients. Https://Clinicaltrials.Gov/Show/NCT00314704. 2006.  1374. NCT. Intermittent Versus Continuous Renal Replacement Therapy for Acute Renal Failure. Https://Clinicaltrials.Gov/Show/NCT00322933. 2006.  1375. NCT. Super High-Flux - High Volume Dialysis in Sepsis-Induced Acute Renal Failure. Https://Clinicaltrials.Gov/Show/NCT00333593. 2006.  1376. NCT. Study of N-Acetylcysteine (NAC) and Continuous Renal Replacement Therapy (CRRT) for the Treatment of Rhabdomyolysis. Https://Clinicaltrials.Gov/Show/NCT00391911. 2006.  1377. NCT. Randomized Trial of Pulsatile vs Non Pulsatile Perfusion on Short Term Changes in Kidney Function Using an Intra-Aortic Ballooon Pump During Cardioplegic Arrest in Patients Undergoing Myocardial Reperfusion. Https://Clinicaltrials.Gov/Show/NCT00454428. 2007.  1378. NCT. a Multifaceted Program for Improving Quality of Care in ICU. Https://Clinicaltrials.Gov/Show/NCT00461461. 2007.  1379. NCT. Coronary Artery Bypass Surgery (CABG) Off or On Pump Revascularization Study. Https://Clinicaltrials.Gov/Show/NCT00463294. 2007.  1380. NCT. Intraoperative Fluid Management Based on Arterial Pulse Pressure Variation During High-Risk Surgery. Https://Clinicaltrials.Gov/Show/NCT00479011. 2007.  1381. NCT. Vitamin D and Carboxy PTH Fragments in Coronary Calcification. Https://Clinicaltrials.Gov/Show/NCT00502268. 2007.  1382. NCT. Open-Label Study To Evaluate the Safety and Efficacy of the Renal Assist Device In Patients With Acute Renal Failure. Https://Clinicaltrials.Gov/Show/NCT00511407. 2007.  1383. NCT. Calcineurin Inhibitor Minimisation in Renal Transplant Recipients With Stable Allograft Function. Https://Clinicaltrials.Gov/Show/NCT00541814. 2007.  1384. NCT. High Dose CVVHDF Compared to Standard Dose CVVHDF. Https://Clinicaltrials.Gov/Show/NCT00561431. 2007.  1385. NCT. CNI-free de Novo Protocol in Patients Undergoing Liver Transplantation With Renal Impairment. Https://Clinicaltrials.Gov/Show/NCT00604357. 2008.  1386. NCT. Sodium Bicarbonate in Cardiac Surgery Study. Https://Clinicaltrials.Gov/Show/NCT00672334. 2008.  1387. NCT. Volume Replacement With Albumin in Severe Sepsis. Https://Clinicaltrials.Gov/Show/NCT00707122. 2008.  1388. NCT. Angiotensin in Septic Kidney Injury Trial. Https://Clinicaltrials.Gov/Show/NCT00711789. 2008.  1389. NCT. Preop Hemodialysis or Intraop Ultrafiltration for Patients With Severe Renal Dysfunction Undergoing Open Heart Surgery. Https://Clinicaltrials.Gov/Show/NCT00720967. 2008.  1390. NCT. Renoprotective Effects of Fluid Prophylaxis Strategies for Contrast Induced Nephropathy (CIN). Https://Clinicaltrials.Gov/Show/NCT00749827. 2008.  1391. NCT. Short-term Atorvastatin's Effect on Acute Kidney Injury Following Cardiac Surgery. Https://Clinicaltrials.Gov/Show/NCT00791648. 2008.  1392. NCT. Saving Residual Renal Function Among Haemodialysis Patients Receiving Irbesartan. Https://Clinicaltrials.Gov/Show/NCT00791830. 2008.  1393. NCT. Argatroban Versus Lepirudin in Critically Ill Patients. Https://Clinicaltrials.Gov/Show/NCT00798525. 2008.  1394. NCT. Fluid Resuscitation in Early Septic Shock. Https://Clinicaltrials.Gov/Show/NCT00819416. 2009.  1395. NCT. Bicarbonate in Cardiac Surgery. Https://Clinicaltrials.Gov/Show/NCT00878956. 2009.  1396. NCT. Prevention of Acute Kidney Injury in Cardiac Surgery Patients. Https://Clinicaltrials.Gov/Show/NCT00921518. 2009.  1397. NCT. Assessing the Impact of Two Methods of Continuous Veno-venous Hemodiafiltration on Time Nursing Work in Intensive Care. Https://Clinicaltrials.Gov/Show/NCT00993733. 2009.  1398. NCT. N-acetyl-cysteine (NAC) and Kidney Graft Function. Https://Clinicaltrials.Gov/Show/NCT00998972. 2009.  1399. NCT. Place of Antibiotics in the Postoperative Acute Lithiasic Cholecystitis. Https://Clinicaltrials.Gov/Show/NCT01015417. 2009.  1400. NCT. Contrast Agent-associated Nephrotoxicity in Intensive Care Unit Patients. Https://Clinicaltrials.Gov/Show/NCT01017796. 2009.  1401. NCT. Target Temperature Management After Cardiac Arrest. Https://Clinicaltrials.Gov/Show/NCT01020916. 2009.  1402. NCT. CNI-free "Bottom"-up Immunosuppression in Patients Undergoing Liver Transplantation. Https://Clinicaltrials.Gov/Show/NCT01023542. 2009.  1403. NCT. Continuous Venovenous Hemofiltration Versus Continuous Venovenuous Hemodialysis. Https://Clinicaltrials.Gov/Show/NCT01062984. 2010.  1404. NCT. Intra-Renal Therapy of Diuretic Unresponsive Acute Kidney Injury. Https://Clinicaltrials.Gov/Show/NCT01073189. 2010.  1405. NCT. RenalGuard System and Contrast Media. Https://Clinicaltrials.Gov/Show/NCT01098032. 2010.  1406. NCT. Effects of High-dose Intravenous Selenium (Selenase®) in Adult Patients Subjected to Elective All-cause Heart Surgery. Https://Clinicaltrials.Gov/Show/NCT01141556. 2010.  1407. NCT. Hemofiltration in Burns: RESCUE (Randomized Controlled Evaluation of Hemofiltration in Adult Burn Patients With Septic Shock and Acute Renal Failure). Https://Clinicaltrials.Gov/Show/NCT01213914. 2010.  1408. NCT. Phase IIb Study of MP4OX in Traumatic Hemorrhagic Shock Patients. Https://Clinicaltrials.Gov/Show/NCT01262196. 2010.  1409. NCT. Neutrophil Gelatinase-associated Lipocalin (NGAL) and Contrast Media Induced Nephropathy. Https://Clinicaltrials.Gov/Show/NCT01292317. 2011.  1410. NCT. A Comparison of Dilute Versus Concentrated Heparin for CRRT Anticoagulation. Https://Clinicaltrials.Gov/Show/NCT01318811. 2011.  1411. NCT. Efficacy of Certican® in Combination With Myfortic® in Renal. Https://Clinicaltrials.Gov/Show/NCT01399242. 2011.  1412. NCT. Efficacy Study of a Selective Cytopheretic Device (SCD) in Patients With Acute Kidney Injury. Https://Clinicaltrials.Gov/Show/NCT01400893. 2011.  1413. NCT. The Efficacity of Hemodiafiltration Versus Hemofiltration for Renal Insufficiency During Intensive Care. Https://Clinicaltrials.Gov/Show/NCT01403220. 2011.  1414. NCT. Perfusion - Pressure - Creatinine Trial. Https://Clinicaltrials.Gov/Show/NCT01408420. 2011.  1415. NCT. Value of 25 mcg Cortrosyn Stimulation Test. Https://Clinicaltrials.Gov/Show/NCT01428336. 2011.  1416. NCT. Personalized Mean Arterial Pressure Management on Renal Function During Septic Shock. Https://Clinicaltrials.Gov/Show/NCT01473498. 2011.  1417. NCT. Enhancing Dialysis Adequacy: effects of Intradialytic Exercise. Https://Clinicaltrials.Gov/Show/NCT01481688. 2011.  1418. NCT. Nafamostat Efficacy and Safety in Critically Ill Patients(NICE). Https://Clinicaltrials.Gov/Show/NCT01486485. 2011.  1419. NCT. Remote Ischemic Preconditioning in Neurological Death Organ Donors. Https://Clinicaltrials.Gov/Show/NCT01515072. 2011.  1420. NCT. Hyperglycemia in Renal Transplantation. Https://Clinicaltrials.Gov/Show/NCT01643382. 2012.  1421. NCT. Randomised Control Trial for Improving Functional Outcome From Stroke in End Stage Renal Disease Patients. Https://Clinicaltrials.Gov/Show/NCT01656213. 2012.  1422. NCT. Peritoneal Dialysis vs Furosemide for Acute Kidney Injury After Cardiopulmonary Bypass. Https://Clinicaltrials.Gov/Show/NCT01709227. 2012.  1423. NCT. L-ornithine L-aspartate in Overt Hepatic Encephalopathy. Https://Clinicaltrials.Gov/Show/NCT01722578. 2012.  1424. NCT. House Calls and Decision Support: improving Access to Live Donor Transplantation. Https://Clinicaltrials.Gov/Show/NCT01786525. 2013.  1425. NCT. Fluid Management Based on Pleth Variability Index (PVI) Monitoring During High-risk Surgery. Https://Clinicaltrials.Gov/Show/NCT01788293. 2013.  1426. NCT. Effects of AN69 ST Hemofilter on Coagulation During Continuous Renal Replacement Therapy in Critically Ill. Https://Clinicaltrials.Gov/Show/NCT01823484. 2013.  1427. NCT. Nesiritide and Renal Function After the Total Artificial Heart. Https://Clinicaltrials.Gov/Show/NCT01836809. 2013.  1428. NCT. Prevention of Vitamin D Deficiency Following Pediatric CHD Surgery: a Phase II Dose Evaluation Randomized Controlled Trial Comparing Usual Care With a High Dose Pre-operative Supplementation Regimen Based on the Institute of Medicine Daily Upper Tolerable Intake Level. Https://Clinicaltrials.Gov/Show/NCT01838447. 2013.  1429. NCT. Citrate Versus Heparin Anticoagulation: effect on Molecules Clearances. Https://Clinicaltrials.Gov/Show/NCT01839578. 2013.  1430. NCT. Safety and Efficacy Study of Intravenous Immunoglobulin to Treat Japanese Encephalitis. Https://Clinicaltrials.Gov/Show/NCT01856205. 2013.  1431. NCT. Atorvastatin Versus Rosuvastatin on Contrast Induced Acute Kidney Injury (PRATO-ACS 2). Https://Clinicaltrials.Gov/Show/NCT01870804. 2013.  1432. NCT. Bortezomib in Late Antibody-mediated Kidney Transplant Rejection. Https://Clinicaltrials.Gov/Show/NCT01873157. 2013.  1433. NCT. The Effectiveness of Smoking Cessation in Prediabetic Smokers. Https://Clinicaltrials.Gov/Ct2/Show/NCT01926041. 2013.  1434. NCT. ISCHEMIA-Chronic Kidney Disease Trial. Https://Clinicaltrials.Gov/Show/NCT01985360. 2013.  1435. NCT. Very Early veRsus Deferred Invasive Evaluation Using Computerized Tomography in Patients With Acute Coronary Syndromes. Https://Clinicaltrials.Gov/Show/NCT02061891. 2014.  1436. NCT. Preventing Early Dialysis Starts. Https://Clinicaltrials.Gov/Show/NCT02183987. 2014.  1437. NCT. Magnesium Balance of Citrate-based Continuous Venovenous Hemofiltration, Effect of Citrate Dose. Https://Clinicaltrials.Gov/Show/NCT02194569. 2014.  1438. NCT. Personalized Versus Standard Hydration for Prevention of CI-AKI: a Randomized Trial With Bioimpedance Analysis. Https://Clinicaltrials.Gov/Show/NCT02225431. 2014.  1439. NCT. The Goal dIrected perFusion Trial in Cardiac Surgery. Https://Clinicaltrials.Gov/Show/NCT02250131. 2014.  1440. NCT. Vasculopathic Injury and Plasma as Endothelial Rescue - OCTAplas Trial (EudraCT no. 2014-000452-28). Https://Clinicaltrials.Gov/Show/NCT02253082. 2014.  1441. NCT. Lung Water by Ultrasound Guided Treatment in Hemodialysis Patients (The Lust Study). Https://Clinicaltrials.Gov/Show/NCT02310061. 2014.  1442. NCT. Acute Kidney Outreach to Reduce Deterioration and Death (AKORDD). Https://Clinicaltrials.Gov/Show/NCT02398682. 2015.  1443. NCT. Central Venous Pressure Guided Hydration Prevention for Contrast-Induced Nephropathy. Https://Clinicaltrials.Gov/Show/NCT02405377. 2015.  1444. NCT. The Routine Use of SSRI's at the Initiation of End-stage Renal Disease Treatment (RoSIE). Https://Clinicaltrials.Gov/Show/NCT02407821. 2015.  1445. NCT. Short Term Spironolactone for Prevention of Acute Kidney Injury After Cardiac Surgery. Https://Clinicaltrials.Gov/Show/NCT02417896. 2015.  1446. NCT. Nephrologist Follow-up Versus Usual Care After an Acute Kidney Injury Hospitalization. Https://Clinicaltrials.Gov/Show/NCT02483039. 2015.  1447. NCT. NAC, NaHCO3 and NS Prophylaxis for CTPA in the ED on Suspicion of PE: a Randomized Controlled Trial. Https://Clinicaltrials.Gov/Show/NCT02483143. 2015.  1448. NCT. Effects of Intraoperative Goal-Directed Fluid Therapy on the Incidence of Postoperative Complications. Https://Clinicaltrials.Gov/Show/NCT02507557. 2015.  1449. NCT. Albumin in Cardiac Surgery. Https://Clinicaltrials.Gov/Show/NCT02560519. 2015.  1450. NCT. Comparative Effectiveness of 30%TSC and Heparin Lock Solution in Hemodialysis Catheters. Https://Clinicaltrials.Gov/Show/NCT02563041. 2015.  1451. NCT. Effect of BM-MSCs on Early Graft Function Recovery After DCD Kidney Transplant. Https://Clinicaltrials.Gov/Show/NCT02563366. 2015.  1452. NCT. The Clinical Study of Improving the Thirst and Hypotension of Hemodialysis Patients. Https://Clinicaltrials.Gov/Show/NCT02583802. 2015.  1453. NCT. Early Endoscopy for Acute Upper Gastrointestinal Bleeding in Acute Coronary Syndrome Patients. Https://Clinicaltrials.Gov/Show/NCT02618980. 2015.  1454. NCT. Low Sodium Dialysate and Ambulatory Blood Pressure Measurement Parameters. Https://Clinicaltrials.Gov/Show/NCT02621450. 2015.  1455. NCT. Anti-Thrombotic Strategy After Trans-Aortic Valve Implantation for Aortic Stenosis. Https://Clinicaltrials.Gov/Show/NCT02664649. 2015.  1456. NCT. Comparison of Outcomes and Access to Care for Heart Failure Trial. Https://Clinicaltrials.Gov/Show/NCT02674438. 2016.  1457. NCT. ACCESS HD: comparing Catheters to Fistulas in Elderly Patients Starting Hemodialysis. Https://Clinicaltrials.Gov/Show/NCT02675569. 2016.  1458. NCT. The Effect of REcombinant Human Thrombopoietin (rhTPO) on Sepsis Patients With aCUte Severe thrombocytopEnia. Https://Clinicaltrials.Gov/Ct2/Show/NCT02707497. 2016.  1459. NCT. Effects of Dexmedetomidine on Microcirculation of Kidney Transplant Recipient. Https://Clinicaltrials.Gov/Show/NCT02707809. 2016.  1460. NCT. Adequate Hydration Therapy Combined With Intravenous Infusion of Isosorbide Dinitrate Prevention for CIN. Https://Clinicaltrials.Gov/Show/NCT02718521. 2016.  1461. NCT. Insulin Therapy Reduce Post-Operative Inflammatory Response After Curative Colorectal Cancer Resection: randomization Controlled Trial. Https://Clinicaltrials.Gov/Show/NCT02746432. 2016.  1462. NCT. The Effect of Simultaneous Renal Replacement Therapy on Extracorporeal Membrane Oxygenation Support for Cardiogenic Shock Patients. Https://Clinicaltrials.Gov/Show/NCT02870946. 2016.  1463. NCT. Effect of Remote Ischemic Preconditioning on the Incidence of Acute Kidney Injury in Patients Undergoing Coronary Artery Bypass Graft Surgery. Https://Clinicaltrials.Gov/Show/NCT02981680. 2016.  1464. NCT. NATIENS: optimal Management and Mechanisms of SJS/TEN. Https://Clinicaltrials.Gov/Ct2/Show/NCT02987257. 2016.  1465. NCT. Effect of Dry-weight Probing Guided by Lung-Ultrasound on Ambulatory Blood Pressure and Arterial Stiffness in Hemodialysis Patients (LUST Sub-Study). Https://Clinicaltrials.Gov/Show/NCT03058874. 2017.  1466. NCT. Early Goal Directed Therapy Using a Physiological Holistic View. The ANDROMEDA-SHOCK Study. Https://Clinicaltrials.Gov/Show/NCT03078712. 2017.  1467. NCT. Multi-center Trial of Goal-directed Fluid Management Based on Pulse Pressure Variation Monitoring. Https://Clinicaltrials.Gov/Show/NCT03128190. 2017.  1468. NCT. Incremental Hemodialysis as a Starting Way of Renal Replacement Therapy. Https://Clinicaltrials.Gov/Ct2/Show/NCT03302546. 2017.  1469. NCT. Recovery After Dialysis-Requiring Acute Kidney Injury. Https://Clinicaltrials.Gov/Show/NCT03305549. 2017.  1470. NCT. Evaluation of Early Association of Terlipressin and Norepinephrine During Septic Shock; the TerliNor Study. Https://Clinicaltrials.Gov/Show/NCT03336814. 2017.  1471. NCT. RCT of Efficacy of Amoxicillin Over Ampicillin on Severe Pneumonia. Https://Clinicaltrials.Gov/Show/NCT03369093. 2017.  1472. NCT. Erythropoietin Role in Acute Kidney Injury. Https://Clinicaltrials.Gov/Show/NCT03401710. 2017.  1473. NCT. Metabolic Resuscitation Using Ascorbic Acid, Thiamine, and Glucocorticoids in Sepsis. Https://Clinicaltrials.Gov/Show/NCT03422159. 2018.  1474. NCT. Endotoxins and Cytokines Removal During Continuous Hemofiltration With oXiris™. Https://Clinicaltrials.Gov/Show/NCT03426943. 2018.  1475. NCT. Prevention of Acute Kidney Injury by N-Acetylcystein in Patients Undergone Cardiac Valve Replacement. Https://Clinicaltrials.Gov/Show/NCT03440268. 2018.  1476. NCT. Effects of Vitamin C Administration on Extravascular Lung Water in Patients With Severe Features of Preeclampsia. Https://Clinicaltrials.Gov/Show/NCT03451266. 2018.  1477. NCT. The Effect of Vitamin E-coated Polysulfone Membrane on Oxidative Stress, Inflammation and Monocytes in Critically Ill Patients in CRRT. Https://Clinicaltrials.Gov/Show/NCT03489759. 2018.  1478. NCT. Tunneled Dialysis Catheters Versus Non-tunneled Dialysis Catheters as First-line for Renal Replacement Therapy in the ICU. Https://Clinicaltrials.Gov/Show/NCT03496935. 2018.  1479. NCT. Evaluation of Routinely Measured Patient-reported Outcomes in Hemodialysis Care. Https://Clinicaltrials.Gov/Show/NCT03535922. 2018.  1480. NCT. Study on the Optimal Strategy for Acute-on-chronic Liver Failure With Integrative Treatment. Https://Clinicaltrials.Gov/Show/NCT03577938. 2018.  1481. NCT. Outcomes of the Use of Sodium Bicarbonate (8.4%) Solution as a Catheter Lock Solution to Prevent Hemodialysis Catheter Loss Due to Lumen Clot Formation. Https://Clinicaltrials.Gov/Show/NCT03627884. 2018.  1482. NCT. The Impact of Different Citrate Concentrations as Locking Solutions on Development of Biofilm and Function of Hemodialysis Catheters. Https://Clinicaltrials.Gov/Show/NCT03683563. 2018.  1483. NCT. Melatonin for Renal Protection in Patients Receiving Polymyxin B. Https://Clinicaltrials.Gov/Show/NCT03725267. 2018.  1484. NCT. Preventing Acute Kidney Injury (AKI) in Pediatric Patients. Https://Clinicaltrials.Gov/Show/NCT03897335. 2019.  1485. NCT. Treatment of Renal Stones With Frankincense (Luban). Https://Clinicaltrials.Gov/Show/NCT03924596. 2019.  1486. NCT. Vacuum Assisted Closure Versus On-demand Relaparotomy in Patients With Fecal or Diffuse Peritonitis. Https://Clinicaltrials.Gov/Show/NCT03932461. 2019.  1487. NCT. Acute Post-cardiac Surgery Renal Failure: prevention Through Individualized Intensive Hemodynamic Management. Https://Clinicaltrials.Gov/Ct2/Show/NCT04005105. 2019.  1488. NCT. Sodium Bicarbonate for the Treatment of Severe Metabolic Acidosis With Moderate or Severe Acute Kidney Injury in ICU. Https://Clinicaltrials.Gov/Ct2/Show/NCT04010630. 2019.  1489. NCT. Impact of Handover of Anesthesia Care on Adverse Postoperative Outcomes. Https://Clinicaltrials.Gov/Show/NCT04016454. 2019.  1490. NCT. Prospective, Controlled, Randomized Multicentric Study on the Management of the Vesical Catheter in Colon Surgery. Https://Clinicaltrials.Gov/Show/NCT04070898. 2019.  1491. NCT. Continuous Renal Replacement Therapy for Acute Kidney Injury by Cooling Blood. Https://Clinicaltrials.Gov/Ct2/Show/NCT04103307. 2019.  1492. NCT. Renal Physiology During Continuous Renal Replacement Therapy. Https://Clinicaltrials.Gov/Ct2/Show/NCT04114747. 2019.  1493. NCT. PDE5i Use in Renal Transplant Recipients. Https://Clinicaltrials.Gov/Ct2/Show/NCT04122105. 2019.  1494. NCT. Expressive Writing in Kidney Transplant Patients. Https://Clinicaltrials.Gov/Show/NCT04143178. 2019.  1495. NCT. ecco2R to facilitatE earLy libEration From mechanicAl Ventilation inpatientS With Copd Acute Exacerbation. Https://Clinicaltrials.Gov/Show/NCT04147104. 2019.  1496. NCT. The "Hypotension Prediction Index" in Patients Undergoing Lung Surgery. Https://Clinicaltrials.Gov/Show/NCT04149314. 2019.  1497. NCT. Efficacy and Safety of Therapy With IgM-enriched Immunoglobulin With a Personalized Dose vs Standard Dose in Patients With Septic Shock. Https://Clinicaltrials.Gov/Show/NCT04182737. 2019.  1498. NCT. Conservative Versus Conventional Oxygen Administration in Critically Ill Patients. Https://Clinicaltrials.Gov/Show/NCT04198077. 2019.  1499. NCT. Trial for the Early Identification of Acute Kidney Injury. Https://Clinicaltrials.Gov/Show/NCT04200950. 2019.  1500. NCT. Early Versus Late Initiation of ECMO (Extracorporal Membrane Oxygenation) Trial (ELIEO-Trial). Https://Clinicaltrials.Gov/Show/NCT04208126. 2019.  1501. NCT. Single-Center Prospective Study to Investigate the Difference in the Incidence of Contrast-Induced Nephropathy in High-Risk Patients With the Use of the Dye-Vert Plus System. Https://Clinicaltrials.Gov/Show/NCT04279457. 2020.  1502. NCT. Establishment of a Personalized Pharmaceutical Plan in Renal or Hepatic Transplant Patients. Https://Clinicaltrials.Gov/Show/NCT04295928. 2020.  1503. NCT. Evaluation of Regional Anticoagulation With Citrate in Extended Hemodialysis. Https://Clinicaltrials.Gov/Show/NCT04297839. 2019.  1504. NCT. Effects of Intraoperative Fluid Therapy on Acute Kidney Injury After Thoracoscopic Lobectomy. Https://Clinicaltrials.Gov/Show/NCT04302467. 2020.  1505. NCT. Early Postoperative CRRT After Liver Transplantation in ACLF Patients With Overt HE. Https://Clinicaltrials.Gov/Show/NCT04317222. 2020.  1506. NCT. Hydroxychloroquine for the Treatment of Patients With Mild to Moderate COVID-19 to Prevent Progression to Severe Infection or Death. Https://Clinicaltrials.Gov/Show/NCT04323631. 2020.  1507. NCT. Hydrocortisone for COVID-19 and Severe Hypoxia. Https://Clinicaltrials.Gov/Show/NCT04348305. 2020.  1508. NCT. Efficacy and Safety of Individualized P2Y12 Receptor Antagonists Treatment Based on Agregometry Versus Fixed Dose Regimen in Patients After Acute Myocardial Infarction. Https://Clinicaltrials.Gov/Show/NCT04369534. 2020.  1509. NCT. Influence of the Autonomic Nervous System in Response to Exercise in Hypertensive Individuals. Https://Clinicaltrials.Gov/Show/NCT04371757. 2020.  1510. NCT. Dapagliflozin in Diabetic Patients (Type 2) With Decompensated Heart Failure. Https://Clinicaltrials.Gov/Show/NCT04385589. 2020.  1511. NCT. Early Use of Long-acting Tacrolimus in Lung Transplant Recipients. Https://Clinicaltrials.Gov/Ct2/Show/NCT04469842. 2020.  1512. NCT. Suloexide in the Treatment of Early Stages of COVID-19. Https://Clinicaltrials.Gov/Show/NCT04483830. 2020.  1513. NCT. Crystalloid FLUID Choices for Resuscitation of Hospitalized Patients. Https://Clinicaltrials.Gov/Show/NCT04512950. 2019.  1514. NCT. Determination of the Hemoadsorption Impact as Adjunctive Treatment Upon the Support Therapy of COVID-19. Https://Clinicaltrials.Gov/Show/NCT04518969. 2020.  1515. NCT. Valproic Acid (VPA) for Acute Kidney Injury (AKI) in Trauma Patients. Https://Clinicaltrials.Gov/Show/NCT04531579. 2020.  1516. NCT. Valproic Acid (VPA) for Acute Kidney Injury (AKI) in Liver Transplant Patients. Https://Clinicaltrials.Gov/Show/NCT04531592. 2020.  1517. NCT. Mild vs Moderate Hypothermic Circulatory Arrest With Unilateral Anterograde Cerebral Perfusion in Hemiarch Replacement. Https://Clinicaltrials.Gov/Show/NCT04569864. 2020.  1518. NCT. Inflammatory Signal Inhibitors for COVID-19 (MATIS). Https://Clinicaltrials.Gov/Show/NCT04581954. 2020.  1519. NCT. Early Mobilization in Ventilated sEpsis & Acute Respiratory Failure Study. Https://Clinicaltrials.Gov/Show/NCT04582760. 2020.  1520. NCT. Extracorporeal CO2 Removal for Acute Decompensation of COPD. Https://Clinicaltrials.Gov/Show/NCT04582799. 2020.  1521. NCT. Efficacy and Safety of a Highly Selective Semipermeable Membrane (AN-69 Oxiris) vs (Standard AN-69) in COVID-19. Https://Clinicaltrials.Gov/Show/NCT04597034. 2020.  1522. NCT. Multicentre Clinical Study to Evaluate the Effect of Personalized Therapy on Patients With Immunoglobulin A Nephropathy. Https://Clinicaltrials.Gov/Show/NCT04662723. 2020.  1523. NCT. Berinert (C1INH) vs Placebo for DGF/IRI. Https://Clinicaltrials.Gov/Show/NCT04696146. 2020.  1524. NCT. Albumin To Enhance Recovery After Acute Kidney Injury. Https://Clinicaltrials.Gov/Show/NCT04705896. 2021.  1525. NCT. DyeVert System and Contrast-induced Acute Kidney Injury. Https://Clinicaltrials.Gov/Ct2/Show/NCT04714736. 2021.  1526. NCT. Comparison of Effectiveness and Complications of Catheter Lock Solutions in Non-tunneled Hemodialysis Catheters. Https://Clinicaltrials.Gov/Show/NCT04772209. 2021.  1527. NCT. Intravenous Imatinib in Mechanically Ventilated COVID-19 Patients. Https://Clinicaltrials.Gov/Show/NCT04794088. 2021.  1528. NCT. Citrate Versus Heparin in Continuous Renal Replacement Therapy. Https://Clinicaltrials.Gov/Show/NCT04865510. 2021.  1529. NCT. ENvarsus for Impaired Glucose Tolerance Post REnal transplAnT. Https://Clinicaltrials.Gov/Show/NCT04973982. 2021.  1530. NCT. The ORTIZ Study: optimising RASi Therapy With SZC. Https://Clinicaltrials.Gov/Show/NCT04983979. 2021.  1531. NCT. To Evaluate the Safety and Efficacy of Preemptive Administration of Continuous Renal Replacement Therapy in Patients With Acute Liver Failure With Cerebral Edema. Https://Clinicaltrials.Gov/Show/NCT04991259. 2021.  1532. NCT. Safety and Efficacy of HA380 Hemoadsorption in Patients With Septic Shock. Https://Clinicaltrials.Gov/Show/NCT04997421. 2021.  1533. NCT. Lokelma for RAAS Maximisation in CKD & Heart Failure. Https://Clinicaltrials.Gov/Show/NCT05004363. 2020.  1534. NCT. Multidiscipline Care for Acute Kidney Disease (AKD). Https://Clinicaltrials.Gov/Show/NCT05064904. 2021.  1535. NCT. The Danish Pre-HCQ COVID Dialysis Study. Https://Clinicaltrials.Gov/Show/NCT05110651. 2020.  1536. NCT. Effectiveness of Remote Ischemic Preconditioning for Prevention of Contrast Induced Acute Kidney Injury in Patients Undergoing Coronary Angiograms. Https://Clinicaltrials.Gov/Show/NCT05147831. 2021.  1537. NCT. HAT for the Treatment of Sepsis Associated With NASTI. Https://Clinicaltrials.Gov/Ct2/Show/NCT05157360. 2021.  1538. NCT. Positive End Espiratory Pressure Trial in Coronavirus Disease 19 Treated With Continuous Positive Airway Pressure. Https://Clinicaltrials.Gov/Show/NCT05178160. 2021.  1539. NCT. Hyperhydration in Children With Shiga Toxin-Producing E. Coli Infection. Https://Clinicaltrials.Gov/Show/NCT05219110. 2022.  1540. NCT. Study Design of the Diacerein in Patients With Covid-19. Https://Clinicaltrials.Gov/Show/NCT05226754. 2022.  1541. NCT. EXtended Use of FOsfomycin for the Treatment of CYstitis in Primary Care. Https://Clinicaltrials.Gov/Show/NCT05254808. 2022.  1542. NCT. Intravenous vs. Oral Hydration to Reduce the Risk of Post-Contrast Acute Kidney Injury After Intravenous Contrast-Enhanced Computed Tomography in Patients With Severe Chronic Kidney Disease. Https://Clinicaltrials.Gov/Show/NCT05283512. 2022.  1543. NCT. Effect of a FLUid Bolus or a Low Dose VAsopressor Infusion on Cardiovascular Collapse Among Critically Ill Adults Undergoing Tracheal Intubation. Https://Clinicaltrials.Gov/Show/NCT05318066. 2022.  1544. NCT. Non-invasive Goal-directed thErapy oN cIrcUlatory Shock. Https://Clinicaltrials.Gov/Show/NCT05336357. 2022.  1545. NCT. Clinical Efficacy of Ulinastatin for Treatment of Sepsis With Systemic Inflammatory Response Syndrome. Https://Clinicaltrials.Gov/Show/NCT05391789. 2021.  1546. NCT. Fast-track Blood Test for Suspected Fever by Deficiency of a Kind of White Blood Cells As Main Defense Against Infection. Https://Clinicaltrials.Gov/Show/NCT05393505. 2021.  1547. NCT. Proactive Prescription-based Fluid Management vs Usual Care in Critically Ill Patients on Kidney Replacement Therapy. Https://Clinicaltrials.Gov/Ct2/Show/NCT05473143. 2022.  1548. NCT. Human Umbilical Cord Mesenchymal Stem Cell(UC-MSC)Delayed Renal Chronic Kidney Disease(CKD3、4 ). Https://Clinicaltrials.Gov/Show/NCT05512988. 2022.  1549. NCT. Graft Acute Kidney Injury: vitamin B3 to Facilitate Renal Recovery In the Early Life of a Transplant. Https://Clinicaltrials.Gov/Show/NCT05513807. 2022.  1550. NCT. Randomized Controlled Trial on 3D Printed Assistive Device for Continuous Ambulatory Peritoneal Dialysis. Https://Clinicaltrials.Gov/Show/NCT05521425. 2022.  1551. NCT. Revascularization Strategy of Multivessel Disease for Patients With Acute Myocardial Infarction Complicated by Cardiogenic Shock Undergoing Veno-arterial Extracorporeal Membrane Oxygenator. Https://Clinicaltrials.Gov/Show/NCT05527717. 2022.  1552. NCT. Colchicine and Inflammation in Hemodialysis Patients. Https://Clinicaltrials.Gov/Show/NCT05677555. 2022.  1553. NCT. Combination of Cytokine Hemosorption and High-volume Hemofiltration in Acute Pancreatitis. Https://Clinicaltrials.Gov/Ct2/Show/NCT05694988. 2023.  1554. NCT. Semaglutide Treatment for Hyperglycaemia After Renal Transplantation. Https://Clinicaltrials.Gov/Ct2/Show/NCT05702931. 2022.  1555. NCT. CKD Specific Telemonitoring Platform to Minimize Adverse Outcomes in High Risk CKD Patients. Https://Clinicaltrials.Gov/Ct2/Show/NCT05726526. 2021.  1556. NCT. Patients Undergoing Continuous Venovenous Hemodiafiltration: effects of Increased Blood Flow. Https://Clinicaltrials.Gov/Ct2/Show/NCT05796661. 2023.  1557. NCT. Efficacy and Safety of corticoSTEROids Added to Standard Therapy in Patients With Acute Heart Failure (STERO-AHF). Https://Clinicaltrials.Gov/Show/NCT05809011. 2023.  1558. NCT. Early Deresuscitation Strategy Driven by Tissue Perfusion in Renal Replacement Therapy in Patients With Acute Renal Failure. Https://Clinicaltrials.Gov/Ct2/Show/NCT05817539. 2023.  1559. NCT. Biomechanical Properties of the Human Ascending Aortic Wall in Aneurysm. Https://Clinicaltrials.Gov/Show/NCT05839990. 2023.  1560. NCT. ADVOS® Versus CVVHD in Metabolic or Mixed Acidosis. Https://Clinicaltrials.Gov/Show/NCT05842369. 2023.  1561. NCT. A Comparative Study of Two Kinds of Hemodialysis Filters. Https://Clinicaltrials.Gov/Show/NCT05899283. 2023.  1562. NCT. Comparison of Plasma Neutrophil Gelatinase-Associated Lipocalin (pNGAL) Level in De-resuscitation With Furosemide Group and Control Group. Https://Clinicaltrials.Gov/Ct2/Show/NCT05939245. 2023.  1563. NCT. The Effect of Sujok Therapy on Symptom Burden and Comfort Level in Patients Undergoing Hemodialysis Treatment. Https://Clinicaltrials.Gov/Ct2/Show/NCT05939505. 2023.  1564. NCT. Acupressure and Fistula Needle Insert Pain Management. Https://Clinicaltrials.Gov/Ct2/Show/NCT05971134. 2023.  1565. NCT. Clinical Analysis of Vitamin B6 in Sepsis. Https://Clinicaltrials.Gov/Ct2/Show/NCT06008223. 2023.  1566. NCT. IHD Versus CRRT for Severe Acute Kidney Injury in Critically Ill Patients. Https://Clinicaltrials.Gov/Ct2/Show/NCT06032884. 2023.  1567. NCT. The Effect of Reiki Application on Pain Severity and Quality of Life in Patients Receiving Hemodialysis Treatment. Https://Clinicaltrials.Gov/Ct2/Show/NCT06063616. 2023.  1568. NCT. Hemodynamic Effects of Variations in Net Ultrafiltration Rate During Continuous Renal Replacement Therapy. Https://Clinicaltrials.Gov/Ct2/Show/NCT06071026. 2023.  1569. NCT. Effect of Vasopressin on Kidney and Cardiac Function in Septic Shock. Https://Clinicaltrials.Gov/Ct2/Show/NCT06125184. 2022.  1570. NCT. Pilot Study on HA380 Column Use in Critically Ill Patients Receiving Extracorporeal Support. Https://Clinicaltrials.Gov/Ct2/Show/NCT06179771. 2023.  1571. NCT. Urinary Parameters to Predict Weaning of Renal Replacement Therapy in the Critically Ill. Https://Clinicaltrials.Gov/Ct2/Show/NCT06214390. 2023.  1572. NCT. Protocol Based-furosemide Stress Test Versus Standard Care to Evaluate Renal Recovery During Continuous Renal Replacement Therapy. Https://Clinicaltrials.Gov/Ct2/Show/NCT06229990. 2024.  1573. NCT. Multi-hospital Electronic Decision Support for Drug-associated Acute Kidney Injury. Https://Clinicaltrials.Gov/Ct2/Show/NCT06264752. 2024.  1574. NCT. Efficacy of the Use of Vasopressin as a Primary Vasoconstrictor in Critically Ill Patients. Https://Clinicaltrials.Gov/Ct2/Show/NCT06265259. 2024.  1575. Neely J, Giraud K, Vuylsteke A. Optimal time to commence continuous veno-venous haemofiltration (CVVH) in cardiac surgery patients: A pilot study. J Cardiothor Vasc an. 2010;24(3):S44. http://doi.org/10.1053/j.jvca.2010.04.015  1576. Negash DT, Dhingra VK, Copland M, Griesdale D, Henderson W. Intensity of continuous renal replacement therapy in acute kidney injury in the intensive care unit: A systematic review and meta-analysis. Vasc Endovasc Surg. 2011;45(6):504-10. http://doi.org/10.1177/1538574411407935  1577. Neijmann ^T, Kural A, Sever N, Doğan H, Sarıkaya S. Evaluation of renal function in rats with moderate and mild brain trauma. Ulusal Travma Ve Acil Cerrahi Dergisi. 2022;28(1):1-7. http://doi.org/10.14744/tjtes.2020.29015  1578. Nemeth E, Soltesz A, Kovacs E, Szakal-Toth Z, Tamaska E, Katona H, et al. Use of intraoperative haemoadsorption in patients undergoing heart transplantation: a proof-of-concept randomized trial. Esc Heart Fail. 2024;11(2):772-82. http://doi.org/10.1002/ehf2.14632  1579. Neumayer HH, Kunzendorf U, Schreiber M. Protective effects of calcium antagonists in human renal transplantation. Kidney Int Suppl. 1992;36:S87-93.  1580. Neumayer HH, Kunzendorf U, Schreiber M. Protective effects of diltiazem and the prostazycline analogue iloprost in human renal transplantation. Renal Failure. 1992;14(3):289-96. http://doi.org/10.3109/08860229209106631  1581. Neumayer HH, Schreiber M, Wagner K. Prevention of delayed graft function in cadaveric kidney transplants by the calcium antagonist diltiazem and the prostacyclin-analogue iloprost--outcome of a prospective randomized clinical trial. Prog Clin Biol Res. 1989;301:289-95.  1582. Neumayer HH, Wagner K. Prevention of delayed graft function in cadaver kidney transplants by diltiazem: Outcome of two prospective, randomized clinical trials. J Cardiovasc Pharm. 1987;10(SUPPL. 10):S170-7.  1583. Ng CF, Luke S, Yee CH, Chu WCW, Wong KT, Yuen JWM. A prospective randomized study comparing the effect of different kidney protection treatment protocols on acute renal injury after extracorporeal shockwave lithotripsy. J Endourol. 2017;31(1):57-65. http://doi.org/10.1089/end.2016.0653  1584. Ngugi NN, McLigeyo SO, Kayima JK. Treatment of hyperkalaemia by altering the transcellular gradient in patients with renal failure: effect of various therapeutic approaches. East Afr Med J. 1997;74(8):503-9.  1585. T NH, HMP B, K B, JL S, C C, O B, et al. Mechanical assist devices for acute cardiogenic shock. Cochrane Db Syst Rev. 2020(6). http://doi.org/10.1002/14651858.CD013002.pub2  1586. B N, WJ C, YF L, Z C, Q Y, YX Z, et al. A prospective, double-blind, randomized, controlled trial on the efficacy and cardiorenal safety of iodixanol vs. iopromide in patients with chronic kidney disease undergoing coronary angiography with or without percutaneous coronary intervention. Catheter Cardio Inte. 2008(7). http://doi.org/10.1002/ccd.21713  1587. Nigwekar SU, Strippoli GFM, Navaneethan SD. Thyroid hormones for acute kidney injury. Cochrane Db Syst Rev. 2013;2013(1). http://doi.org/10.1002/14651858.CD006740.pub2  1588. M N, Y K, Y N. Effect of tolvaptan on renal function in the early postoperative period after cardiac surgery. Innovations: Technology and Techniques in Cardiothoracic and Vascular Surgery. 2017(Supplement 3). http://doi.org/10.1097/IMI.0000000000000415  1589. M N, C S, M M, Y O, T T, N S, et al. Oral nicorandil for prevention of cardiac death in hemodialysis patients without obstructive coronary artery disease: a propensity-matched patient analysis. Nephron. Clinical Practice. 2011(4). http://doi.org/10.1159/000329112  1590. T N, V L, D M, J N, E N, A B, et al. POS-806 LOW DOSE COLCHICINE PROPHYLAXIS FOR SYMPTOMATIC COVID-19 PREVENTION IN PATIENTS ON KIDNEY REPLACEMENT THERAPY: OUTCOMES OF AN OBSERVATIONAL COHORT STUDY. Kidney Int Rep. 2021(4). http://doi.org/10.1016/j.ekir.2021.03.839  1591. AR N, NW L, RH P. Effect of renal failure and hemodialysis on cephacetrile pharmacokinetics. Clinical Pharmacology and Therapeutics. 1972(6). http://doi.org/10.1002/cpt1972136887  1592. NL. Management of angiotensin inhibitors during the perioperative period. Https://Trialsearch.Who.Int/Trial2.Aspx?TrialID=NL8763. 2019.  1593. Nnodum B, Sundar P, Hill C, Lamontagne S. TORSEMIDE-INDUCED ALLERGIC INTERSTITIAL NEPHRITIS NECESSITATING HEMODIALYSIS. Chest. 2019;156(4):A1947. http://doi.org/10.1016/j.chest.2019.08.1933  1594. AC N, HH H, D S, SB S, RJ C, D S, et al. Detrimental effect of cyclosporine on initial function of cadaver renal allografts following extended preservation. Results of a randomized prospective study. Transplantation. 1986(2). http://doi.org/10.1097/00007890-198608000-00010  1595. PA N, JM G, FJ R, P E, A F, G A, et al. De novo use of everolimus with elimination or minimization of cyclosporine in renal transplant recipients. Transpl P. 2011(9). http://doi.org/10.1016/j.transproceed.2011.10.032  1596. NTR. IMPRESS in STEMI. Https://Trialsearch.Who.Int/Trial2.Aspx?TrialID=NTR1079. 2007.  1597. NTR. Coronary angiography after cardiac arrest. Https://Trialsearch.Who.Int/Trial2.Aspx?TrialID=NTR4973. 2014.  1598. Nugent K, Berdine G, Pena C. Does Fluid Administration Based on Fluid Responsiveness Tests such as Passive Leg Raising Improve Outcomes in Sepsis? Curr Cardiol Rev. 2022;18(5):18-23. http://doi.org/10.2174/1573403X18666220304202556  1599. Oakland H, Andrews J. Cavitary rhizopus infection. J Gen Intern Med. 2016;31(2):S578.  1600. Z O, A C, L C, S F, M G, C M, et al. Higher versus Lower Continuous Renal Replacement Therapy Intensity in Critically ill Patients with Liver Dysfunction. Blood Purificat. 2018(1‐3). http://doi.org/10.1159/000480224  1601. O'Brien Z, Finnis M, Gallagher M, Bellomo R. Early Treatment with Human Albumin Solution in Continuous Renal Replacement Patients. Blood Purificat. 2021;50(2):205-13. http://doi.org/10.1159/000509890  1602. Occhipinti G, Laudani C, Spagnolo M, Greco A, Capodanno D. Diuresis-matched versus standard hydration in patients undergoing percutaneous cardiovascular procedures: meta-analysis of randomized clinical trials. Rev Esp Cardiol. 2023;76(10):759-66. http://doi.org/10.1016/j.recesp.2023.02.004  1603. Oda T. Acute kidney injury after off-pump coronary artery bypass grafting. Circ J. 2010;74(6):1069-70. http://doi.org/10.1253/circj.CJ-10-0329  1604. G O, B T, B H, M K, M B, P C, et al. Efficacy and safety of basiliximab in pediatric renal transplant patients receiving cyclosporine, mycophenolate mofetil, and steroids. Transplantation. 2008(9). http://doi.org/10.1097/TP.0b013e318188af15  1605. CK O, KH H, J H, YH K, YL K, YS K. Safety and efficacy of the early introduction of everolimus with reduced-exposure cyclosporine a in de novo kidney recipients. Transplantation. 2015(1). http://doi.org/10.1097/TP.0000000000000225  1606. HJ O, JN A, S O, H R, JP L, DK K, et al. VolumE maNagement Under body composition monitoring in critically ill patientS on CRRT: study protocol for a randomized controlled trial (VENUS trial). Trials. 2018(1). http://doi.org/10.1186/s13063-018-3056-y  1607. O'Hara JF, Mascha EJ, Bonilla A. Fenoldopam renal effects in solitary partial nephrectomy. Canadian Journal of Anesthesia. 2011;58:S16. http://doi.org/10.1007/s12630-011-9586-3  1608. S O, A L, H G, H W, G T, F R, et al. A randomized pilot study of cyclosporin G in renal transplantation. Transpl Int. 1992. http://doi.org/10.1007/978-3-642-77423-2_136  1609. Okoduwa A, Ahmed N, Guo Y, Scipione MR, Papadopoulos J, Eiras DP, et al. Nephrotoxicity associated with intravenous polymyxin b once- versus twice-daily dosing regimen. Antimicrob Agents Ch. 2018;62(8). http://doi.org/10.1128/AAC.00025-18  1610. JG O, F W, KR R, G G, PS K, SW B, et al. Gender-Specific Differences in Baseline, Peak, and Delta Serum Creatinine: the NACSELD Experience. Digest Dis Sci. 2017(3). http://doi.org/10.1007/s10620-016-4416-7  1611. Oliveras L, Codina S, Coloma A, Pérez-Garzón RM, Sbraga F, Boza E, et al. NEPHROLOGY INTERVENTION IN PATIENTS WAITING FOR CARDIAC SURGERY: A RANDOMIZED CLINICAL TRIAL. Nephrology. 2022;27:30. http://doi.org/10.1111/nep.14099  1612. AM O, D R, C M, L C, S K, OK G, et al. Comparison of intraoperative aminophylline versus furosemide in treatment of oliguria during pediatric cardiac surgery. Pediatr Crit Care Me. 2016(8). http://doi.org/10.1097/PCC.0000000000000834  1613. T O, H G, K M, H K, H H, M I. Immediate hemodialysis after percutaneous transvenous angioplasty increases patency rates of arterio-venous fistula. Clin Nephrol. 2004(4). http://doi.org/10.5414/cnp62301  1614. Onuigbo M. FOUR-YEAR ACCOUNT OF RENAL AND MORTALITY OUTCOMES AFTER ELECTIVE DISCONTINUATION OF LONG-TERM RAAS BLOCKADE IN CKD PATIENTS WITH OTHERWISE INEXPLICABLE PROGRESSIVE NEW-ONSET ACUTE KIDNEY INJURY. Am J Kidney Dis. 2023;81(4):S6-7. http://doi.org/10.1053/j.ajkd.2023.01.023  1615. Onuigbo M. WCN23-1002 Four-Year Renal and Mortality Outcomes After Stopping Long-Term RAAS Blockade in CKD Patients with Otherwise Inexplicable Progressive New-Onset Acute-Kidney-Injury: A Post STOP ACEi Trial Review. Kidney Int Rep. 2023;8(3):S37. http://doi.org/10.1016/j.ekir.2023.02.084  1616. Onuigbo MA. A Four-Year Report on Renal Outcomes Following the Elective Withdrawal of Long-Term Renin-Angiotensin-Aldosterone Blockade in a Cohort of Patients With Otherwise Inexplicable New-Onset and Progressive Acute Kidney Injury. Cureus J Med Science. 2022;14(10):e30794. http://doi.org/10.7759/cureus.30794  1617. K O, K P, A K, L V, I N, E K. The effect of heparin rinse on the biocompatibility of continuous veno-venous hemodiafiltration. Int J Artif Organs. 2002(6). http://doi.org/10.1177/039139880202500606  1618. JC O, Y W, N M, B A, L A, F A, et al. Causes and Characteristics of Death in Intensive Care Units: a Prospective Multicenter Study. Anesthesiology. 2017(5). http://doi.org/10.1097/ALN.0000000000001612  1619. D OC, C S, K D, L N, FS T. Colloids for fluid resuscitation: what is their role in patients with shock? Minerva Anestesiol. 2014(8).  1620. O'Reilly P, Tolwani A. Renal replacement therapy III: IHD, CRRT, SLED. Crit Care Clin. 2005;21(2):367-78. http://doi.org/10.1016/j.ccc.2005.01.004  1621. J O, F L, M F, N P, E G, E G, et al. Should Asymptomatic Bacteriuria Be Systematically Treated in Kidney Transplant Recipients? Results From a Randomized Controlled Trial. Am J Transplant. 2016(10). http://doi.org/10.1111/ajt.13829  1622. O O, M V, D A, M A, S M, R P. The predictive value of left ventricular hypertrophy in peritoneal dialysis patients: results of the study ADEMEX. Periton Dialysis Int. 2012.  1623. Orme RML, Perkins GD, McAuley DF, Liu KD, Mason AJ, Morelli A, et al. An efficacy and mechanism evaluation study of Levosimendan for the Prevention of Acute oRgan Dysfunction in Sepsis (LeoPARDS): Protocol for a randomized controlled trial. Trials. 2014;15(1). http://doi.org/10.1186/1745-6215-15-199  1624. Oskuei A, Amin SO, Connolly D, Geeti A, Kaufman D. Ability of the acute kidney injury to improve the prognostic performance of national early warning score. Intens Care Med. 2014;40(1):S116. http://doi.org/10.1007/s00134-013-3451-5  1625. AM Ø, AN J, S B, NP E, FH M, JN B. Effect of 0.9% NaCl compared to plasma-lyte on biomarkers of kidney injury, sodium excretion and tubular transport proteins in patients undergoing primary uncemented hip replacement - a randomized trial. Bmc Nephrol. 2021(1). http://doi.org/10.1186/s12882-021-02310-4  1626. Oudemans-van Straaten HM, van Schilfgaarde M, Molenaar PJ, Wester JP, Leyte A. Hemostasis during low molecular weight heparin anticoagulation for continuous venovenous hemofiltration: a randomized cross-over trial comparing two hemofiltration rates. Critical Care (London, England). 2009;13(6):R193.  1627. CAJ O, RC M, RA P, van den Boogaard WMC, DSJ K, van de Wetering J, et al. Fasting before living-kidney donation: effect on donor well-being and postoperative recovery: study protocol of a multicenter randomized controlled trial. Trials. 2022(1). http://doi.org/10.1186/s13063-021-05950-x  1628. M O, T P, S A, BA U, H Y, A I, et al. Addition of N-acetyl cysteine to carvedilol decreases the incidence of acute renal injury after cardiac surgery. Clin Cardiol. 2014(2). http://doi.org/10.1002/clc.22227  1629. PACTR. Early Prediction of Acute Kidney Injury post operative. Https://Trialsearch.Who.Int/Trial2.Aspx?TrialID=PACTR202002537852294. 2020.  1630. Paganini EP, Sandy D, Moreno L, Kozlowski L, Sakai K. The effect of sodium and ultrafiltration modelling on plasma volume changes and haemodynamic stability in intensive care patients receiving haemodialysis for acute renal failure: A prospective, stratified, randomized, cross-over study. Nephrol Dial Transpl. 1996;11(SUPPL. 8):32-7.  1631. Pais FM, Sinha P, Liu KD, Matthay MA. Influence of clinical factors and exclusion criteria on mortality in ards observational studies and randomized controlled trials. Resp Care. 2018;63(8):1060-9. http://doi.org/10.4187/respcare.06034  1632. S P, C M, JM C, GP Y, BS R, T L. Esterified starch as a treatment for acute gastroenteritis-a double blind randomized controlled trial. Gastroenterology. 2013(5).  1633. PM P, T O, JH Z, RA S, MW S. Design of the VA/NIH Acute Renal Failure Trial Network (ATN) Study: intensive versus conventional renal support in acute renal failure. Clinical Trials (London, England). 2005(5).  1634. PM P, JH Z, TZ O, GM C, ST C, D C, et al. Intensity of renal support in critically ill patients with acute kidney injury. New Engl J Med. 2008(1). http://doi.org/10.1056/NEJMoa0802639  1635. SC P, EYM C, DO M, F B, GFM S. Interventions for preventing bone disease in kidney transplant recipients. Cochrane Db Syst Rev. 2019(10). http://doi.org/10.1002/14651858.CD005015.pub4  1636. J PS, A PG, L MA, A P, L G, JC C, et al. Action of quinine sulphate on the incidence of muscle cramps during hemodialysis (author's transl). Med Clin-Barcelona. 1980(6).  1637. Panah F, Ghorbanihaghjo A, Argani H, Haiaty S, Rashtchizadeh N, Hosseini L, et al. The effect of oral melatonin on renal ischemia-reperfusion injury in transplant patients: A double-blind, randomized controlled trial. Transpl Immunol. 2019;57:101241. http://doi.org/10.1016/j.trim.2019.101241  1638. YY P, M D, A H, J C, C T, S M. Pharmacokinetics, safety and tolerability of serelaxin in patients with severe renal impairment or end-stage renal disease. Eur J Heart Fail. 2015. http://doi.org/10.1002/ejhf.277  1639. DVS P, C A, O M, ML U. Prediction of Mortality or the Need for Continued Renal Replacement Therapy following Acute Kidney Injury Requiring. Journal of the American Society of Nephrology : JASN. 2016.  1640. Pankratz VS, Argyropoulos C, Abdel-Kader K, Liang KV, Palevsky PM, Unruh ML. SOFA Scores as Predictors of Mortality and Dialysis Dependency in Acute Kidney Injury. J Am Soc Nephrol. 2015;26:470A.  1641. Pannu R, McKusick MA, Oderich G, Misra S. Five year outcomes of drug eluting stents in renal artery stenosis. J Vasc Interv Radiol. 2011;22(3):S73. http://doi.org/10.1016/j.jvir.2011.01.187  1642. Panwar R, Van Haren F, Cazzola F, Nourse M, Brinkerhoff G, Quail A. Standard care versus individualized blood pressure targets among critically ill patients with shock: A multicenter feasibility and preliminary efficacy study. J Crit Care. 2022;70. http://doi.org/10.1016/j.jcrc.2022.154052  1643. Z P, PW G, JS F. Effects of Acute Exercise on Cardiac Autonomic Response and Recovery in Non-Dialysis Chronic Kidney Disease Patients. Res Q Exercise Sport. 2023(3). http://doi.org/10.1080/02701367.2022.2057401  1644. L P, B T, R E, L DS, E-D M, J N, et al. Rationale, design and baseline characteristics of pediatric renal transplant recipients in cradle study: a randomised study to evaluate the effect of early everolimus initiation to reduce calcineurin inhibitor exposure and to withdraw steroid. Pediatric Nephrology (Berlin, Germany). 2015(9). http://doi.org/10.1007/s00467-015-3158-7  1645. Paplaczyk K, Rhodes N, Weslander E, Kaiho T, Valadez A, Kurihara C, et al. Perioperative Cefepime Pharmacokinetics Using Therapeutic Drug Monitoring. J Heart Lung Transpl. 2024;43(4):S645-6. http://doi.org/10.1016/j.healun.2024.02.1024  1646. Parienti JJ, Mégarbane B, Fischer MO, Lautrette A, Gazui N, Marin N, et al. Catheter dysfunction and dialysis performance according to vascular access among 736 critically ill adults requiring renal replacement therapy: A randomized controlled study. Crit Care Med. 2010;38(4):1118-25. http://doi.org/10.1097/CCM.0b013e3181d454b3  1647. SI P, CR F, PG M, R G, A S, R S, et al. Pharmacokinetic/pharmacodynamic relationships of FTY720 in kidney transplant recipients. Brazilian Journal of Medical and Biological Research = Revista Brasileira De Pesquisas Medicas E Biologicas. 2005(5). http://doi.org/10.1590/S0100-879X2005000500005  1648. Park JH, Jang HR, Huh W, Kim DJ, Kim YG, Oh HY, et al. A predictive model for successful conversion of continuous renal replacement therapy to intermittent hemodialysis for acute kidney injury in critical ill patients. Nephrol Dial Transpl. 2016;31:i542-3. http://doi.org/10.1093/ndt/gfw198.23  1649. Park MH, Shim HS, Kim WH, Kim HJ, Kim DJ, Lee SH, et al. Clinical risk scoring models for prediction of acute kidney injury after living donor liver transplantation: A retrospective observational study. Plos One. 2015;10(8). http://doi.org/10.1371/journal.pone.0136230  1650. Park S, Kim DK, Joo KW, Kim YS, Lee H. Prediction Model for Postoperative Acute Kidney Injury after Noncardiac Major Surgery Using Machine Learning. J Am Soc Nephrol. 2016;27:248A.  1651. K P, P H, P H, L C, M M, R C, et al. Chronic anticoagulation is not associated with a reduced risk of acute kidney injury in hospitalised Covid-19 patients. Bmc Nephrol. 2021(1). http://doi.org/10.1186/s12882-021-02436-5  1652. Parker RA, Himmelfarb J, Tolkoff-Rubin N, Chandran P, Wingard RL, Hakim RM. Prognosis of patients with acute renal failure requiring dialysis: results of a multicenter study. Am J Kidney Dis. 1998;32(3):432-43. http://doi.org/10.1053/ajkd.1998.v32.pm9740160  1653. A P, De Simone P, J P, E S, J P, H I, et al. Protein kinase C inhibitor sotrastaurin in de novo liver transplant recipients: a randomized phase II trial. Am J Transplant. 2015(5). http://doi.org/10.1111/ajt.13175  1654. MRJ P, A M, A S. Outcome of carotid artery stenting in patient with combined carotid and coronary artery disease. J Am Coll Cardiol. 2018(16).  1655. J P, D DC, M C, L P, JM G, AM F, et al. Interaction between everolimus and tacrolimus in renal transplant recipients: a pharmacokinetic controlled trial. Transplantation. 2010(8). http://doi.org/10.1097/TP.0b013e3181ccd7f2  1656. J P, C Q, J Z, D H. Steroid withdrawal in renal transplant patients on triple therapy with a calcineurin inhibitor and mycophenolate mofetil: a meta-analysis of randomized, controlled trials. Transplantation. 2004(10). http://doi.org/10.1097/01.TP.0000140969.43761.1F  1657. NN P, T T, C J, H L, P R, SJ G, et al. Developing novel renoprotective agents for the prevention of post cardiac surgery acute kidney injury. Journal of the Intensive Care Society. 2011(1).  1658. Patel B, Carson P, Shah M, Garg L, Agarwal M, Agrawal S, et al. Acute kidney injury requiring dialysis and in-hospital mortality in patients with chronic kidney disease and non–ST-segment elevation acute coronary syndrome undergoing early vs delayed percutaneous coronary intervention: A nationwide analysis. Clin Cardiol. 2017;40(12):1303-8. http://doi.org/10.1002/clc.22828  1659. Patel M, Thimons DG, Winston JL, Langholff W, McGowan T. An Open-Label, Randomized, Multicenter, Controlled Study of Epoetin Alfa for the Treatment of Anemia of Chronic Kidney Disease in the Long Term Care Setting. J Am Med Dir Assoc. 2012;13(3):244-8. http://doi.org/10.1016/j.jamda.2010.09.009  1660. Patidar KR, Peng JL, Pike F, Orman ES, Glick M, Kettler CD, et al. Associations Between Mean Arterial Pressure and Poor ICU Outcomes in Critically Ill Patients With Cirrhosis: Is 65 The Sweet Spot? Crit Care Med. 2020;48(9):E753-60. http://doi.org/10.1097/CCM.0000000000004442  1661. LC P. Chronic allograft nephropathy: an update. Kidney Int. 1999(3). http://doi.org/10.1046/j.1523-1755.1999.00611.x  1662. Pavilonis M, De Waele J, Decruyenaere J, Adukauskiene D, Hoste E. Adverse effects of volume therapy with hes solutions on kidney function: Meta-analysis of prospective, randomized controlled trials. Intens Care Med. 2009;35:S41.  1663. W P, Z R, H R, P L, A Y, P L, et al. Sodium zirconium cyclosilicate (ZS-9) for severe hyperkalemia: a post-HOC analysis of the phase 3 harmonize trial. Acad Emerg Med. 2015(5). http://doi.org/10.1111/acem.12644  1664. A P, I I, I V, J K, S I, J I, et al. Biomarkers of acute kidney injury in pediatric cardiac surgery. Clin Biochem. 2013(13‐14). http://doi.org/10.1016/j.clinbiochem.2013.07.008  1665. Peddi V, Ratner L, Cooper M, Gaber O, Feng S, Tso P, et al. Treatment with QPI-1002, a short interfering (SI) RNA for the prophylaxis of delayed graft function. Transplantation. 2014;98:153. http://doi.org/10.1097/01.tp.0000452122.74597.08  1666. KR P, HB R, JV P, MR S, EJ E, VE H. Failure of remote ischemic preconditioning to reduce the risk of postoperative acute kidney injury in children undergoing operation for complex congenital heart disease: a randomized single-center study. J Thorac Cardiov Sur. 2012(3). http://doi.org/10.1016/j.jtcvs.2011.08.044  1667. Peerapornratana S, Priyanka P, Wang S, Smith A, Singbartl K, Palevsky PM, et al. Sepsis-Associated Acute Kidney Disease. Kidney Int Rep. 2020;5(6):839-50. http://doi.org/10.1016/j.ekir.2020.03.005  1668. Peerapornratana S, Srisawat N. The Role of Urine NGAL for Early Detection of Colistin-Induced AKI: A Randomized Controlled Trial. J Am Soc Nephrol. 2016;27:242A.  1669. Penagaluru N, Makey D, Patel U, Mehandru S, D'Agati V. Recurrent acute tubular necrosis - A rare presentation of hypercalcemia. Am J Kidney Dis. 2010;55(4):A89. http://doi.org/10.1053/j.ajkd.2010.02.237  1670. L P, A W, CH W, AW C, DA S, C G. Antibody induction versus placebo, no induction, or another type of antibody induction for liver transplant recipients. Cochrane Db Syst Rev. 2014(6). http://doi.org/10.1002/14651858.CD010253.pub2  1671. Penny-Dimri JC, Cochrane AD, Perry LA, Smith JA. Characterising the Role of Perioperative Erythropoietin for Preventing Acute Kidney Injury after Cardiac Surgery: Systematic Review and Meta-Analysis. Heart Lung Circ. 2016;25(11):1067-76. http://doi.org/10.1016/j.hlc.2016.04.016  1672. PER-. A PHASE 3, MULTI-CENTER, RANDOMIZED, DOUBLE-BLIND, DOUBLE-DUMMY STUDY TO EVALUATE THE EFFICACY, SAFETY, AND TOLERABILITY OF CARBAVANCE (MEROPENEM/RPX7009) COMPARED TO PIPERACILLIN/TAZOBACTAM IN THE TREATMENT OF COMPLICATED URINARY TRACT INFECTIONS, INCLUDING ACUTE PYELONEPHRITIS, IN ADULTS. Https://Trialsearch.Who.Int/Trial2.Aspx?TrialID=PER-002-15. 2015.  1673. Perez PJ, Emerson S, Van Klei WA, Famure S, Li Y, Kim SJ, et al. Delayed Graft Function after Kidney Transplantation: Quantifying the role of Intraoperative Hypotension (The DeGift Study). Canadian Journal of Anesthesia. 2022;69:S242-3. http://doi.org/10.1007/s12630-022-02316-y  1674. Perez-Fernandez XLL, Sileanu FE, Riera JS, Liu KD, Kellum JA. Mortality Factors in Septic Shock Patients Requiring Continuous Renal Replacement Therapy and Timing Effect Based in Urine Output. J Am Soc Nephrol. 2015;26:114A.  1675. Perez-Valdivieso JR, Bes-Rastrollo M, Monedero P, de Irala J, Lavilla FJ. Prognosis and serum creatinine levels in acute renal failure at the time of nephrology consultation: an observational cohort study. Bmc Nephrol. 2007;8:14. http://doi.org/10.1186/1471-2369-8-14  1676. PE P, D B, P C, M M, W L, A G, et al. Ferric Citrate Dosing in Iron Deficiency Anemia in Nondialysis-Dependent Chronic Kidney Disease. Am J Nephrol. 2021(7). http://doi.org/10.1159/000516012  1677. Perkovic V, Jardine MJ, Neal B, Bompoint S, Heerspink HJL, Charytan DM, et al. Canagliflozin and renal outcomes in type 2 diabetes and nephropathy. New Engl J Med. 2019;380(24):2295-306. http://doi.org/10.1056/NEJMoa1811744  1678. Perner A, Haase N, Wetterslev J, Aneman A, Tenhunen J, Guttormsen AB, et al. Comparing the effect of hydroxyethyl starch 130/0.4 with balanced crystalloid solution on mortality and kidney failure in patients with severe sepsis (6S--Scandinavian Starch for Severe Sepsis/Septic Shock trial): study protocol, design and rationale for a double-blinded, randomised clinical trial. Trials. 2011;12:24. http://doi.org/10.1186/1745-6215-12-24  1679. Perry M, Ali N, Mount K, Ryder L, Murphy C, Phillips G, et al. Association between intravenous fluid chloride content and acute kidney injury in adults with sepsis. Crit Care Med. 2015;43(12):258-9. http://doi.org/10.1097/01.ccm.0000474858.98721.1e  1680. MD P, D C, SM G, S I, J R, D C, et al. Mycophenolate mofetil for the treatment of a first acute renal allograft rejection: the Mycophenolate Mofetil Acute Renal Rejection Study Group. Transplantation. 1998(2).  1681. Peters E, Mehta RL, Murray PT, Hummel J, Joannidis M, Kellum JA, et al. Study protocol for a multicentre randomised controlled trial: Safety, Tolerability, efficacy and quality of life Of a human recombinant alkaline Phosphatase in patients with sepsis-associated Acute Kidney Injury (STOP-AKI). Bmj Open. 2016;6(9). http://doi.org/10.1136/BMJOPEN-2016-012371  1682. MW P, TS M, M H, MN K, A G, CJS H, et al. Low-dose hydrocortisone in patients with COVID-19 and severe hypoxia (COVID STEROID) trial-Protocol and statistical analysis plan. Acta Anaesth Scand. 2020(9). http://doi.org/10.1111/aas.13673  1683. V P, E T. Intermittent hemodiafiltration in acute renal failure in critically ill patients. Clin Nephrol. 2001(4).  1684. Pettilä V, Merz T, Wilkman E, Perner A, Karlsson S, Lange T, et al. Targeted tissue perfusion versus macrocirculation-guided standard care in patients with septic shock (TARTARE-2S): study protocol and statistical analysis plan for a randomized controlled trial. Trials. 2016;17:384. http://doi.org/10.1186/s13063-016-1515-x  1685. Pham PTT, Pham PCT, Wilkinson AH. Management of renal dysfunction in the liver transplant recipient. Curr Opin Organ Tran. 2009;14(3):231-9. http://doi.org/10.1097/MOT.0b013e32832b34a4  1686. Philip F, Gornik HL, Rajeswaran J, Blackstone EH, Shishehbor MH. The impact of renal artery stenosis on outcomes after open-heart surgery. J Am Coll Cardiol. 2014;63(4):310-6. http://doi.org/10.1016/j.jacc.2013.09.046  1687. Pickkers P, Angus DC, Bass K, Bellomo R, van den Berg E, Bernholz J, et al. Phase-3 trial of recombinant human alkaline phosphatase for patients with sepsis-associated acute kidney injury (REVIVAL). Intens Care Med. 2024;50(1):68-78. http://doi.org/10.1007/s00134-023-07271-w  1688. Pickkers P, Schouten J, Laterre PF, Vincent JL, Groeneveld J, Jorens P, et al. Alkaline phosphatase improves kidney function in ICU patients with sepsis-induced acute kidney injury. Intens Care Med. 2010;36:S88. http://doi.org/10.1007/s00134-010-1999-x  1689. Pierce AC, Smith J, Bowman L, Wills A. Nesiritide does not prevent post surgical acute kidney injury in a cardiac transplant population. Am J Transplant. 2010;10:305. http://doi.org/10.1111/j.1600-6143.2010.03108.x  1690. Pieri M, Agracheva N, Bonaveglio E, Greco T, De Bonis M, Covello RD, et al. Bivalirudin versus heparin as an anticoagulant during extracorporeal membrane oxygenation: a case-control study. J Cardiothor Vasc an. 2013;27(1):30-4. http://doi.org/10.1053/j.jvca.2012.07.019  1691. Pike F, Yealy DM, Kellum JA, Huang DT, Barnato AE, Eaton TL, et al. Protocolized Care for Early Septic Shock (ProCESS) statistical analysis plan. Crit Care Resusc. 2013;15(4):301-10.  1692. Pilar Ovalle B, Andrea Vogel S, Guiliana Córdova L, Jaime Cerda L, Felipe Cavagnaro SM. Renal replacement therapy after cardiac surgery with extracorporeal circulation. Revista Chilena De Pediatria. 2012;83(1):24-32. http://doi.org/10.4067/S0370-41062012000100003  1693. AM P, J KH, NS K, MJ R, JV C, DP O, et al. Clinical impact of baseline chronic kidney disease in patients undergoing transcatheter or surgical aortic valve replacement. Catheter Cardio Inte. 2019(4). http://doi.org/10.1002/ccd.27928  1694. Pisano A, Monti G, Landoni G. Levosimendan: New indications and evidence for reduction in perioperative mortality? Current Opinion in Anaesthesiology. 2016;29(4):454-61. http://doi.org/10.1097/ACO.0000000000000357  1695. KP P, AR M, S J, G B, WO B, H L, et al. Toxicity versus rejection--or why conversions between cyclosporine A and FK506 were performed after liver transplantation. Clin Transplant. 1995(3 Pt 1).  1696. Platz KP, Mueller AR, Blumhardt G, Bachmann S, Bechstein WO, Kahl A, et al. Nephrotoxicity after orthotopic liver transplantation in cyclosporin A and FK 506-treated patients. Transplant International : Official Journal of the European Society for Organ Transplantation. 1994;7 Suppl 1:S52-7.  1697. Platz KP, Mueller AR, Blumhardt G, Bachmann S, Bechstein WO, Kahl A, et al. Nephrotoxicity following orthotopic liver transplantation: A comparison between cyclosporine and FK506. Transplantation. 1994;58(2):170-8.  1698. J P, C E, C F, J K, J P, B G. An amino acid-based peritoneal dialysis fluid buffered with bicarbonate versus glucose/bicarbonate and glucose/lactate solutions: an intraindividual randomized study. Periton Dialysis Int. 1999(5).  1699. Poddar B. Protocol-based care for early septic shock. Natl Med J India. 2014;27(5):267-8.  1700. MC P, WD S, AS P, C N, AJ P, CL E, et al. Feasibility of physical and occupational therapy beginning from initiation of mechanical ventilation. Crit Care Med. 2010(11). http://doi.org/10.1097/CCM.0b013e3181f270c3  1701. R P, R K, MF M, M M, S V, Van Buren C, et al. A trial of the prostaglandin E1 analogue, enisoprost, to reverse chronic cyclosporine-associated renal dysfunction. Am J Kidney Dis. 1992(4). http://doi.org/10.1016/s0272-6386(12)70295-0  1702. Ponce D, Balbi AL. Different prescribed doses of high volume peritoneal dialysis and outcome of patients with acute kidney injury. Periton Dialysis Int. 2012;32:S4.  1703. Ponce D, Berbel MN, Abrão JMG, Goes CR, Balbi AL. A randomized clinical trial of high volume peritoneal dialysis versus extended daily hemodialysis for acute kidney injury patients. Int Urol Nephrol. 2013;45(3):869-78. http://doi.org/10.1007/s11255-012-0301-2  1704. C P, M C, G T, S S, G S, P R, et al. A randomized trial of everolimus and low-dose cyclosporine in renal transplantation: with or without steroids? Transpl P. 2014(10). http://doi.org/10.1016/j.transproceed.2014.05.087  1705. C P, A T, GP S, V C, G R, P A, et al. A randomized study comparing three cyclosporine-based regimens in cadaveric renal transplantation. Italian Multicentre Study Group for Renal Transplantation (SIMTRe). Journal of the American Society of Nephrology : JASN. 1997(4). http://doi.org/10.1681/ASN.V84638  1706. Ponticelli C, Minetti L, Di Palo FQ, Vegeto A, Belli L, Corbetta G, et al. The Milan clinical trial with cyclosporine in cadaveric renal transplantation. A three-year follow-up. Transplantation. 1988;45(5):908-13. http://doi.org/10.1097/00007890-198805000-00013  1707. Ponticelli C, Tarantino A, Montagnino G, Aroldi A, Banfi G, De Vecchi A, et al. A randomized trial comparing triple-drug and double-drug therapy in renal transplantation. Transplantation. 1988;45(5):913-8. http://doi.org/10.1097/00007890-198805000-00014  1708. Powers HR, Hellinger WC, Cortese C, Elrefaei M, Khouzam S, Spiegel M, et al. Histologic acute graft pyelonephritis after kidney transplantatio
[truncated: 184,422 more chars]
