# Supplementary material for: Selection of timing of continuous renal replacement therapy in patients with acute kidney injury: A meta-analysis of randomized controlled trials
Source: PLoS One. 2025 Mar 25;20(3):e0320351. doi: 10.1371/journal.pone.0320351 (PMC11936205; doi:10.1371/journal.pone.0320351)
Supplement: S3 Table — (DOCX) [file pone.0320351.s011.docx]

**S2 Table. Risk of bias for each study.**

| **Study** | **D1** | **D2** | **D3** | **D4** | **D5** | **D6** | **D7** |
| --- | --- | --- | --- | --- | --- | --- | --- |
| **An 2021** | Low | Unclear | Unclear | Unclear | Low | Low | Low |
| **Bouman 2002** | Low | Low | Unclear | Low | Low | Low | Unclear |
| **Combes 2015** | Low | Low | Low | Low | Low | Low | Low |
| **Geri 2019** | Low | High | Low | Low | Low | Low | Low |
| **Lumlertgul 2018** | Low | Low | Low | Low | Low | Low | Low |
| **Payen 2009** | Low | Unclear | Unclear | Unclear | Low | Low | Low |
| **Srisawat 2018** | Low | Low | Low | Low | Low | Low | Low |
| **Sugahara 2004** | Unclear | Unclear | Unclear | Low | High | Low | Unclear |
| **Xia 2019** | Unclear | Unclear | Unclear | Low | Low | Low | Low |
| **Yang 2019** | Unclear | Unclear | Unclear | Low | Low | High | Unclear |
| **Yin 2018** | Unclear | Unclear | Low | Low | Low | Low | Unclear |
| **Zarbock 2016** | Low | Low | Low | Low | Low | Low | Low |

D1: Random sequence generation

D2: Allocation concealment

D3: Blinding of participants and personnel

D4: Blinding of outcome assessment

D5: Incomplete outcome data

D6: Selective reporting

D7: Other bias
