# Supplementary material for: Selection of timing of continuous renal replacement therapy in patients with acute kidney injury: A meta-analysis of randomized controlled trials
Source: PLoS One. 2025 Mar 25;20(3):e0320351. doi: 10.1371/journal.pone.0320351 (PMC11936205; doi:10.1371/journal.pone.0320351)
Supplement: S4 Table — (DOCX) [file pone.0320351.s012.docx]

**S4 Table.** **Raw data used in current meta-analysis.** (Name of data extractors: Jiawei Qi and Wenwen Wu; Date of data extraction: July 10, 2024. All authors confirmed study eligibility).

| **Study** | **Outcome** | |  | **Early CRRT group** | | | | | **Delayed CRRT group** | | | | |  |
| --- | --- | --- | --- | --- | --- | --- | --- | --- | --- | --- | --- | --- | --- | --- |
|  |  | |  | Events | | | | Total | Events | | | | Total |  |
| **Bouman 2002** | 28-day mortality | |  | 11 | | | | 35 | 9 | | | | 36 |  |
| **Combes 2015** | 28-day mortality | |  | 40 | | | | 112 | 40 | | | | 112 |  |
| **Geri 2019** | 28-day mortality | |  | 11 | | | | 17 | 13 | | | | 18 |  |
| **Lumlertgul 2018** | 28-day mortality | |  | 36 | | | | 58 | 35 | | | | 60 |  |
| **Srisawat 2018** | 28-day mortality | |  | 10 | | | | 20 | 9 | | | | 20 |  |
| **Xia 2019** | 28-day mortality | |  | 15 | | | | 30 | 13 | | | | 30 |  |
| **Yang 2019** | 28-day mortality | |  | 27 | | | | 71 | 39 | | | | 71 |  |
| **Yin 2018** | 28-day mortality | |  | 6 | | | | 33 | 6 | | | | 30 |  |
| **Zarbock 2016** | 28-day mortality | |  | 34 | | | | 112 | 48 | | | | 119 |  |
|  |  | |  | **Early CRRT group** | | | | | **Delayed CRRT group** | | | | |  |
|  |  | |  | Events | | Total | | | Events | | | Total | |  |
| **An 2021** | 60-day mortality | |  | 18 | | 78 | | | 33 | | | 78 | |  |
| **Combes 2015** | 60-day mortality | |  | 48 | | 112 | | | 42 | | | 112 | |  |
| **Lumlertgul 2018** | 60-day mortality | |  | 45 | | 58 | | | 44 | | | 60 | |  |
| **Yin 2018** | 60-day mortality | |  | 12 | | 33 | | | 9 | | | 30 | |  |
| **Zarbock 2016** | 60-day mortality | |  | 43 | | 112 | | | 60 | | | 119 | |  |
|  |  | |  | **Early CRRT group** | | | | | **Delayed CRRT group** | | | | |  |
|  |  | |  | Events | | Total | | | Events | | Total | | |  |
| **Combes 2015** | 90-day mortality | |  | 51 | | 112 | | | 43 | | 112 | | |  |
| **Lumlertgul 2018** | 90-day mortality | |  | 47 | | 58 | | | 44 | | 60 | | |  |
| **Yin 2018** | 90-day mortality | |  | 12 | | 33 | | | 10 | | 30 | | |  |
| **Zarbock 2016** | 90-day mortality | |  | 44 | | 112 | | | 65 | | 119 | | |  |
|  |  | |  | **Early CRRT group** | | | | | **Delayed CRRT group** | | | | |  |
|  |  | |  | Events | | Total | | | Events | | Total | | |  |
| **Geri 2019** | 14-day mortality | |  | 11 | | 17 | | | 13 | | 18 | | |  |
| **Payen 2009** | 14-day mortality | |  | 20 | | 37 | | | 17 | | 39 | | |  |
| **Sugahara 2004** | 14-day mortality | |  | 2 | | 14 | | | 12 | | 14 | | |  |
|  |  | |  | **Early CRRT group** | | | | | **Delayed CRRT group** | | | | |  |
|  |  | |  | Events | | Total | | | Events | | Total | | |  |
| **Bouman 2002** | Hospital mortality |  | | 18 | | 35 | | | 14 | | 36 | | |  |
| **Combes 2015** | Hospital mortality |  | | 50 | | 112 | | | 44 | | 112 | | |  |
| **Lumlertgul 2018** | Hospital mortality |  | | 43 | | 58 | | | 41 | | 60 | | |  |
| **Zarbock 2016** | Hospital mortality | |  | | 41 | | 112 | | 56 | 119 | | | |  |
|  |  | |  | **Early CRRT group** | | | | | **Delayed CRRT group** | | | | |  |
|  |  | |  | Events | | Total | | | Events | | Total | | |  |
| **Bouman 2002** | Number of patients receiving RRT | |  | 35 | | 35 | | | 30 | | 36 | | |  |
| **Combes 2015** |  |  |  | 111 | | 112 | | | 64 | | 112 | | |  |
| **Lumlertgul 2018** |  |  |  | 57 | | 58 | | | 45 | | 60 | | |  |
| **Srisawat 2018** | Number of patients receiving RRT | |  | 20 | | | | 20 | 8 | | | | 20 |  |
| **Zarbock 2016** |  |  |  | 112 | | | | 112 | 108 | | | | 119 |  |
|  |  | |  | **Early CRRT group** | | | | | **Delayed CRRT group** | | | | |  |
|  |  | |  | Events | | | | Total | Events | | | | Total |  |
| **Combes 2015** | Number of patients dependent on RRT at day 28 | |  | 6 | | | | 72 | 2 | | | | 72 |  |
| **Lumlertgul 2018** |  |  |  | 7 | | | | 22 | 10 | | | | 25 |  |
| **Srisawat 2018** |  |  |  | 1 | | | | 10 | 6 | | | | 11 |  |
| **Sugahara 2004** |  |  |  | 2 | | | | 12 | 0 | | | | 2 |  |
| **Xia 2019** |  |  |  | 2 | | | | 15 | 12 | | | | 17 |  |
| **Zarbock 2016** |  |  |  | 18 | | | | 78 | 26 | | | | 71 |  |
|  |  | |  | **Early CRRT group** | | | | | **Delayed CRRT group** | | | | |  |
|  |  | |  | Events | | Total | | | Events | | | Total | |  |
| **Bouman 2002** | Number of patients dependent on RRT at discharge | |  | 1 | | 17 | | | 0 | | | 22 | |  |
| **Combes 2015** |  |  |  | 2 | | 62 | | | 2 | | | 68 | |  |
| **Lumlertgul 2018** |  |  |  | 3 | | 15 | | | 6 | | | 19 | |  |
| **Zarbock 2016** |  |  |  | 9 | | 70 | | | 18 | | | 59 | |  |
|  |  | | **Early CRRT group** | | | | | | **Delayed CRRT group** | | | | | |
|  |  | | Mean | SD | | Total | | | Mean | | SD | | | Total |
| **Bouman 2002** | Length of stay in the ICU | | 13.0 | 11.9 | | 35 | | | 13.5 | | 11.7 | | | 36 |
| **Lumlertgul 2018** |  |  | 12.0 | 14.1 | | 58 | | | 13.5 | | 14.8 | | | 60 |
| **Yang 2019** |  |  | 10.6 | 1.6 | | 71 | | | 15.3 | | 2.0 | | | 71 |
| **Yin 2018** |  |  | 13.7 | 8.7 | | 33 | | | 14.9 | | 9.8 | | | 30 |
| **Zarbock 2016** |  |  | 19.0 | 14.8 | | 112 | | | 22.0 | | 17.8 | | | 119 |
|  |  | | **Early CRRT group** | | | | | | **Delayed CRRT group** | | | | | |
|  |  | | Mean | SD | | Total | | | Mean | | SD | | | Total |
| **Bouman 2002** | Length of stay in the hospital | | 27.0 | 20.7 | | 35 | | | 35.5 | | 38.5 | | | 36 |
| **Lumlertgul 2018** |  |  | 29.7 | 35.5 | | 58 | | | 32.6 | | 34.9 | | | 60 |
| **Yin 2018** |  |  | 27.0 | 16.0 | | 33 | | | 29.2 | | 15.9 | | | 30 |
| **Zarbock 2016** |  |  | 44.2 | 41.9 | | 112 | | | 64.6 | | 70.6 | | | 119 |
|  |  | | **Early CRRT group** | | | | | | **Delayed CRRT group** | | | | | |
|  |  | | Mean | SD | | Total | | | Mean | | SD | | | Total |
| **Bouman 2002** | Duration of mechanical ventilation | | 11.0 | 11.1 | | 35 | | | 12.0 | | 10.4 | | | 36 |
| **Combes 2015** |  |  | 7.0 | 8.1 | | 112 | | | 6.0 | | 6.7 | | | 112 |
| **Yin 2018** |  |  | 4.8 | 2.0 | | 33 | | | 9.1 | | 6.6 | | | 30 |
| **Zarbock 2016** |  |  | 5.2 | 5.0 | | 112 | | | 7.5 | | 10.7 | | | 119 |
|  |  | |  | **Early CRRT group** | | | | | **Delayed CRRT group** | | | | |  |
|  |  | |  | Events | | | | Total | Events | | | | Total |  |
| **Combes 2015** | Hypotension | |  | 87 | | | | 112 | 74 | | | | 112 |  |
| **Lumlertgul 2018** | Hypotension | |  | 20 | | | | 58 | 12 | | | | 60 |  |
| **Zarbock 2016** | Hypotension | |  | 2 | | | | 112 | 1 | | | | 119 |  |
|  |  | |  | **Early CRRT group** | | | | | **Delayed CRRT group** | | | | |  |
|  |  | |  | Events | | | | Total | Events | | | | Total |  |
| **Bouman 2002** | Thrombocytopenia | |  | 3 | | | | 35 | 2 | | | | 36 |  |
| **Combes 2015** | Thrombocytopenia | |  | 56 | | | | 112 | 37 | | | | 112 |  |
| **Yin 2018** | Thrombocytopenia | |  | 1 | | | | 33 | 0 | | | | 30 |  |
|  |  | |  | **Early CRRT group** | | | | | **Delayed CRRT group** | | | | |  |
|  |  | |  | Events | | Total | | | Events | | | Total | |  |
| **Combes 2015** | Hypophosphatemia | |  | 57 | | 112 | | | 19 | | | 112 | |  |
| **Lumlertgul 2018** | Hypophosphatemia | |  | 13 | | 58 | | | 2 | | | 60 | |  |
|  |  | |  | **Early CRRT group** | | | | | **Delayed CRRT group** | | | | |  |
|  |  | |  | Events | | Total | | | Events | | | Total | |  |
| **Lumlertgul 2018** | Arrhythmias | |  | 21 | | 58 | | | 16 | | | 60 | |  |
| **Zarbock 2016** | Arrhythmias | |  | 1 | | 112 | | | 0 | | | 119 | |  |
|  |  | |  | **Early CRRT group** | | | | | **Delayed CRRT group** | | | | |  |
|  |  | |  | Events | | Total | | | Events | | Total | | |  |
| **Lumlertgul 2018** | Hypocalcemia | |  | 4 | | 58 | | | 4 | | 60 | | |  |
| **Zarbock 2016** | Hypocalcemia | |  | 75 | | 112 | | | 71 | | 119 | | |  |
|  |  | |  | **Early CRRT group** | | | | | **Delayed CRRT group** | | | | |  |
|  |  | |  | Events | | Total | | | Events | | Total | | |  |
| **Bouman 2002** | Bleeding events | |  | 7 | | 35 | | | 3 | | 36 | | |  |
| **Combes 2015** | Bleeding events | |  | 35 | | 112 | | | 34 | | 112 | | |  |
| **Lumlertgul 2018** | Bleeding events | |  | 1 | | 58 | | | 3 | | 60 | | |  |
|  | | | | | | | | | | | | | | |
