# Supplementary material for: Selection of timing of continuous renal replacement therapy in patients with acute kidney injury: A meta-analysis of randomized controlled trials
Source: PLoS One. 2025 Mar 25;20(3):e0320351. doi: 10.1371/journal.pone.0320351 (PMC11936205; doi:10.1371/journal.pone.0320351)
Supplement: S5 Table — (DOCX) [file pone.0320351.s013.docx]

**S5 Table.** **Assessment of certainty of evidence using the GRADE approach for included outcomes.**

| **Quality assessment** | | | | | | | | | | | **Effect** | |  |
| --- | --- | --- | --- | --- | --- | --- | --- | --- | --- | --- | --- | --- | --- |
| No. of  studies | | Risk of bias | | Inconsistency | Indirectness | | Imprecision | | Other | | Point estimate  (95% CI) | | Quality |
| 28-day mortality | | | | | | | | | | | | | |
| 9 | | Not serious | | Not serious | Not serious | | Not serious | | None | | RR = 0.91  (0.79 to 1.06) | | HIGH |
| 60-day mortality | | | | | | | | | | | | | |
| 5 | | Not serious | | Serious^1^ | Not serious | | Not serious | | None | | RR = 0.91  (0.70 to 1.17) | | Moderate |
| 90-day mortality | | | | | | | | | | | | | |
| 4 | | Not serious | | Serious^1^ | Not serious | | Not serious | | None | | RR = 1.00 (0.77 to 1.28) | | Moderate |
| 14-day mortality | | | | | | | | | | | | | |
| 3 | | Not serious | | Serious^1^ | Not serious | | Serious^2^ | | None | | RR = 0.73 (0.34 to 1.57) | | Low |
| Hospital mortality | | | | | | | | | | | | | |
| 4 | Not serious | | Not serious | | Not serious | Not serious | | None | | RR = 1.01 (0.86 to 1.19) | | HIGH | |
| Number of patients receiving RRT | | | | | | | | | | | | | |
| 5 | | Not serious | | Serious^1^ | Not serious | | Not serious | | None | | RR = 1.41 (1.09 to 1.83) | | Moderate |
| Number of patients dependent on RRT at day 28 | | | | | | | | | | | | | |
| 6 | | Not serious | | Serious^1^ | Not serious | | Serious^2^ | | None | | RR = 0.62 (0.33 to 1.18) | | Low |
| Number of patients dependent on RRT at discharge | | | | | | | | | | | | | |
| 4 | | Not serious | | Not serious | Not serious | | Serious^3^ | | None | | RR = 0.57 (0.32 to 0.99) | | Moderate |
| Length of stay in the ICU | | | | | | | | | | | | | |
| 5 | | Not serious | | Not serious | Not serious | | Serious^2^ | | None | | MD = -3.24 (-5.14 to -1.35) | | Moderate |
| Length of stay in the hospital | | | | | | | | | | | | | |
| 4 | | Not serious | | Not serious | Not serious | | Serious^2^ | | None | | MD = -7.00  (-14.60 to 0.60) | | Moderate |
| Duration of mechanical ventilation | | | | | | | | | | | | | |
| 4 | | Not serious | | Serious^1^ | Not serious | | Serious^2^ | | None | | MD = -1.67 (-4.24 to 0.91) | | Low |
| Hypotension | | | | | | | | | | | | | |
| 3 | | Not serious | | Not serious | Not serious | | Not serious | | None | | RR = 1.26  (1.06 to 1.50) | | HIGH |
| Thrombocytopenia | | | | | | | | | | | | | |
| 3 | | Not serious | | Not serious | Not serious | | Not serious | | None | | RR = 1.53  (1.11 to 2.10) | | HIGH |
| Hypophosphatemia | | | | | | | | | | | | | |
| 2 | | Not serious | | Not serious | Not serious | | Not serious | | None | | RR = 3.35  (2.18 to 5.15) | | HIGH |
| Arrhythmias | | | | | | | | | | | | | |
| 2 | | Not serious | | Not serious | Not serious | | Serious^3^ | | None | | RR = 1.41 (0.83 to 2.41) | | Moderate |
| Hypocalcemia | | | | | | | | | | | | | |
| 2 | | Not serious | | Not serious | Not serious | | Not serious | | None | | RR = 1.12 (0.92 to 1.36) | | HIGH |
| Bleeding events | | | | | | | | | | | | | |
| 3 | | Not serious | | Not serious | Not serious | | Not serious | | None | | RR = 1.08 (0.75 to 1.56) | | HIGH |

RRT, renal replacement therapy; ICU, intensive care unit; RR, risk ratio; CI, confidence interval.

^1^High I^2^ value

^2^Confidence interval too wide

^3^Imprecision owing to fewer incidents observed
