# Supplementary material for: Selection of timing of continuous renal replacement therapy in patients with acute kidney injury: A meta-analysis of randomized controlled trials
Source: PLoS One. 2025 Mar 25;20(3):e0320351. doi: 10.1371/journal.pone.0320351 (PMC11936205; doi:10.1371/journal.pone.0320351)
Supplement: Attachment — Submitted filename: Response to Reviewers.docx [file pone.0320351.s015.docx]

**Responses to the Comments of Academic editor and Reviewers**

We would like to express our sincere gratitude to the editors and reviewers for their time and effort in reviewing our manuscript. We greatly appreciate their insightful comments, which have significantly contributed to the improvement of our work. Each comment has been carefully considered, and we have made the necessary revisions accordingly. Our responses are provided in blue, and the changes to the manuscript are highlighted in blue.

Academic editor:

Before we proceed with your manuscript, please address the following prompts:

a) If there are ethical or legal restrictions on sharing a de-identified data set, please explain them in detail (e.g., data contain potentially identifying or sensitive patient information, data are owned by a third-party organization, etc.) and who has imposed them (e.g., a Research Ethics Committee or Institutional Review Board, etc.). Please also provide contact information for a data access committee, ethics committee, or other institutional body to which data requests may be sent.

b) If there are no restrictions, please upload the minimal anonymized data set necessary to replicate your study findings to a stable, public repository and provide us with the relevant URLs, DOIs, or accession numbers. For a list of recommended repositories, please see

https://journals.plos.org/plosone/s/recommended-repositories. You also have the option of uploading the data as Supporting Information files, but we would recommend depositing data directly to a data repository if possible.

We will update your Data Availability statement on your behalf to reflect the information you provide.

RESPONSE: We sincerely apologize for the oversight regarding the required data. In our revised submission, we have included the necessary data as a supporting information file. Thank you for your understanding and continued support.

3. Please include captions for your Supporting Information files at the end of your manuscript, and update any in-text citations to match accordingly. Please see our Supporting Information guidelines for more information: <http://journals.plos.org/plosone/s/supporting-information>.

RESPONSE: In our revised manuscript, we have added the title of the “Supporting Information” files at the end and updated the in-text citations accordingly. Thank you for your guidance.

4. As required by our policy on Data Availability, please ensure your manuscript or supplementary information includes the following:

A numbered table of all studies identified in the literature search, including those that were excluded from the analyses.

For every excluded study, the table should list the reason(s) for exclusion.

If any of the included studies are unpublished, include a link (URL) to the primary source or detailed information about how the content can be accessed.

RESPONSE: In our revised submission, we have summarized a table that outlines the studies included in the literature search, those that were excluded, and the reasons for their exclusion (see Supporting Information file S2 Table).

A table of all data extracted from the primary research sources for the systematic review and/or meta-analysis. The table must include the following information for each study:

Name of data extractors and date of data extraction

Confirmation that the study was eligible to be included in the review.

All data extracted from each study for the reported systematic review and/or meta-analysis that would be needed to replicate your analyses.

If data or supporting information were obtained from another source (e.g. correspondence with the author of the original research article), please provide the source of data and dates on which the data/information were obtained by your research group.

RESPONSE: In our revised submission, we have included a table summarizing all data extracted from each study for the reported systematic review, in accordance with the specified requirements (see S4 Table in the Supporting Information file). Thank you for your consideration.

If applicable for your analysis, a table showing the completed risk of bias and quality/certainty assessments for each study or outcome.  Please ensure this is provided for each domain or parameter assessed. For example, if you used the Cochrane risk-of-bias tool for randomized trials, provide answers to each of the signalling questions for each study. If you used GRADE to assess certainty of evidence, provide judgements about each of the quality of evidence factor. This should be provided for each outcome.

RESPONSE: In our revised submission, we address each signaling question for every RCT using the Cochrane Randomized Trials Risk of Bias Tool for risk of bias assessment (see S3 Table in the Supporting Information file). Additionally, we have created a table to assess the quality of evidence for all outcomes using the GRADE approach, providing judgments on each factor influencing the quality of evidence (see S5 Table in the Supporting Information file). Thank you for your review.

An explanation of how missing data were handled.

RESPONSE: Thank you for your insightful comments. We would like to offer the following clarifications regarding the handling of missing data:

- **For dichotomous variables**: Analyses were conducted directly based on the results from the included studies, without the need for additional estimation.
- **For continuous variables**: Where feasible, we utilized the available data directly. In instances where only the median (MD) and interquartile range (IQR) were reported, we employed standard approximation methods to convert these into the mean (M) and standard deviation (SD) as follows:
  - MD ≈ M
  - IQR ≈ 1.35 × SD

We trust this explanation addresses your concerns. Thank you again for your valuable feedback.

Reviewer #1

Major:

1. My biggest comment relates to the rationale or justification for undertaking this review given the rich data available in previous and relatively recent high-quality reviews (see: https://pubmed.ncbi.nlm.nih.gov/33407756/ https://pubmed.ncbi.nlm.nih.gov/33407756/ and https://pubmed.ncbi.nlm.nih.gov/32334654/ as examples). If the rationale is that these reviews were not exclusively restricted to CRRT, it still begs the question as to why we think a special comparison of early vs. late CRRT is relevant to the reader. We know from prior studies and meta-analyses that the mode of dialysis in AKI has not shown to influence outcomes (for example: https://www.sciencedirect.com/science/article/abs/pii/S08839441173 01727) nor has the timing. Also, the Cochrane review includes a sub-analysis by mode. I am thus hard-pressed to understand what new information the current review is contributing.

The authors need to substantiate and provide a convincing rationale for undertaking this exercise.

RESPONSE: We appreciate your thorough review of our manuscript and your insightful comments. As you noted, one of the key reasons for conducting this meta-analysis was to examine continuous renal replacement therapy (CRRT). Previous studies and meta-analyses have indicated that the dialysis modality for acute kidney injury (AKI) does not significantly impact outcomes. Specifically, a secondary analysis of the AKIKI and IDEAL-ICU studies demonstrated that CRRT, when used as the first modality, showed no survival or kidney recovery benefit compared to intermittent hemodialysis (IHD) and may even be associated with less favorable outcomes in patients with milder disease severity (see: <https://pubmed.ncbi.nlm.nih.gov/35379300/> as examples).

Recent findings from a secondary analysis of the STARRT-AKI trial suggest that in critically ill patients with severe acute kidney injury (AKI), the initiation of continuous renal replacement therapy (CRRT) is associated with a significant reduction in the composite outcome of mortality or dependence on renal replacement therapy (RRT) at 90 days, compared to intermittent hemodialysis (IHD) (see: [https://pubmed.ncbi.nlm.nih.gov/37815560/](https://pubmed.ncbi.nlm.nih.gov/37815560/)). Furthermore, a larger population study demonstrated that after adjusting for potential confounders, the odds of RRT dependence at 90 days post-discharge among survivors of RRT for AKI were 30% lower for those initially treated with CRRT versus IHD. These results were consistent across multiple sensitivity analyses (see: [https://pubmed.ncbi.nlm.nih.gov/38460295/](https://pubmed.ncbi.nlm.nih.gov/38460295/)).

Consequently, there remains ongoing debate regarding the optimal modality of renal replacement therapy (RRT) for patients with acute kidney injury (AKI).

We analyzed inconsistencies in some of the results; early initiation of CRRT was associated with lower ICU length of stay and a significant reduction in RRT dependency at discharge. among the adverse effects, early CRRT was associated with a higher incidence of thrombocytopenia and hypophosphatemia compared with delayed CRRT.

The Cochrane review conducted subanalyses of selected outcomes related to continuous renal replacement therapy (CRRT) by dialysis modality, including death, death or non-recovery of kidney function at day 90, and recovery of renal function. However, it did not include subanalyses for other critical outcomes by dialysis modality, such as 28-day mortality, 60-day mortality, 14-day mortality, Number of patients dependent on RRT (at discharge and day 28), the number of patients treated with CRRT, length of stays in the ICU and hospital, mechanical ventilation duration, and various adverse events. Furthermore, the review encompassed only eight randomized controlled trials (RCTs) pertaining to CRRT. In our study, we incorporated a broader range of RCTs to evaluate the role of CRRT as an initial treatment for acute kidney injury (AKI), thereby expanding the scope of the analysis. Our objective was to comprehensively assess the impact of CRRT timing on the prognosis of patients with AKI.

2. Was the protocol registered on PROSPERO or an equivalent publicly available repository to evaluate the adherence to prespecified aims and analysis plans?

RESPONSE: We sincerely appreciate your constructive feedback regarding the limitations of our article. We regret that we have not yet pre-registered our study in PROSPERO or an equivalent public repository. However, we want to assure you that the search criteria, data extraction, inclusion and exclusion criteria, and data synthesis were rigorously conducted according to a predetermined plan established by our team.

In the revised manuscript, we have addressed the comments from the editors and reviewer 2 by comprehensively assessing the quality of evidence for all outcomes using the GRADEpro Guidelines Development Tools software. Additionally, we evaluated heterogeneity among studies through χ² tests and I² statistics, without altering our original results.

3. The authors mention they conducted the review in accordance with PRISMA guidelines (methods, lines 48 and 49): PRISMA is a guideline for reporting systematic reviews and not for the conduct. Please correct this.

Response: Thank you for your valuable feedback. In response to your suggestion, we have revised “doing this meta-analysis” to “reporting this meta-analysis.” We appreciate your insights and aim to ensure clarity in our work.

Minor:
1. How were disagreements resolved? (search strategy, lines 55 and 56)

RESPONSE: Thank you for your valuable comments. We appreciate your insights and apologize for any oversight. The search strategy was developed collaboratively between J.Q. and W.W., and we have now included a section detailing how disagreements were resolved in the revised manuscript. Thank you for your understanding and support.

2. Exclusion criteria: what do they mean by 'repeated analysis of experimental data'? (lines 63 and 64)

RESPONSE: Thank you for your valuable comments and for highlighting this important aspect of our methodology. The term “repeated analysis of experimental data” refers to the practice of evaluating or interpreting the same dataset multiple times within a study. This includes activities such as secondary data analysis, post hoc analyses, substudies, or Bayesian reanalysis of randomized controlled trials.（see: <https://pubmed.ncbi.nlm.nih.gov/36331570/> <https://pubmed.ncbi.nlm.nih.gov/29351007/> https://pubmed.ncbi.nlm.nih.gov/37592355/ and <https://pubmed.ncbi.nlm.nih.gov/36008827/> as examples）

Our exclusion criterion is designed to ensure that the datasets we analyze remain original and free from multiple interpretations, which could compromise the validity of our conclusions. By adhering to this criterion, we aim to enhance the integrity and reliability of our findings.

We appreciate your attention to this detail and hope this clarification adequately addresses your concerns.

3. Table 1: There seem to be issues with referencing and it shows up in the document as 'errors'. Please fix this.

RESPONSE: Thank you for your careful review and feedback on our manuscript. We note the issues you mentioned regarding the citation formatting of Table 1. We have double-checked and fixed all citation errors to ensure that they comply with the journal's formatting requirements.

4. Attention needs to be paid to consistency of decimal points throughout the manuscript. Authors should stick to 1 or 2 decimal places and be consistent.

RESPONSE: Thank you for your valuable feedback. In the revised manuscript, we have standardized the data presentation by adjusting all values to two decimal places, ensuring consistency throughout the document.

5. Lines 111: change 'main ending' to 'main outcome' or 'primary outcome'

RESPONSE: We sincerely appreciate your valuable feedback and have revised the term “main ending” to “main outcome” in the newly submitted manuscript. Thank you for your insightful suggestions.

6. Line 113: change 'composite mortality' to 'pooled mortality'

RESPONSE: We sincerely appreciate your valuable feedback. We have made the correction from 'composite mortality' to 'pooled mortality' in the revised manuscript. Thank you for your thoughtful suggestion.

7. Authors refer to 14-day, 60-day and 90-day mortality outcomes as rates. These are not really rates and are instead percentages or proportions. Please correct this.

RESPONSE:We sincerely appreciate your valuable comments. In the revised manuscript, we have corrected the terminology from "14-day, 60-day, and 90-day death rate" to "14-day, 60-day, and 90-day mortality" to accurately reflect the nature of these outcomes. Thank you for helping us enhance the clarity of our work.

8. Table 2 is best presented as a forest plot. Also what is the rationale for the break up of the age subgroup as less than 64 and more than 64 years? And how was the cut off for SOFA and APACHE defined as low and high?

**RESPONSE:** We sincerely appreciate your valuable suggestion and have revised Table 2 to present it as a forest plot (see S4 Fig, S5 Fig, and S6 Fig).

Regarding the age subgroup classification of less than 64 years and more than 64 years, we based this on the mean ages from the nine studies included in our analysis, which were 58.9, 59.5, 60.8, 66.4, 66.8, 67.0, 67.1, 67.3, and 68.5 years, respectively. To enhance our ability to detect age-related effects on outcomes, we grouped the first three studies (younger cohort) and the last six studies (older cohort) together. This led us to select age subgroup values of 64 years as a meaningful cut-off.

The definitions for low and high SOFA and APACHE II scores were established based on prior literature. For SOFA scores, we classified scores of ≥12 as high, as referenced in Gaudry et al. (see: [https://pubmed.ncbi.nlm.nih.gov/32334654/](https://pubmed.ncbi.nlm.nih.gov/32334654/)). For APACHE II scores of ≥25 were considered high, given that they indicate a significantly increased hospital mortality rate (>50%) in non-surgical ICU patients (see: <https://pubmed.ncbi.nlm.nih.gov/3928249/> Fig. 3).

Thank you once again for your constructive feedback.

9. in the PRISMA flow diagram, under 'screening' the authors report that they excluded 2443 studies- why? Please list the reasons.

**RESPONSE:** We sincerely appreciate your valuable comments. In our study, we utilized NoteExpress software to exclude 2443 records based on title and summary. The reasons for these exclusions are as follows:

- **Irrelevant studies**: 2295
- **Reviews and letters**: 43
- **Meta-analyses**: 33
- **Conference abstracts**: 25
- **Study protocols**: 31
- **Repeated analyses**: 14
- **Corrections**: 2

We have included these exclusion reasons in PRISMA 2020 flow diagram for clarity. Thank you for your understanding.

Reviewer #2:

The threshold value of I2 should not be used to determine random or fix models. The two methods have different assumptions and research aims. Need to evaluate more carefully the nature of studies and check whether they share a common effect.

**RESPONSE:** Thank you for your thorough review and insightful comments on our manuscript. Based on your suggestions, we have revised our assessment of inter-study heterogeneity. We will use a combined assessment of the chi-square test and I² statistic based on the following criteria: when a p-value of ≤0.1 or an I² ≥30% indicates substantial heterogeneity, we will use a random-effects model; on the contrary, a fixed-effects model will be used if the between-study heterogeneity is low. Thank you again for your valuable comments.

Table 1. Study column: Error. Reference source not found.

**RESPONSE:** We sincerely apologize for the oversight regarding the citation. The reference source has been corrected. Thank you for bringing this to our attention.

Figure 1 provide reasons for excluding 2443 records.

**RESPONSE:** We sincerely appreciate your valuable comments. In our study, we utilized NoteExpress software to exclude 2443 records based on title and summary. The reasons for these exclusions are as follows:

- **Irrelevant studies**: 2295
- **Reviews and letters**: 43
- **Meta-analyses**: 33
- **Conference abstracts**: 25
- **Study protocols**: 31
- **Repeated analyses**: 14
- **Corrections**: 2

We have included these exclusion reasons in PRISMA 2020 flow diagram for clarity. Thank you for your understanding.

Forest plot figures, Change the left column title as “Study” instead of “Study or Subgroup”.

**RESPONSE:** Thank you for your valuable comments. In response to your suggestion, we have revised the title of the left column in the forest plot from “Study or Subgroup” to “Study” in the resubmitted manuscript.

Capitalize the first letters of some studies.

**RESPONSE:** We appreciate your feedback and apologize for the oversight. In the resubmitted manuscript, we have corrected the initials of the studies in the forest map to ensure they are capitalized. Thank you for your valuable correction.
