# Supplementary material for: Selection of timing of continuous renal replacement therapy in patients with acute kidney injury: A meta-analysis of randomized controlled trials
Source: PLoS One. 2025 Mar 25;20(3):e0320351. doi: 10.1371/journal.pone.0320351 (PMC11936205; doi:10.1371/journal.pone.0320351)
Supplement: Attachment — Submitted filename: Response_to_Reviewers.docx [file pone.0320351.s016.docx]

**Responses to the Comments of Academic editor and Reviewers**

We thank the editors and reviewers for their time and effort in reviewing our manuscript. We appreciate their comments, which have helped improve our work. Each comment has been carefully considered, and the necessary revisions have been made, as required. Our responses are provided below in blue, and changes to the manuscript are highlighted in blue.

Academic editor:

Before we proceed with your manuscript, please address the following prompts:

a) If there are ethical or legal restrictions on sharing a de-identified data set, please explain them in detail (e.g., data contain potentially identifying or sensitive patient information, data are owned by a third-party organization, etc.) and who has imposed them (e.g., a Research Ethics Committee or Institutional Review Board, etc.). Please also provide contact information for a data access committee, ethics committee, or other institutional body to which data requests may be sent.

b) If there are no restrictions, please upload the minimal anonymized data set necessary to replicate your study findings to a stable, public repository and provide us with the relevant URLs, DOIs, or accession numbers. For a list of recommended repositories, please see

https://journals.plos.org/plosone/s/recommended-repositories. You also have the option of uploading the data as Supporting Information files, but we would recommend depositing data directly to a data repository if possible.

We will update your Data Availability statement on your behalf to reflect the information you provide.

**RESPONSE**: Thank you for bringing this to our attention. We confirm that there are no ethical or legal restrictions on sharing our dataset. We have now included the complete anonymized dataset as supporting information files, which contain all data necessary to replicate our analyses. While we considered uploading the dataset to a public repository, we chose to provide all data as supplementary materials to help ensure data accessibility to all readers. We are committed to ensuring data transparency and appreciate your guidance on this matter.

3. Please include captions for your Supporting Information files at the end of your manuscript, and update any in-text citations to match accordingly. Please see our Supporting Information guidelines for more information: <http://journals.plos.org/plosone/s/supporting-information>.

**RESPONSE**: Thank you for your comment. The manuscript has been reformatted, as per the journal guidelines, and supplementary information captions have been included at the end of the main manuscript file. In-text citation style has been updated, as required.

4. As required by our policy on Data Availability, please ensure your manuscript or supplementary information includes the following:

A numbered table of all studies identified in the literature search, including those that were excluded from the analyses.

For every excluded study, the table should list the reason(s) for exclusion.

If any of the included studies are unpublished, include a link (URL) to the primary source or detailed information about how the content can be accessed.

**RESPONSE**: Thank you for this recommendation. We have now included supplementary tables, which list all studies screened for this meta-analysis, including reasons for exclusion (S2 Table).

A table of all data extracted from the primary research sources for the systematic review and/or meta-analysis. The table must include the following information for each study:

Name of data extractors and date of data extraction

Confirmation that the study was eligible to be included in the review.

All data extracted from each study for the reported systematic review and/or meta-analysis that would be needed to replicate your analyses.

If data or supporting information were obtained from another source (e.g. correspondence with the author of the original research article), please provide the source of data and dates on which the data/information were obtained by your research group.

**RESPONSE:** Thank you for your guidance. We have included all this information in S4 Table. This table provides details on data extractor name, data extraction date, study eligibility criteria, and outcome-related data.

If applicable for your analysis, a table showing the completed risk of bias and quality/certainty assessments for each study or outcome.  Please ensure this is provided for each domain or parameter assessed. For example, if you used the Cochrane risk-of-bias tool for randomized trials, provide answers to each of the signalling questions for each study. If you used GRADE to assess certainty of evidence, provide judgements about each of the quality of evidence factor. This should be provided for each outcome.

**RESPONSE:** Thank you for your comment. We have now included information on the risk of bias assessment results for each included RCT. To achieve this, we have used the Cochrane Risk of Bias Tool, which evaluates key items, including random sequence generation, allocation concealment, blinding, incomplete outcome data, selective reporting, and other sources of bias (S3 Table). These assessments were conducted independently by two reviewers with disagreements resolved through consensus or consultation with a third reviewer. Additionally, we have compiled GRADE findings for each factor (risk of bias, inconsistency, indirectness, imprecision, and publication bias) across all pre-specified outcomes (S5 Table).

An explanation of how missing data were handled.

**RESPONSE**: Thank you for your query. Our approach to missing data was as follows:

- **For dichotomous variables**: Analyses were conducted directly based on the extracted data.
- **For continuous variables**: Where feasible, we utilized the available data directly. In instances where only the median (MD) and interquartile range (IQR) were reported, we used standard approximation methods to obtain the mean (M) and standard deviation (SD) as follows:
  - MD = M
  - IQR =1.35 × SD

Reviewer #1

Major:

1. My biggest comment relates to the rationale or justification for undertaking this review given the rich data available in previous and relatively recent high-quality reviews (see: https://pubmed.ncbi.nlm.nih.gov/33407756/ https://pubmed.ncbi.nlm.nih.gov/33407756/ and https://pubmed.ncbi.nlm.nih.gov/32334654/ as examples). If the rationale is that these reviews were not exclusively restricted to CRRT, it still begs the question as to why we think a special comparison of early vs. late CRRT is relevant to the reader. We know from prior studies and meta-analyses that the mode of dialysis in AKI has not shown to influence outcomes (for example: https://www.sciencedirect.com/science/article/abs/pii/S08839441173 01727) nor has the timing. Also, the Cochrane review includes a sub-analysis by mode. I am thus hard-pressed to understand what new information the current review is contributing.

The authors need to substantiate and provide a convincing rationale for undertaking this exercise.

**RESPONSE**: Thank you for your query and for highlighting the recent high-quality evidence on questions related to the one we aimed to answer in our study. The cited studies do in fact provide valuable evidence on the use of continuous renal replacement therapy (CRRT) and associated implications for acute kidney injury (AKI). However, we believe that our review supplements the existing evidence by clarifying the effects of early vs. late CRRT in patients with AKI. This study builds on previous research that examined this question, incorporating recent findings and thereby reaching conclusions of particular clinical relevance. We believe that a review focused on early vs. late initiation of CRRT, with updated data, is warranted for the following reasons:

1. **Findings of particular clinical relevance：**We found that early initiation of CRRT was associated with a short ICU stay and a significant reduction in RRT dependency at discharge, even if there was no significant reduction in the mortality rate.
2. **Recent Developments**: Recent studies have shown the advantages of early initiation of CRRT over intermittent hemodialysis (IHD) (see: <https://pubmed.ncbi.nlm.nih.gov/37815560/> and [https://pubmed.ncbi.nlm.nih.gov/38460295/)](https://pubmed.ncbi.nlm.nih.gov/38460295/),). If CRRT is better than IHD, then early IHD may not improve the prognosis of patients with AKI. However, the question remains: Does early CRRT improve the prognosis of patients with AKI? As a result, we incorporated additional studies and evaluated multiple outcomes.
3. **Subgroup Analyses in Existing Reviews**: To date, no meta-analysis has specifically investigated the impact of the timing of CRRT on AKI across all etiologies. While the cited Cochrane review does include a sub-analysis by modality, it only examines a limited number of outcome metrics and incorporates few studies related to CRRT. We believe that our study is relatively more focused, allowing to address this controversy with greater precision.
4. **Potential for Impact on Practice**: Despite a large body of evidence, the optimal timing for CRRT initiation remains unclear, and clinical practice varies widely. We believe that our review may support evidence-based clinical practice, potentially informing future development of clinical guidelines.

We have expanded the rationale section of the manuscript to clearly articulate our rationale and highlight the potential impact of our study.

As you noted, the primary objective of this meta-analysis was to evaluate the impact of early CRRT initiation on AKI. This may be of interest to readers for the following reasons.

1. Previous studies and meta-analyses have generally concluded that the dialysis modality for AKI does not impact outcomes. However, recent evidence suggests that early CRRT is superior to IHD in the treatment of patients with AKI.

For example, recent findings from the STARRT-AKI trial suggest that in critically ill patients with severe AKI, the initiation of CRRT is associated with a significant reduction in the composite outcome of mortality or dependence on renal replacement therapy (RRT) at 90 days, compared to IHD (<https://pubmed.ncbi.nlm.nih.gov/37815560/>). Additionally, a larger population-based study indicated that the odds of RRT dependence at 90 days post-discharge were 30% lower for those initially treated with CRRT than for those treated with IHD (<https://pubmed.ncbi.nlm.nih.gov/38460295/>).

These findings underscore the ongoing debate regarding the optimal modality of RRT for patients with AKI. If CRRT is better than IHD, then early IHD may not improve the prognosis of patients with AKI. However, the question remains: Does early CRRT improve the prognosis of patients with AKI? As a result, we incorporated additional studies and evaluated multiple outcomes. Our meta-analysis sought to resolve this question by specifically examining the timing of CRRT initiation and its implications for outcomes. Notably, we found that early initiation of CRRT was associated with a short ICU stay and a significant reduction in RRT dependency at discharge. Among adverse effects, early CRRT was linked with a higher incidence of thrombocytopenia and hypophosphatemia, compared with those associated with delayed CRRT.

While the Cochrane review conducted sub-analyses of selected outcomes related to CRRT by dialysis modality (e.g., death or non-recovery of kidney function at day 90, recovery of renal function), it did not include sub-analyses for several clinically important outcomes by dialysis modality. These include 28-day mortality, 60-day mortality, 14-day mortality, the number of patients dependent on RRT (at discharge and day 28), ICU and hospital lengths of stay, mechanical ventilation duration, and various adverse events. Furthermore, the Cochrane review included only eight RCTs pertaining to CRRT.

In contrast, our review incorporated a broader range of RCTs and outcomes to provide a more comprehensive evaluation of CRRT as an initial treatment for AKI. By addressing critical outcomes and expanding the evidence base, we aim to provide a more nuanced understanding of the impact of CRRT timing on the prognosis of patients with AKI. This focus will enable us to bridge important gaps in the literature and offer new insights into CRRT use in clinical practice.

2. Was the protocol registered on PROSPERO or an equivalent publicly available repository to evaluate the adherence to prespecified aims and analysis plans?

**RESPONSE**: Thank you for your query. We regret that this study was not pre-registered in PROSPERO or an equivalent public repository. This was due to an oversight, and we acknowledge this as a limitation of our study.

Despite the lack of formal pre-registration, our protocol followed the rigorous criteria presented by the PRISMA guidelines, which were defined before the study started and remained unchanged throughout the study, including, search strategies, study eligibility criteria, and data management and synthesis protocols. The steps undertaken were also recorded throughout the study to ensure protocol adherence.

3. The authors mention they conducted the review in accordance with PRISMA guidelines (methods, lines 48 and 49): PRISMA is a guideline for reporting systematic reviews and not for the conduct. Please correct this.

**RESPONSE**: Thank you for highlighting this nuance, which was misrepresented in our manuscript. We have now corrected this statement.

Minor:
1. How were disagreements resolved? (search strategy, lines 55 and 56)

**RESPONSE**: Thank you for your query. Disagreements were resolved by discussion between two reviewers. We have included this information in the revised manuscript.

2. Exclusion criteria: what do they mean by 'repeated analysis of experimental data'? (lines 63 and 64)

**RESPONSE**: Thank you for your query. The term “repeated analysis of experimental data” refers to the practice of evaluating or interpreting the same dataset multiple times within a study. This includes activities such as secondary data analysis, post-hoc analyses, sub-studies, or Bayesian reanalysis of randomized controlled trials (e.g., <https://pubmed.ncbi.nlm.nih.gov/36331570/>, <https://pubmed.ncbi.nlm.nih.gov/29351007/>, https://pubmed.ncbi.nlm.nih.gov/37592355/, and <https://pubmed.ncbi.nlm.nih.gov/36008827/>). In other words, these are studies where existing datasets have been reanalyzed to test new hypotheses. Such studies may introduce bias if overrepresented in a meta-analysis and were thus excluded from our study. We believe that this decision has helped ensure the validity and generalizability of our findings.

3. Table 1: There seem to be issues with referencing and it shows up in the document as 'errors'. Please fix this.

**RESPONSE**: Thank you for bringing these errors to our attention. We believe these errors have now been fixed and should not affect the legibility of Table 1. The manuscript has been reformatted as per the journal requirements.

4. Attention needs to be paid to consistency of decimal points throughout the manuscript. Authors should stick to 1 or 2 decimal places and be consistent.

**RESPONSE**: Thank you for your valuable feedback. In the revised manuscript, we have standardized the data presentation by adjusting all values to two decimal places, ensuring consistency throughout the document.

5. Lines 111: change 'main ending' to 'main outcome' or 'primary outcome'

**RESPONSE**: Thank you for your feedback. We have changed the term “main ending” to “main outcome” in the revised version of the manuscript.

6. Line 113: change 'composite mortality' to 'pooled mortality'

**RESPONSE**: Thank you for your comment. We have made this change to the terminology in the revised manuscript.

7. Authors refer to 14-day, 60-day and 90-day mortality outcomes as rates. These are not really rates and are instead percentages or proportions. Please correct this.

**RESPONSE**: Thank you for your comment. In the revised manuscript, we have corrected the terminology from "14-day, 60-day, and 90-day death rate" to "14-day, 60-day, and 90-day mortality" to accurately reflect the nature of these outcomes as percentages or proportions rather than rates.

8. Table 2 is best presented as a forest plot. Also what is the rationale for the break up of the age subgroup as less than 64 and more than 64 years? And how was the cut off for SOFA and APACHE defined as low and high?

**RESPONSE:** Thank you for your suggestion. We have revised Table 2 to present the data as forest plots (S4 Fig, S5 Fig, and S6 Fig in the revised manuscript).

The age cutoff in this study was based on the mean ages reported in the nine studies included in our analysis, which were 58.9, 59.5, 60.8, 66.4, 66.8, 67.0, 67.1, 67.3, and 68.5 years. To better detect age-related effects on outcomes, we grouped together the first three studies (mean age <64 years, representing the younger cohort) and the remaining six studies (mean age ≥64 years, representing the older cohort). While 64 years may not represent a universally established clinical threshold, this data-driven approach was adopted to enhance the comparability and interpretability of age-related subgroup analyses.

The thresholds for low and high SOFA and APACHE II scores were established based on the literature. SOFA scores of ≥12 were classified as high, consistent with the criteria used in Gaudry et al. (<https://pubmed.ncbi.nlm.nih.gov/32334654/>), which identified this threshold as indicative of severe organ dysfunction. For APACHE II, scores of ≥25 were considered high, reflecting significantly increased hospital mortality rates (>50%) in non-surgical ICU patients, as reported in Knaus et al. (<https://pubmed.ncbi.nlm.nih.gov/3928249/> Fig 3). These thresholds align with existing evidence and were selected to meaningfully stratify disease severity in our analysis.

9. in the PRISMA flow diagram, under 'screening' the authors report that they excluded 2443 studies- why? Please list the reasons.

**RESPONSE:** Thank you for your query. In our study, we used NoteExpress software to exclude 2,443 records based on title and summary. The reasons for these exclusions are as follows:

- **Irrelevant studies**: 2,295
- **Reviews and letters**: 43
- **Meta-analyses**: 33
- **Conference abstracts**: 25
- **Study protocols**: 31
- **Repeated analyses**: 14
- **Corrections**: 2

We have included these reasons for exclusion in the PRISMA 2020 flow diagram for clarity.

Reviewer #2:

The threshold value of I2 should not be used to determine random or fix models. The two methods have different assumptions and research aims. Need to evaluate more carefully the nature of studies and check whether they share a common effect.

**RESPONSE:** Thank you for your comment, which has led us to revise our approach to assessing heterogeneity among studies to include both the chi-square test and I² statistic. We have done this as follows: When the p-value was greater than 0.10, and I^2^ was less than 30%, we used the fixed-effects model; in contrast, when the p-value was less than or equal to 0.10, or I^2^ was greater than or equal to 30%, we used the random-effects model. This adjustment did not affect the results. We have updated the Methods section of the manuscript to reflect these changes.

Table 1. Study column: Error. Reference source not found.

**RESPONSE:** We apologize for this oversight. The referenced source has been corrected. Thank you for bringing this to our attention.

Figure 1 provide reasons for excluding 2443 records.

**RESPONSE:** Thank you for your query. In our study, we used NoteExpress software to exclude 2,443 records based on title and summary. The reasons for these exclusions are as follows:

- **Irrelevant studies**: 2,295
- **Reviews and letters**: 43
- **Meta-analyses**: 33
- **Conference abstracts**: 25
- **Study protocols**: 31
- **Repeated analyses**: 14
- **Corrections**: 2

We have included these reasons for exclusion in the PRISMA 2020 flow diagram for clarity.

Forest plot figures, Change the left column title as “Study” instead of “Study or Subgroup”.

**RESPONSE:** Thank you for your comment. In response to your suggestion, we have revised the title of the left column in all forest plot figures from “Study or Subgroup” to “Study” in the resubmitted manuscript. This change has been applied consistently across all relevant figures to enhance clarity and align with your recommendation.

Capitalize the first letters of some studies.

**RESPONSE:** In the resubmitted manuscript, we have ensured that the first letters of all study titles in the forest plot figures are appropriately capitalized. These corrections have been implemented consistently across all forest plots to improve accuracy and presentation. Thank you for bringing this to our attention.
